# Establishment of a Patient-Derived Xenograft Model of Colorectal Cancer in CIEA NOG Mice and Exploring Smartfish Liquid Diet as a Source of Omega-3 Fatty Acids

**DOI:** 10.3390/biomedicines9030282

**Published:** 2021-03-10

**Authors:** Helle Samdal, Lene C Olsen, Knut S Grøn, Elin S Røyset, Therese S Høiem, Ingunn Nervik, Pål Sætrom, Arne Wibe, Svanhild A Schønberg, Caroline H H Pettersen

**Affiliations:** 1 Department of Computer Science, Faculty of Information Technology and Electrical Engineering, Norwegian University of Science and Technology (NTNU), 7491 Trondheim, Norway; helle.samdal@ntnu.no (H.S.); pal.satrom@ntnu.no (P.S.); 2 Department of Clinical and Molecular Medicine, Faculty of Medicine and Health Sciences, Norwegian University of Science and Technology (NTNU), 7491 Trondheim, Norway; lene.c.olsen@ntnu.no (L.C.O.); knut.s.gron@ntnu.no (K.S.G.); elin.s.royset@ntnu.no (E.S.R.); therese.s.hoiem@ntnu.no (T.S.H.); ingunn.nervik@ntnu.no (I.N.); arne.wibe@ntnu.no (A.W.); svanhild.schonberg@ntnu.no (S.A.S.); 3 Bioinformatics Core Facility—BioCore, Norwegian University of Science and Technology (NTNU), 7491 Trondheim, Norway; 4 Department of Pathology, St. Olav’s University Hospital, N-7006 Trondheim, Norway; 5 K.G. Jebsen Center for Genetic Epidemiology, Norwegian University of Science and Technology (NTNU), 7491 Trondheim, Norway; 6 Department of Surgery, St. Olav’s University Hospital, N-7006 Trondheim, Norway

**Keywords:** PDX, patient-derived xenograft, CRC, colorectal cancer, omega-3 fatty acids

## Abstract

Cancer patient-derived xenografts (PDXs) better preserve tumor characteristics and microenvironment than traditional cancer cell line derived xenografts and are becoming a valuable model in translational cancer research and personalized medicine. We have established a PDX model for colorectal cancer (CRC) in CIEA NOG mice with a 50% engraftment rate. Tumor fragments from patients with CRC (*n* = 5) were engrafted in four mice per tumor (*n* = 20). Mice with established PDXs received a liquid diet enriched with fish oil or placebo, and fatty acid profiling was performed to measure fatty acid content in whole blood. Moreover, a biobank consisting of tissue and blood samples from patients was established. Histology, immunohistochemistry and in situ hybridization procedures were used for staining of tumor and xenograft tissue slides. Results demonstrate that key histological characteristics of the patients’ tumors were retained in the established PDXs, and the liquid diets were consumed as intended by the mice. Some of the older mice developed lymphomas that originated from human Ki67^+^, CD45^+^, and EBV^+^ lymphoid cells. We present a detailed description of the process and methodology, as well as possible issues that may arise, to refine the method and improve PDX engraftment rate for future studies. The established PDX model for CRC can be used for exploring different cancer treatment regimes, and liquid diets enriched with fish oil may be successfully delivered to the mice through the drinking flasks.

## 1. Introduction

In preclinical studies there has been a tradition for establishing cancer xenografts in immunosuppressed mice using cancer-derived cell lines (CDX). However, the results from CDX studies often do not correlate with the results from clinical studies, partly because cancer cell lines fail to represent the true complexity and heterogeneity of tumors (reviewed in [1,2,3]). This has led to a need for preclinical experimental models that better reflect the clinical situation. Patient-derived xenograft (PDX) models were first described in the 1970s [4,5], and the models have been refined over the last few decades. PDX models reflect more of the heterogeneity and individual differences of tumors compared to traditional preclinical CDX models (reviewed in [1,2,3,6]). However, the logistics related to the establishment of a PDX study are challenging, and there is a need for more literature guiding the initiation of PDX models.

The successful establishment of PDXs depends on several factors. The choice of animal model is important, and immunodeficient mice are found to have a higher engraftment rate for xenografts from foreign tissue as their lack of functional immune system would refrain from interfering with the foreign engrafted substance. Immunodeficient NOD/SCID/IL2Rg null (NOG) mice lack functional natural killer cells as well as B and T lymphocytes, and have defective macrophages, complement activity and dendritic cells [7]. This makes the NOG mouse an appropriate host model for establishment of PDXs, with improved tumor take rates compared to previous NOD, SCID and nude mice ([7], reviewed in [6,8]). The disruption of the interleukin 2 receptor subunit gamma (*IL2Rg*) gene reduces the chance of spontaneous lymphoma development in these animals, which was a known problem in previous NOD/SCID models [9,10]. Other important factors affecting the successful establishment of PDX models include the characteristics of the tumor subtypes, the site of implantation, the viability of the tumor cells, metastatic potential, preoperative patient treatment, contamination level in tumor specimens, time from tumor removal to implantation, the surgical procedure technique (reviewed in [1]), as well as tissue acquisition strategy [11].

In this study we aim at describing the procedure for the establishment of a PDX model of colorectal cancer (CRC). The outcome of CRC has improved significantly over the past few decades. However, CRC is still the second and third most common cancer type worldwide among women and men, respectively [12], and the second most common cancer type in Norway among both sexes [13]. Research related to improved treatment, and especially personalized treatment, for CRC patients is therefore highly needed. PDX models are currently the preferred model for preclinical studies in CRC, and studies have found the successful PDX engraftment rate to be between 56–87.5% (reviewed in [1]). We have performed a pilot study on the establishment of a PDX model for CRC in immunosuppressed CIEA NOG mice with the aim to perform preclinical combination studies of components with anticancer potential, such as cytostatic and designed diets with omega-3 polyunsaturated fatty acids (PUFAs). Combined with biobanking of healthy- and tumor tissue for whole exome- and RNA sequencing, as well as protein analyses, blood samples, and extensive clinical data via patient journals and cancer registers (e.g., the Norwegian Cancer Registry), the PDX model may be useful in the search for novel molecular biomarkers predicting responses to different anticancer drugs. The focus of this paper is to report the establishment of a PDX procedure and to test a delivery method for an omega-3 fatty acid enriched diet in mice.

## 2. Experimental Section

### 2.1. Study Design

**Figure 1:**
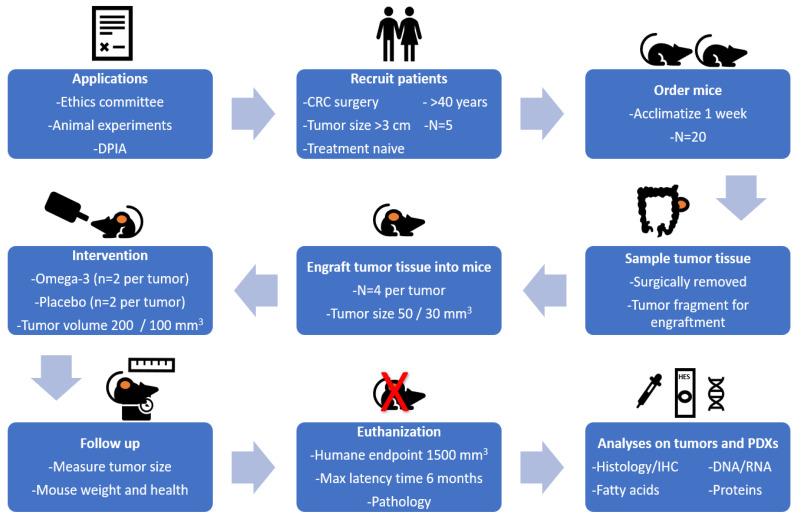
Study design for the establishment of patient-derived xenografts (PDXs) for colorectal cancer (CRC) in immune suppressed CIEA NOG mice.

Figure 1 gives an overview of the study design for establishing PDXs for CRC in immune suppressed mice. Surgically removed tumor fragments from 5 patients with CRC were engrafted into 4 mice per tumor. When the PDX growth was established, mice were provided with the intervention (omega-3 fatty acid (FA) or placebo enriched nutrition drinks). Follow up of the mice included assessment of animal health and weight, as well as tumor size. Mice were euthanized at humane endpoint defined by tumor volume, max latency time, and pathology, or after eight weeks treatment. Tumor and blood samples were collected for indicated analyses.

### 2.2. Patient Characteristics and Inclusion Criteria

Patients enrolled in the study were scheduled for cancer surgery at St. Olav’s University Hospital in Trondheim between April and September 2019. Medical records of patients scheduled for consultation at the preoperative clinic at St. Olav’s University Hospital were assessed. Patients were included based on the following criteria; clinically verified colon- or rectal cancer stage 1–4, tumor size exceeding 3 cm in diameter, and age > 40 years with no preoperative treatment. After signing an informed consent form, data regarding gender, age, diagnosis, tumor type, stage of disease, prior cancer treatment, use of lipid modifying medicaments, and intake of fish, cod liver oil, and omega-3 supplements were collected. Five patients who met the inclusion criteria following preoperative evaluation were included in this study. The study was approved by the Regional ethics committee for central Norway (REC ID 2017/2048, date: 10 October 2018). A Data Protection Impact Assessment (DPIA) for the project was performed in cooperation with the Norwegian Centre for Research Data (NSD) and was approved by both St. Olav’s University Hospital and NTNU. Data were securely stored at the HEMIT net at St. Olav’s University Hospital, locked by 2 step authorization by chip and password.

### 2.3. Patient Blood Sampling

Blood samples and related information were collected, processed and stored (tubes preassigned cryptic barcode from the Biobyte^®^ system (Biobank1^®^, Trondheim, Norway)) by Biobank1^®^. To minimize the blood sampling burden of the patients, blood samples for the study was sampled together with the standard clinical blood tests. Blood plasma and serum were frozen at −80 °C. Whole blood was stored at −80 °C for verification of germline mutations that may be found in normal colon tissue of patients and requires follow up and genetic counseling of these patients. Whole blood (2 × 50 µL) was spotted onto Whatman 903 protein saver cards (Whatman products (Cytiva), Little Chalfont, Buckinghamshire, UK), dried for 2 h at room temperature, and frozen at −80 °C for later FA profiling.

### 2.4. Collection of Colorectal Tumor and Healthy Tissue

**Figure 2:**
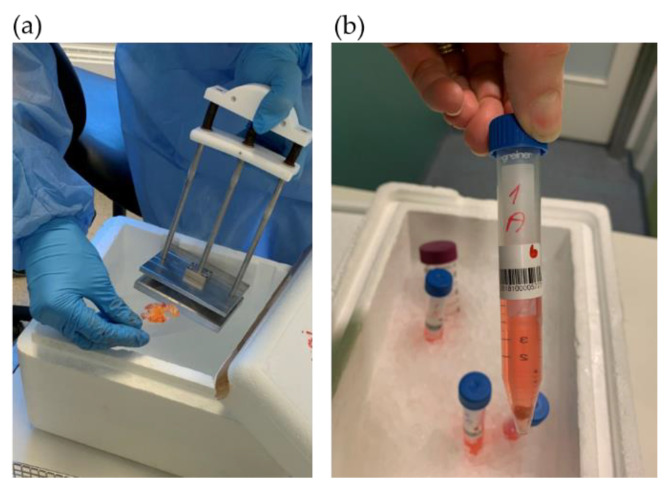
Tumor tissue sampling for: (**a**) biobanking and (**b**) PDX procedure.

Surgical personnel were informed about the request for tissue biopsies through a message in the surgery clinic’s operation plan stating “Biobank Colcan”. Biopsies of tumor and fresh surrounding tissue for biobanking were collected by the Biobank1^®^ personnel. A slide of tissue was frozen in liquid nitrogen using a special clamp (Figure 2a).

**Figure 3:**
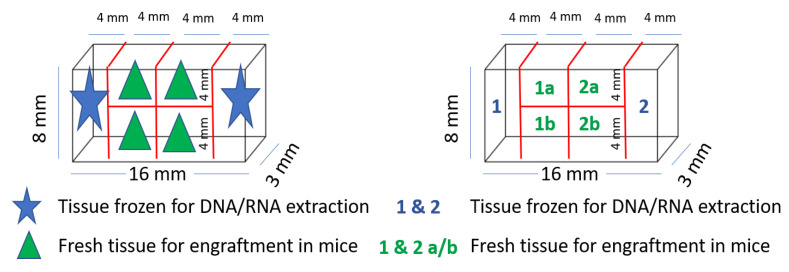
Tumor tissue fragmentation and naming for PDX procedure and exome sequencing.

Tissue samples of both tumor and healthy colorectal tissue were prepared for exome and RNA sequencing, protein isolation and IHC. A fresh tumor tissue sample for PDX was cut into equally sized fragments (Figure 3), placed in sterile tubes (Figure 2b) containing cold Dulbecco′s modified Eagle′s medium (DMEM, #D6429, Sigma Aldrich, Saint-Louis, MO, USA) supplemented with 10% fetal bovine serum (10270-098, Gibco, Thermo Fisher Scientific, Waltham, MA, USA), 1% nonessential amino acids (M7145, Sigma-Aldrich, MO, USA) and 1% gentamicin (15710049, Gibco). Location related to the tumor fragments frozen for DNA extraction was noted (Figure 3). All samples and information such as warm and cold ischemic time were registered deidentified in Biobank1^®^’s program Biobyte (Biobank1^®^, Trondheim, Norway). The CRC tissue fragments for PDX were kept on ice and transported from the clinic to the animal facility, where the mice were prepared for the surgical procedure.

### 2.5. PDX Procedure in Immunodeficient Mice

The mice were acclimatized for minimum 1 week after arrival, and four mice per cage were housed at a switched 12-h light/dark cycle at the Comparative Medicine Core Facility (CoMed), NTNU. Due to the compromised immune system of the CIEA NOG mice, all water, food, and cages were autoclaved before use. At the time of engraftment, the mice were 11 weeks or older.

**Figure 4:**
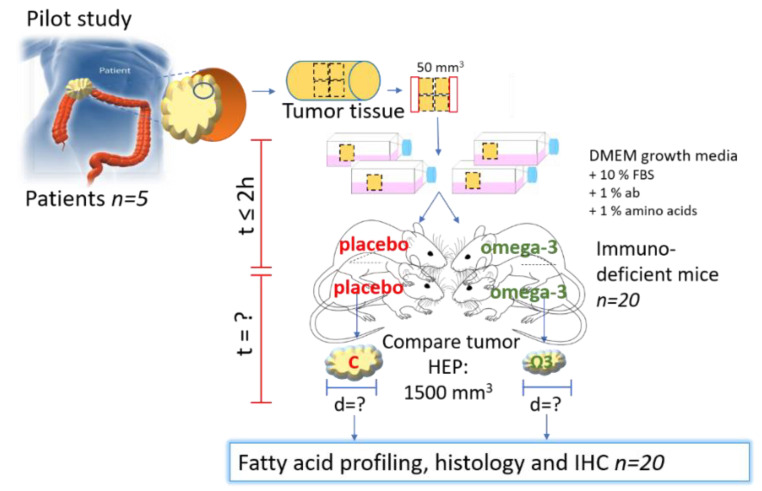
Experimental design of PDX of CRC in mice. C = control/placebo, ω-3 = omega-3, HEP = humane endpoint. Part of the figure is modified from [14] with approval.

CRC tissue fragments were engrafted subcutaneously into female opportunist free CIEA NOG^®^ (NOG) mice (NOD.Cg-PrkdcscidII2rgtm1Sug/JicTac, Taconic Biosciences, Rensselaer, NY, USA). The size of the tissue fragments from the two first patient tumors were about 4 *×* 4 *×* 3 mm (~50 mm^3^), and for the last three tumors about 3 *×* 3 *×* 3 mm (~30 mm^3^). Tissue fragments from each tumor were engrafted in four mice; in total twenty mice (see Figure 4, and Supplementary file 1).

The surgical table and equipment were cleaned with ethanol and the surgical equipment (scissors, forceps, cotton swabs) was autoclaved. The mice were weighed to estimate correct dosing of medications and put into an anesthesia induction chamber provided with 2% isoflurane (ESDG9623C, Baxter, Deerfield, IL, USA, 0.6% N_2_ and 0.4% O_2_) gas using an anesthetic vaporizer, until they were asleep. Eye gel (Viscotears, 597562, Dr. Gerhard Mann chem.-pharm. Fabrik GmbH, Wülfrath, Germany) was applied, and the mice were placed on a heating pad covered with a surgery tissue and kept anaesthetized using a nose cone with 1.5% isoflurane. The anesthetic level was checked by foot pinch using forceps and the mouse was marked by ear clip. The incision area was shaved and washed with Hibiscrub (596023, Mölnlycke, Gøteborg, Sweeden) and chlorohexidine (007269, Fresenius Kabi, Bad Homburg vor der Höhe, Germany). Metacam (025388, Boehringer IngelheimVetmedica GmbH, Germany, 2–3 mg/kg) and Marcaine (169912, Aspen Pharma Traiding Limited, Ireland, 0.04 mg/kg) were given subcutaneously for systemic and local pain relief, respectively. The tumor fragments were placed in a sterile petri dish and washed briefly with sterile physiological NaCl. A small cut (3–4 mm) was made in the skin in front of the back curve, and the tissue fragment was placed in a small pocket under the skin using forceps. The wound was closed by stitching using surgical sutures (Ethicon^®^ VicrylTM 5-0, Ethicon^®^, Somerville, NJ, USA), before the mice were placed on clean paper in a new cage.

A bullet point form was followed during surgery (Supplementary Figure S2-1). Details regarding surgery and anesthesia were registered in a log form (Supplementary Figure S2-2). The animal experiments were approved by the Norwegian Food Safety Authority (Mattilsynet, FOTS ID 12823).

### 2.6. Postoperative Follow-Up

The mice were observed for two hours after surgery. If any stiches were opened by the mice within two hours, the mice were anesthetized and restitched. Metacam was injected subcutaneously 24 h postoperative for systemic pain relief. The mice were housed in single cages for 1–2 weeks after surgery until the wounds had healed, before placing the mice together in groups of four mice per cage. The “rat and mouse No. 1 Maintenance Autoclavable” (RMIA, Special Diets Service, Essex, UK) pellet food and water were autoclaved before use. The pellet food was available to the mice at all time. Water was available until the start of treatment regime; see details below. The mice were weighed 1–3 times per week, and observations were noted in a score sheet for each animal (Supplementary file 1). Subcutaneous tumor size was measured 1–3 times per week as soon as the xenograft was palpable using a digital caliper and registered in the score sheet. Tumor volume was calculated using the following formula V = (length × width^2^)/2. In addition, tumor growth was recorded regularly by imaging.

### 2.7. Mouse Blood Sampling

**Figure 5:**
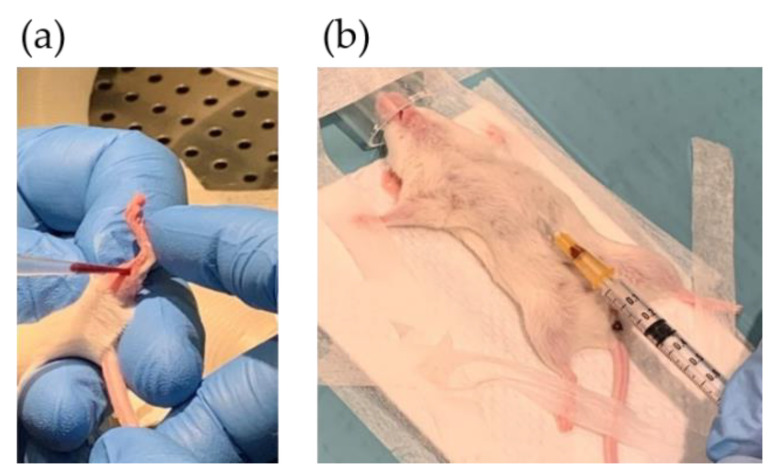
Blood sampling by puncturing: (**a**) vena saphena and (**b**) the heart.

Blood samples were taken from vena saphena before treatment was initiated (Figure 5a). The mice were restrained inside a 50 mL tube with an air opening in the tip and the sampling area was shaved and washed with 70% ethanol. A scalpel was used to punctuate the vein, and 50 µL blood was collected with a pipette and spotted on a Whatman card. The card was dried for two hours at room temperature and stored at −80 °C until FA profiling. An additional <150 µL blood was taken for blood plasma preparation and stored at −80 °C.

At the endpoint, the mice were anesthetized, and blood was collected from the main vein/heart (Figure 5b) before euthanizing the mice by cutting the aorta. Whole blood was spotted on Whatman cards (50 µL) for FA profiling. To prepare plasma, blood was drained from the vein and transferred to Microvette CB 300 capillary tubes with EDTA (Sarstedt, Nümbrecht, Germany). Samples were mixed, centrifuged (3000 rpm, 4 °C, 10 min) and aliquoted. Whatman cards and plasma were stored at −80 °C.

### 2.8. Treatment with Liquid Diets

When growth of the xenograft was confirmed, mice were given either the nutrition drink Smartfish Remune with fish oil (DHA and EPA (2000 mg/200 mL), Smartfish AS, Oslo, Norway), or placebo containing an equal amount of rapeseed oil (Smartfish AS). The diets were isocaloric and isolipidic, differing only by the type of oil. The liquid diets were aliquoted, frozen at −20 °C, thawed, and freshly provided. Mice were given nutrition drinks five days a week for seven hours a day during their active time period; otherwise fresh water was available. The drinking bottles containing the nutrition drink were weighed before and after the seven-hour feeding period to estimate daily intake. A human dose of 4 g omega-3 FAs/day is equivalent to ~1.6 mL Smartfish Remune daily. Animal equivalent dose (AED) based on body surface area was calculated by the equation: AED (mg/kg) = human dose (mg/kg) * (Human Km/Animal Km) = (4000 mg/60 kg) * (37/3) = 822.22 [15]. Oral consumption volume (OCV) was calculated by the equation: OCV (mL) = animal weight [kg] * animal dose (mg/kg)/(concentration mg/mL) = (0.020 kg * 822.22 mg/kg)/(10 mg/mL) = 1.6 mL.

### 2.9. Euthanasia, Necropsy and Tissue Sampling

At the humane endpoint, characterized by either a tumor volume of 1500 mm^3^, reduced health/weight or a xenograft not growing after 6 months, blood sampling, and euthanization were performed as described above and in Supplementary file 3. The tumor area was shaved and washed with ethanol before a cut was made in the skin and the xenograft was collected and picture was taken. The xenograft was divided in parts for histology/IHC, RNA sequencing, and protein analysis. The remaining tumor was collected in tubes and frozen in liquid N_2_ for protein- and RNA analysis. The samples were stored at –80 °C. Tumor tissue (both PDX and any secondary tumors) for histology/IHC was collected in neutral buffered formaldehyde (NBF, 9713.1000, BDH Chemicals, VWR, Radnor, PA, USA, equal to 10% neutral buffered formalin, >10 times tumor volume). The mice bearing xenografts from the last three patient tumors were necropsied. The abdomen was opened, and the lungs, heart, liver, kidneys, stomach, colon, spleen, and thymus were localized, inspected and collected in NBF for histology analysis. Gross findings were registered on an autopsy card (modified from [16]) for each animal (Supplementary file 1).

### 2.10. Fatty Acid Profiling and Omega-3 Index in Blood

The profiling of FAs in human and mouse whole blood was performed using gas chromatography with flame-ionization detection (GC-FID). Samples were analyzed by Vitas AS, Oslo, Norway. Two punches of human whole blood from the Whatman cards were diluted in sodium methylate (900 µL, 0.5 M). FA methyl esters (FAME) were formed by methylation (20 min, 600 rpm, 50 °C) and extracted with distilled water (300 µL) and hexane (500 µL) before thorough mixing (5 min) and centrifugation (5 min, 4000× *g*, 10 °C). The sample (3 µL) was injected into the GC-FID with an HP 7890A Gas Chromatograph System (Agilent Technologies, Palo Alto, CA, USA). FAs were separated on a Supelco 30 m × 250 µm × 0.2 µm column and the results for 11 different FAs were reported as g FAME/100 g FAME. Omega-3 index was calculated by the formula: Omega-3 index = (g DHA FAME + g EPA FAME)/total g FAME * 100.

### 2.11. Histology and Immunohistochemistry Analysis

To compare the histology and molecular characteristics of patient tumor tissue with the corresponding mouse xenografts, tissue samples were taken for histology and immunohistochemistry (IHC) analyses. Tumor tissue and PDX fragments were collected in tubes with 4% NBF. The Cellular and Molecular Imaging Core Facility (CMIC), Department of Clinical and Molecular Medicine (IKOM), NTNU used a histological routine procedure to process, paraffin embed (FFPE), section (4 µm) and dry (60°) the samples. The slides were stained with hematoxylin, erythrosin and saffron (HES) in the automatic slide stainer Sakura Tissue-Tek © PrismaTM. The slides were dried further in the instrument’s heat chamber, before being deparaffinized through several baths of Tissue Clear and rehydrated through descending grades of ethanol to water. The slides were stained in Haematoxylin followed by bluing in water, before being stained with Erythrosine and rinsed in water for removal of excess dye. Slides were further dehydrated through ascending grades of ethanol and stained in saffron before being rinsed in several baths of absolute ethanol and cleared in Tissue Clear before cover slipping in Sakura Tissue-Tek © GlasTM automatic coverslipper. The sections were dried overnight in a well-ventilated place due to chemical evaporation. Dyes used were: Heamatoxylin (CellPath/Chemi-Tecnic, RHD-1475-100, CI No 75290), Erythrosine 239 (VWR, no 720-0179) and saffron (Chemi-Tecnic as, Chroma 5A-394, CI No 75100). Interpretation was performed by experienced pathologists using light microscopy.

For IHC analysis FFPE tissue sections (4 µm) were cut onto SuperFrost Plus slides, dried overnight at 37 °C, and then baked for 2 h at 60 °C. The sections were dewaxed in Tissue Clear and rehydrated through graded alcohols to water in an automatic slide stainer (Sakura Tissue-Tek © Prisma™). Next, the sections were pretreated in Target Retrieval Solution, High or Low pH (Dako, Agilent, Santa Clara, CA, USA, K8004/5) in PT Link (Dako) for 20 min at 97 °C to facilitate antigen retrieval. Further staining was performed on the Dako Autostainer. Following soaking in wash buffer, endogenous peroxidase activity was quenched by incubation in Peroxydase block (Dako S2023). Sections were washed in wash buffer and incubated with primary antibody for 40 min. Further, the slides were washed in wash buffer before incubating for 30 min in labelled polymer HRP anti-Mouse (Dako K4001) and DAB (Dako K3468) to develop the stain. Tris-buffered saline (TBS) (Dako K8007) was used throughout for the washing steps. In Sakura Tissue-Tek © Prisma™, the slides were lightly counterstained with Hematoxylin, dehydrated through ascending grades of ethanol, cleared in Tissue Clear, and coverslipped. For the tissue studied, appropriate negative controls were performed by omitting the primary antibody. Antibodies used were Ki67 (M7240, clone MIB-1, Dako), CD45 (M0701, clones 2B11 + PD7/26, Dako) and CD20 (M0755, clone L26, Dako). Interpretation was performed by experienced pathologists using light microscopy.

### 2.12. In Situ Hybridization

To detect Epstein–Barr-Virus (EBV)-encoded RNA the Inform EBER (EBV early RNA) probe (Ventana Medical Systems, Inc., Oro Valley, AZ, USA) was used for in situ hybridization (ISH) of NBF fixated paraffin embedded patient tumor and lymphoma tissue sections. The EBER ISH staining was routinely performed at the Unit of Immunohistochemistry, Department of Pathology, St. Olav’s University Hospital. Paraffin embedded tissue sections were cut at 3 µm onto Superfrost slides (Thermo Scientific, Waltham, MA, USA, Superfrost plus, #J1800AMNZ). On the slides was also a known positive sample (as control). Each sample was sectioned onto two slides, one for the EBER probe and one for a Negative control probe (REF 800-2847). The section with the negative control probe was used for identification of unspecific staining. The sections were dried at 60 °C for 1 h. The analysis was performed on BenchMark Ultra instrument (Ventana, Roche, Basel, Switzerland) using the Ventana ISH IView Blue Detection Kit (Ventana, Roche, #800-092), ISH protease 3 (Ventana, Roche, #780-4149), RED II counterstain (Ventana, Roche, #780-2218) and INFORM EBER Probe (Ventana, Roche, #800-2842). After the ISH process the slides were dehydrated through ascending grades of ethanol and xylene before cover slipping in Microm Cover- Tech CTM6 (Thermo Scientific) automatic coverslipper. Interpretation was performed by experienced pathologists using light microscopy.

### 2.13. Data Analysis

All data analyses were performed in R. For the FA profiling, a hierarchical mixed linear model accounting for multiple measurements from the same mice were fitted using the lme() function from the nmle package. Overall effects of each FA was assessed using the Anova() function, and pairwise differences using the ghlt() function to perform post hoc Tukey tests. In addition to the internal adjustments of *p*-values for multiple testing within each model as calculated by the ghlt() function, *p*-values were further adjusted based on the number of FAs measured using the Bonferroni correction.

## 3. Results

### 3.1. Patient and Tumor Characteristics

**Figure 6:**
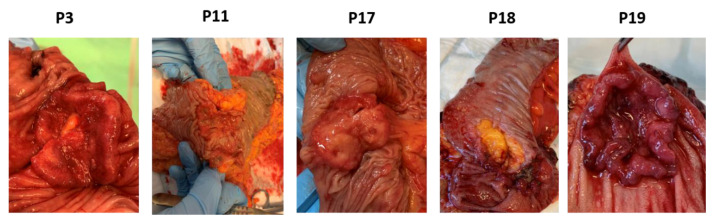
Pathology of CRC tumors from patients (p) 3, 11, 17, 18 and 19.

All invited patients attended the preoperative information meeting and accepted enrollment of the study by signing an informed consent form. Table 1 shows the patient and tumor characteristics along with information regarding the patient’s intake of fish, cod liver oil, omega-3 supplements, and lipid modifying medications recorded from the questionnaires. The included patients consisted of four males and one female (average age 67.6 years). Three tumors were surgically removed from the colon and two from the rectum. One tumor was described as ulcerating, two tumors were exophytic and ulcerating, and two stricturated the colon. All tumors were adenocarcinomas, differed in morphology (Figure 6), and the tumor sizes were between 3.7–5 cm across the largest diameter. Three tumors were staged T3 and two tumors T4. Lymph nodes close to the tumor were affected in two patients; meanwhile, no other metastases were found in any of the patients. All patients reported an intake of omega-3 supplements 0–3 times monthly, two patients had a daily intake of cod liver oil, and all patients had intake of fish 1–3 times weekly.

### 3.2. Establishment of Patient-Derived Xenografts of Colorectal Cancer

**Figure 7:**
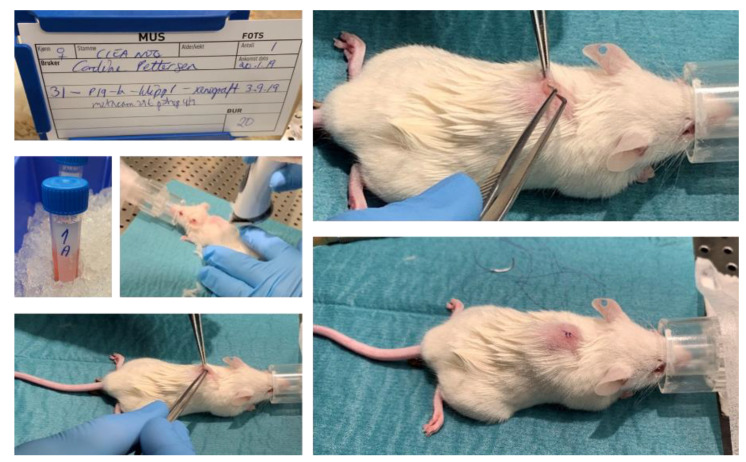
Implantation of a tumor fragment into immune suppressed CIEA NOG mouse #31.

Fresh tissue fragments from five primary CRC tumors were engrafted in four immunodeficient CIEA NOG mice per tumor (total *n* = 20, Figure 4). Animal and engraftment details are given in Table 2. The age of the mice varied from 11 to 33 weeks. Tissue fragments were placed in DMEM on ice within ~20 min and implanted subcutaneously in the mice within 60 min after the tissue was collected (Table 2 and Figure 7).

**Figure 8:**
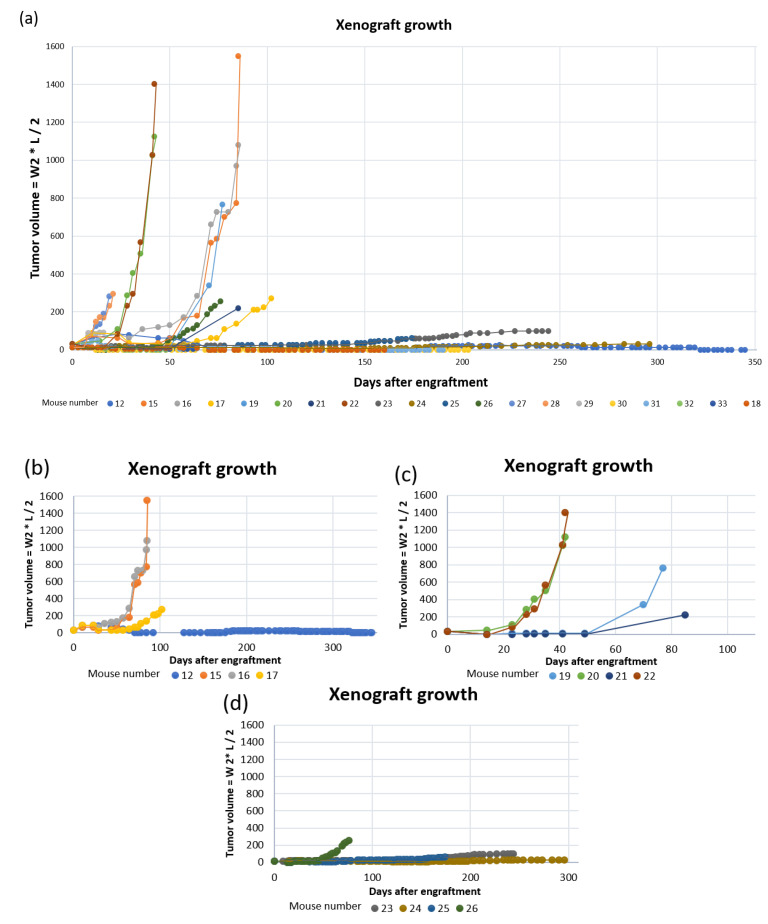
PDX growth curves for: (**a**) all mice; (**b**) mouse #12, 15, 16 and 17 (tumor fragments from patient 3); (**c**) mouse #19, 20, 21 and 22 (tumor fragments from patient 11) and; (**d**) mouse #23, 24, 25 and 26 (tumor fragments from patient 17).

Of the twenty mice engrafted with tumor tissue fragments, ten mice established tumor growth (Figure 8), giving a total engraftment rate of 50%. The average number of days to established xenograft growth was 87 (±51) days (Table 2). The engraftment rate and average number of days until established xenograft growth using 50 mm^3^ fragments were 88% and 65 (±23) days, respectively (Table 2). When engrafting 30 mm^3^ tumor fragments, only the tumor from patient 17 established growth of three xenografts after an average of 137 (±60) days. Only four PDXs exceeded humane endpoint of 1500 mm^3^ before euthanization. Of these, two originated from patient tumor 3 and two from patient tumor 11 (engrafted with 50 mm^3^ fragments).

**Figure 9:**
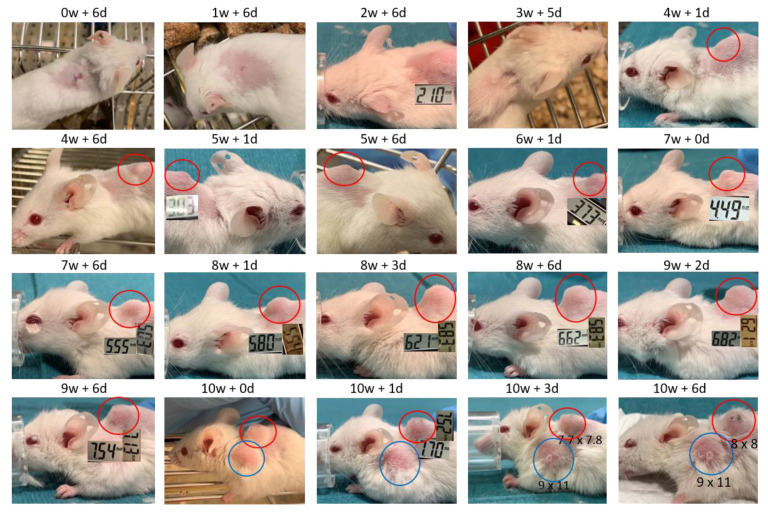
Images of PDX growth (red ring) for mouse #26 (patient 17). Secondary tumor from week 10 (blue ring). Numbers are millimeter (mm) length of the tumor in two dimensions. W = week, d = days.

**Figure 10:**
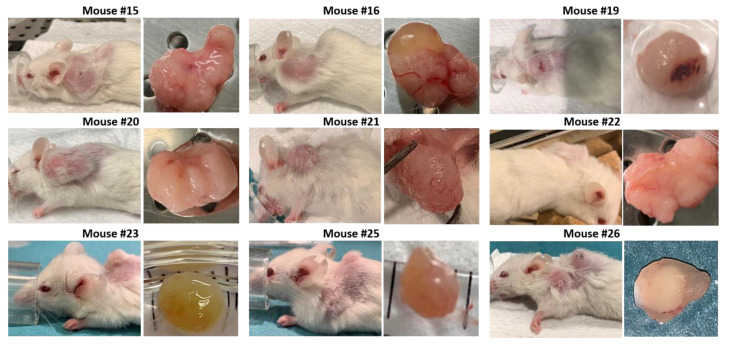
Images of 9 of 10 established PDXs, taken at the day of euthanization. Mouse #17 died during the anesthetic procedure (isoflurane) when assessing tumor size and the tumor was not sampled.

Images and score sheets including animal weight and PDX size are presented in Supplementary file 1. As an example, pictures of PDX growth for mouse #26 are shown in Figure 9, which also illustrates the growth of a secondary tumor. Images of 9 of the 10 established PDXs are presented in Figure 10.

The weight for most mice was stable throughout the study (Supplementary Figure S4-1, Table 3). However, some mice were euthanized due to reduced weight and their general health condition (Table 3). Both mice #19 and #21 had a weight reduction of about 10–15% at the day of euthanization.

### 3.3. Development of Secondary Tumors and Abscesses

**Figure 11:**
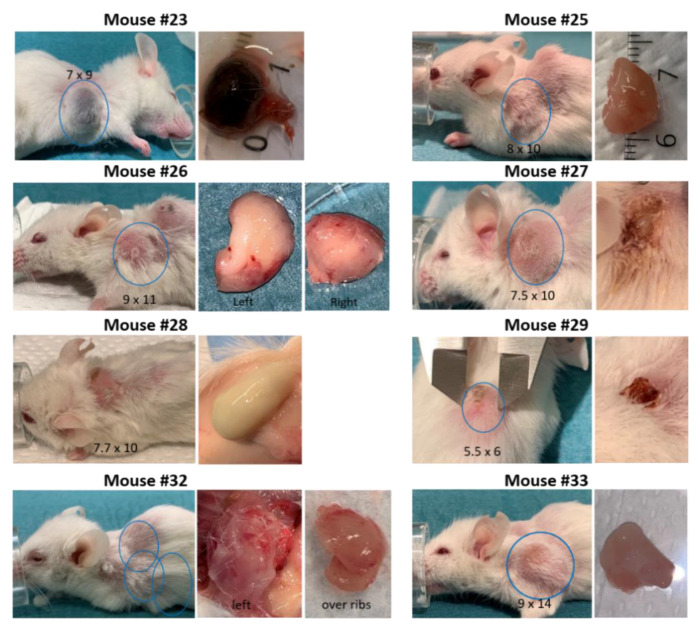
Pictures of mice developing secondary tumors (mouse #23, 25, 26, 32 and 33) or abscesses (mouse #27, 28 and 29). Numbers represent tumor size in mm.

Some mice developed growth of secondary tumors or abscesses. Mouse #23 developed a dark colored secondary tumor behind the right foreleg. At necropsy the tumor was found to contain a dark dense liquid, probably due to blood accumulation (Figure 11).

Four mice (#25, 26, 32 and 33) developed spontaneous rapidly growing secondary tumors located on the front shoulders (Figure 11). Necropsy of mouse #32 indicated red swollen legs and shoulders with bleeding areas in addition to the secondary tumor.

Three of the four mice engrafted with tumor fragments from patient 18 developed abscesses in the surgery area after tissue implantation and were euthanized. However, treatment was initialized before the “tumors” were recognized as abscesses (Figure 11 and Supplementary Figures S1-27-2, S1-28-2 and S1-29-2). For mouse #28 the abscess was intact when removed at euthanization (Figure 11).

### 3.4. Histological Similarity between Patient Tumors and Patient-Derived Xenografts

**Figure 12:**
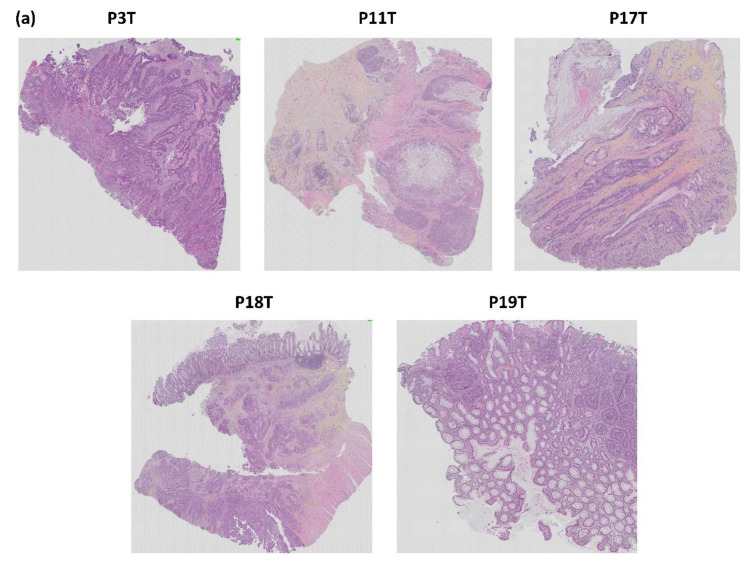

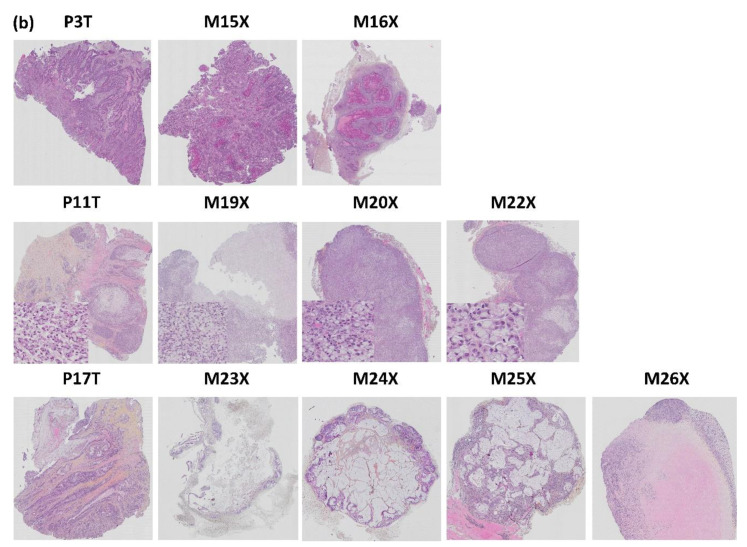
Histopathological comparison of: (**a**) the five patient CRC tumors and (**b**) three patient tumors and matched PDX tissue sections. The tissue sections are stained with HES. *p* = patient, T = tumor, M = mouse, X = xenograft.

The histological architecture of the growing PDXs demonstrated high correlation to the primary tumors as shown by hematoxylin, erythrosine and saffron (HES) staining of tumor tissue slides (Figure 12). All five patient tumors were confirmed by pathologists to be colorectal adenocarcinomas. The histomorphology of the growing PDXs was similar to the histomorphology of the primary tumors, reflecting the heterogeneity of the primary tumors. We did not observe any direct changes in morphology between fish oil and placebo treated PDXs.

The tumor from patient 3 was described as a typical colorectal adenocarcinoma. The glandular forms found in the HES stained section were also observed in the corresponding xenografts in mouse #15 and 16. Mouse #17 had a growing xenograft, but died due to technical problems during anesthesia and the xenograft was not sampled. Mouse #12 did not establish growth of the xenograft.

HES staining of the histology slide from patient 11 showed that the tumor was compact and mainly consisted of signet ring cells. These cells are rare CRC cells with the nucleus placed at one side and a large mucus droplet filling most of the cell. Growth was established for all four PDXs from patient tumor 11. However, mouse #21 was euthanized due to acutely reduced health and the xenograft was not sampled. HES staining confirmed that the corresponding PDXs had a high degree of histopathological similarity to the patient tumor (Figure 12).

**Figure 13:**
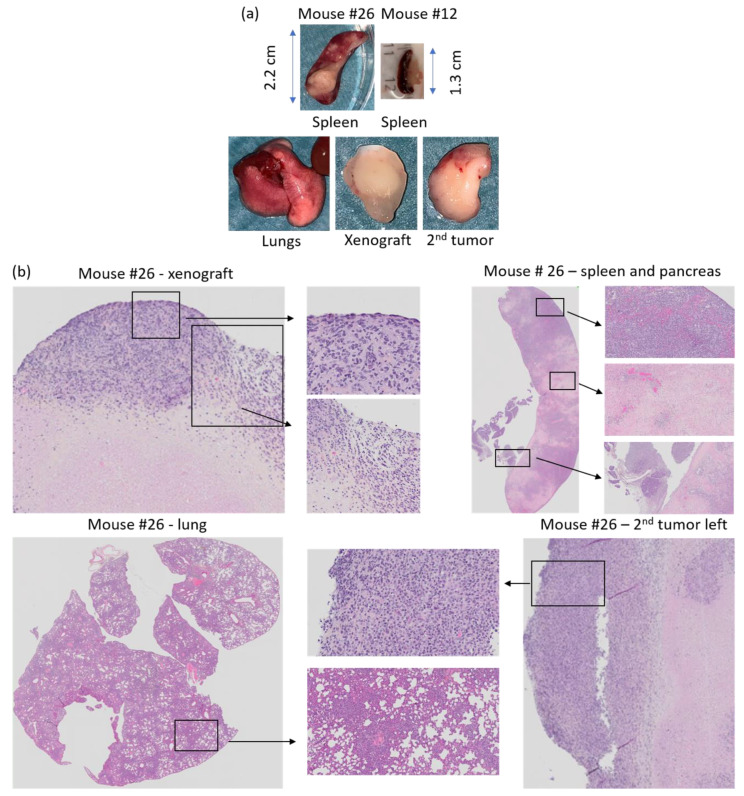
(**a**) Tissue collected at necroscopy from mouse #26 and spleen from mouse #12 and (**b**) HES staining of PDX, spleen, second tumor left shoulder, and lung from mouse #26. Xenograft; necrotic tissue surrounded by lymphoid cancer cells. Spleen/pancreas; lymphoid cancer cells and pale necrotic tissue areas in the spleen. Lung; dense areas with lymphoid cancer cells. Secondary tumor left shoulder; compact tumor with lymphoid cancer cells.

The tumor from patient 17 had classical CRC histology with glandular forming and mucus producing cells (Figure 12). Growth was established for three out of four PDXs; mouse #23, 25 and 26. The corresponding PDXs of mice #23, 24 and 25 were adenocarcinomas with varying degree of mucus production (Figure 12). Mouse #23 was euthanized before reaching maximal tumor size due to reduced weight. Mouse #24 had a growing xenograft, but did not pass as “established PDX” due to a diameter less than 5 mm. PDXs from both mouse #23 and 24 had tumor glandules with necrotic debris in the lumen (typical for CRC tumors), as well as a necrotic core (Figure 12, Supplementary Figures S1-23-9 and S1-24-6). The PDX of mouse #25 contained glandular forming cells surrounded by a dense infiltrate of lymphoid cells (Figure 12). The outer part of the xenograft had cells with irregular nuclear membranes indicating stressed cells. The xenograft from mouse #26 had a large pale necrotic core surrounded by a dense lymphoid filtrate (Figure 12 and Figure 13).

The tumors from patients 18 and 19 were both confirmed to be typical colorectal adenocarcinoma (Figure 12). However, none of the xenografts established growth in the host mice. The tumor from patient 18 grew in small glands and strands through the muscle layer of the bowel wall. However, as mentioned, three out of four mice engrafted with tumor fragments from patient 18 were euthanized due to rapidly developing abscesses before the xenografts were established, hence there are no histology results for these.

### 3.5. Histology of Secondary Tumors and Affected Organs

After euthanasia, the mice engrafted with tumor tissue from the three last patient tumors were necropsied. To study the histology by HES staining, lungs, heart, spleen, liver and any secondary tumors were sampled.

HES stained histology slides of the secondary tumors from mouse #25, 26, 32 and 33 contained malignant looking lymphoid cells (lymphoma). The secondary tumor of mouse #25 was a massive tumor consisting of lymphoid cells, while the spleen, pancreas and lungs appeared healthy (Supplementary Figure S1-25-3). Mouse #26 developed lymphoid tumors on both axes, and tumor areas with lymphoid cells were observed in the spleen, pancreas and lungs. Moreover, the lungs, spleen and pancreas had fields with pale necrotic tissue areas, and the spleen was enlarged (2.2 cm, Figure 13a,b, Supplementary Figure S1-26-4) compared to normal spleen from mouse #12 (1.3 cm, Figure 13a).

Mouse #32 developed lymphoma and was euthanized before growth of the xenograft was established. Lymphoid cancer cells were also found in the lungs and red swollen leg and shoulders of mouse #32. The spleen was enlarged (>2 cm) and contained lymphoid tumor cells (Supplementary Figures S1-32-3 and S1-32-6). Mouse #33 had lymphoid cancer cells present in the secondary tumor, lymph node from the neck and in the enlarged spleen, where we also observed pale necrotic areas (Supplementary Figure S1-33-6).

Mouse #23 developed a secondary tumor behind the right foreleg (Figure 11). At necroscopy the tumor consisted of a bladder containing dark liquid. The liquid was washed away using sterile NaCl and the rest was stored in 4% neutral buffered formaldehyde (NBF). HES staining did not indicate any lymphoid cells. The tumor appeared more like a cyst with liquid filled structures lined with benign looking epithelium (Supplementary Figure S1-23-9).

### 3.6. Origin of Cells Found in Lymphomas and Presence of Epstein–Barr Virus

**Figure 14:**
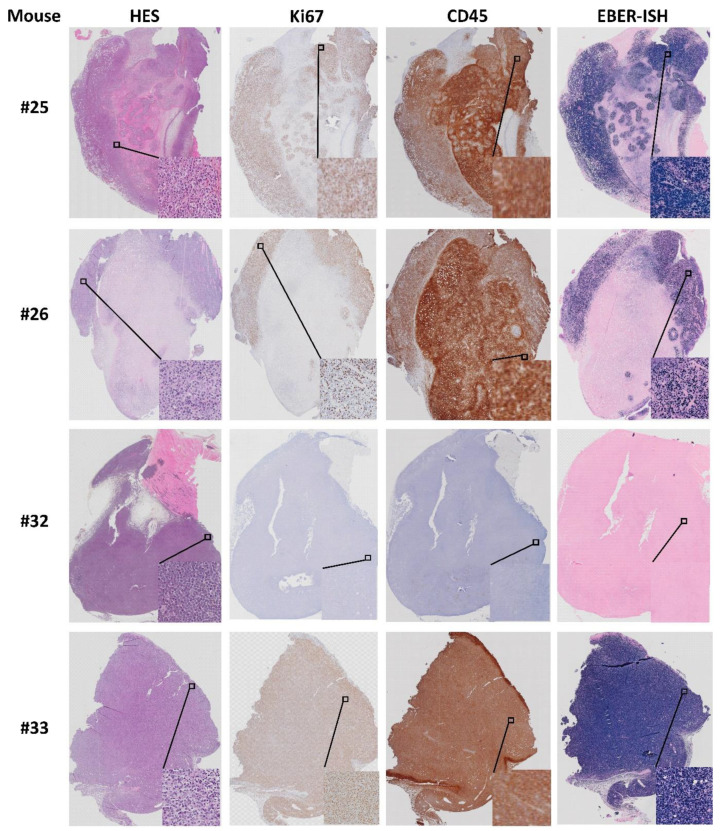
Characterization of lymphomas from mouse #25, 26, 32 and 33. HES, Ki67, CD45 and EBV RNA (EBER; see methods) staining.

All four lymphoma cases (mouse #25, 26, 32 and 33) were composed of actively proliferating neoplastic lymphoid cells including a high number of mitotic cells (Figure 14). To distinguish between human and murine cells and confirm lymphoid cell origin, lymphoma slides were IHC stained with anti Ki67 and leukocyte common antigen (LCA/CD45) specific for human cells (Figure 14). Three of the lymphomas were positive for human specific Ki67 (MIB1, Dako) indicating human cell origin, whereas the fourth was negative for the MIB1 antibody. The three MIB1 positive lymphomas were also CD45 positive (mouse #25, 26 and 33, Figure 14), while mouse #32 was negative for CD45, human specific Ki67 (Figure 11) and CD20 (Supplementary Figure S1-32-7). Hence, the lymphoma of mouse #32 is likely to have a murine origin. Other studies have reported that formation of human lymphomas in PDX models can be a result of outgrowth of Epstein–Barr-Virus (EBV) transformed lymphoid cells from the original tumor [10,17]. To address this, we tested whether the four lymphomas and the two respective patient CRC tumors were positive for EBV. The results demonstrate that three Ki67+ and CD45+ lymphomas were positive for EBV-coded RNA in the nuclei (Figure 14), indicating that EBV was latent in the tumors of patients 17 and 19 from which the lymphomas originated. However, the patient tumors (results not shown) and the lymphoma from mouse #32 (Figure 14) were negative for EBV RNA. The method controls for IHC and ISH were negative (results not shown).

### 3.7. Intake of Liquid Diet

When PDXs reached “established growth”, mice were given liquid diets; Smartfish Remune Peach supplemented with either omega-3 FAs (fish oil) or rapeseed oil (placebo). The mice were observed to drink from the bottles (Supplementary Figure S1-23-4), and the nutrition drink was observed in the stomach of some of the animals at necroscopy. The daily intake (mL) per animal was estimated by weighing the drinking bottles before and after they were provided to the mice (density 1.047 g/mL). However, there was a considerable amount of spillage/leakage from the bottles, hence the estimated intake was inaccurate. Only mouse #23 was given the drink for the scheduled 8 weeks.

### 3.8. Fatty acid Profiling of Patient and Mouse Whole Blood

**Figure 15:**
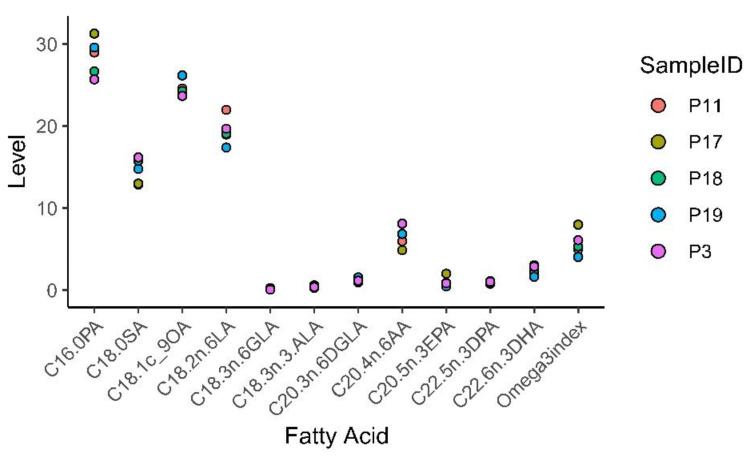
Patient whole blood omega-3 fatty acid (FA) profiling and Omega-3 index.

Whole blood from both patients and mice were spotted on Whatman filter cards to analyze FA content in blood by FA profiling. The results presented in Supplementary Table S5-1 and Figure 15 show that the FA content and Omega-3 index in whole blood varied between patients. The content of the omega-3 FAs eicosapentaenoic acid (EPA), docosapentaenoic acid (DPA) and docosahexaenoic acid (DHA), as well as the Omega-3 index, were highest in patient 17 who reported a daily intake of cod liver oil. Patient 19 had the lowest content of EPA and DHA, and Omega-3 index. This patient reported a daily intake of cod liver oil; however, only during wintertime (blood sample taken in August, Norwegian wintertime September to April).

**Figure 16:**
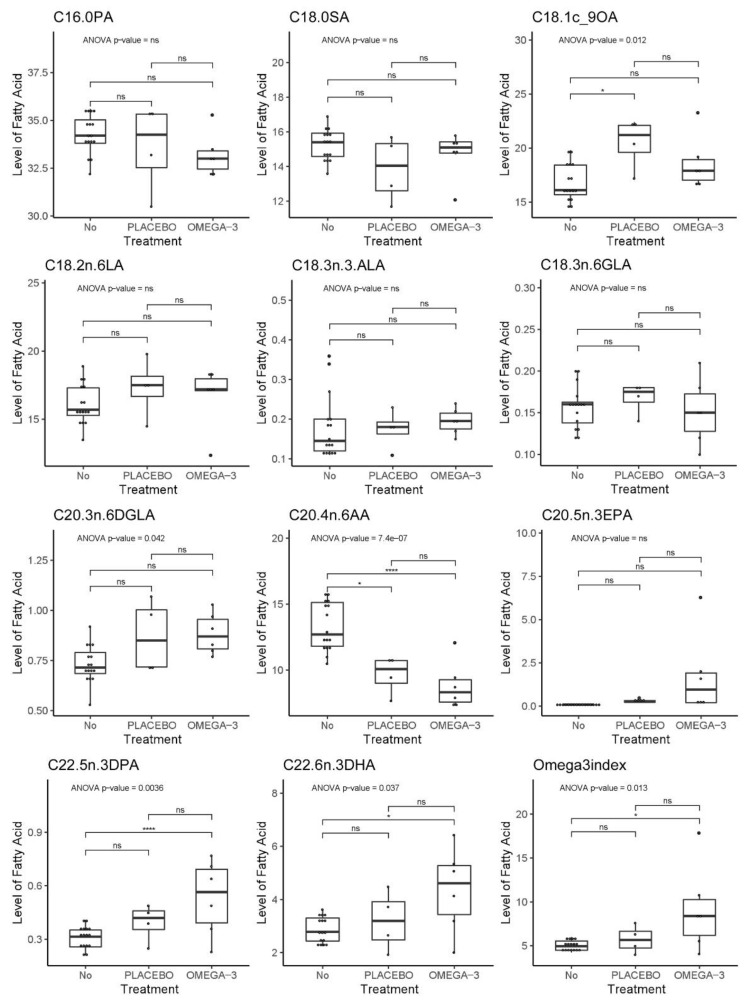
Mouse whole blood FA profiling and Omega-3 index. Stars indicate significant Tukey post hoc test. *p*-values indicate significance in the ANOVA model.

Mouse blood samples were obtained before and after treatment to detect changes in blood FA content during treatment. In mice, the levels of EPA, DHA, and DPA significantly correlated with each other and the Omega-3 index as indicated in Supplementary Figure S5-1. An analysis of variance (ANOVA) on a hierarchical mixed model fitted to each FA and accounting for the measurements before and after treatment in the same mouse model, showed significant effects for oleic acid (OA), digamma linoleic acid (DGLA), arachidonic acid (AA), DPA, DHA, and the Omega-3 index (Figure 16). As expected, the average content of DHA as well as the Omega-3 index were higher in whole blood from the mice receiving fish oil compared to untreated mice (included blood samples from mice before treatment) as shown by a post hoc Tukey test. However, a rise in DHA content was not found in all mice within the fish oil group (mouse #22 and 27). Moreover, we also observed a trend of increased levels of DPA, DHA, and Omega-3 index for mice in the placebo group, although this was not statistically significant. The intake of long chain omega-3 FAs is known to reduce the content of long chain omega-6 FAs since they compete for the same enzymes during FA synthesis (reviewed in [18]). Mice from both treatment groups had reduced AA content in the blood compared to untreated mice; however, the level was lowest in the fish oil group.

## 4. Discussion

The aim of this study was to establish a preclinical PDX model of CRC in immunodeficient mice and give a thorough presentation of the procedure. In addition, we wanted to evaluate administration of omega-3 FAs enriched in a liquid diet in this model.

PDXs preserve the biological characteristics of tumors better than CDX models, and therefore serve as a better research model for personalized cancer treatment. PDXs with established growth may be considered first generation xenografts, while several studies have made PDX lines (third generation PDX) stored in a PDX line biobank for future studies [19]. By using first generation xenografts there is a risk of failure to establish growth, while growth has already been confirmed with second or third generation PDX lines. However, in order to include more patients and use fewer mice, we decided to use first generation xenografts in this study. The study design and group sizes were based on the assumption that data obtained in animal studies typically have a standard deviation of 35%. Power calculations suggested that a sample size of *n* > 10 would allow detection of a 30% change with a significant level of 0.05 and a power of 0.08. However, we experienced that patient tumors had a high degree of heterogeneity and different tumorigenic levels. Not all tumors gave established PDXs, and if growth was established, the latency time was highly variable. Some mice developed abscesses or secondary tumors that reduced animal health and hence the PDX development time. These are important issues that we will address in future PDX studies to estimate required group sizes.

Successful PDX establishment relies on several factors, one of them being the animal host. The CIEA NOG mouse has been shown to be a good host for establishment of PDX models due to its severe immunodeficiency [7]. Tissue acquisition strategy is an important factor, and Katsiampoura et al. found that surgical tissue samples doubled the engraftment rate for PDXs compared to biopsies [11]. Based on this, we chose to use surgically removed tissue samples instead of tissue biopsies in our study. Katsiampoura et al. also found that previous cancer therapy reduced PDX engraftment rate due to the potential growth reduction effect on the tumor and reduction in viable cancer cells [11]. We therefore included treatment naïve patients that did not receive any preoperative treatment. In our study, time from tissue sampling from the tumor to engraftment in mice was up to 1 h. Others have found that implantation of tumor tissue after 12–24 h was equally effective as 2 h, which gives researchers a wider time frame to engraft the tissue samples [11]. For engraftment of tissue from the first two patient tumors, we used tumor fragments sized 50 mm^3^, in line with the study by Katsiampoura et al. [11]. However, for the three last patient tumors, we reduced the size of tumor fragments to 30 mm^3^ to reduce the size of the wound and possibly the distress to the mice. Other studies have used CRC tumor fragments as small as 1–2 mm^3^ [20]. The engraftment site for the PDXs is also important to consider. During CDX studies, cancer cells are usually injected at the flank of the mouse. However, the CIEA NOG mice are very active and during initial tests, the mice opened the stiches and the wound within the first two hours after surgery. We therefore engrafted the tissue in front of the back curve of the mouse so that it would be less accessible. A possible drawback for studying CRC may be that this PDX model uses heterotopic subcutaneous engraftment of the CRC tissue, instead of using an orthotopic model where tissue is implanted into the original source organ in the animal. However, subcutaneous PDX models for CRC are readily used as they are easy to detect, monitor, and characterize (reviewed in [1]).

The first mice (*n* = 8) engrafted with tumor fragments were 11–13 weeks old compared to over 6 months old for the last mice (*n* = 12). The PDXs engrafted in mice at a younger age had a higher engraftment rate compared to the older mice. However, the engraftment rate may also be affected by the size of the tumor fragments, and younger mice were engrafted with larger tumor fragments compared with the older mice. In future PDX studies we will strive to use mice aged 8–12 weeks and use 3.5 × 3.5 mm tumor fragments to increase the PDX engraftment rate. When size of the tumor fragments is reduced, the amount of cancer cells implanted is also reduced, which may affect the growth of the xenografts. Larger fragments will likely represent the heterogeneity of the tumors to a larger extent.

The establishment of PDXs from gastrointestinal tumors has a higher engraftment rate compared to several other cancer types (reviewed in [6]). In this study the total successful engraftment rate was 50%, which is comparable to the engraftment rates reported in the studies by Chijiwa et al. (58% for gastrointestinal tumors) [21] and Katsiampoura et al. (56% for CRC) [11]. However, when using surgically removed CRC tumors for engraftment, Katsiampoura et al. found an engraftment rate of 72% [11], which is comparable to the study by Cho et al. and Wimsatt et al. that reported an engraftment rate of 67% and 64%, respectively [20,22]. When engrafting different types of cancer tissue in CIEA NOG mice, Fujii et al. found that CRC tissue had the highest engraftment rate at approximately 32% [23]. The engraftment rate will be affected by the latency time; that is, the time allowed for growth of the PDX to establish in the animal. In this pilot study, we chose to wait up to six months for growth to establish. However, studies have reported a latency time for CRC PDXs for up to 12 months ([11], reviewed in [24]). Hence, a longer latency time may increase the engraftment rate. In line with our findings, a recent study by Abdirahman et al. also reported an allowed six month latency period until established CRC PDX growth [19], and Chijiwa et al. stated that animals were sacrificed as “failed” if mice did not develop PDX growth over six months from engraftment [21].

The most common CRC tumor type is adenocarcinoma, representing over 90% of all colorectal carcinomas (reviewed in [25]). All five patient tumors in this study were adenocarcinomas, and most PDXs had similar differentiation as the original tumor. However, HES staining of the tumor from patient 11 showed that the tumor consisted mainly of signet ring cells. This is a rare type of CRC which is found in <1% of CRC cases (reviewed in [25]). The fact that the corresponding xenografts showed the same histology and signet ring cell type illustrates the correlation between histology of the original patient tumor and the corresponding PDXs. Signet ring cell carcinomas are often poorly differentiated (high grade) and may give a worse outcome compared to other adenocarcinomas. However, as shown in Table 1, the tumor from patient 11 was microsatellite instability high (MSI-H) and BRAF mutated, which gives an intermediate prognosis (reviewed in [25]). Whether this could affect the engraftment rate and latency time remains to be investigated.

When establishing PDX models, there is a risk of spontaneously developing mouse tumors. In our study, the secondary tumors were first detected close to the location of the xenograft. Hence, inspection and comparison of the histology and molecular markers from the primary tumors and the corresponding xenografts are necessary to be able to distinguish spontaneously growing tumors from xenografts, and to ensure that key characteristics of the original tumors are maintained in the PDXs.

The secondary tumors of mice #25, 26, 32 and 33 were recognized by pathology experts as lymphomas. However, the CIEA NOG mice are reported to have a very low incidence of developing spontaneous lymphomas [9,10]. Yasuda et al. reported spontaneously developing tumors in only 1.31% of the mice, and of these only 0.60% developed thymic lymphoma [10]. In our study, mice developing lymphoma had enlarged spleens with the presence of lymphoid cancer cells. The same was also reported by Yasuda et al. and Fujii et al. [10,17], indicating that the lymphoid cancer cells were distributed systemically. The low incidence of spontaneous lymphomas in CIEA NOG mice is due to the knockout of the *IL2Rg* gene [8]. Fujii et al. found that in 30% of the CRC PDX cases, lymphoma cells replaced the original tumor cells and that the morphology of these tumors was similar to EBV-transformed B cells in SCID mouse [23], which were reported in thirteen of fifty cases in a study by Itoh et al. [26]. Fujii et al. related the findings to the amount of B cells in the original specimen, which is known to be high in colorectal tissue, even though the tumors had nonlymphoid origin [23]. They reasoned that the severe immunodeficiency of the CIEA NOG mouse enhanced the effect of EBV [23]. Some studies have also shown the ability of EBV-transformed human B cells to form a lymphoid tumor as a result of outgrowth from the xenograft [27,28,29,30,31]. Both Choi et al. and Butler et al. found lymphomas with human origin only after the engraftment of the tumor tissue into NOG/NSG mice, but not nude mice [28,29]. They explained these findings by the loss of immune (NK) cells in the NOG/NSG mice compared to nude mice, which makes the NOG/NSG mice more vulnerable to the activation of EBV infected B cells compared to nude mice, which have active NK cells. Butler et al. found that the lymphoma incidence of human B cell origin could be reduced by giving the animals a single dose of the CD20 antibody rituximab at the engraftment time [28]. In our study, we found that three of four lymphomas consisted of human Ki67+ and CD45+ EBV transformed lymphoid cells. Hence the EBV was likely latent in lymphoid cells in the tumor, but at a very low level since it was not detected by EBER ISH in the patient samples. EBV is known as an oncovirus and is found latent in more than 90% of humans (reviewed in [32]). These rapidly growing lymphomas resulted in reduced time for the xenografts to establish due to increased tumor burden and/or reduced general health of the affected animals, as found in the study by Chjiwa et al. [21]. The lymphoma in mouse #32 was somewhat different from the three other lymphoma cases; the same lymphoid cancer cell type was found in the lungs, spleen, both shoulders and one hind leg, as well as in what was believed to be the remainder of the xenograft (Supplementary Figure S1-32-7). The lymphoma of mouse #32 was negative for human specific Ki67, CD20, CD45 and EBV, giving an indication that this lymphoma may be of murine origin. Despite the low rate of formation of spontaneous lymphomas in CIEA NOG mice, Yasuda et al. found thymic lymphoma to be the most common spontaneous tumors in NOG mice with a total incidence of 0.6% [10]. The NOD scid gamma (NSG) mice are also expected to have low incidence of spontaneous lymphomas. However, Moyer et al. found murine lymphomas in a PDX model in NSG mice and separated them from human-derived lymphomas using the same Ki67 MIB1 antibody as used in our study [33].

Three mice developed rapidly growing abscesses within one week after surgery. This may indicate that the tumor fragments were contaminated during the procedure or that the tumor tissue contained intracellular bacteria. For future studies we will provide the mice with antibiotics in the drinking water for 1 week after engraftment to reduce the risk of infection and the formation of abscesses.

Omega-3 PUFAs from fish oil have previously been shown to have a growth inhibitory effect on CRC cells both in vitro ([34,35], reviewed in [36]) and in vivo [37,38]. In addition, some studies have found omega-3 PUFAs to act as adjuvants to anticancer therapies [18]. Most studies that are testing treatment strategies involving omega-3 FA supplemented diets in animal studies have used omega-3 FA enriched pellet diets ([37,38], reviewed in [18]). However, Busquets et al. used oral administration of Smartfish Remune drink with omega-3 FAs as juice blocks to mice for 18 days in a CDX model, where significantly reduced primary tumor growth was observed [39]. We administered Smartfish Remune with fish oil or placebo to mice with established PDXs for 8 weeks. However, only mouse #23 completed the 8-week treatment period (placebo). The other mice were euthanized earlier (Table 3). We estimated that each mouse should drink 1.6 mL nutrition drink to achieve an adequate daily intake of DHA and EPA. Spillage was observed in cages of all mice receiving nutrition drink, meaning that the daily estimated intake was probably higher than the actual intake for all mice receiving treatment. Meanwhile, observations of mice drinking directly from the bottles, detection of nutrition drink in the stomach and results from the whole blood FA profiling, confirmed intake of the nutrition drink.

We performed FA quantification/profiling to investigate whether the patients’ reported intake of fish and omega-3 supplements correlated with their FA profile. Patient 17 stated a daily intake of cod liver oil and had the highest whole blood levels of EPA, DPA and DHA, as well as the Omega-3 index. Patient 17 also had the lowest whole blood level of AA, an omega-6 PUFA known to be partially reduced in membrane phospholipids when omega-3 PUFA intake increases (reviewed in [18,40]). Patient 19 also stated to have a daily intake of cod liver oil but had the lowest Omega-3 index. However, this patient had a daily intake of cod liver oil at wintertime (Table 1), and the blood sample was taken in August. This probably influenced the EPA and DHA levels (which are included in the Omega-3 index) due to an assumed washout period for omega-3 PUFAs of about 12 weeks [41]. Although in vitro cell lines and in vivo animal studies show promising effects of omega-3 PUFAs on cancer growth, there have been few clinical trials exploring and providing evidence of a potential clinical anticancer effect of these PUFAs. However, there are studies reporting significant advantages of combining conventional cancer treatment with omega-3 PUFAs for some cancer types, and that a higher intake of marine omega-3 PUFAs after CRC diagnosis was associated with lower cancer-associated death and longer disease-free survival (reviewed in [18]).

FA profiling showed that the average content of the omega-3 PUFAs EPA, DPA, and DHA increased in both mice given fish oil and placebo compared to untreated animals, but the levels were highest and only significant in the mice provided with fish oil. Changes in the Omega-3 index and the DPA, as well as OA, DGLA, and AA content were statistically significant in the ANOVA analysis. Rapeseed oil is known to be rich in OA (over 50%) [42], and as expected, whole blood from mice given the placebo drink had the highest OA content. However, rapeseed oil does not contain EPA, DPA, or DHA, but it does contain around 8% ALA [42], which is the precursor for synthesis of EPA, DPA, and DHA in mammals. Several experimental animal studies using omega-3 enriched fish oil diets have used corn oil as control oil [18]; however, rapeseed oil was chosen due to lower concentration of omega-6 PUFAs. The reduction in the whole blood content of AA in both fish oil- and placebo treated mice may be considered positive, as AA is a precursor for omega-6 FA derived pro-inflammatory eicosanoids, while eicosanoids from the omega-3 FA EPA are considered anti-inflammatory (reviewed in [43]).

Regarding estimation of FA levels, whole blood reflects the content of both plasma and blood cells and is a more easily obtainable approach compared to using blood plasma [44]. Whole blood is readily sampled as dried blood spots (DBS) which is considered an adequate approach to analyze the content of FAs and long chain omega-3 PUFAs if FA oxidation is prevented [45,46]. Since the average levels of marine omega-3 PUFAs were highest in the mice receiving nutrition drink with fish oil, we consider the DBS analysis method for FA profiling as suitable for our study. This method also applies for analyses of cytokines and vitamin D levels.

## 5. Conclusions

In this study we established a method for the engraftment of CRC PDXs in CIEA NOG mice with an engraftment rate of 50%. The highest engraftment rate was obtained when engrafting larger tumor fragments in young mice. Max latency time was set to six months; however, this time frame should be extended in future PDX setups in order to increase the engraftment rate. The optimal engraftment site was in front of the back curve of the mice to prevent the mice from opening the wounds. Histological staining confirmed that the established PDXs originated from human CRC adenocarcinoma. Some of the older mice developed abscesses or secondary tumors which originated from human Ki67, CD45, and EBV positive lymphoid cells. These are important findings that researchers should be aware of when planning and performing PDX studies. We have presented a strategy to successfully provide mice with fish oil and placebo by liquid diets. The intake of omega-3 FAs was confirmed by the increased omega-3 ratio in blood. The PDX model described represents a valuable research tool for the assessment of different anticancer treatment strategies. Furthermore, the establishment of a biobank with tissue and blood samples from CRC patients will provide a unique platform for future translational research.

## Figures and Tables

**Table 1:** Patient and tumor characteristics (*n* = 5), and questionnaire answers.

	Patient 3	Patient 11	Patient 17	Patient 18	Patient 19
Gender (Male/Female)	M	F	M	M	M
Age	69	65	77	62	65
Tumor site	Colon	Colon	Colon	Rectum	Rectum
Tumor anatomy	Exophytic	Stenosing, ulcerating	Stricturating, ulcerating	Ulcerating	Exophytic, ulcerating
Tumor type	Adenocarcinoma	Signet cell carcinoma	Adenocarcinoma with mucus	Adenocarcinoma	Adenocarcinoma
Tumor stage (TMN)	T3N0M0	T3N2M0	T4N0M0	T4aN1M0	T3N0M0
Tumor differentiation grade	Medium		Medium	Medium	Medium
Tumor size (cm)	4	5	4	4	3.7
Tumor MSI status		MSI-high	MSS	MSS	MSS
Mutations		BRAF	BRAF	KRAS	
Pre-operative cancer treatment	No	No	No	No	No
Previous cancer diagnoses			Yes		No
Intake of omega-3 supplements	0–3/month	0–3/month	0–3/month	0–3/month	0–3/month
Intake of cod liver oil	0–3/month	0–3/month	Daily	0–3/month	Daily ^1^
Intake of fish	1–3/week	1–3/week	1–3/week	1–3/week	1–3/week
Use of lipid reducing medication	No	No	No	Yes	No

^1^ Daily intake of cod liver oil only in the wintertime (September to April). The blood sample was taken in August.

**Table 2:** Animal and engraftment details.

Patient ID	Patient 3				Patient 11				Patient 17				Patient 18				Patient 19			
Mouse ID	12	15	16	17	19	20	21	22	23	24	25	26	27	28	29	30	31	32	33	18
Tumor fragment ID	1a	1b	2a	2b	1a	1b	2a	2b	1a	1b	2a	2b	1a	1b	2a	2b	1a	1b	2a	2b
Ear clip	1	2	3	4	1	2	3	4	1	2	3	4	1	2	3	4	1	2	3	4
Age at engraftment (week)	11	11	11	11	17	17	17	17	31	31	31	31	31	31	31	31	33	33	33	33
Tumor fragment size (mm^3^)	50	50	50	50	50	50	50	50	30	30	30	30	30	30	30	30	30	30	30	30
Days to established growth	-	64	64	99	70	34	91	34	185	-	174	52	-	-	-	-	-	-	-	-

**Table 3:** Characteristics of all mice at the time of euthanization.

Mouse #	Clinical Symptoms & Comments	Necropsy Findings	Body Weight	PDX Size (mm)	Second Tumor Size (mm)
12	PDX not growing after 6 months.	Only a spot at engraftment side. Dark cystic structure close to pancreas.	Stable	-	-
15	Placebo treatment until PDX max size.	Large solid tumor.	Stable	10 × 15.5	-
16	Smartfish Remune treatment until PDX max size.	Large tumor, partly with liquid. Visible blood veins to tumor.	Stable	11.5 × 13	-
17	Smartfish Remune treatment until it died during anesthesia.	No samples taken.	Stable	7.5 × 7.5	-
19	Ulcerating xenograft. Smartfish Remune treatment.	Large solid tumor with blood traces.	Slightly reduced	11.3 × 12	-
20	Placebo treatment until PDX reached max size.	Large solid tumor.	−10–20%	11.5 × 17	-
21	Reduced general health and reduced weight. Large PDX.	Solid tumor. Low blood volume. No samples taken.	−10–15%	7 × 9	-
22	Smartfish Remune treatment until PDX max size.	Large solid tumor w/visible blood veins.	Stable	11 × 17	-
23	Reduced weight. Placebo treatment (8 weeks). Possibly rectal prolapse.	Small spleen. Second tumor with dark liquid inside. Clog of fur and food in stomach.	−10–20%	5.8 × 5.9	7 × 9
24	Did not reach “established growth” 3 months after animals without growing xenografts were euthanized.	Small slowly growing PDX. Whitish lungs. Normal organs.	Stable	3.9 × 4	-
25	Reduced general health. Large second tumor	Established growth of PDX. Whitish lungs. Large second tumor left shoulder.	Stable	5 × 5	8 × 10
26	Ulcerating xenograft. Smartfish Remune treatment. Large second tumor.	Enlarged spleen w/white fields. Whitish lungs. Two second tumors; left and right shoulder.	−10%	8 × 8	9 × 11 (left)
27	Abscess mistaken for PDX until it burst. Smartfish Remune treatment.	Wound at the abscess site. Small xenograft under the skin.	Stable	-	-
28	Abscess mistaken as PDX in the beginning. Placebo treatment	Intact abscess 9.5 × 10 mm containing green liquid. Only a spot at engraftment site	Stable	-	-
29	Abscess mistaken as PDX until it burst. Smartfish Remune treatment	Wound where abscess has burst. Small xenograft under the skin	Stable	-	-
30	Did not reach “established growth” after 6 months.	Normal organs. A small bump in the liver. Trace of PDX under the skin	Stable	-	-
31	Did not reach “established growth” after 6 months.	Small xenograft under the skin. Normal organs.	Stable	-	-
32	Reduced general health. Second tumor. Did not reach “established growth”. Liquid in the eye.	Thick wounded skin at the neck. Whitish lungs. Enlarged spleen >2 cm. Red/swollen legs, shoulders and spine second tumor over the ribs.	Stable	-	-
33	Did not reach “established growth”. Large second tumor.	Large second tumor. No visible xenograft. Enlarged spleen.	Stable	-	9 × 14
18	Did not reach “established growth”. Rectal prolapse.	Swollen, bloody anal opening. Whitish part of one lung. Traces of xenograft under skin. Enlarged spleen ca 2.5 cm	−10%	-	-

## Supplementary Materials

The following are available online at www.mdpi.com/xxx/s1-s5: Supplementary file 1: Animal details; Supplementary file 2: Bullet point form for surgery and anesthesia log; Supplementary file 3: Bullet point form for euthanization, blood and tissue sampling; Supplementary file 4: Mice weight curves; Supplementary file 5: Fatty acid profiling.
