# Supplementary material for: Establishment of a Patient-Derived Xenograft Model of Colorectal Cancer in CIEA NOG Mice and Exploring Smartfish Liquid Diet as a Source of Omega-3 Fatty Acids
Source: Biomedicines. 2021 Mar 10;9(3):282. doi: 10.3390/biomedicines9030282 (PMC8000445; doi:10.3390/biomedicines9030282)
Supplement: Supplementary file 1 [file biomedicines-09-00282-s001.pdf]

Supplementary Materials

Content: Page

- Supplementary file 1: 2-158
- Supplementary file 2: 159-160
- Supplementary file 3: 161
- Supplementary file 4: 162
- Supplementary file 5: 163-164

Supplementary file 1

-Figures are named by:
supplementary file 1 - mouse # - figure #

Content: Page:

- Mouse 12: 3-10
- Mouse 15: 11-17
- Mouse 16: 18-24
- Mouse 17: 25-28
- Mouse 18: 29-33
- Mouse 19: 34-40
- Mouse 20: 41-47
- Mouse 21: 48-51
- Mouse 22: 52-58
- Mouse 23: 59-70
- Mouse 24: 71-80
- Mouse 25: 81-90
- Mouse 26: 91-105
- Mouse 27: 106-111
- Mouse 28: 112-117
- Mouse 29: 118-123
- Mouse 30: 124-132
- Mouse 31: 133-141
- Mouse 32: 142-150
- Mouse 33: 151-158

Mouse 12

- Surgery date: 01.04.19
  - Age (weeks) 11
  - Weight (g) 20.5
  - Tumor fragment P3-1A
  - Ear marks 1
  - Fragment size 4*4 mm

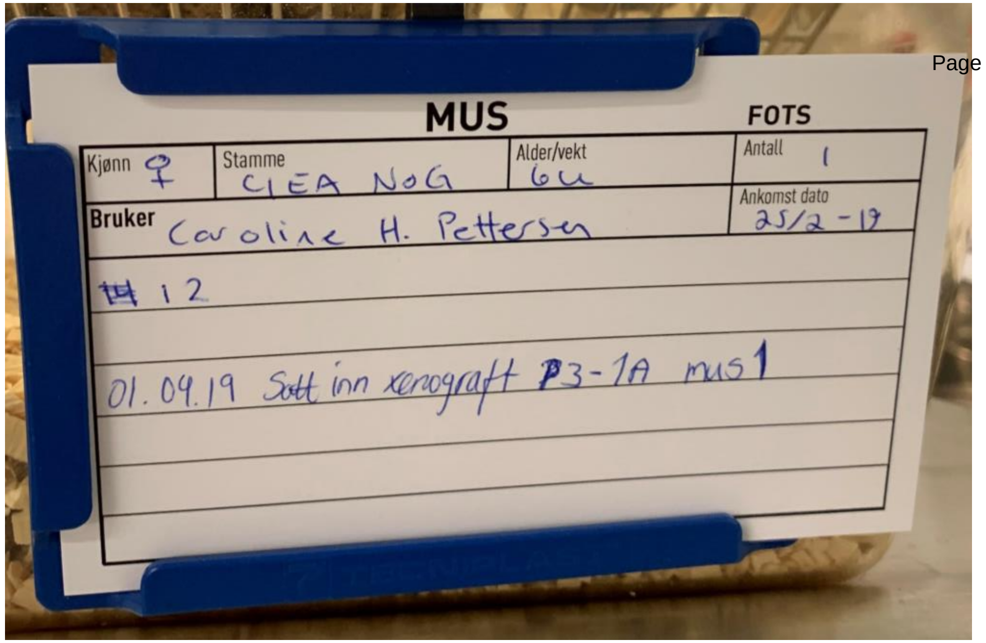

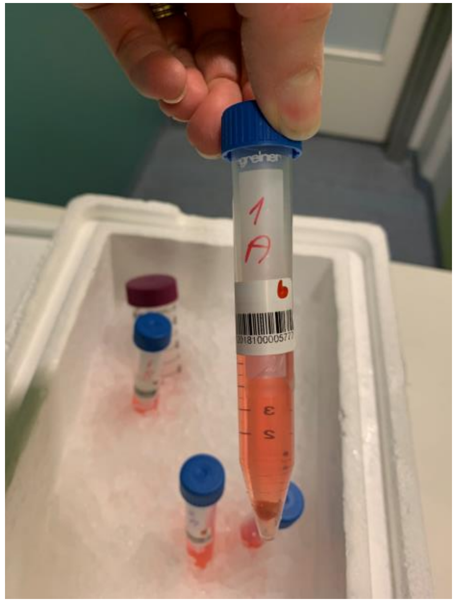

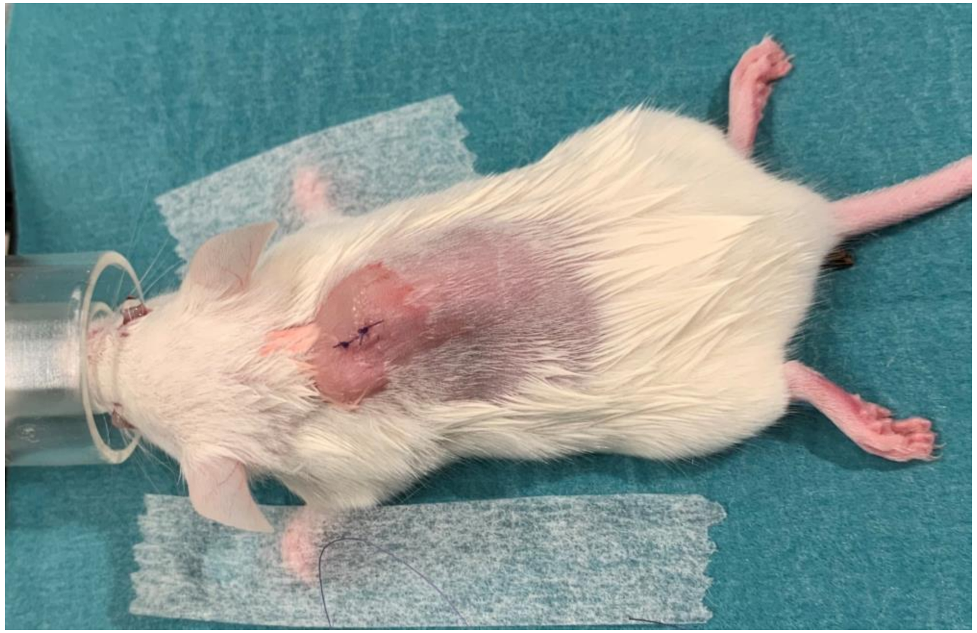

Supplementary figure 1-12-1.
Pictures from the engraftment day.

Supplementary figure 1-2. Pictures from follow up and tumor size measurement

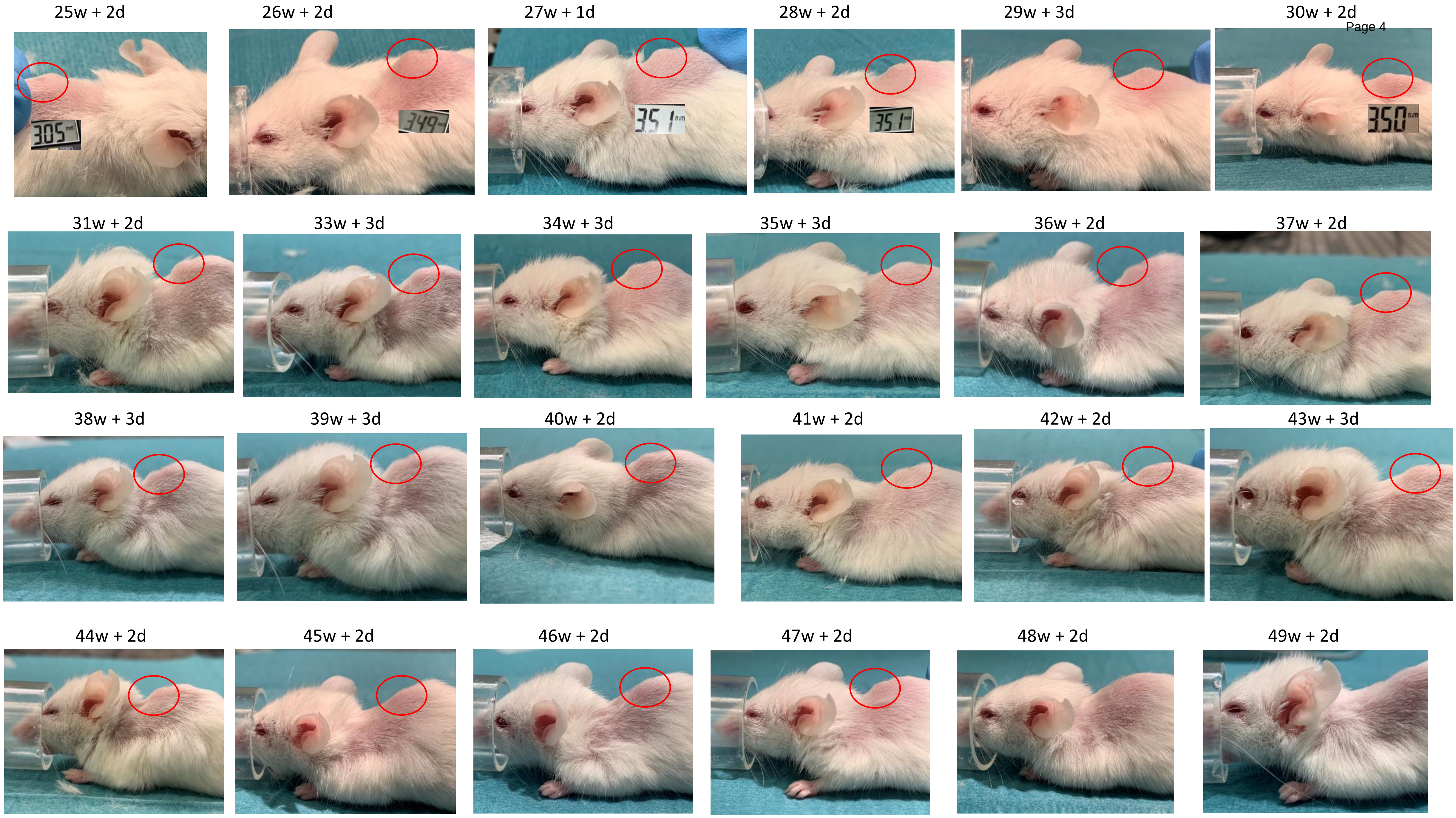

Treatment:

- Mouse 12 was used as a «social mouse» during treatment with Smartfish containing fish oil for mouse 17
  - Did not have growing xenograft at that time, were suspected to have removed xenograft itself

Supplementary figure 1-12-3. Pictures form euthanization of mouse 12 (11.03.2020 / 49w + 2d)

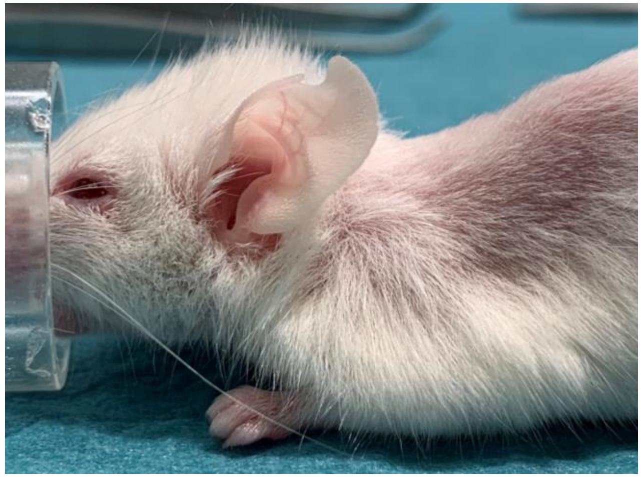

Xenograft almost not visiable

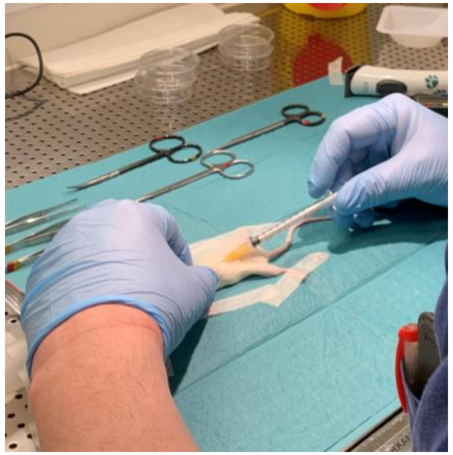

Necroscopy

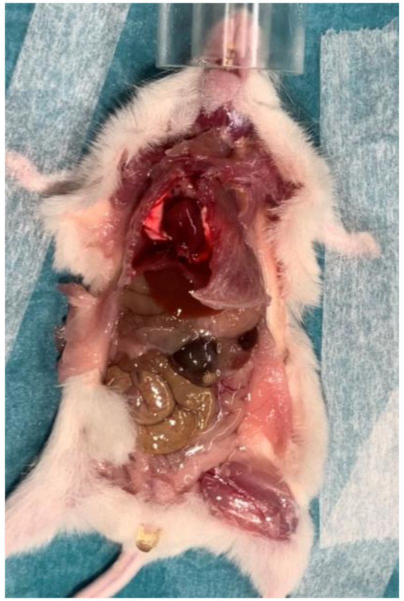

Necroscopy

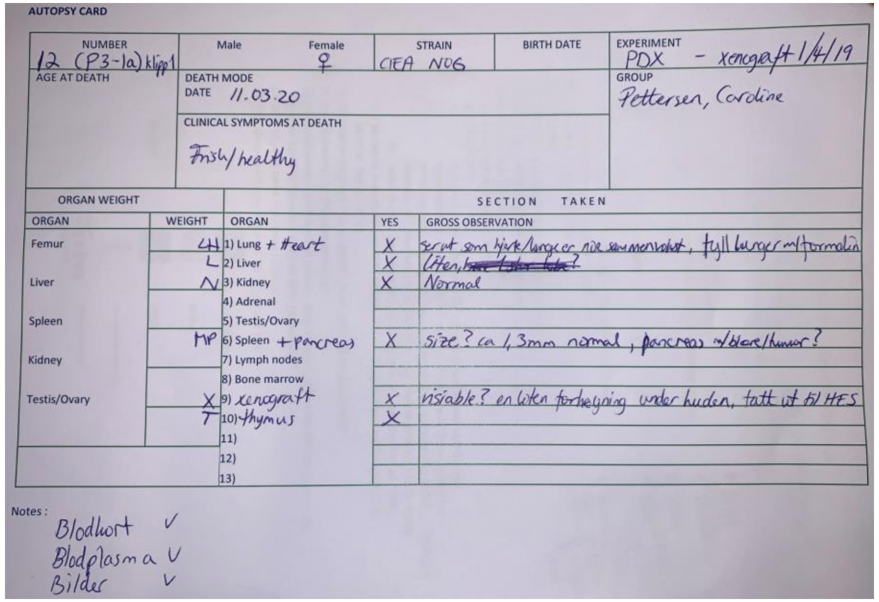

AUTOPSY CARD

NUMBER	Male	Female	STRAIN	BIRTH DATE	EXPERIMENT
12 (P3-1a) klipp1		♀	CIEA NOG		PDX - xenograft 1/4/19

AGE AT DEATH:

DEATH MODE

DATE 11.03.20

GROUP: Pettersen, Caroline

CLINICAL SYMPTOMS AT DEATH: Frisk/healthy

ORGAN WEIGHT: ORGAN	WEIGHT	SECTION TAKEN: ORGAN	YES	GROSS OBSERVATION
Femur	LH	1) Lung + Heart	X	ser ut som hjerte/lunger er noe sammenvokst, fyll lunger m/formalin
	L	2) Liver	X	Liten, [illegible]?
Liver	N	3) Kidney	X	Normal
		4) Adrenal		
Spleen		5) Testis/Ovary		
	MP	6) Spleen + pancreas	X	size? ca 1,3mm normal, pancreas m/blære/tumor?
Kidney		7) Lymph nodes		
		8) Bone marrow		
Testis/Ovary	X	9) xenograft	X	visiable? en liten forhøyning under huden, tatt ut til HES
	T	10) thymus	X	
		11)		
		12)		
		13)		

Notes :
Blodkort ✓
Blodplasma ✓
Bilder ✓

Autopsy card modified from:
http://www.pathbase.net/Necropsy_of_the_Mouse/

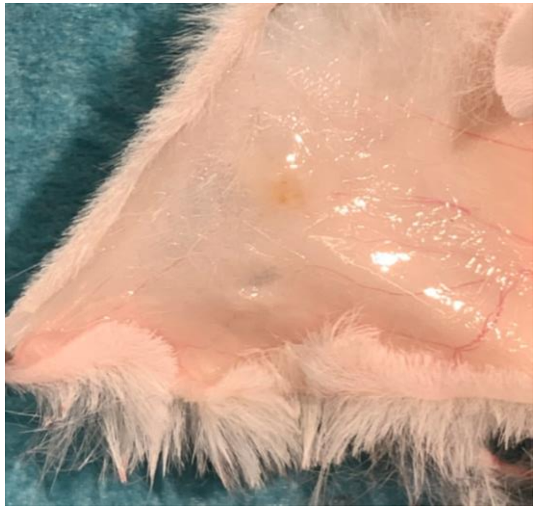

Only small trace visiable of xenograft

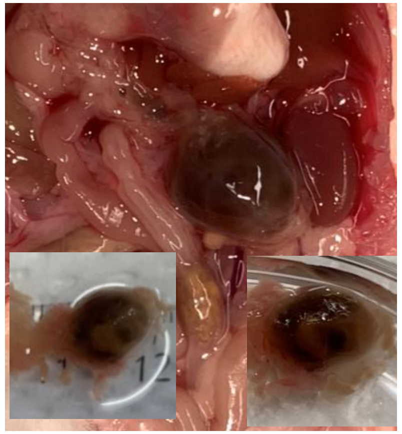

Undefined dark «bladder» with liquid close to pancreas.

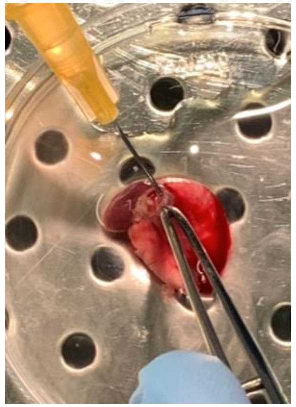

Lungs filled with formalin

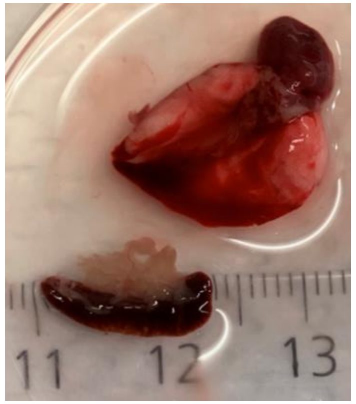

Spleen, lungs and heart

Supplementary figure 1-12-4. Pictures of scoresheets

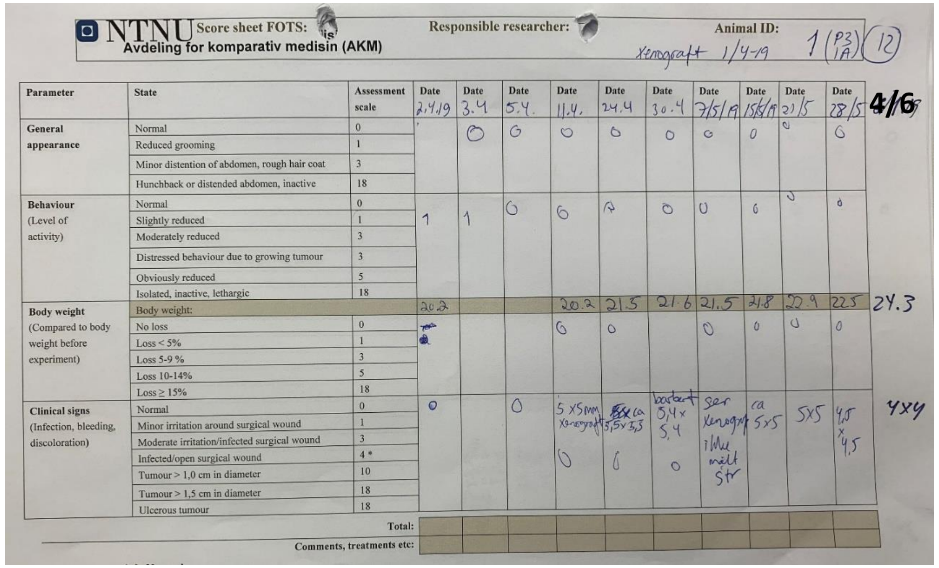

NTNU Score sheet FOTS: Responsible researcher: Animal ID: 1 (P3 1A) (12)
Avdeling for komparativ medisin (AKM)
Xenograft 1/4-19

Parameter	State	Assessment scale	2.4.19	3.4	5.4.	11.4.	24.4	30.4	7/5/19	15/5/19	21/5	28/5	4/6
General appearance	Normal	0		0	0	0	0	0	0	0	0	0	
Behaviour (Level of activity)	Normal / Slightly reduced	0 / 1	1	1	0	0	0	0	0	0	0	0	
Body weight	Body weight:		20.2			20.2	21.5	21.6	21.5	21.8	22.9	22.5	24.3
Body weight (Compared to body weight before experiment)	No loss	0	[illegible]			0	0		0	0	0	0	
Clinical signs (Infection, bleeding, discoloration)			0		0	5x5mm xenograft 0	[illegible] 3,5x3,3 0	[illegible] 5,4x5,4 0	ser xenograft ikke målt str	ca 5x5	5x5	4,5 x 4,5	4x4

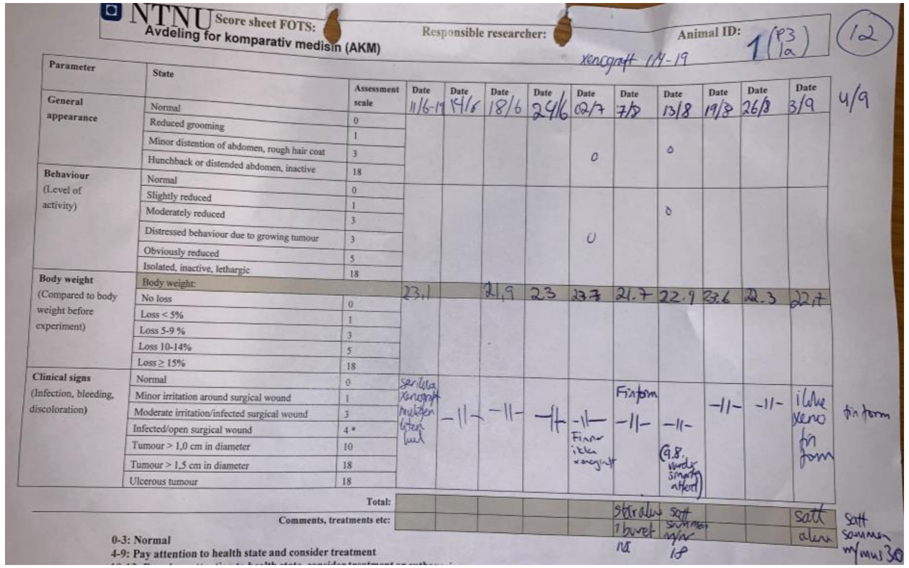

NTNU Score sheet FOTS: Responsible researcher: Animal ID: 1 (P3 1a) (12)
Avdeling for komparativ medisin (AKM)
Xenograft 1/4-19

Parameter	State	Assessment scale	11/6-19	14/6	18/6	24/6	02/7	7/8	13/8	19/8	26/8	3/9	4/9
General appearance	Minor distention of abdomen, rough hair coat	3					0		0				
Behaviour (Level of activity)							0		0				
Body weight	Body weight:		23.1		21.9	23	23.7	21.7	22.9	23.6	22.3	22.7	
Clinical signs (Infection, bleeding, discoloration)			ser ikke xenograft [illegible]	-ll-	-ll-	-ll-	-ll- Finner ikke xenograft	Fin form -ll-	-ll- (9,8 [illegible])	-ll-	-ll-	ikke xeno fin form	fin form
Comments, treatments etc:							[illegible]	[illegible]	[illegible]			satt alene	satt sammen [illegible] 30

0-3: Normal
4-9: Pay attention to health state and consider treatment

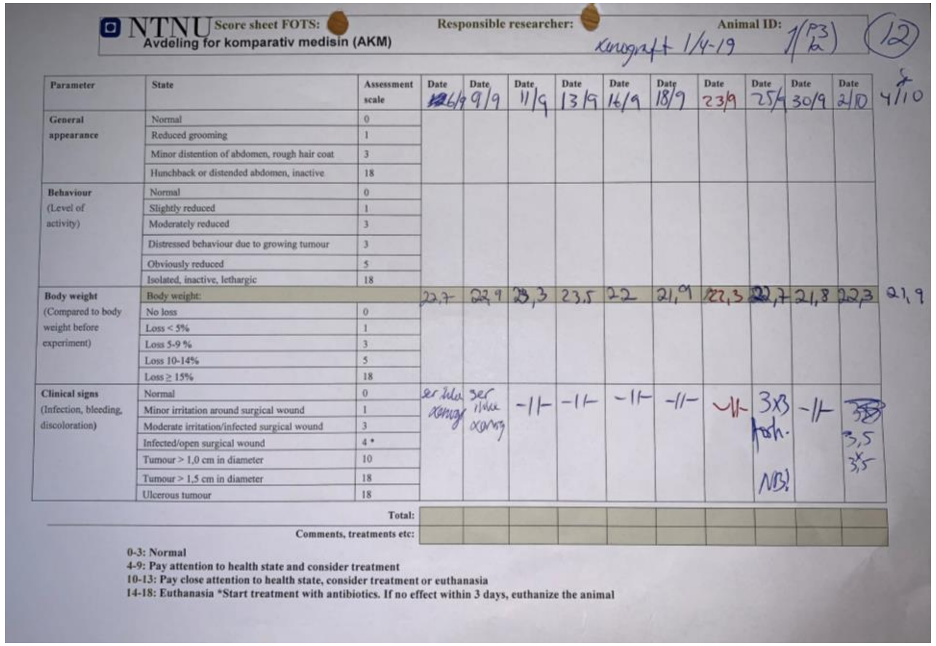

NTNU Score sheet FOTS: Responsible researcher: Animal ID: 1 (P3 1a) (12)
Avdeling for komparativ medisin (AKM)
Xenograft 1/4-19

Parameter	State	Assessment scale	6/9	9/9	11/9	13/9	16/9	18/9	23/9	25/9	30/9	2/10	4/10
Body weight	Body weight:		22.7	22.9	23,3	23.5	22	21,9	22,3	22,7	21,8	22,3	21,9
Clinical signs (Infection, bleeding, discoloration)			ser ikke xenogr	ser ikke xenogr	-ll-	-ll-	-ll-	-ll-	-ll-	3x3 [illegible] NB!	-ll-	3,5 x 3,5	

0-3: Normal
4-9: Pay attention to health state and consider treatment
10-13: Pay close attention to health state, consider treatment or euthanasia
14-18: Euthanasia *Start treatment with antibiotics. If no effect within 3 days, euthanize the animal

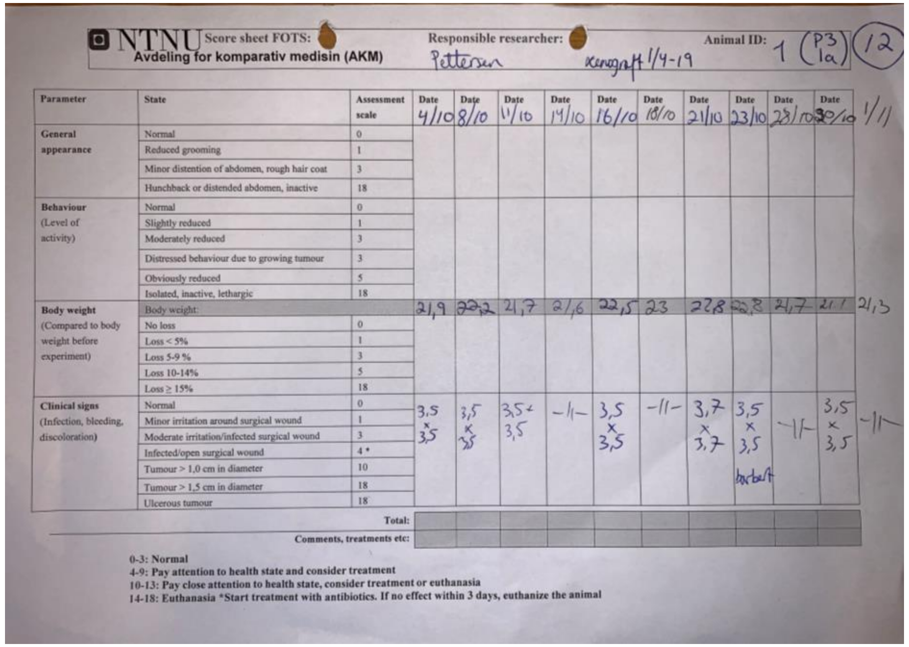

NTNU Score sheet FOTS: Responsible researcher: Pettersen Animal ID: 1 (P3 1a) (12)
Avdeling for komparativ medisin (AKM)
Xenograft 1/4-19

Parameter	State	Assessment scale	4/10	8/10	11/10	14/10	16/10	18/10	21/10	23/10	28/10	30/10	1/11
Body weight	Body weight:		21,9	22,2	21,7	21,6	22,5	23	22,8	22,3	21,7	21,[illegible]	21,3
Clinical signs (Infection, bleeding, discoloration)			3,5 x 3,5	3,5 x 3,5	3,5 x 3,5	-ll-	3,5 x 3,5	-ll-	3,7 x 3,7	3,5 x 3,5 [illegible]	-ll-	3,5 x 3,5	-ll-

0-3: Normal
4-9: Pay attention to health state and consider treatment
10-13: Pay close attention to health state, consider treatment or euthanasia
14-18: Euthanasia *Start treatment with antibiotics. If no effect within 3 days, euthanize the animal

Supplementary figure 1-12-4. Pictures of scoresheets

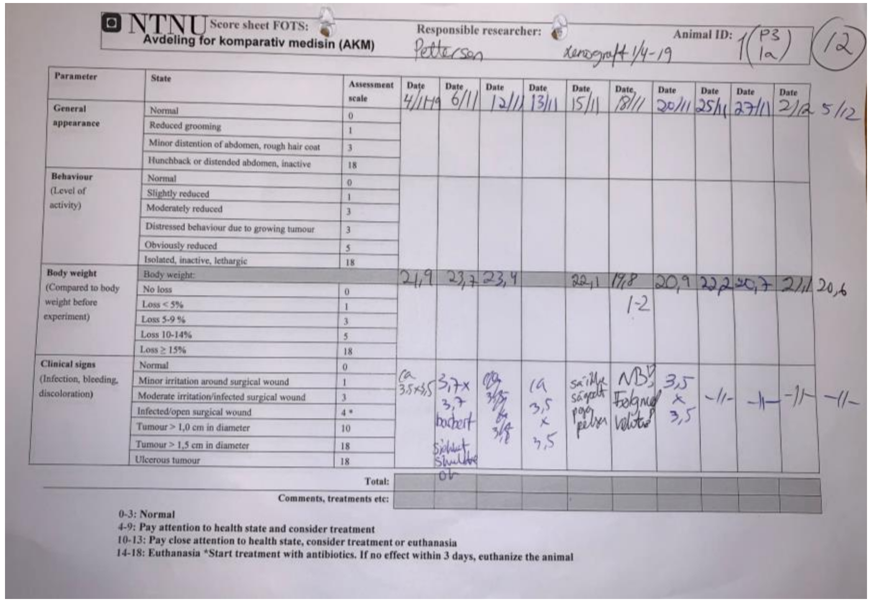

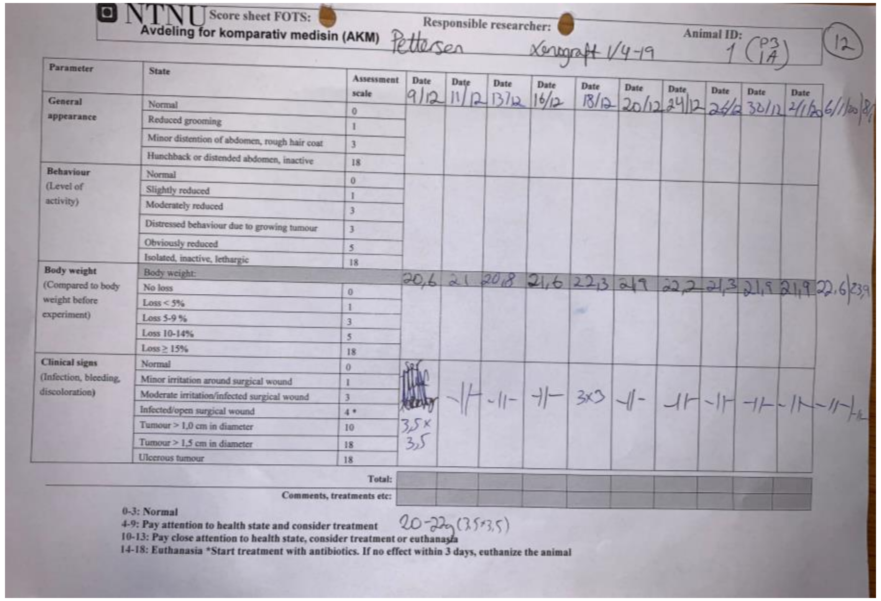

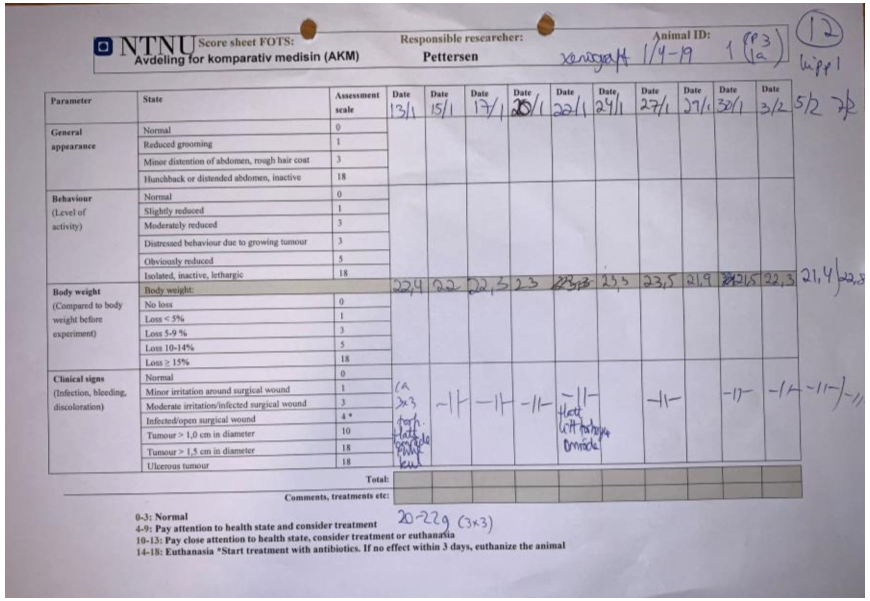

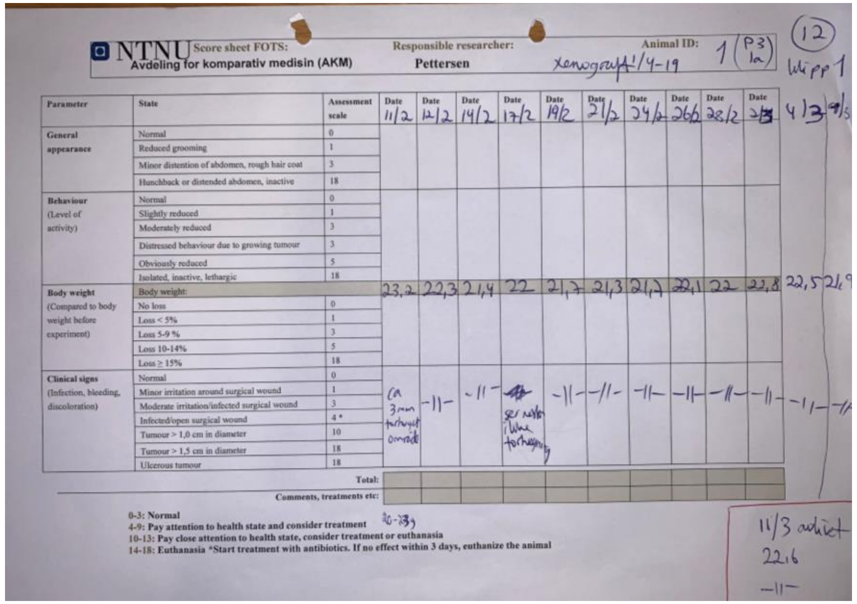

Supplementary figure 1-12-5. Mouse and xenograft growth curves

Weight curve

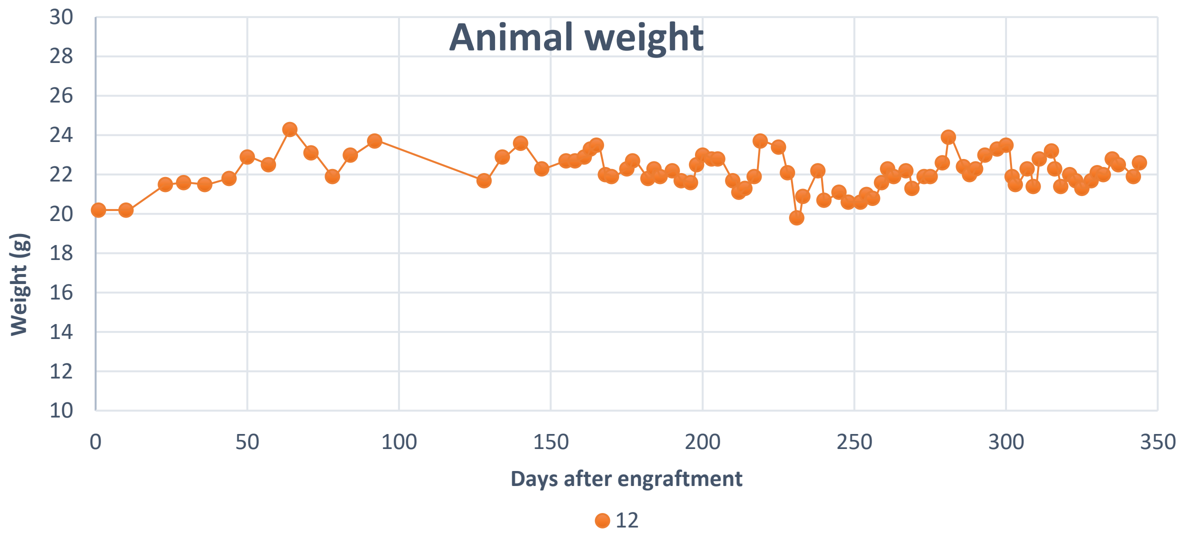

Xenograft growth curve

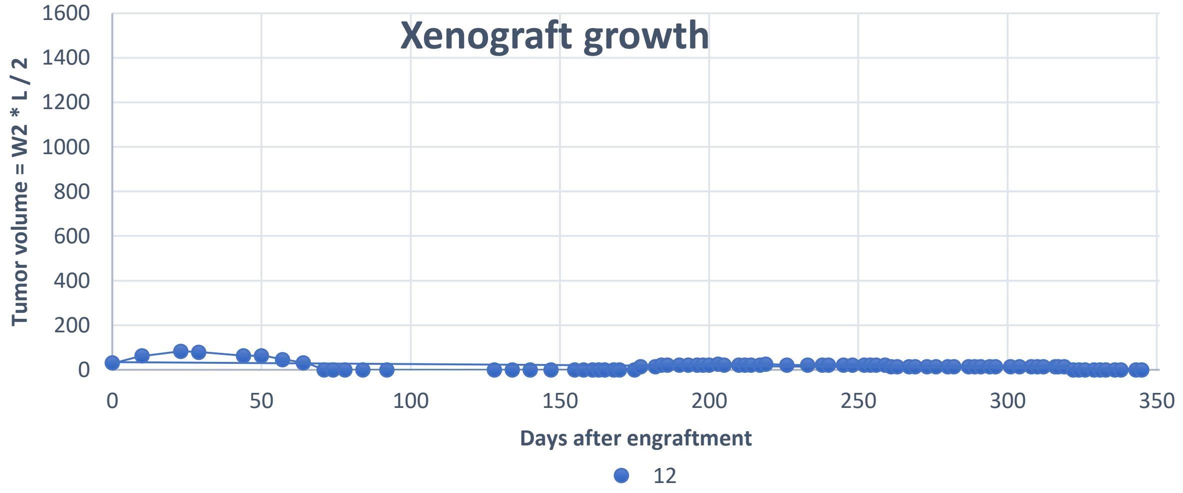

Supplementary figure 1-12-6. Histology pictures

M12X – tissue spot at surgery location at euthanization (thought to be remainings of engrafted tumor material)

- Stained with hematoxylin, eosine and saphron (HES)
- Probably not xenograft tissue
- No tumor cells found
- Muscle cells, fat, mucus, connective tissue
- Traces of plant cells outside but also inside tissue? Possibly paper leftovers.

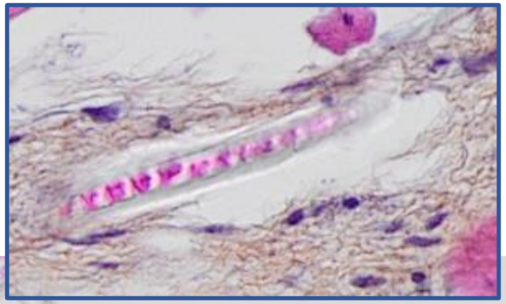

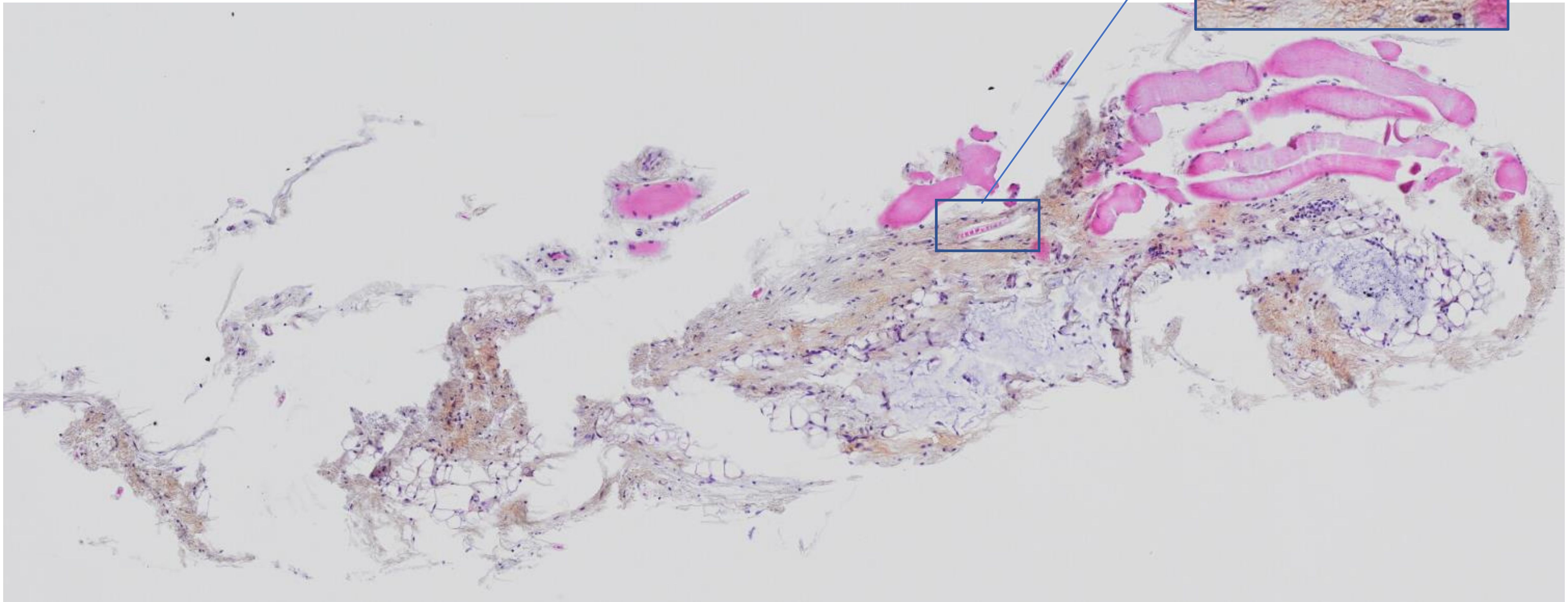

Supplementary figure 1-12-6. Histology pictures

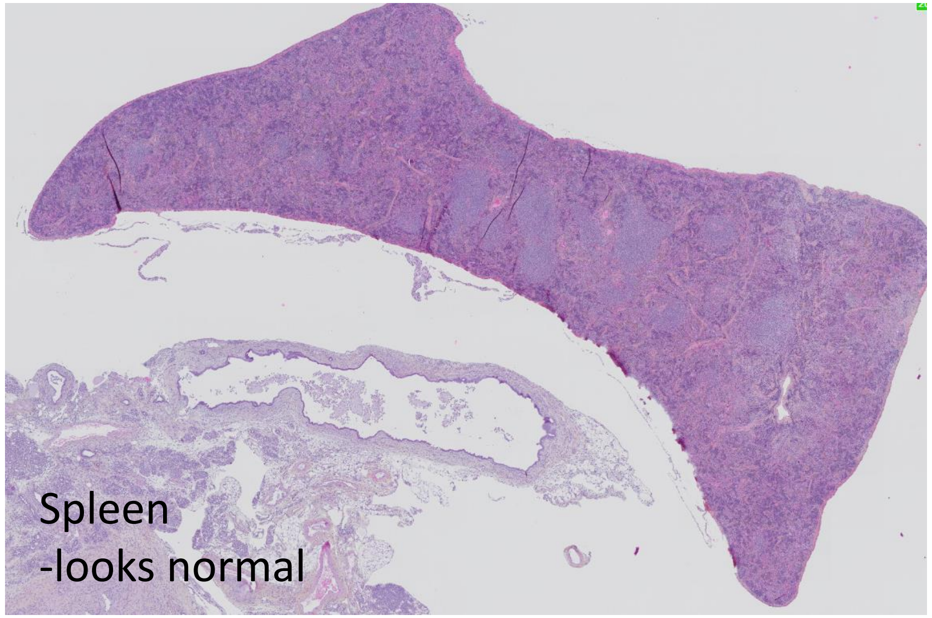

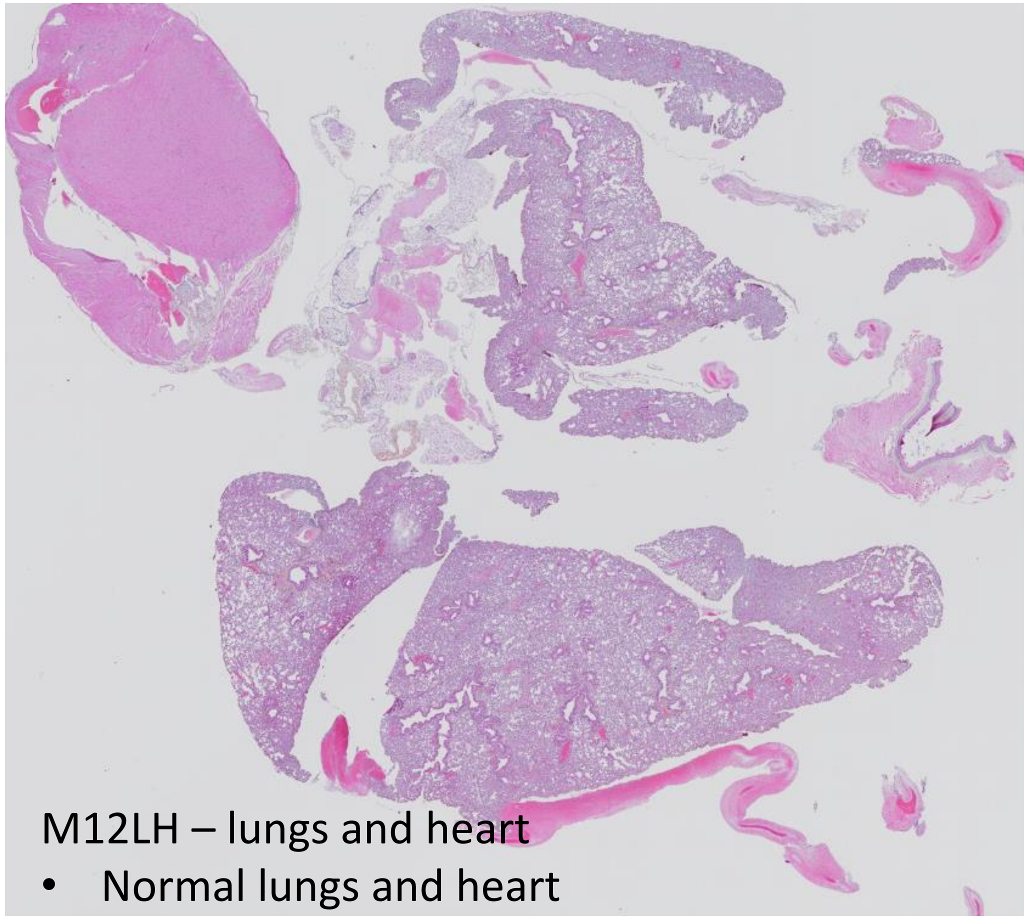

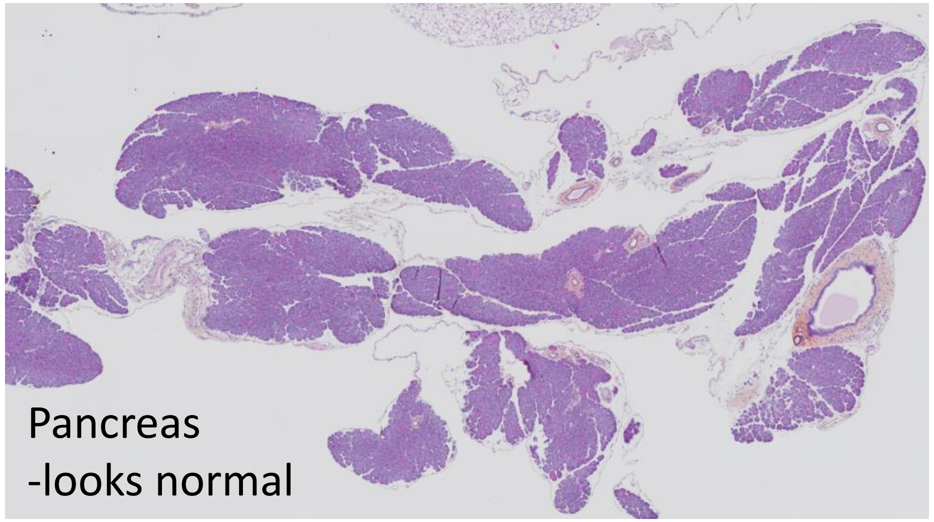

Mouse 15

- Surgery date: 01.04.18
  - Age (weeks) 11
  - Weight (g) 18.5
  - Tumor fragment P3-1B
  - Ear marks 2

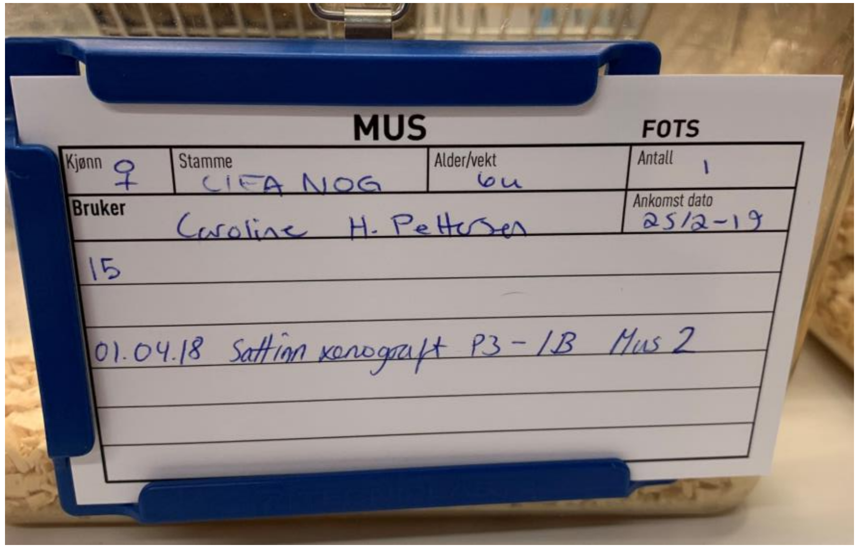

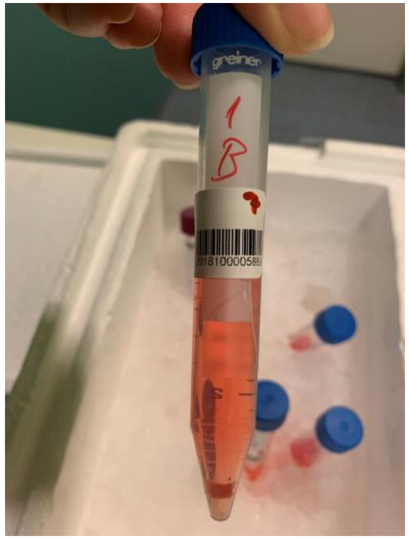

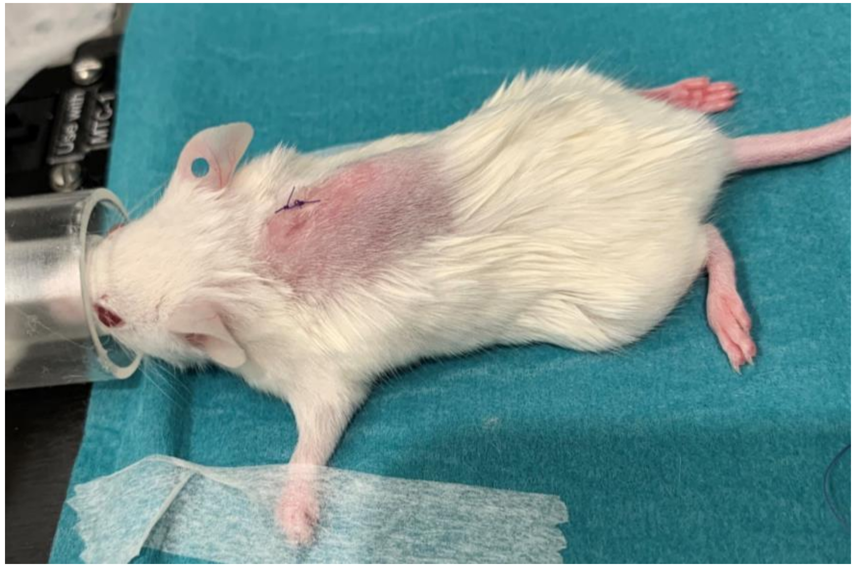

Supplementary figure 1-15-1. Pictures from the engraftment day

Supplementary figure 1-15-2. Pictures from follow up(14.06.19) and score sheets

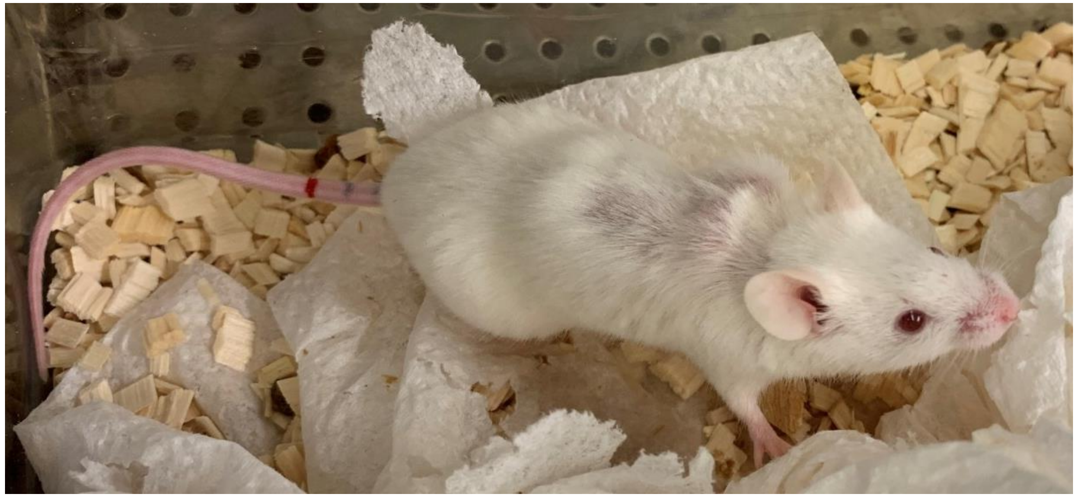

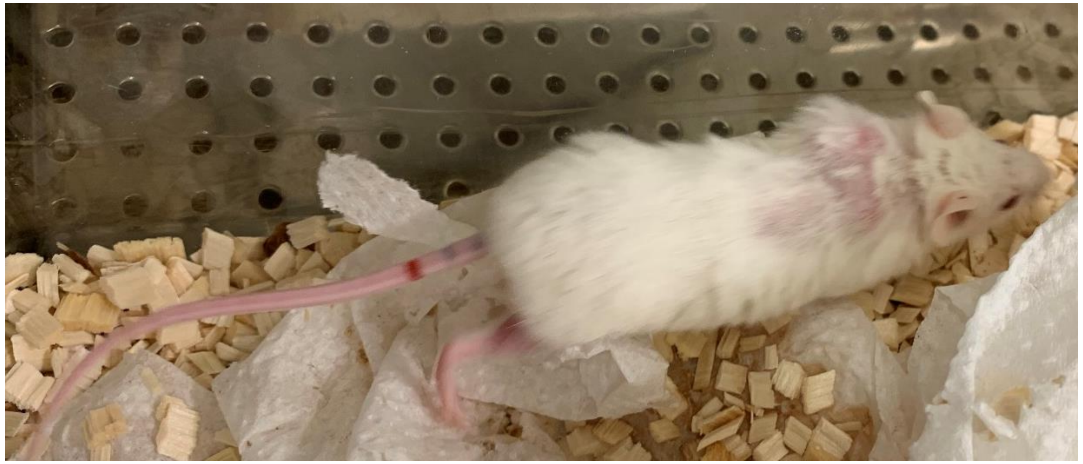

Blood sample and treatment

- Blood samples before and after treatment
  - Before: Vena Saphena
  - After: heart
- Placebo treatment

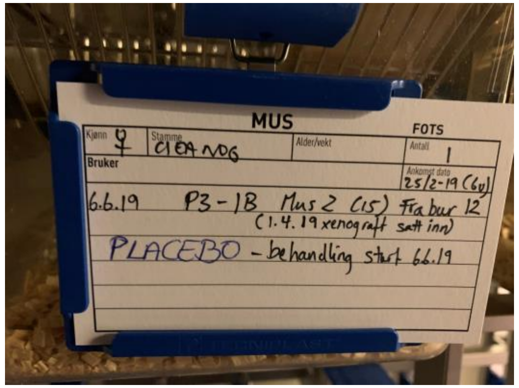

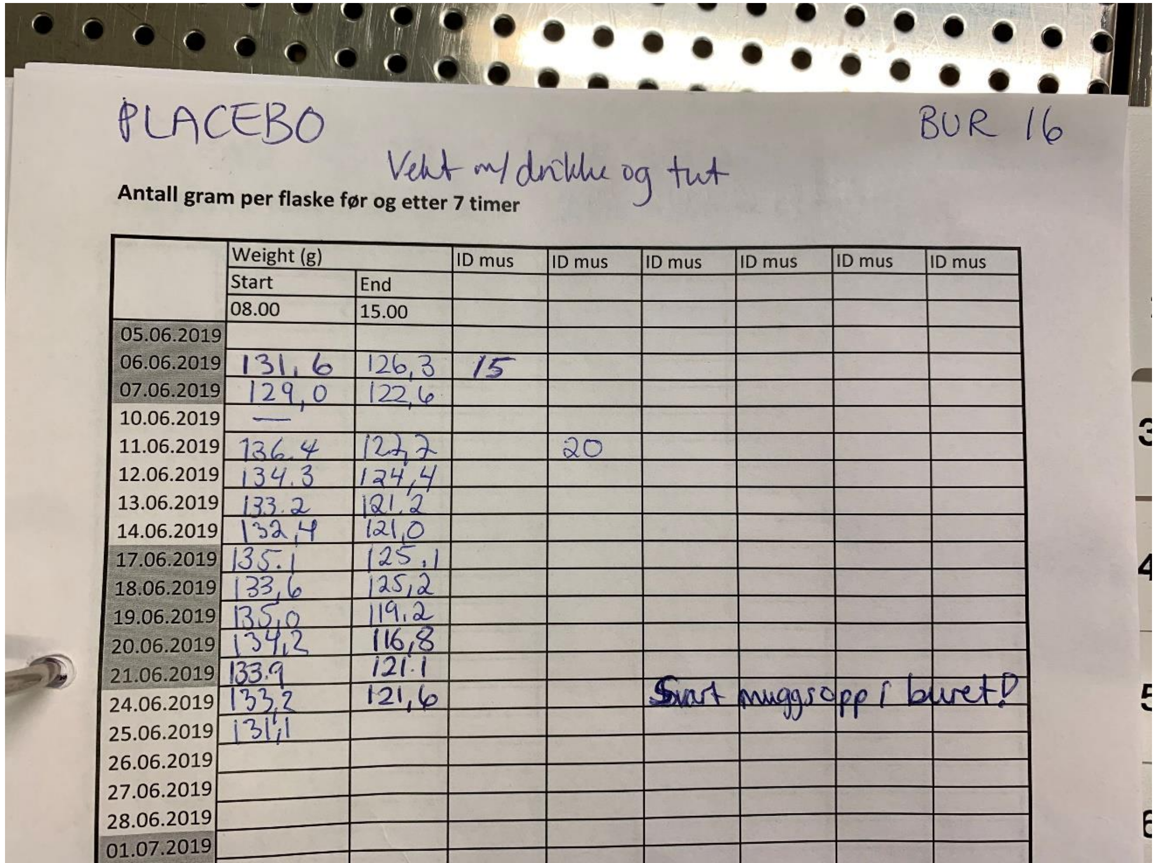

PLACEBO BUR 16

Vekt m/ drikke og tut

Antall gram per flaske før og etter 7 timer

	Weight (g)		ID mus	ID mus	ID mus	ID mus	ID mus	ID mus
	Start	End						
	08.00	15.00						
05.06.2019								
06.06.2019	131,6	126,3	15					
07.06.2019	129,0	122,6						
10.06.2019	—							
11.06.2019	136.4	122,2		20				
12.06.2019	134.3	124,4						
13.06.2019	133.2	121,2						
14.06.2019	132,4	121,0						
17.06.2019	135.1	125,1						
18.06.2019	133,6	125,2						
19.06.2019	135,0	119,2						
20.06.2019	134,2	116,8						
21.06.2019	133.9	121.1						
24.06.2019	133,2	121,6			Svart muggsopp i buret!			
25.06.2019	131,1							
26.06.2019								
27.06.2019								
28.06.2019								
01.07.2019								

Supplementary figure 1-15-3. List over treatment with placebo. Numbers are weight in grams before and after provided to the mice.

Supplementary figure 1-15-4. Pictures from euthanization (25.06.19)

- Necroscopy
  - Xenograft size 15.5 x 10
  - Blood sample (blood card and plasma)
  - Tumor tissue for
    - Histology
    - RNA isolation
    - Protein isolation

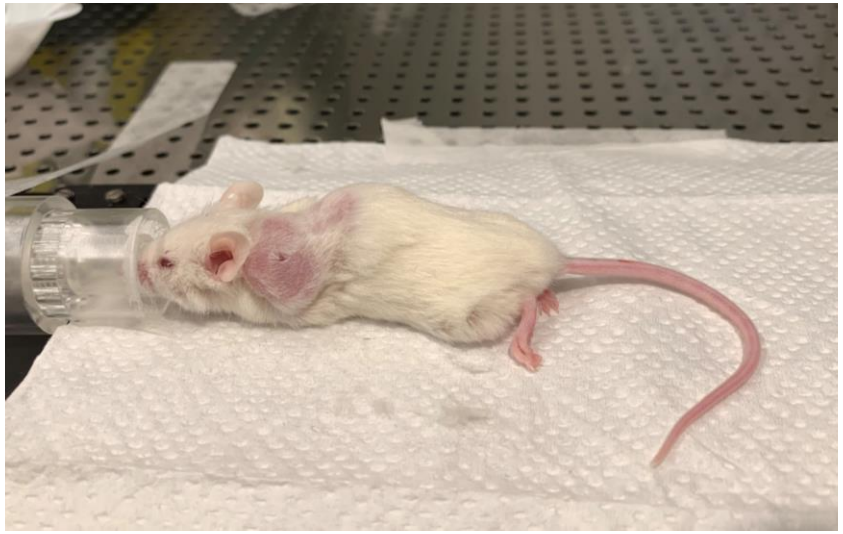

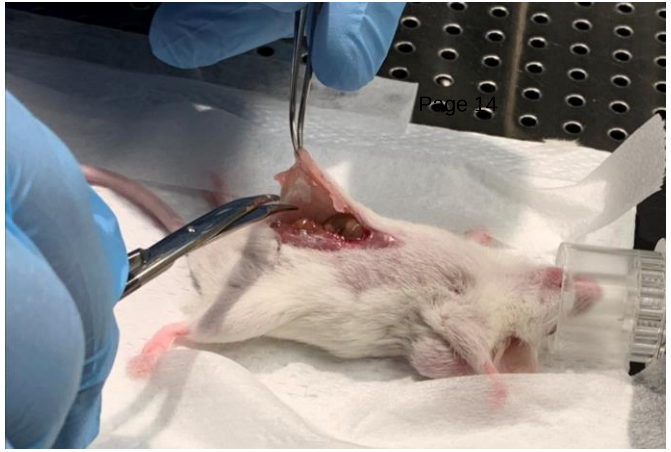

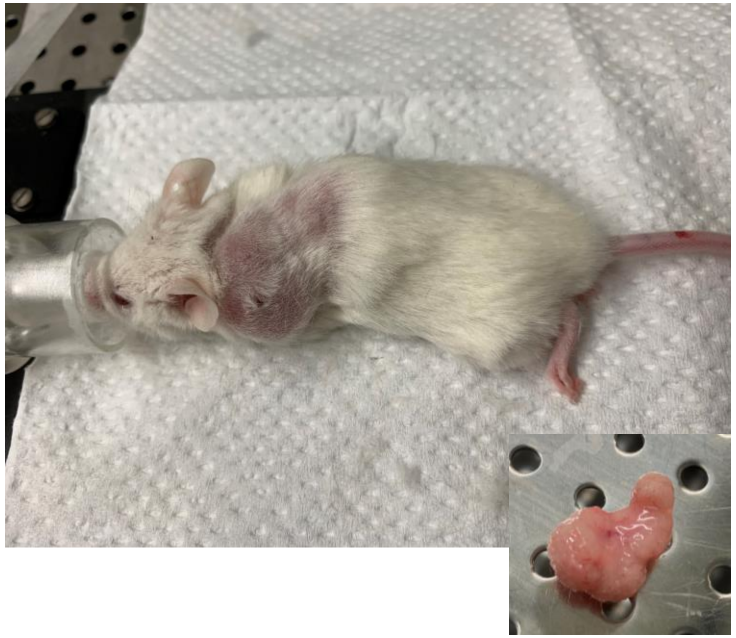

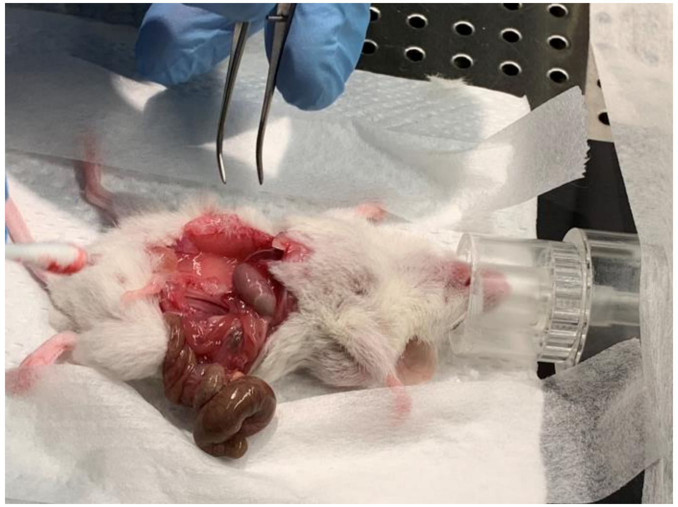

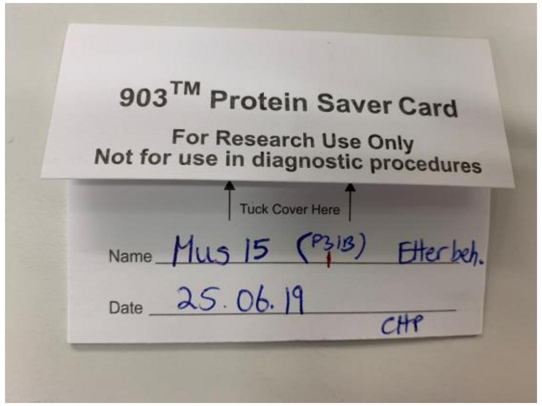

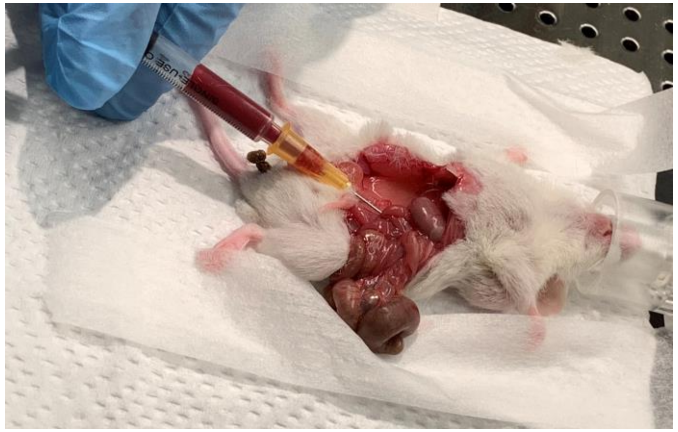

Supplementary figure 1-15-5. Pictures of score sheets

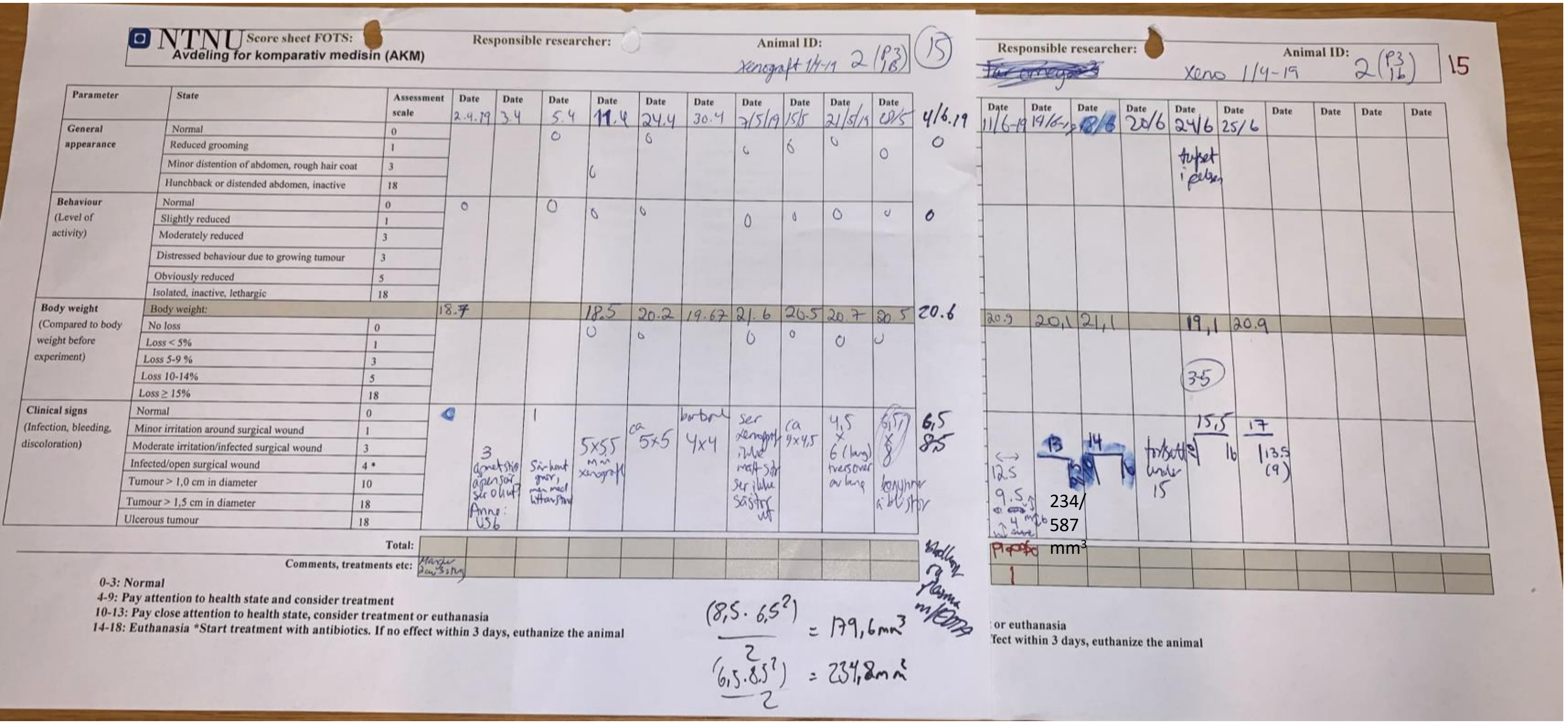
NTNU Score sheet FOTS: Responsible researcher: Animal ID: xenograft 1/4-19 2 (P3/1B) 15
Avdeling for komparativ medisin (AKM)

Parameter	State	Assessment scale	Date 2.4.19	Date 3.4	Date 5.4	Date 11.4	Date 24.4	Date 30.4	Date 7/5/19	Date 15/5	Date 21/5/19	Date 28/5	4/6.19
General appearance	Normal	0			0	0	0		0	0	0	0	0
	Reduced grooming	1											
	Minor distention of abdomen, rough hair coat	3											
	Hunchback or distended abdomen, inactive	18											
Behaviour (Level of activity)	Normal	0	0		0	0	0		0	0	0	0	0
	Slightly reduced	1											
	Moderately reduced	3											
	Distressed behaviour due to growing tumour	3											
	Obviously reduced	5											
	Isolated, inactive, lethargic	18											
Body weight (Compared to body weight before experiment)	Body weight:		18.7			18.5	20.2	19.67	21.6	20.5	20.7	20.5	20.6
	No loss	0				0	0		0	0	0	0	
	Loss < 5%	1											
	Loss 5-9 %	3											
	Loss 10-14%	5											
	Loss ≥ 15%	18											
Clinical signs (Infection, bleeding, discoloration)	Normal	0		3 [illegible]	1 [illegible]	5x5.5 mm xenograft	ca 5x5	4x4	ser xenograft [illegible]	ca 9x4,5	4,5 x 6 [illegible]	6,5 x 8 [illegible]	6,5 8,5
	Minor irritation around surgical wound	1											
	Moderate irritation/infected surgical wound	3											
	Infected/open surgical wound	4 *											
	Tumour > 1,0 cm in diameter	10											
	Tumour > 1,5 cm in diameter	18											
	Ulcerous tumour	18											
Total:													
Comments, treatments etc:													

0-3: Normal
4-9: Pay attention to health state and consider treatment
10-13: Pay close attention to health state, consider treatment or euthanasia
14-18: Euthanasia *Start treatment with antibiotics. If no effect within 3 days, euthanize the animal

$\frac{(8{,}5 \cdot 6{,}5^2)}{2} = 179{,}6 mm^3$

$\frac{(6{,}5 \cdot 8{,}5^2)}{2} = 234{,}8 mm^3$

Responsible researcher: Animal ID: Xeno 1/4-19 2 (P3/1B) 15

Date 11/6-19	Date 19/6	Date [illegible]	Date 20/6	Date 24/6	Date 25/6	Date	Date	Date	Date
				[illegible]					
20.9	20,1	21,1		19,1	20.9				
				3-5					
12.5 9.5 [illegible]	13	14	[illegible] 15	15,5 16	17 13.5 (9)				

234/ 587 mm³

or euthanasia
fect within 3 days, euthanize the animal

Supplementary figure 1-15-6. Mouse and xenograft growth curves

Weight curve

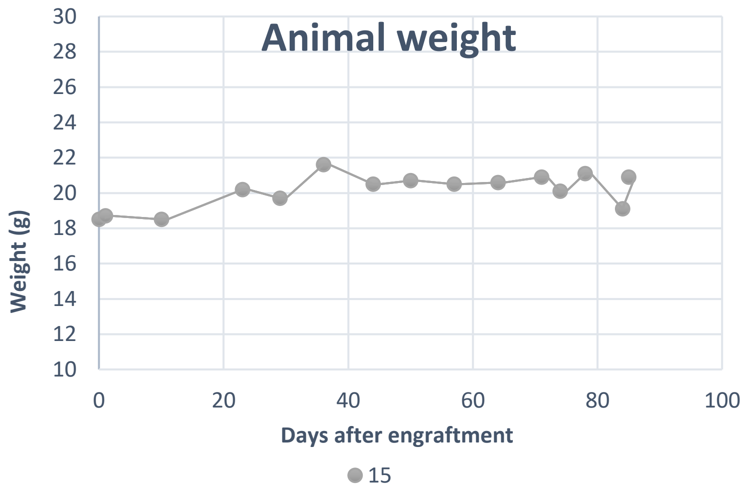

Xenograft growth curve

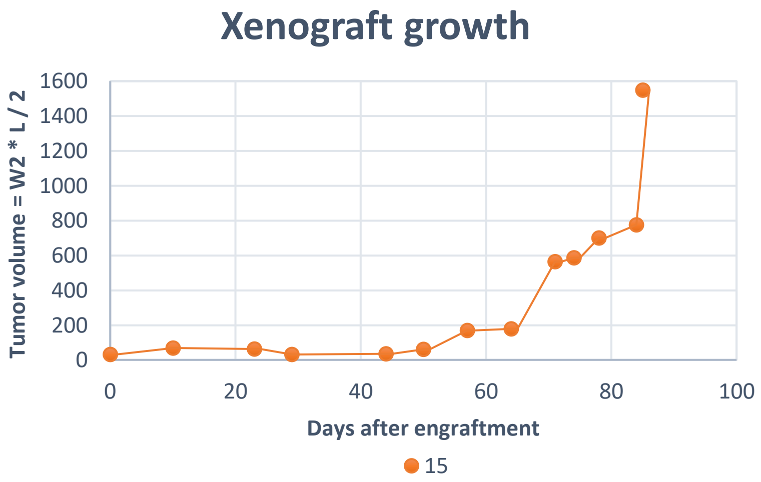

Supplementary figure 1-15-7. Histology pictures

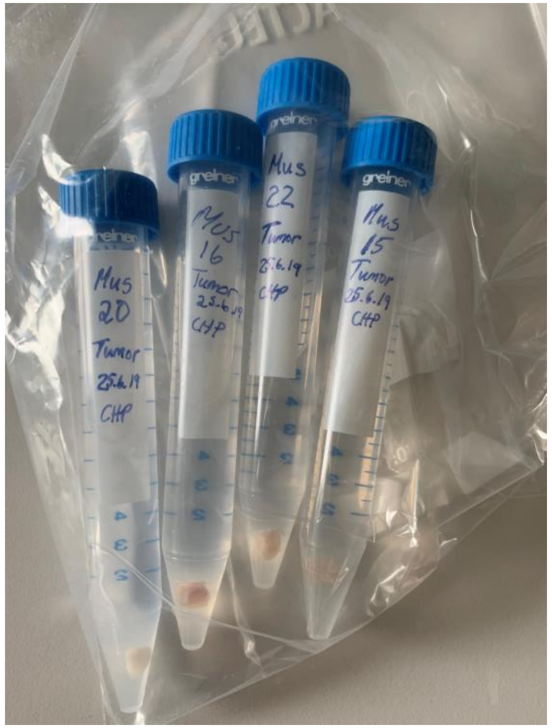

M15X

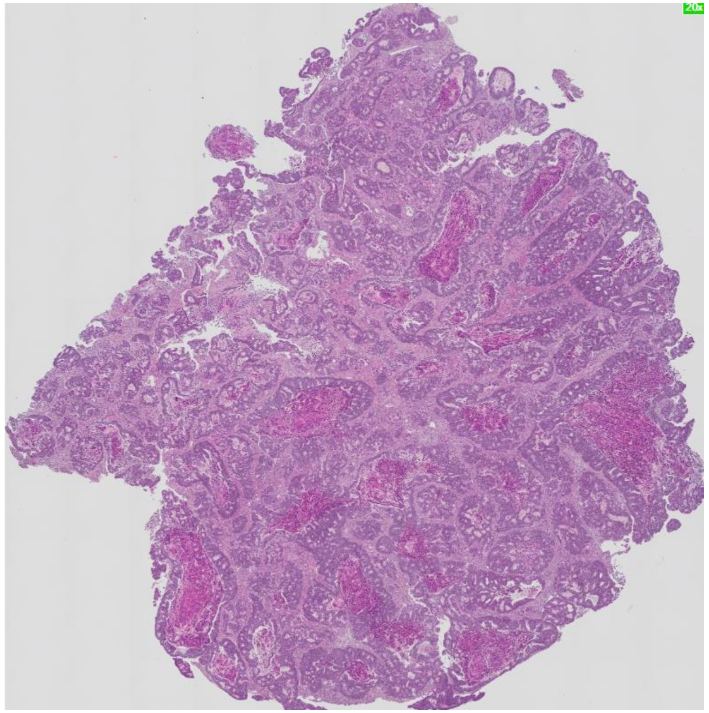

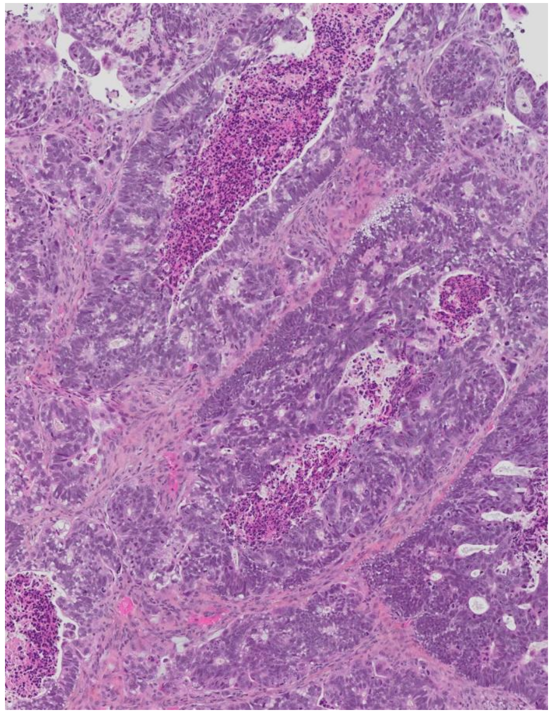

- Typical colorectal adenocarcinoma
- Very similar to the HES staining og patient tumor 3
- Gland forming cells
- Necrotic debris withing glandular lumen

Mouse 16

- Surgery date: 01.04.19
  - Age (weeks) 11
  - Weight (g) 18.2
  - Tumor fragment P3-2A
  - Ear marks 3

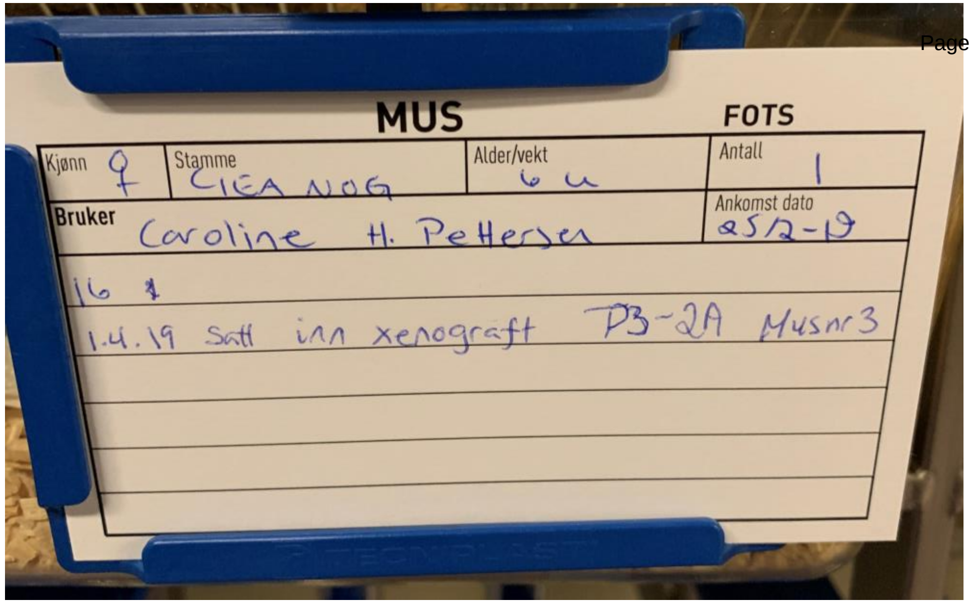

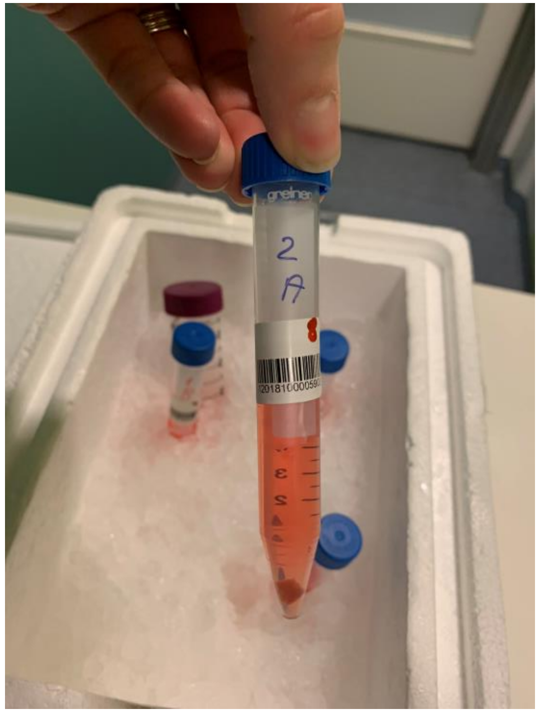

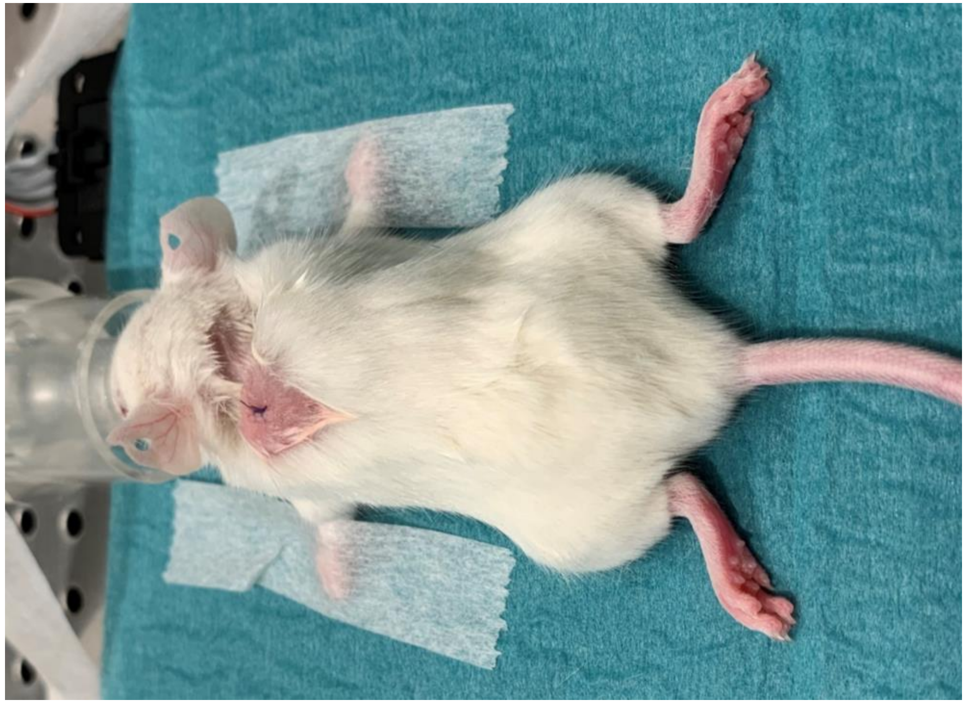

Supplementary figure 1-16-1.
Pictures from the engraftment day

Supplementary figure 1-16-2.

Pictures from follow up (14.06.19)

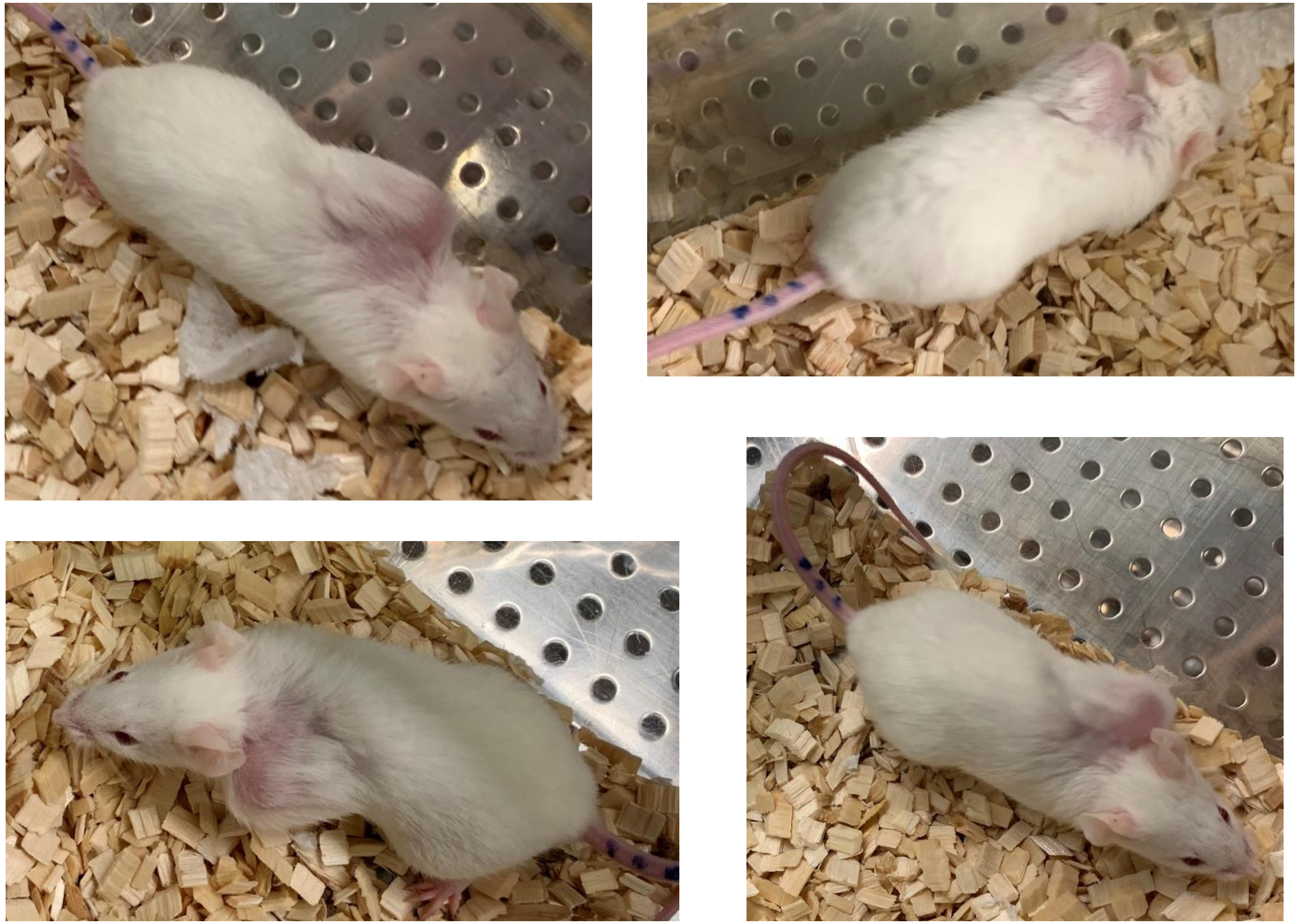

Blood sample and treatment

- Before: Vena Saphena
- After: heart 04.06.19
- Treatment: Smartfish with fishoil (OMEGA-3)

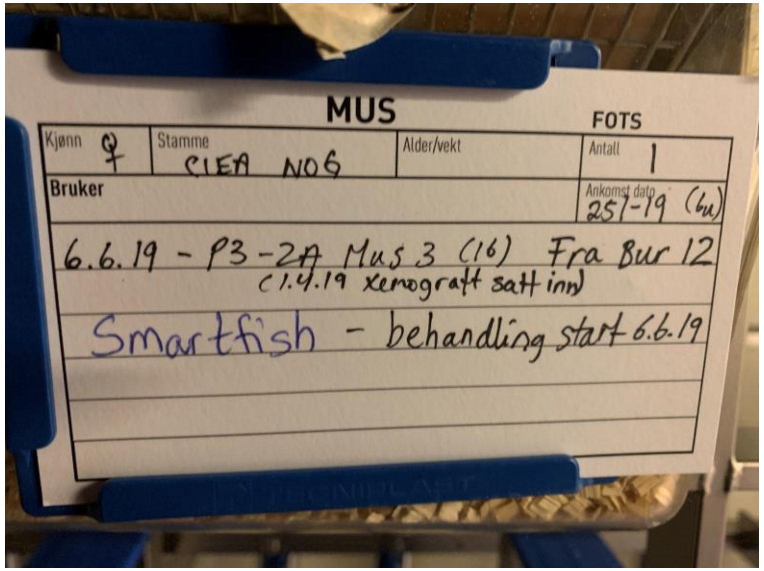

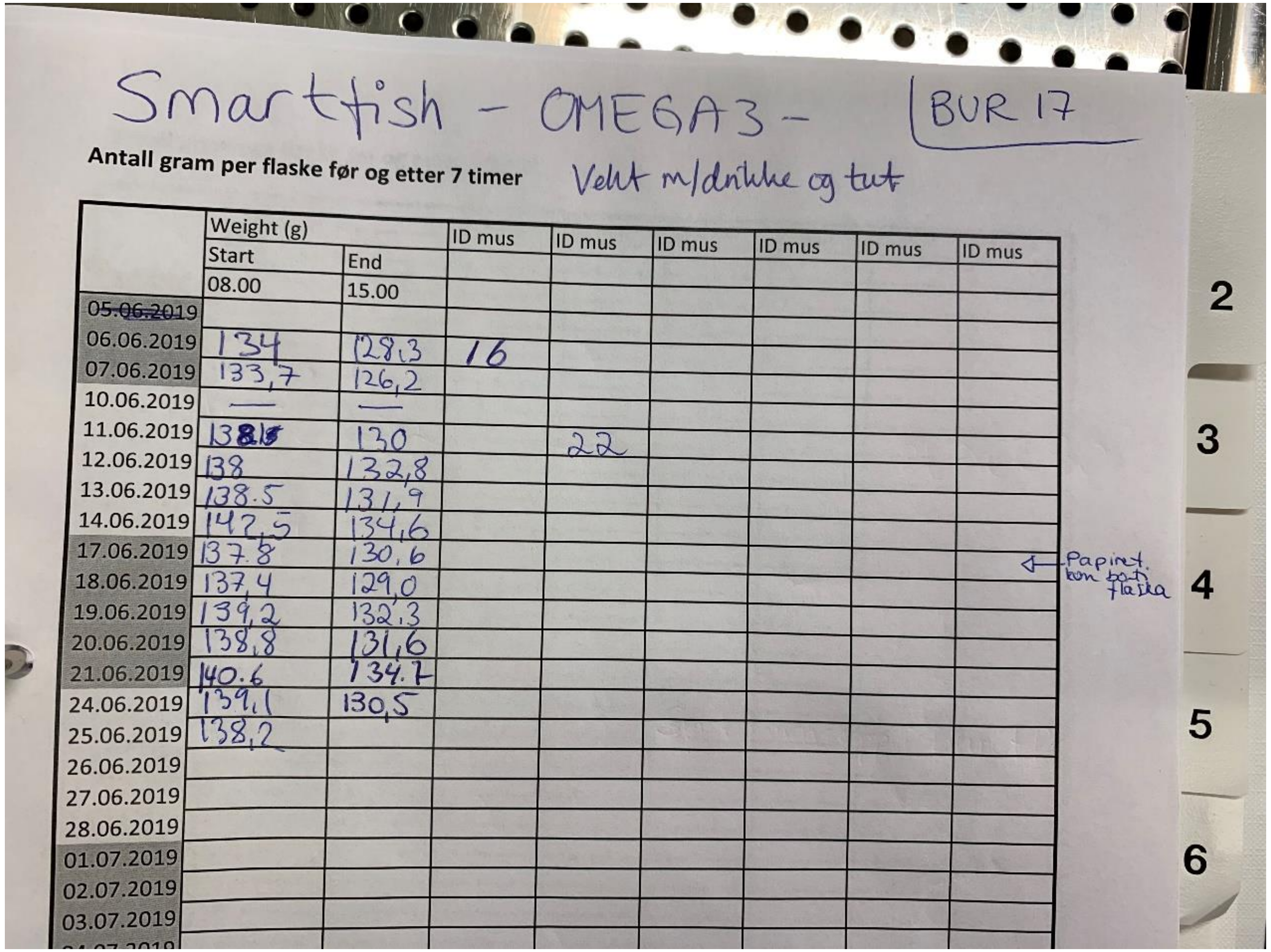

Smartfish - OMEGA3 - BUR 17

Antall gram per flaske før og etter 7 timer   Veht m/drikke og tut

	Weight (g)		ID mus	ID mus	ID mus	ID mus	ID mus	ID mus
	Start 08.00	End 15.00						
~~05.06.2019~~								
06.06.2019	134	128,3	16					
07.06.2019	133,7	126,2						
10.06.2019	—	—						
11.06.2019	138[illegible]	130		22				
12.06.2019	138	132,8						
13.06.2019	138.5	131,9						
14.06.2019	142,5	134,6						
17.06.2019	137.8	130,6						
18.06.2019	137,4	129,0						
19.06.2019	139,2	132,3						
20.06.2019	138,8	131,6						
21.06.2019	140.6	134.7						
24.06.2019	139,1	130,5						
25.06.2019	138,2							
26.06.2019								
27.06.2019								
28.06.2019								
01.07.2019								
02.07.2019								
03.07.2019								

← Papiret kom bak flaska

2 3 4 5 6

Supplementary figure 1-16-3. List with weight registrations of the drinking bottle (with Smartfish Remune Peach drink) before and after provided to the mice

Supplementary figure 1-16-4. Pictures from euthanization

- Xenograft size 13 x 11.5
- Xenograft partly liquid
- Visiable blood vain formation
- Blood sample (blood card and plasma)
- Tumor tissue for
  - Histology
  - RNA isolation
  - Protein isolation

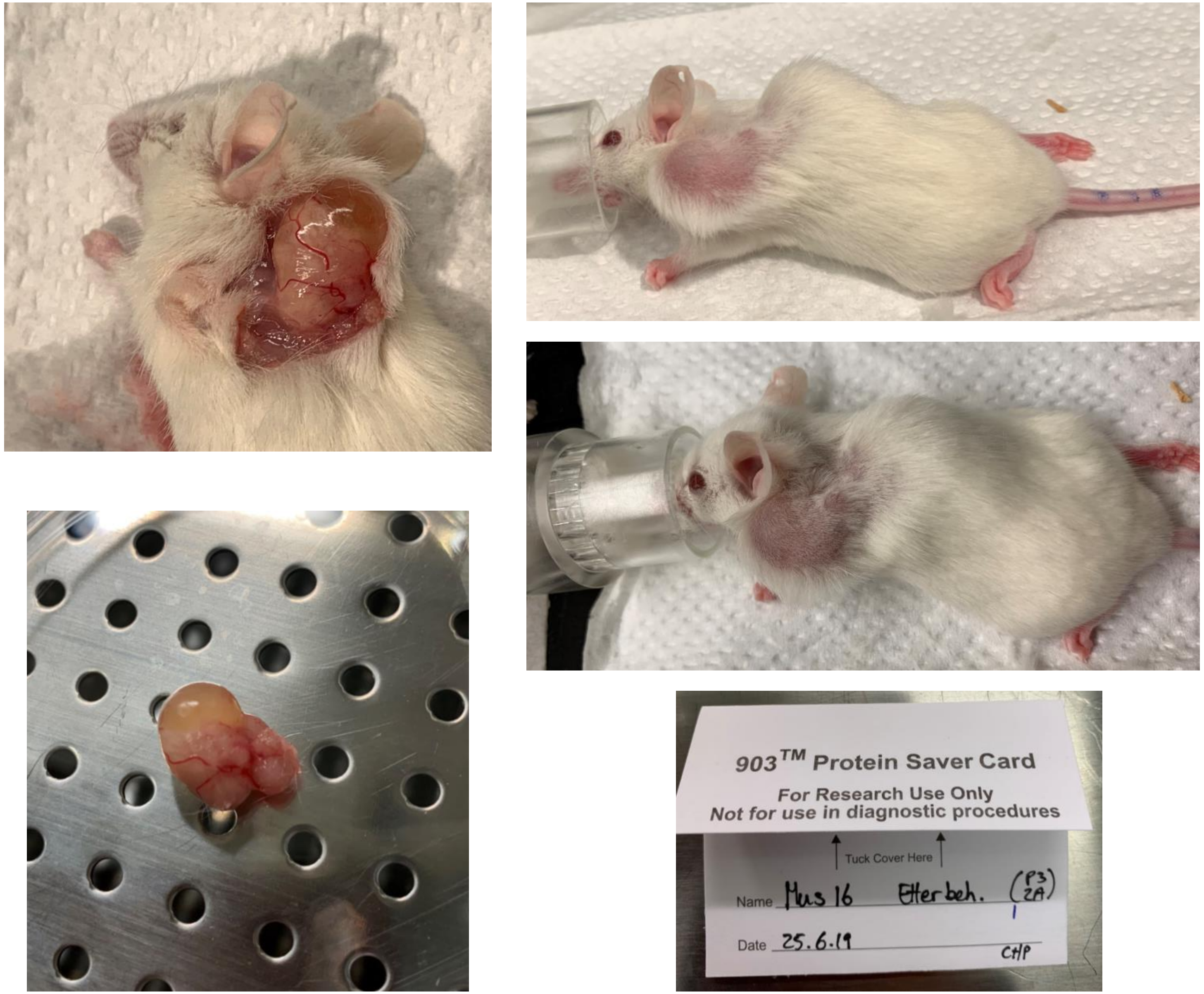

Supplementary figure 1-16-5. Pictures of score sheets

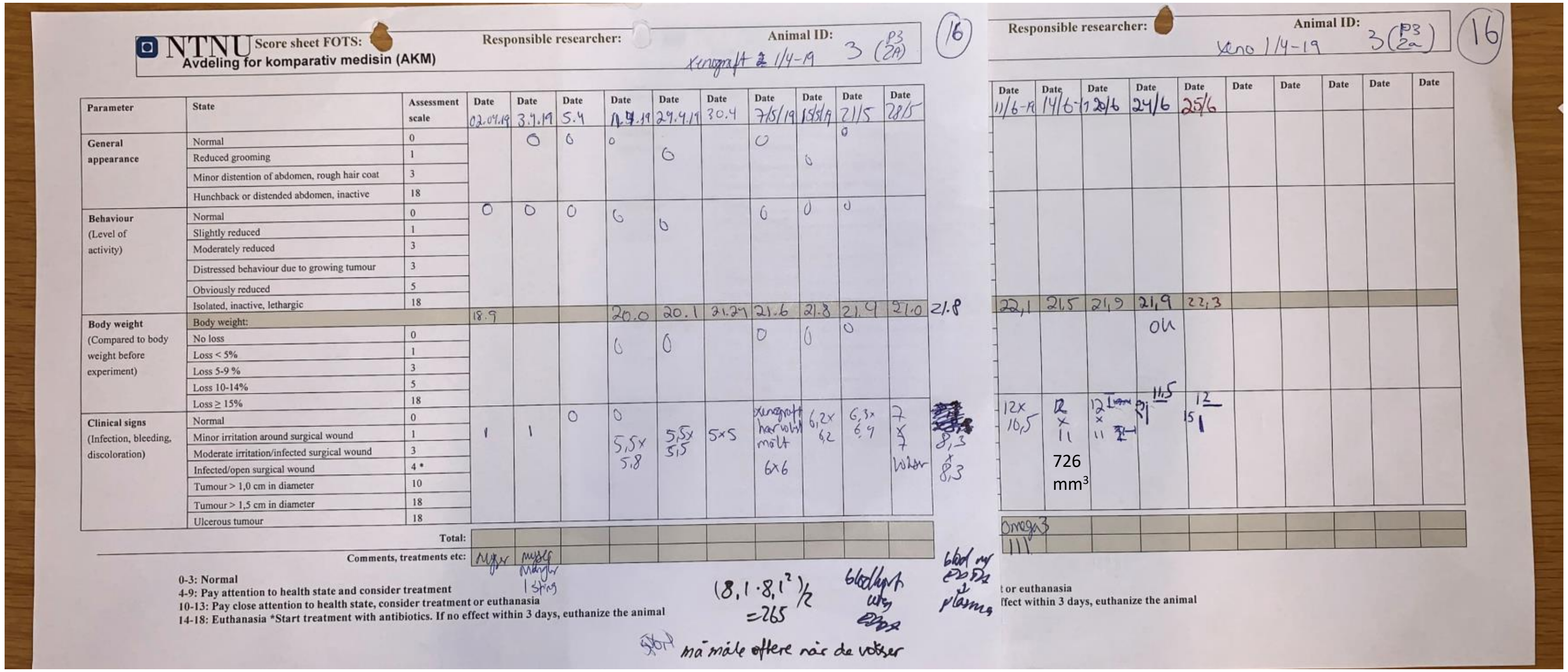

Supplementary figure 1-16-6. Mouse and xenograft growth curves

Weight curve

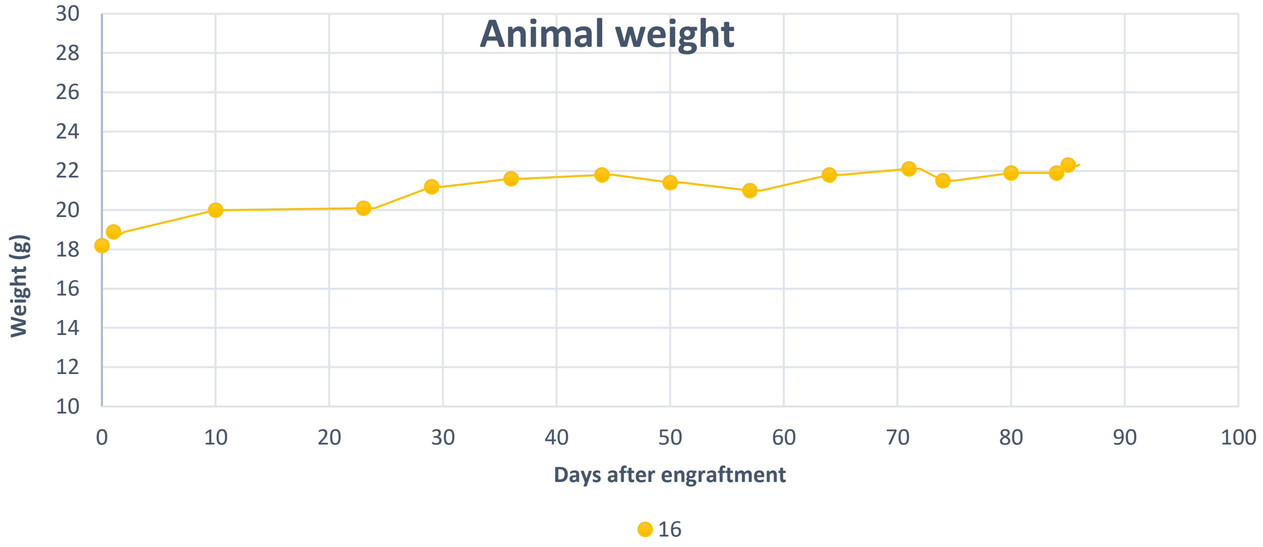

Xenograft growth curve

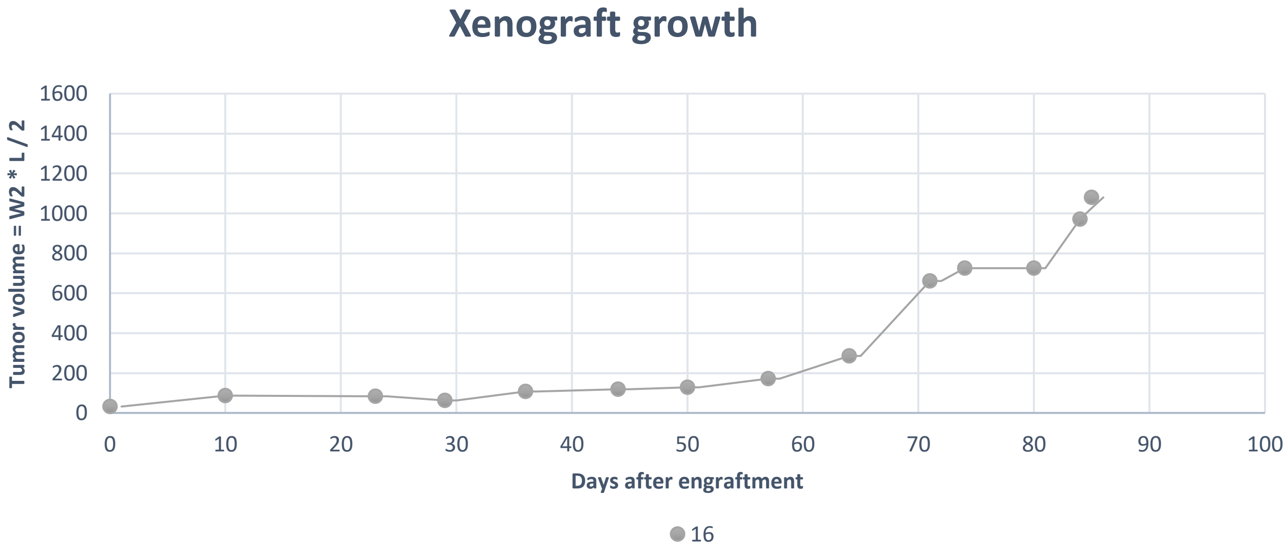

Supplementary figure 1-16-7. Histology pictures

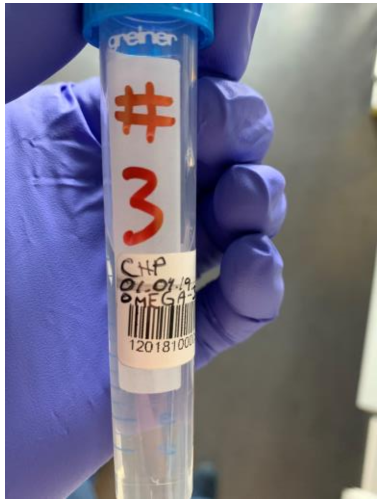

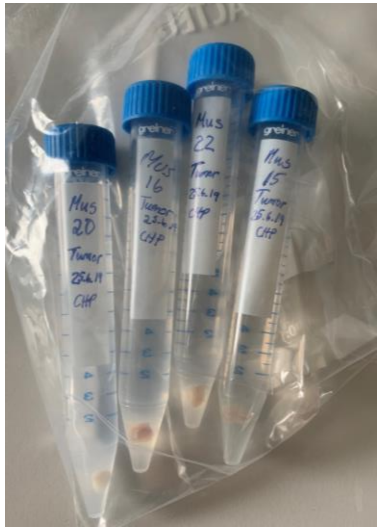

M16X

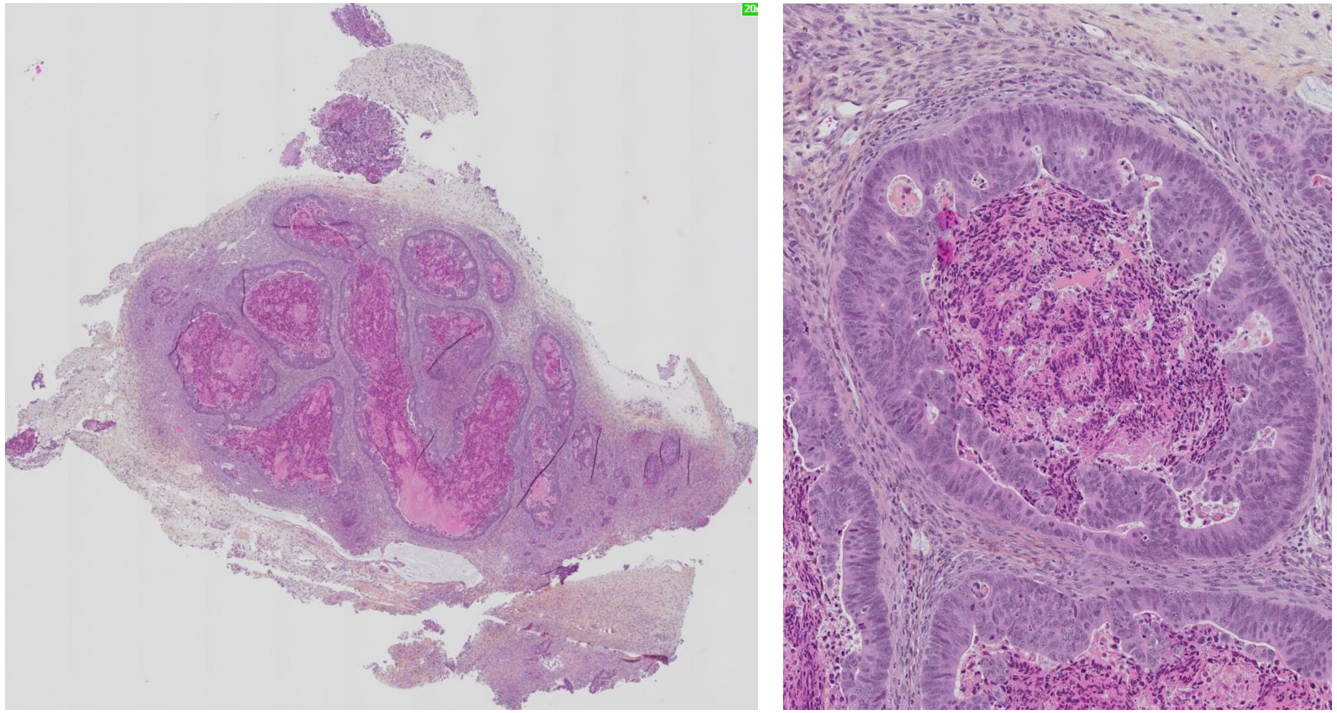

- Typical colorectal adenocarcinoma
- Very similar to the HES staining of patient tumor 3
- Necrotic debri within glandulas

Mouse 17

- Surgery date: 01.04.19
  - Age (weeks) 11
  - Weight (g) 18.4
  - Tumor fragment P3-2B
  - Ear marks 4

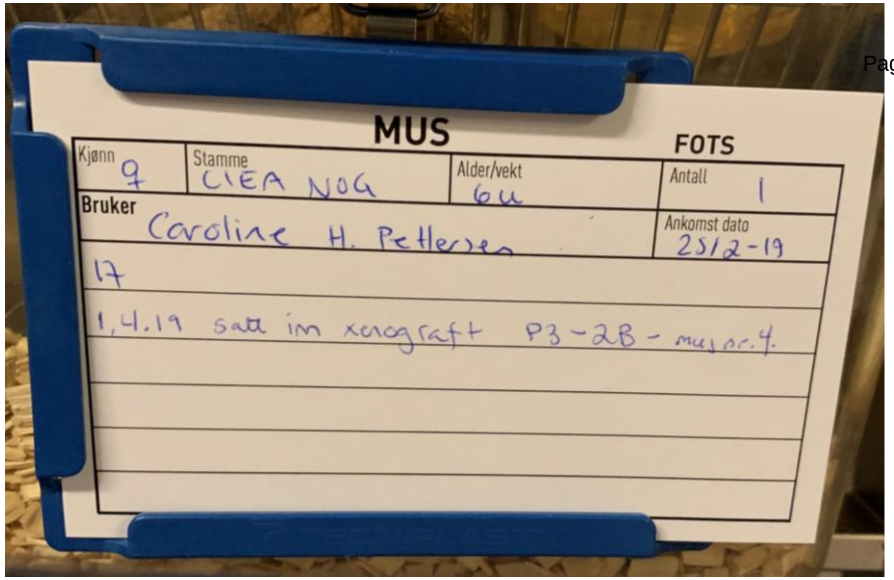

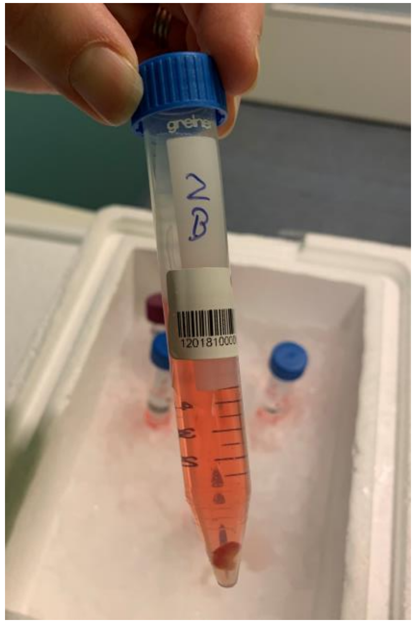

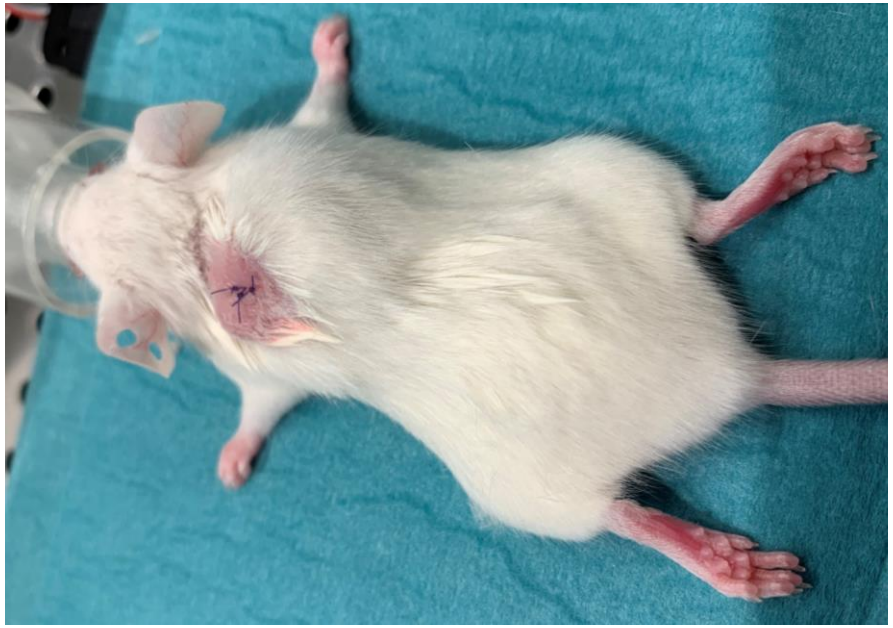

Supplementary figure 1-17-1.
Pictures from the engraftment day

Supplementary figure 1-17-2. List over weight registrations of the drinking bottle before and after provided to the mice

Treatment:

- Smartfish with fishoil (OMEGA-3) ; started 04.07.19.
  - Xenograft 7.5 x 7.5 mm
  - Mouse 12 was used as social animal together wtih mouse 17
- Died 12.07.19 during anasthesia
- Xenograft not sampled
- Xenograft not pictured

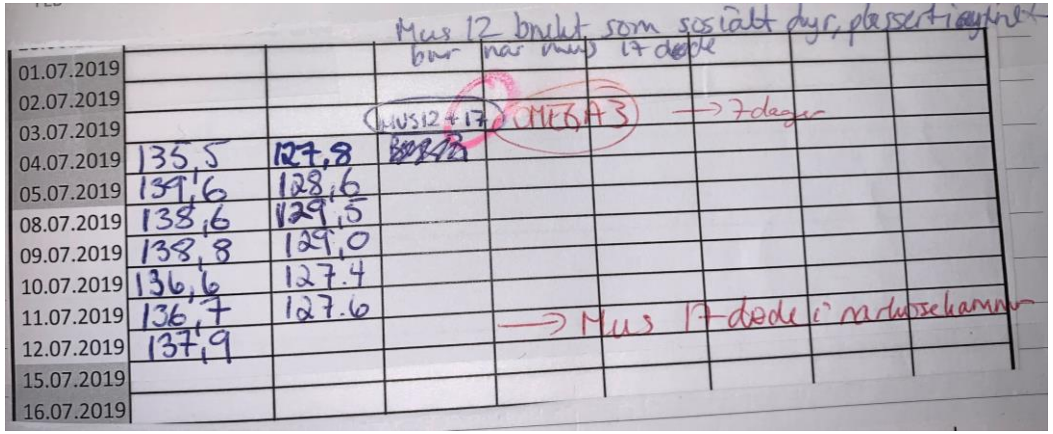

01.07.2019		
02.07.2019		
03.07.2019		
04.07.2019	135,5	127,8
05.07.2019	139,6	128,6
08.07.2019	138,6	129,5
09.07.2019	138,8	129,0
10.07.2019	136,6	127.4
11.07.2019	136,7	127.6
12.07.2019	137,9	
15.07.2019		
16.07.2019		

Supplementary figure 1-17-3. Pictures of score sheets

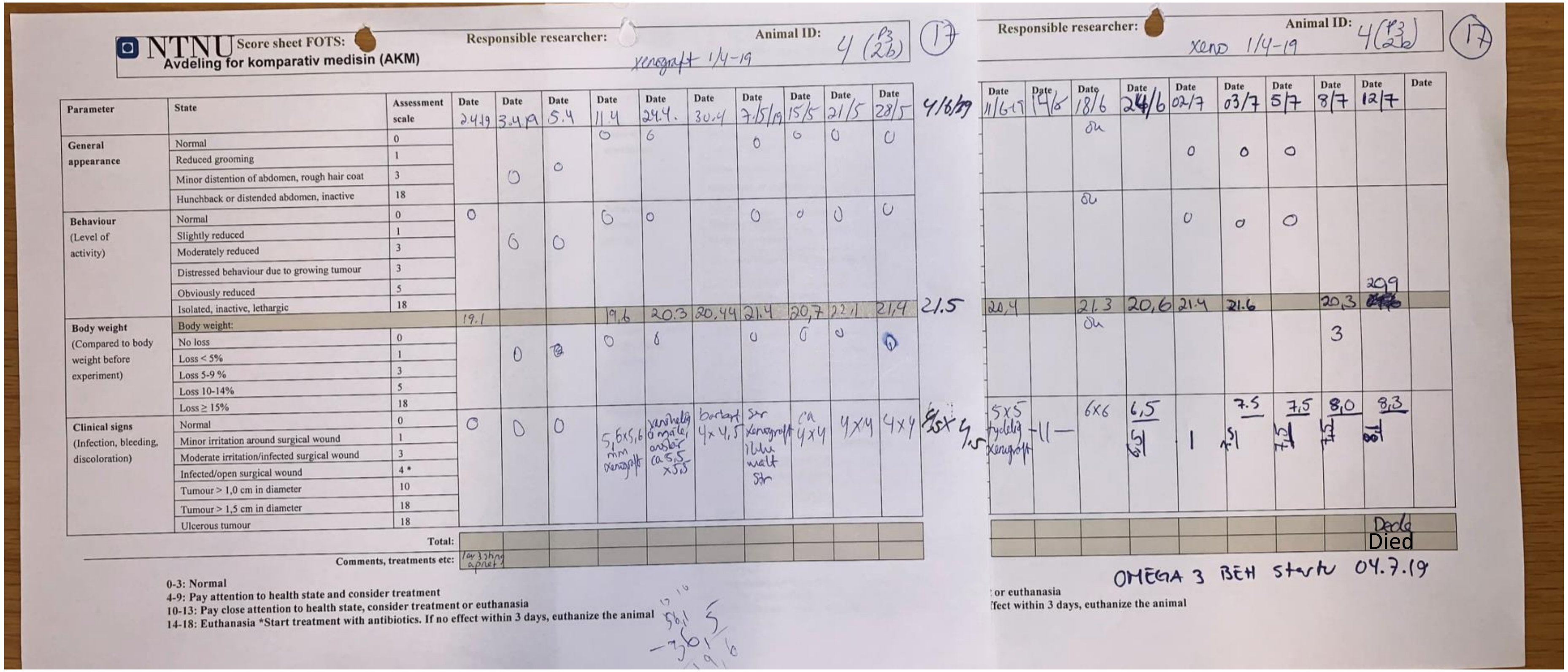
NTNU Score sheet FOTS: Avdeling for komparativ medisin (AKM)

Responsible researcher: xenograft 1/4-19

Animal ID: 4 (P3 2b) 17

Parameter	State	Assessment scale
General appearance	Normal	0
	Reduced grooming	1
	Minor distention of abdomen, rough hair coat	3
	Hunchback or distended abdomen, inactive	18
Behaviour (Level of activity)	Normal	0
	Slightly reduced	1
	Moderately reduced	3
	Distressed behaviour due to growing tumour	3
	Obviously reduced	5
	Isolated, inactive, lethargic	18
Body weight (Compared to body weight before experiment)	Body weight:	
	No loss	0
	Loss < 5%	1
	Loss 5-9 %	3
	Loss 10-14%	5
	Loss ≥ 15%	18
Clinical signs (Infection, bleeding, discoloration)	Normal	0
	Minor irritation around surgical wound	1
	Moderate irritation/infected surgical wound	3
	Infected/open surgical wound	4 *
	Tumour > 1,0 cm in diameter	10
	Tumour > 1,5 cm in diameter	18
	Ulcerous tumour	18
Total:		
Comments, treatments etc:		

Dates: 2.4.19, 3.4.19, 5.4, 11.4, 24.4., 30.4, 7/5/19, 15/5, 21/5, 28/5, 4/6/19, 11/6-19, 14/6, 18/6, 24/6, 02/7, 03/7, 5/7, 8/7, 12/7

Body weight: 19.1, 19.6, 20.3, 20.44, 21.4, 20.7, 22.1, 21.4, 21.5, 20.4, 21.3, 20.6, 21.4, 21.6, 20.3, 20.9

Died

OMEGA 3 BEH start 04.7.19

0-3: Normal
4-9: Pay attention to health state and consider treatment
10-13: Pay close attention to health state, consider treatment or euthanasia
14-18: Euthanasia *Start treatment with antibiotics. If no effect within 3 days, euthanize the animal

Supplementary figure 1-17-4. Mouse and xenograft growth curves

Weight curve

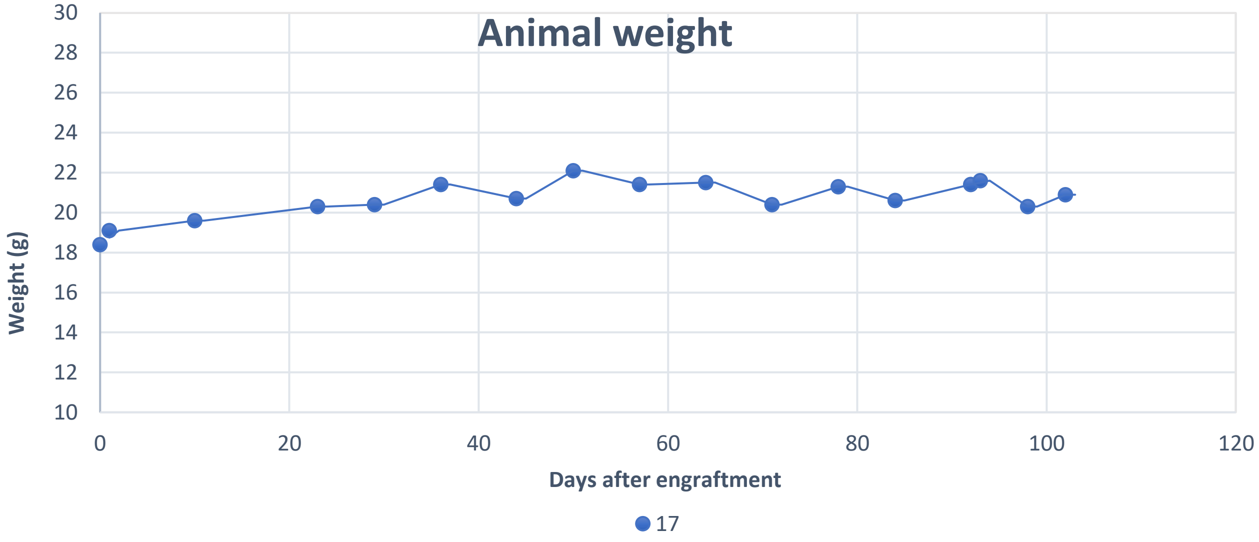

Xenograft growth curve

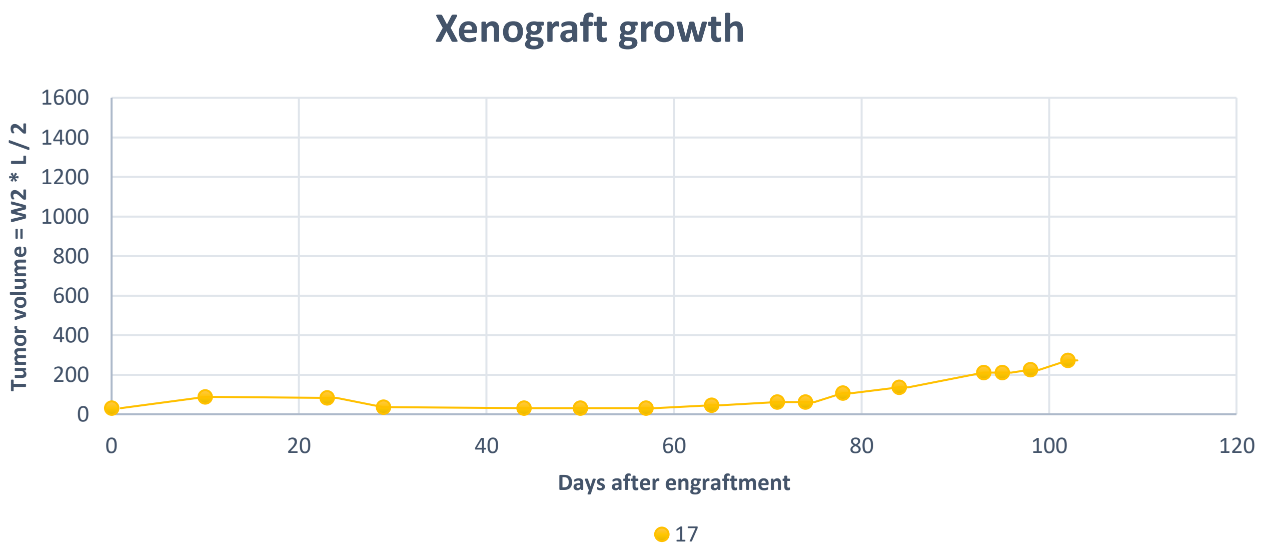

Mouse 18

- Surgery date: 03.09.19
    - Age (weeks) 33
    - Weight (g) 22.1
    - Tumor fragment P19-2b
    - Fragment size 3 x 3 mm
    - Ear marks 4

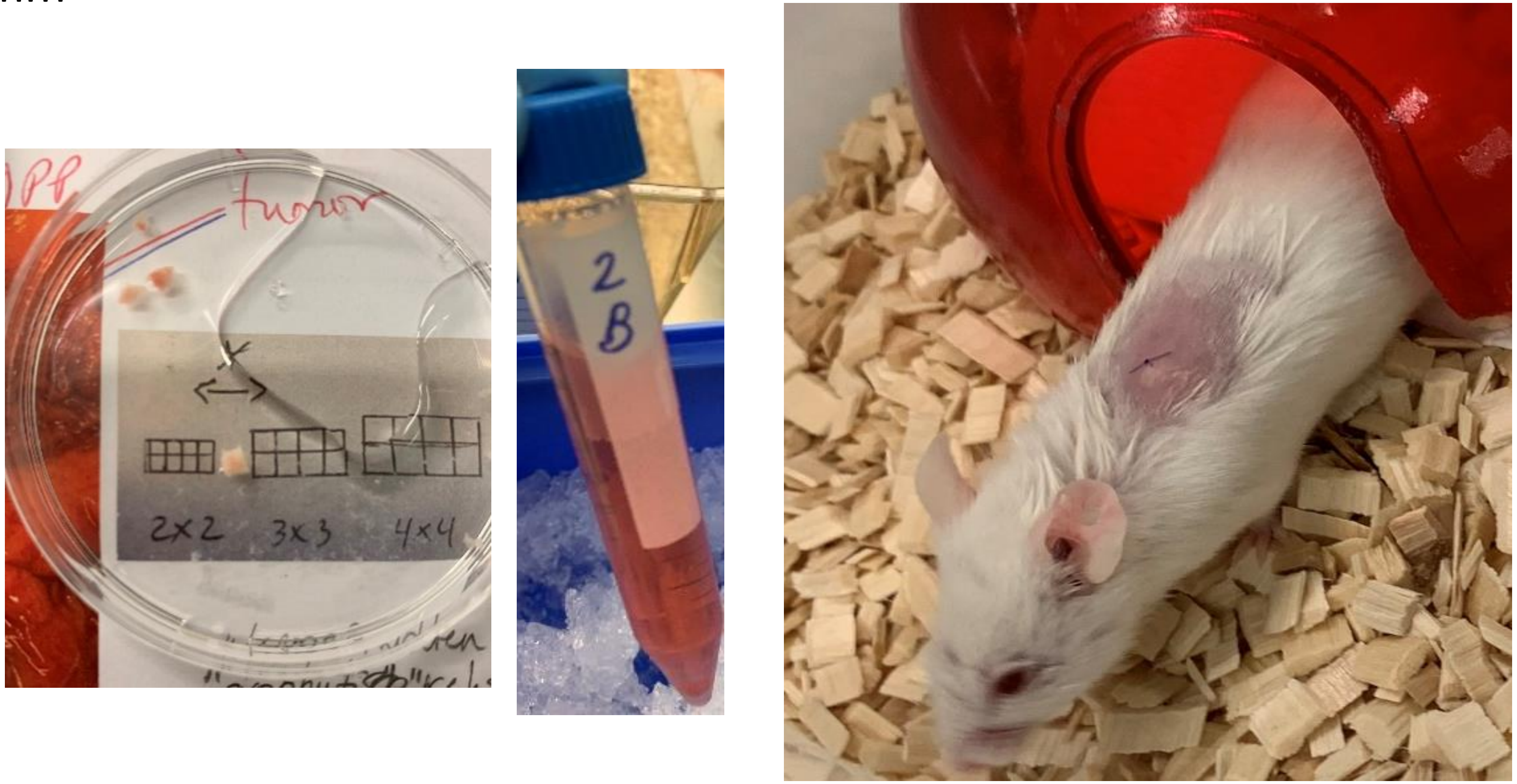

Supplementary figure 1-18-1.
Pictures from the engraftment day

Supplementary figure 1-18-2. Pictures from follow up and tumor size measurement

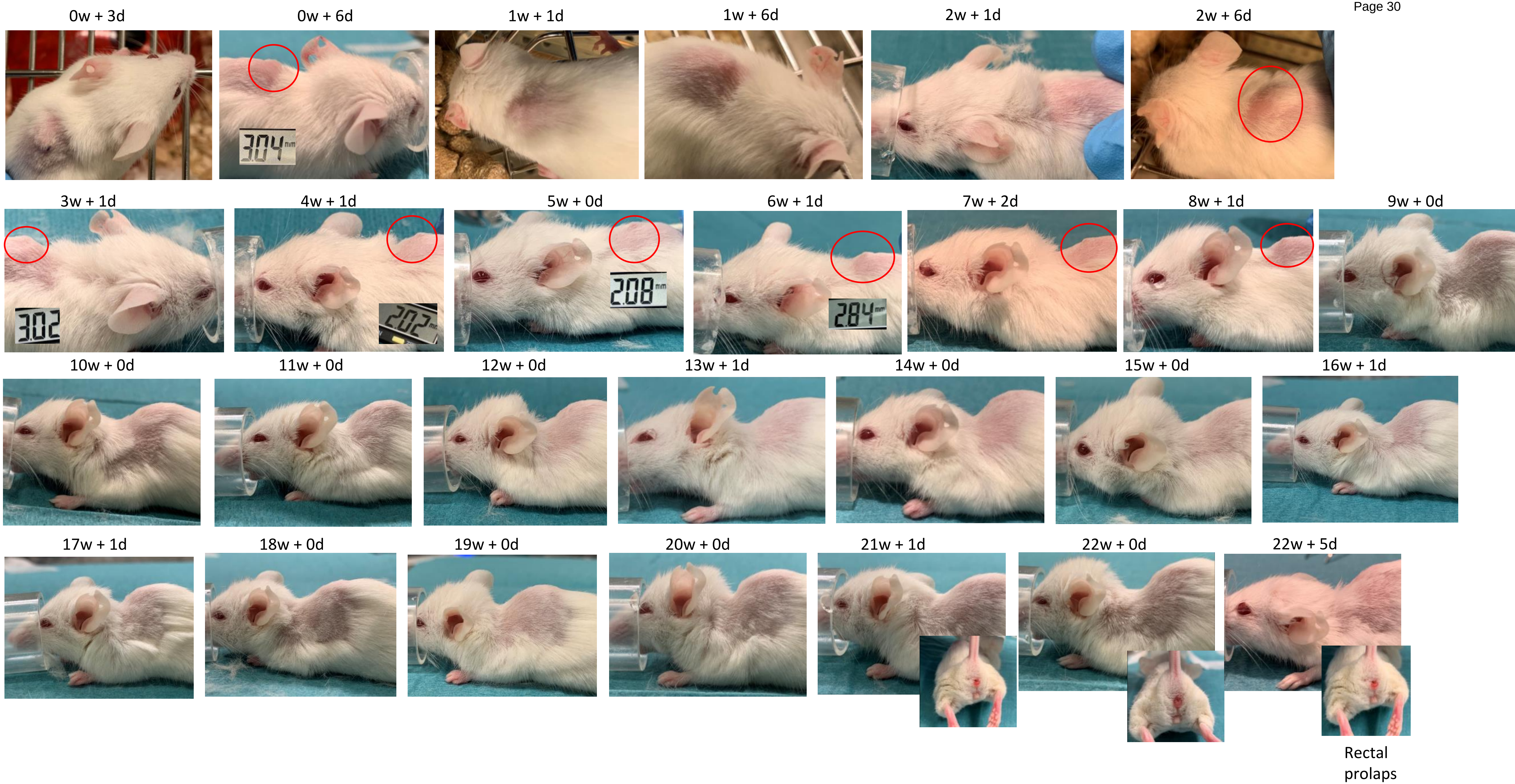

Supplementary figure 1-18-3. Pictures euthanization (10.02.20)

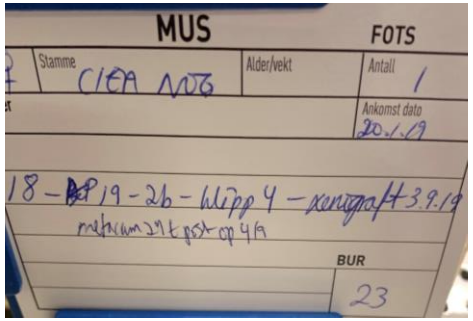

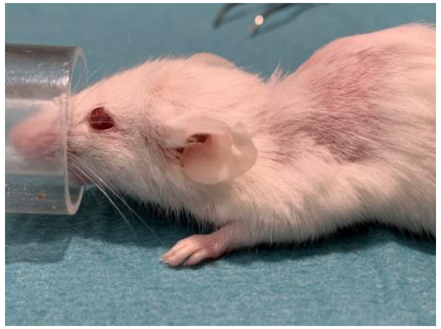

Reduced health, Rectal prolaps; svollen bloody anal opening
Weight sligtly reduced.

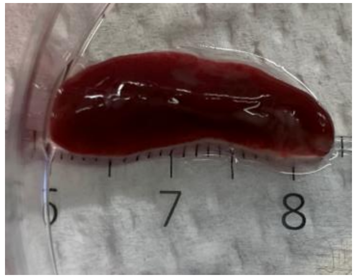

Large spleen, ca 2,5 cm, normal colour

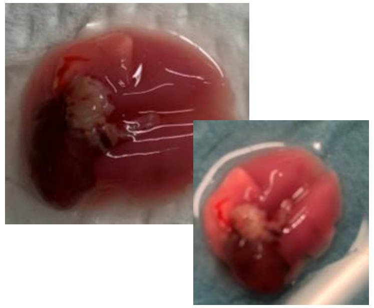

Whitish part of one lung

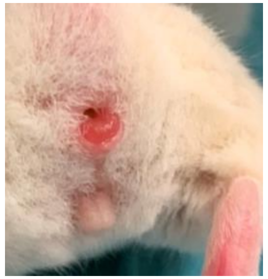

Red, svollen anal opening

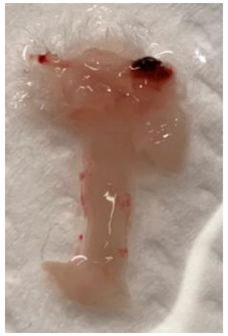

Bloody anal opening of rectum

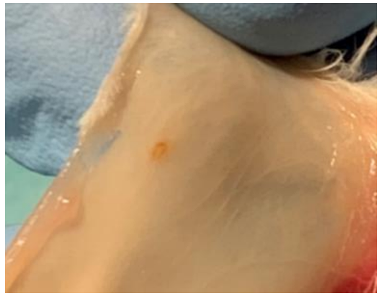

Only a small spot left of the xenograft

Before euthanization:

- Reduced health
- Rectal prolaps; red, svollen anus opening
- Reduced Weight 19,8 g
- Not growing xenograft (xenograft almost not visible)
- No treatment given

After euthanization:

- Enlarged spleen 2,5 cm
- Whitish part of one lung
- Xenograft did not seem to have grown

Histology samples:

- **M32ET** Rectum
- **M32N** Kidneys
- **M32MP** Milt/pancreas
- **M32LH** Lung and Heart
- **M32L** Liver

Supplementary figure 1-18-4. Pictures of score sheets

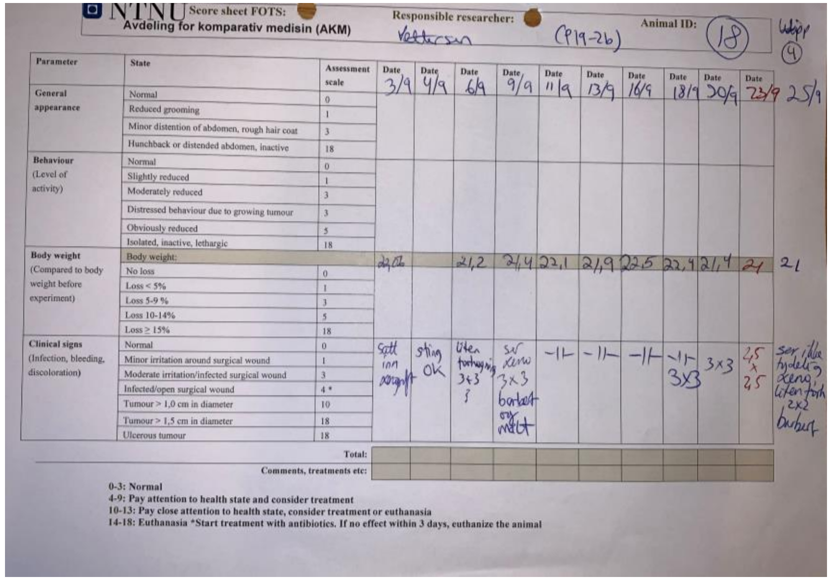

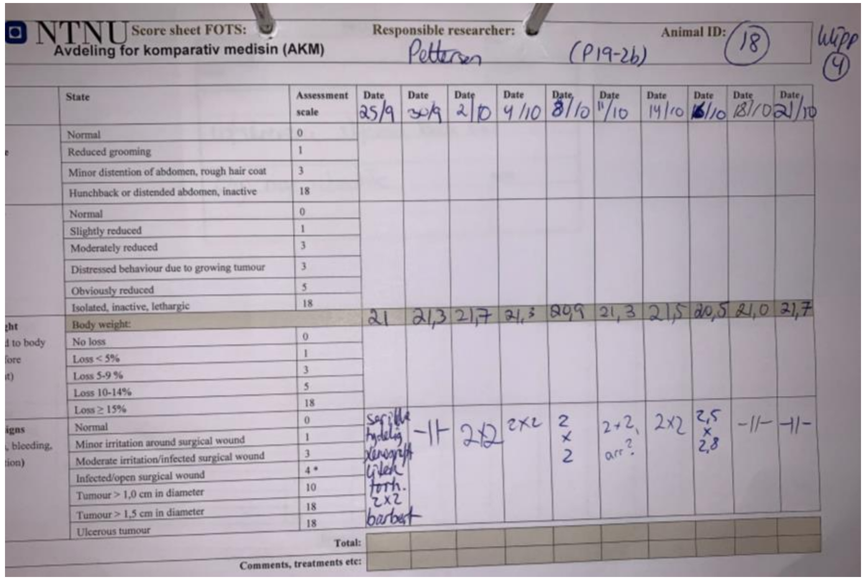

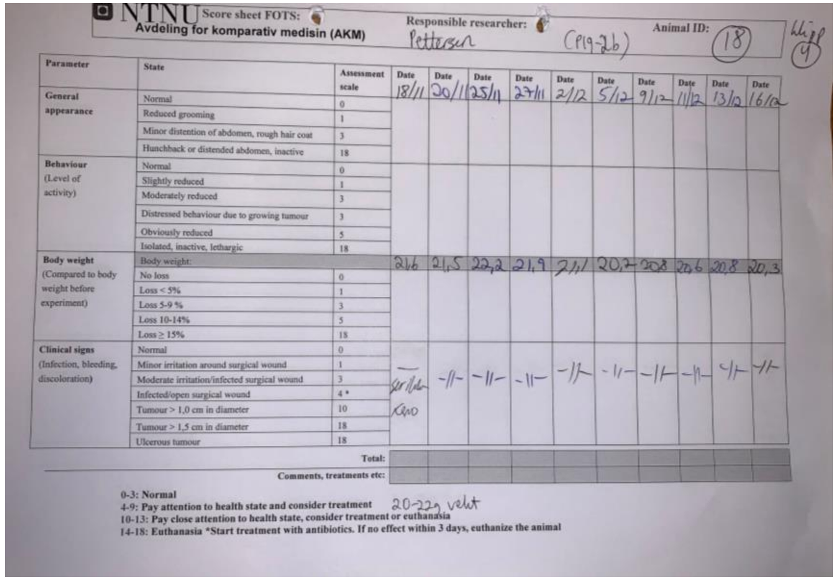

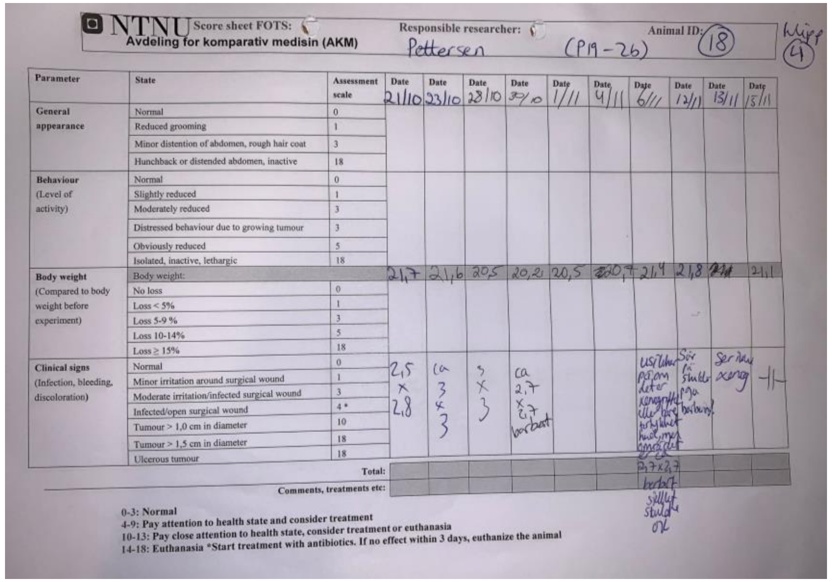

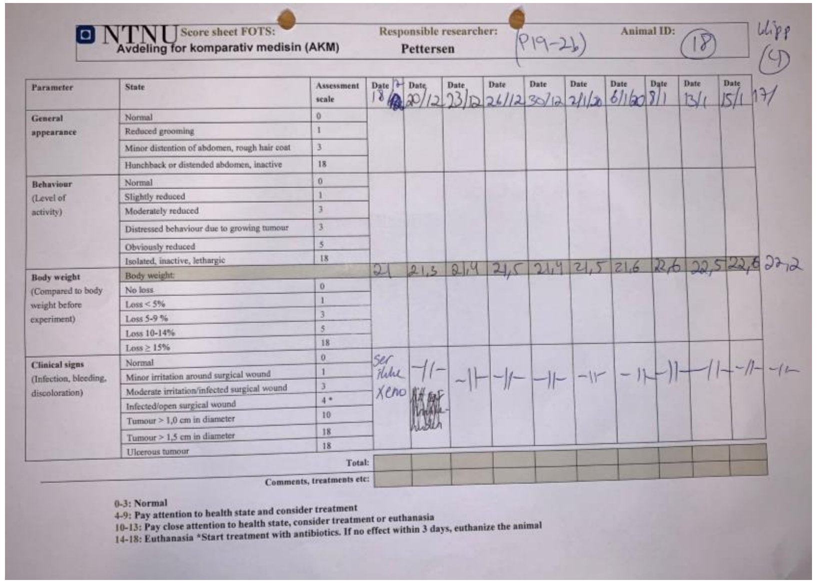

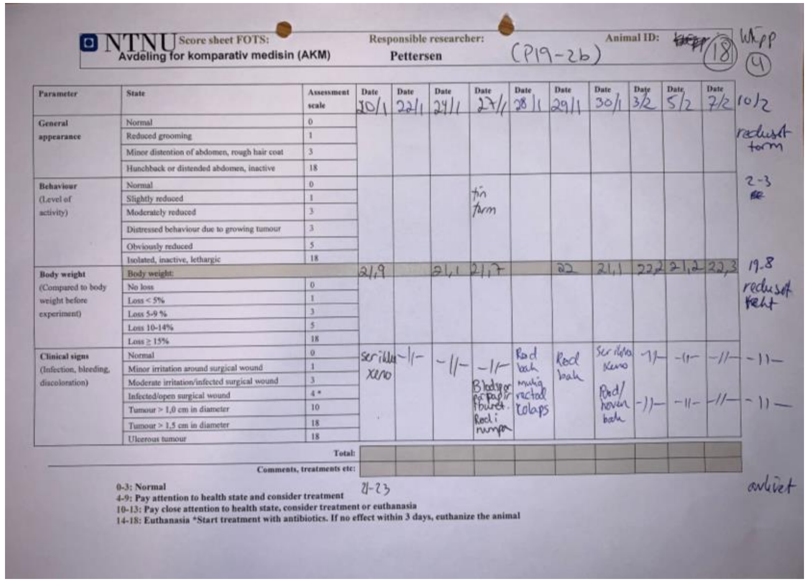

Supplementary figure 1-18-5. Mouse and xenograft growth curves

Weight curve

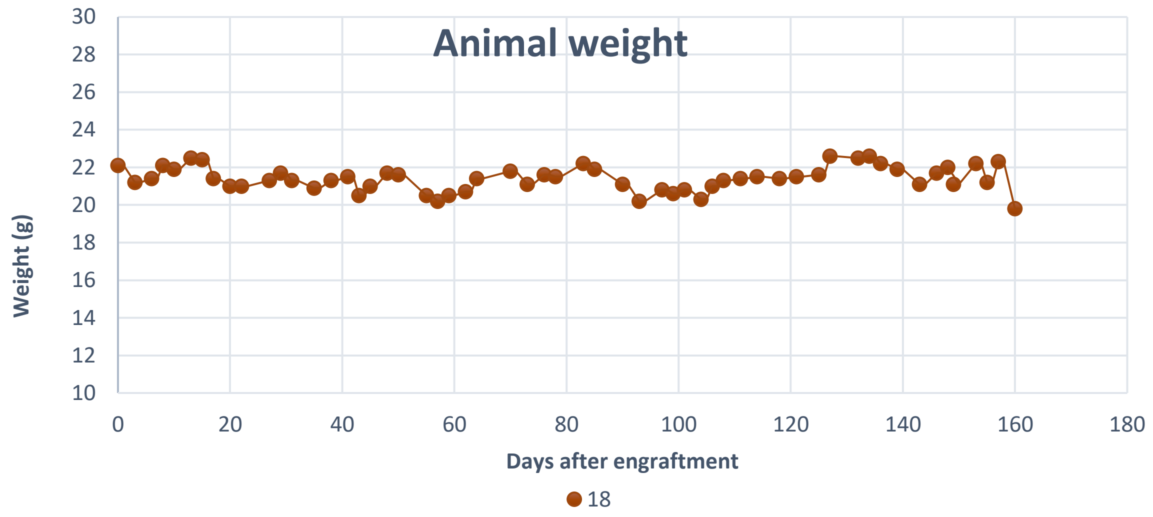

Xenograft growth curve

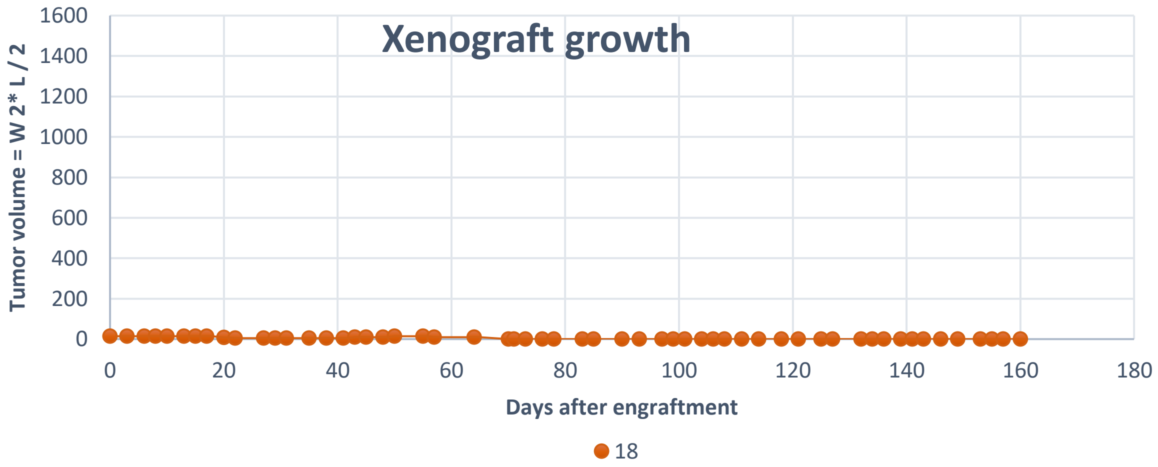

Mouse 19

- Surgery date: 14.05.19
    - Age (weeks) 17
    - Weight (g) 20.6
    - Tumor fragment P11-1A
    - Fragment size 4*4 mm
    - Ear marks 1

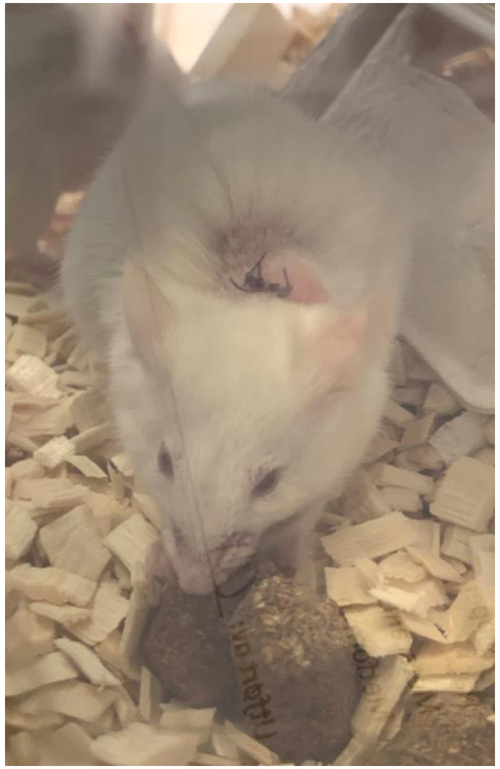

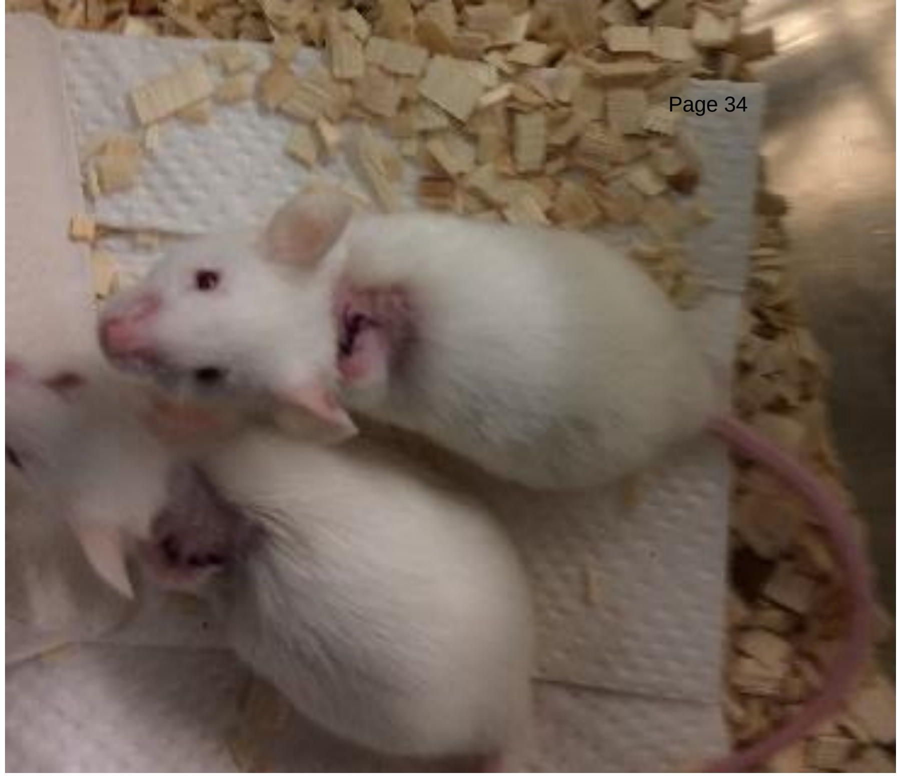

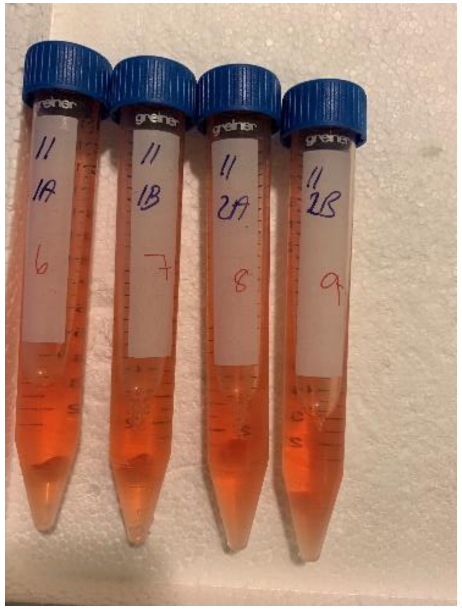

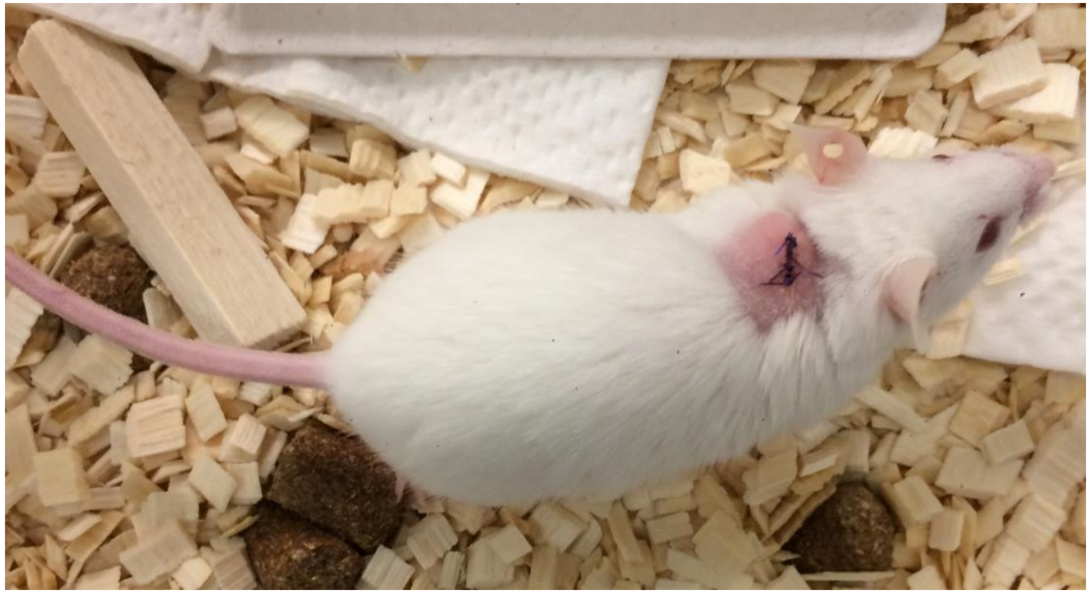

Supplementary figure 1-19-1. Pictures from the engraftment day

Supplementary figure 1-19-2. Pictures from follow up

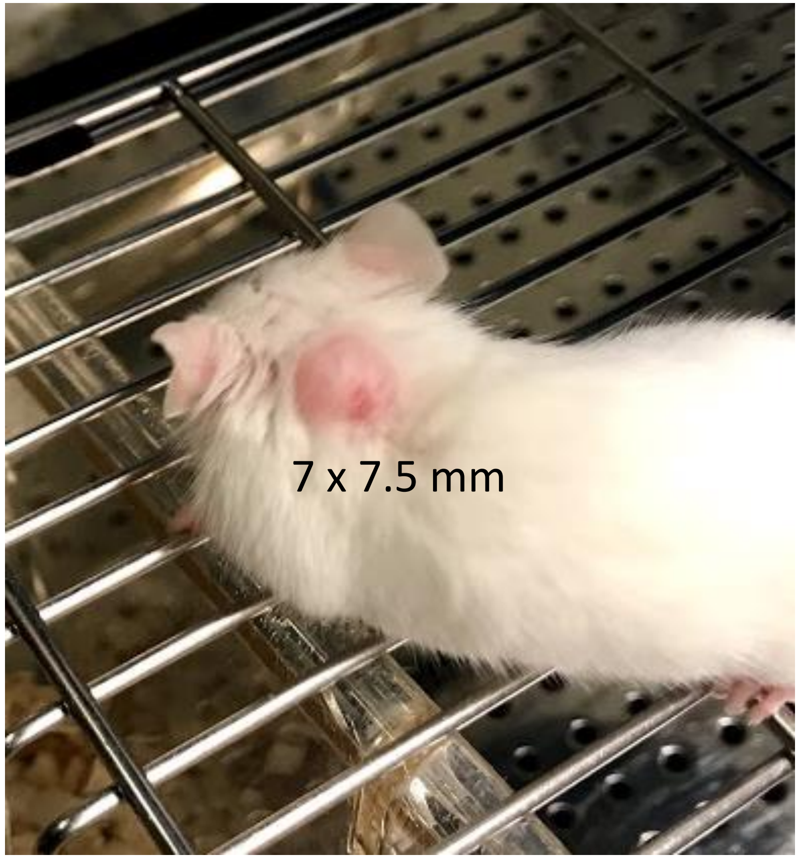

18.07.2019

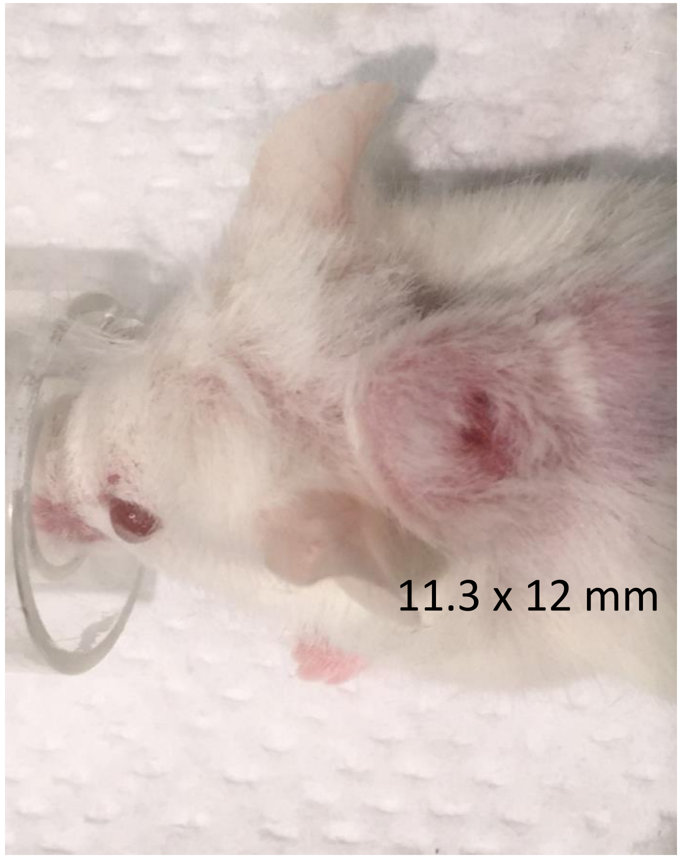

30.07.2019

Supplementary figure 1-19-3. List over weight registrations of the drinking bottle (with Smartfish Remune Peach) before and after provided to the mouse

- Smartfish with fishoil (OMEGA-3)
- 5 days
- Blodsamples were taken before and after treatment

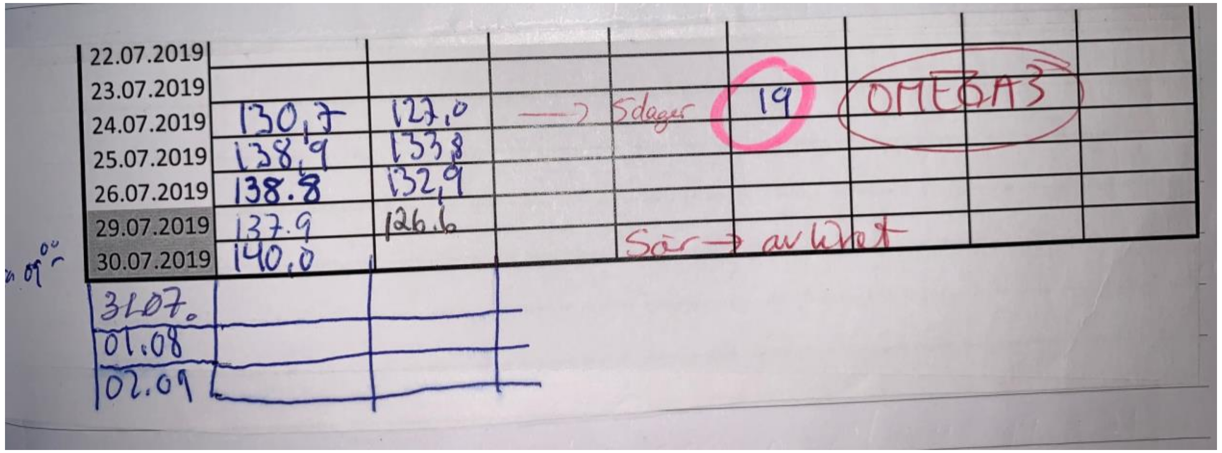

Supplementary figure 1-19-4. Pictures from euthanization (30.07.19)

- Xenograft size 11.3 x 12 mm
- Wound on skin over xenograft
- Blood sample (blood card and plasma)
- Tumor tissue for
    - Histology
    - RNA isolation
    - Protein isolation
- Note; stomach contains nutrition drink

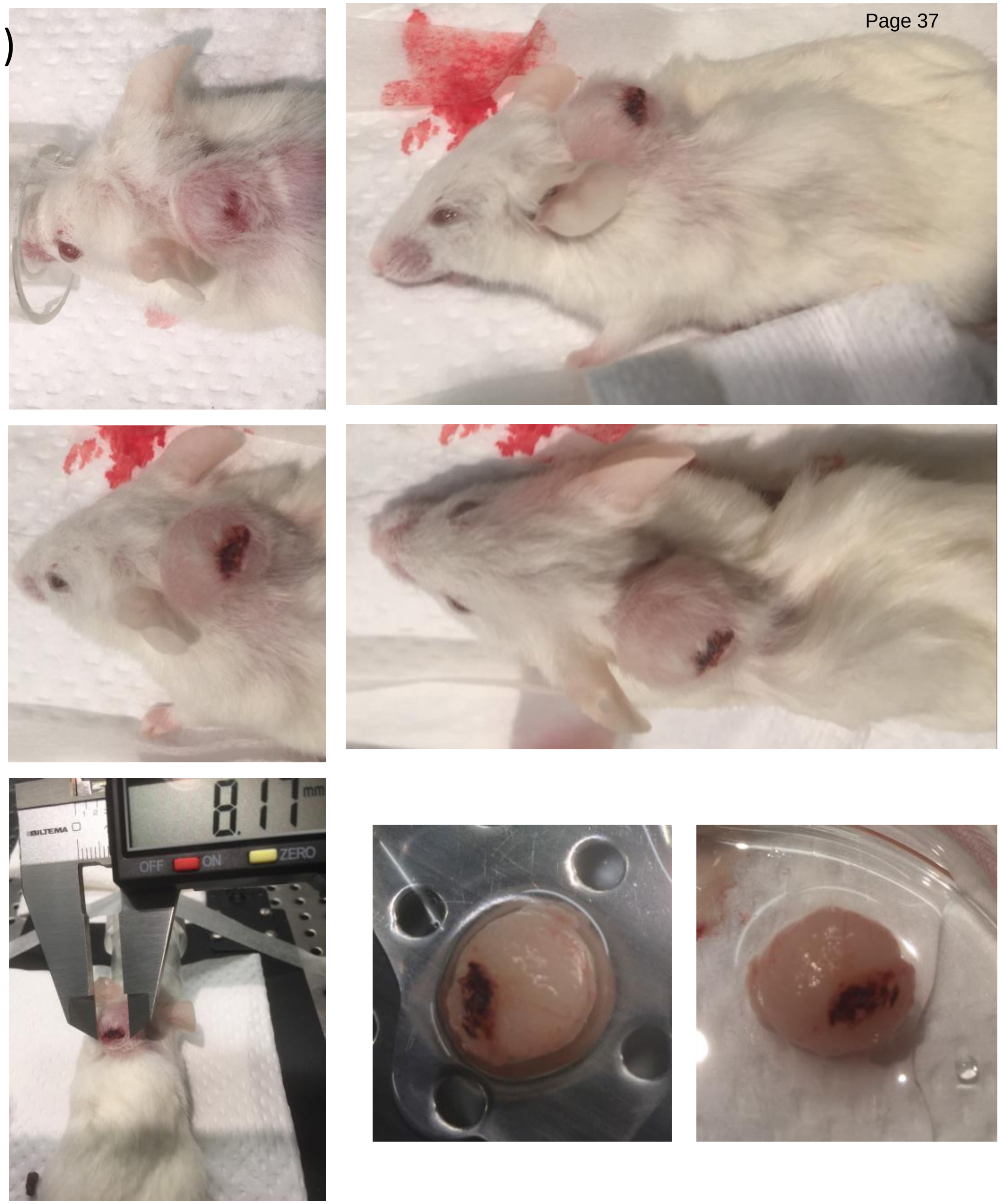

Supplementary figure 1-19-5. Pictures of score sheet

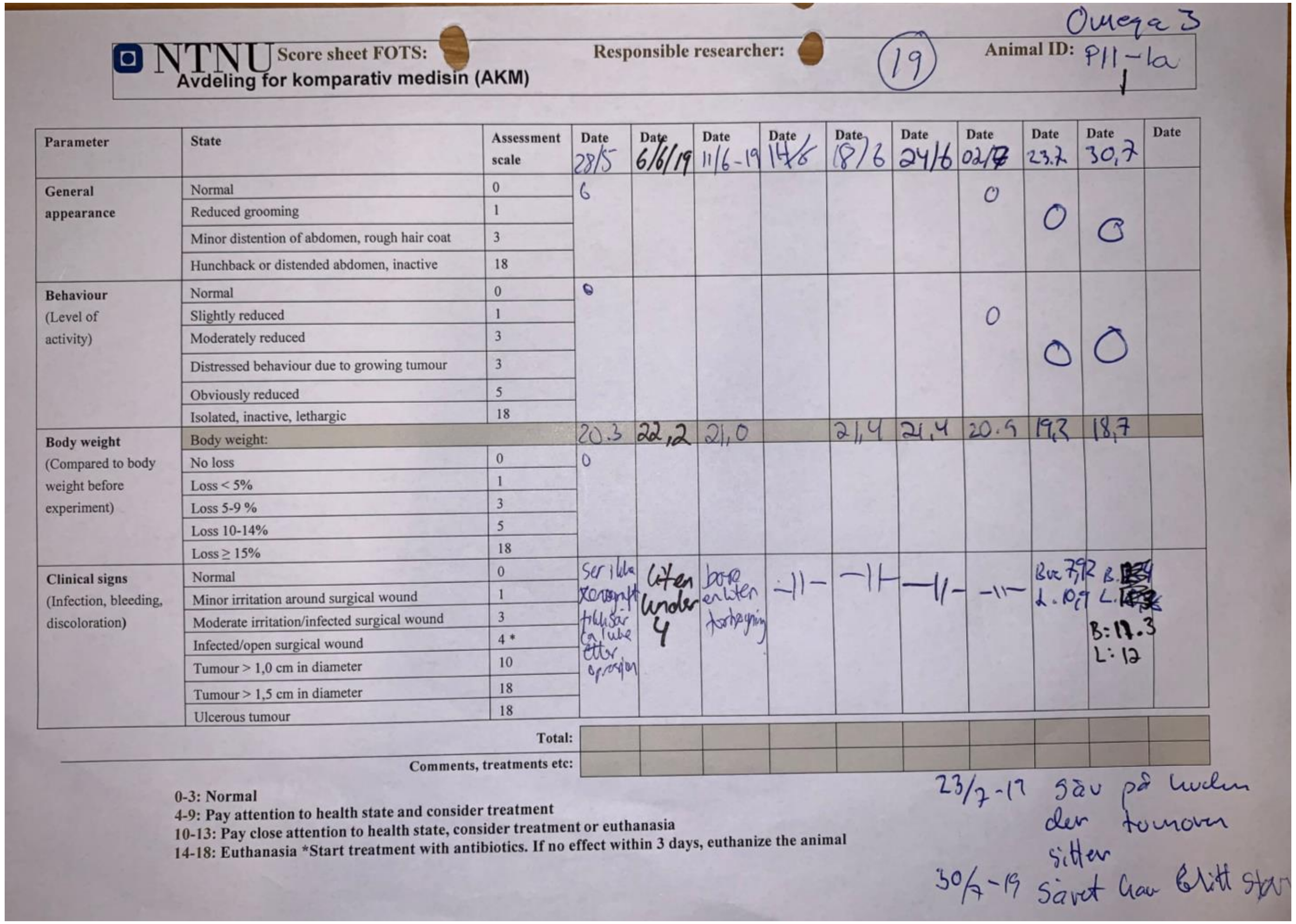

Omega 3

NTNU Score sheet FOTS: Responsible researcher: (19) Animal ID: P11-1a

Avdeling for komparativ medisin (AKM)

Parameter	State	Assessment scale	Date 28/5	Date 6/6/19	Date 11/6-19	Date 14/6	Date 18/6	Date 24/6	Date 02/7	Date 23.7	Date 30,7	Date
General appearance	Normal	0	6						0			
	Reduced grooming	1								0		
	Minor distention of abdomen, rough hair coat	3									0	
	Hunchback or distended abdomen, inactive	18										
Behaviour (Level of activity)	Normal	0	0									
	Slightly reduced	1							0			
	Moderately reduced	3									0	
	Distressed behaviour due to growing tumour	3								0		
	Obviously reduced	5										
	Isolated, inactive, lethargic	18										
Body weight (Compared to body weight before experiment)	Body weight:		20.3	22,2	21,0		21,4	21,4	20.9	19,3	18,7	
	No loss	0	0									
	Loss < 5%	1										
	Loss 5-9 %	3										
	Loss 10-14%	5										
	Loss ≥ 15%	18										
Clinical signs (Infection, bleeding, discoloration)	Normal	0	Ser ikke [illegible] til sår [illegible] etter operasjon	Liten under 4	bare en liten forhøyning	-11-	-11-	-11-	-11-	B: 7,92 L: 10,9	B. [illegible] L. [illegible] B: 17.3 L: 12	
	Minor irritation around surgical wound	1										
	Moderate irritation/infected surgical wound	3										
	Infected/open surgical wound	4 *										
	Tumour > 1,0 cm in diameter	10										
	Tumour > 1,5 cm in diameter	18										
	Ulcerous tumour	18										
		Total:										
		Comments, treatments etc:										

0-3: Normal
4-9: Pay attention to health state and consider treatment
10-13: Pay close attention to health state, consider treatment or euthanasia
14-18: Euthanasia *Start treatment with antibiotics. If no effect within 3 days, euthanize the animal

23/7-19 Sår på huden der tumoren sitter

30/7-19 Såret har blitt stør[illegible]

Supplementary figure 1-19-6. Mouse and xenograft growth curves

Weight curve

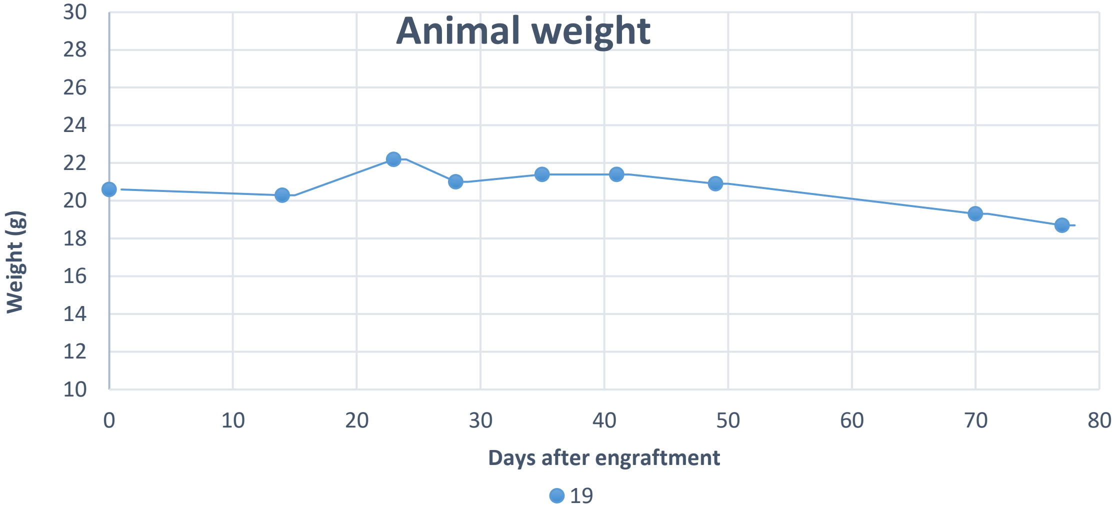

Xenograft growth curve

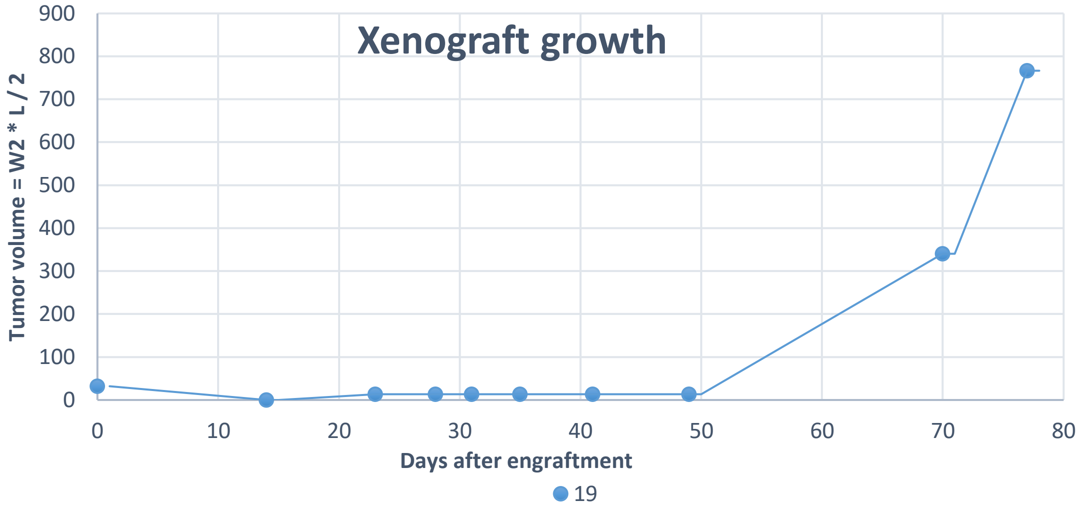

Supplementary figure 1-19-7. Histology pictures

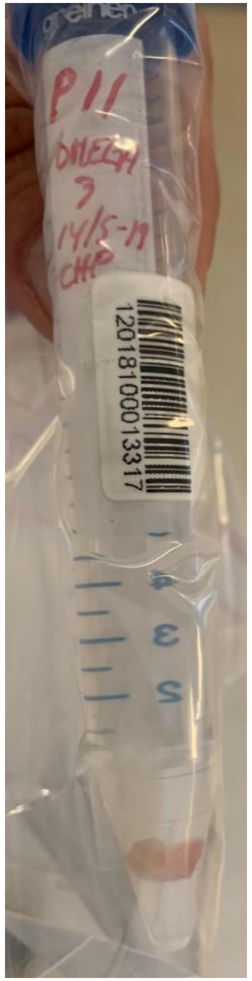

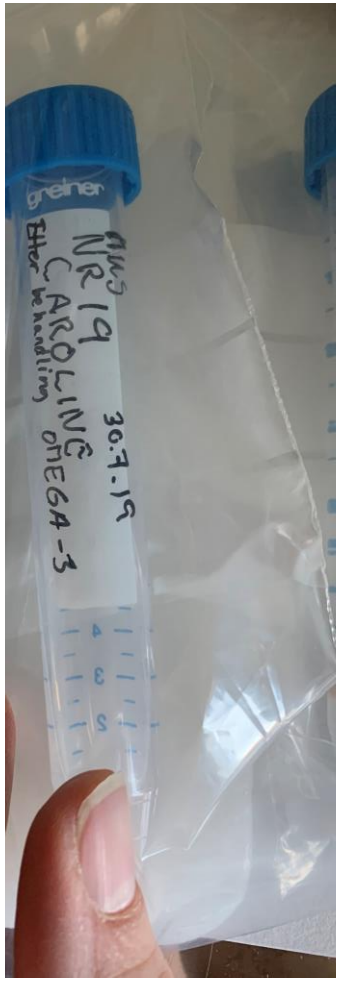

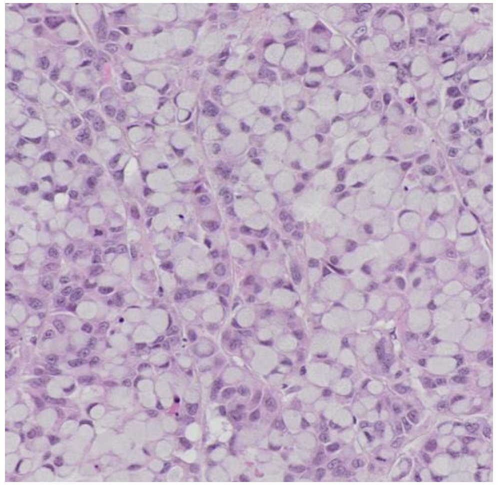

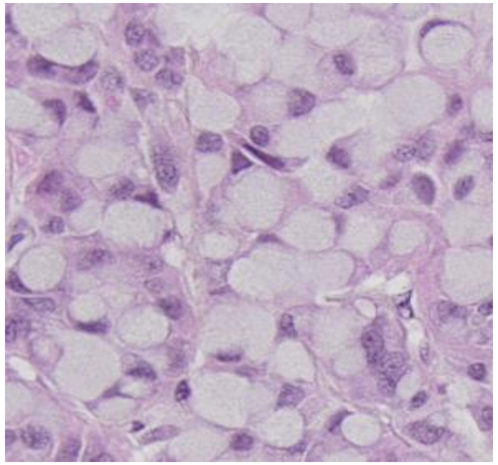

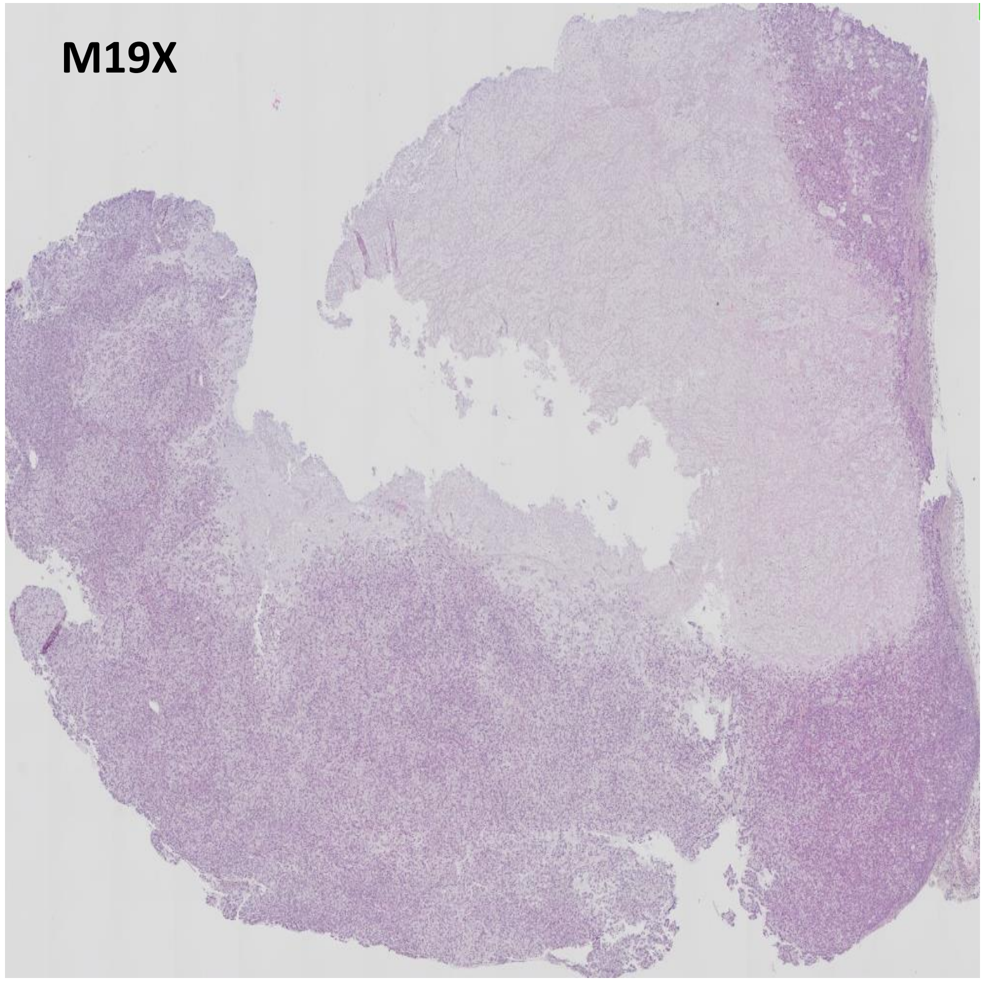

- Signet ring cells

- Signet ring cell adenocarcinoma
- Some necrosis

Mouse 20

- Surgery date: 14.5.19
  - Age (weeks) 17
  - Weight (g) 24.0
  - Tumor fragment P11-1B
  - Fragment size 4*4
  - Ear marks 2

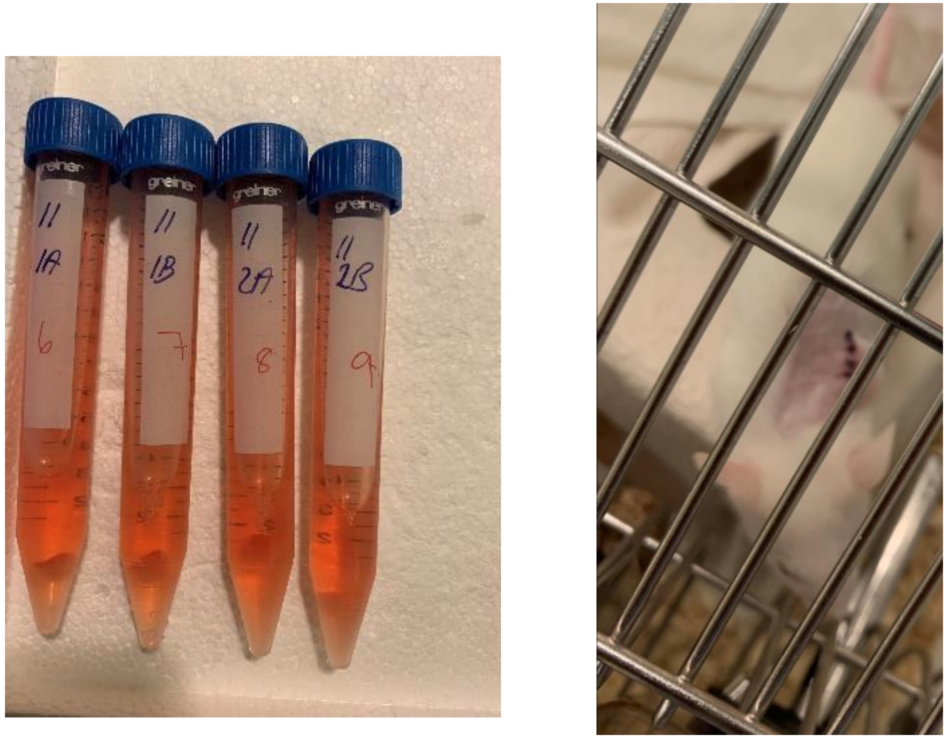

Supplementary figure 1-20-1. Pictures from the engraftment day

Supplementary figure 1-20-2. Pictures from follow up (14.06.19)

- the day before treatment start

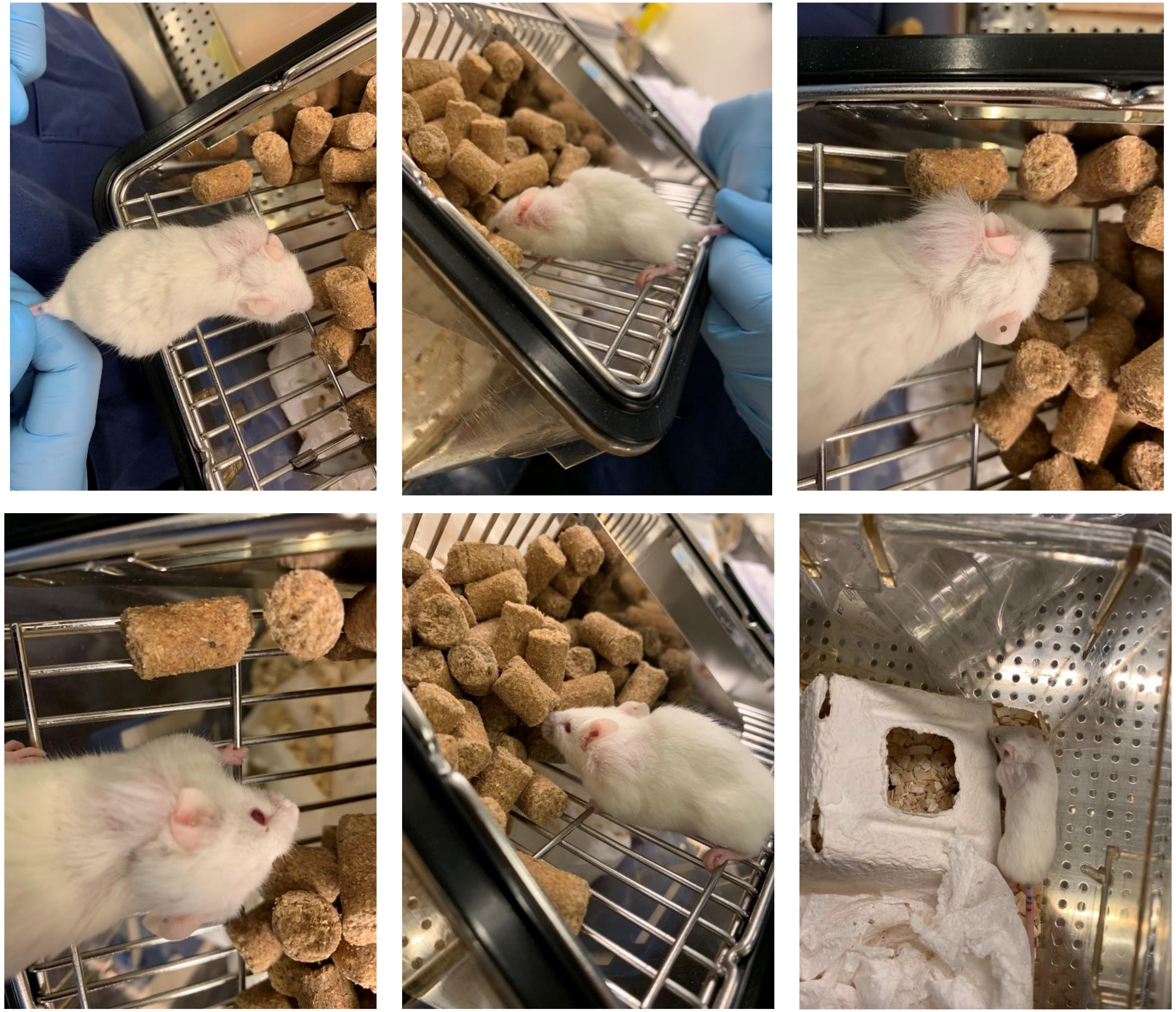

Blood sample and treatment

- Before: Vena Saphena11.06.19
  - Hard to get blood sample from
- After: Heart
- Treatment: Placebo

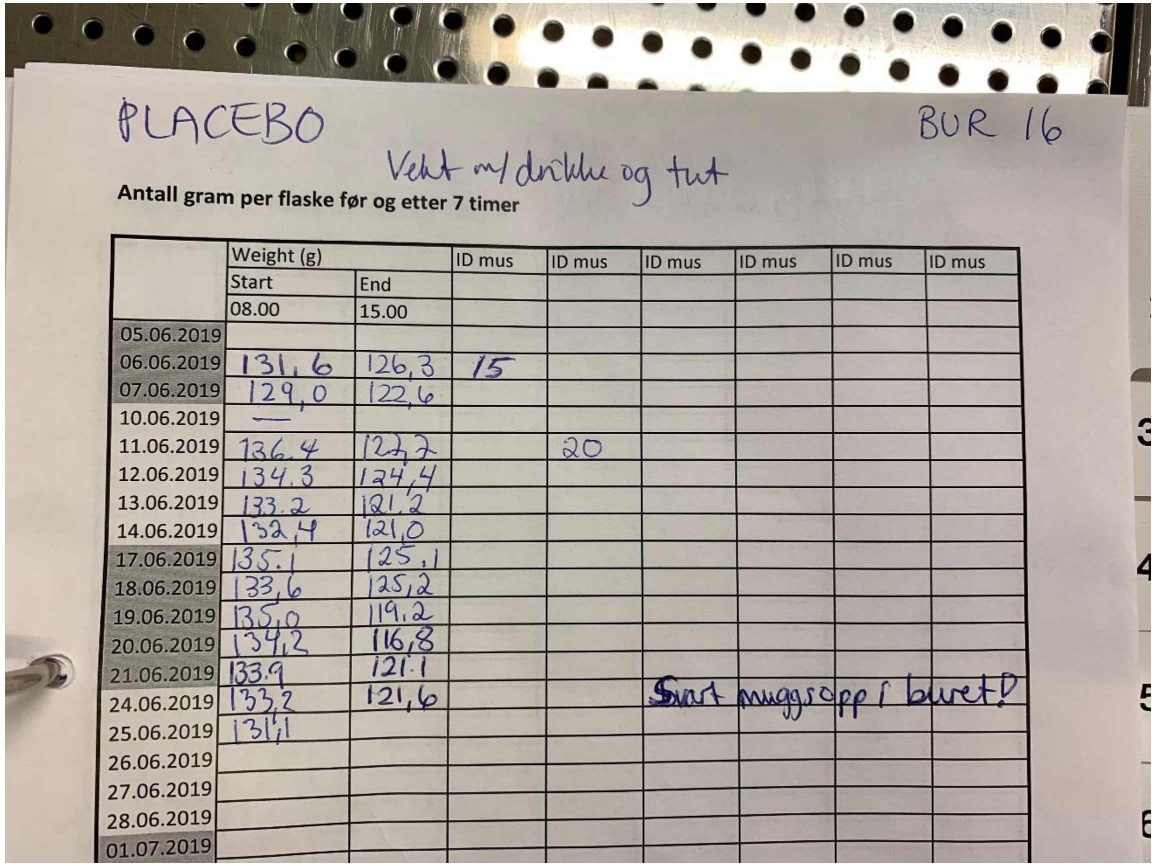

PLACEBO — BUR 16

Vekt m/ drikke og tut

Antall gram per flaske før og etter 7 timer

	Weight (g)		ID mus	ID mus	ID mus	ID mus	ID mus	ID mus
	Start	End						
	08.00	15.00						
05.06.2019								
06.06.2019	131,6	126,3	15					
07.06.2019	129,0	122,6						
10.06.2019	—							
11.06.2019	136.4	122,2		20				
12.06.2019	134.3	124,4						
13.06.2019	133.2	121.2						
14.06.2019	132,4	121,0						
17.06.2019	135.1	125,1						
18.06.2019	133,6	125,2						
19.06.2019	135,0	119.2						
20.06.2019	134,2	116,8						
21.06.2019	133.9	121.1						
24.06.2019	133,2	121,6			Svart muggsopp i buret!			
25.06.2019	131,1							
26.06.2019								
27.06.2019								
28.06.2019								
01.07.2019								

Supplementary figure 1-20-3. List over weight registrations of the drinking bottle (with Placebo) before and after provided to the mice

Supplementary figure 1-20-4. Pictures from euthanization

- Xenograft size 17 x 11.5
- Blood sample (blood card and plasma)
- Tumor tissue for
  - Histology
  - RNA isolation
  - Protein isolation

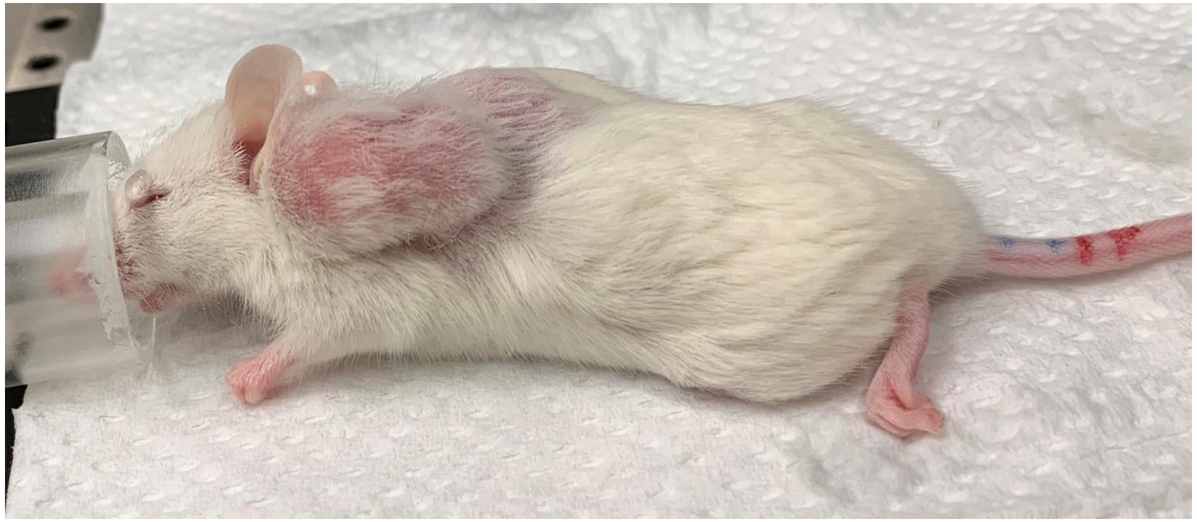

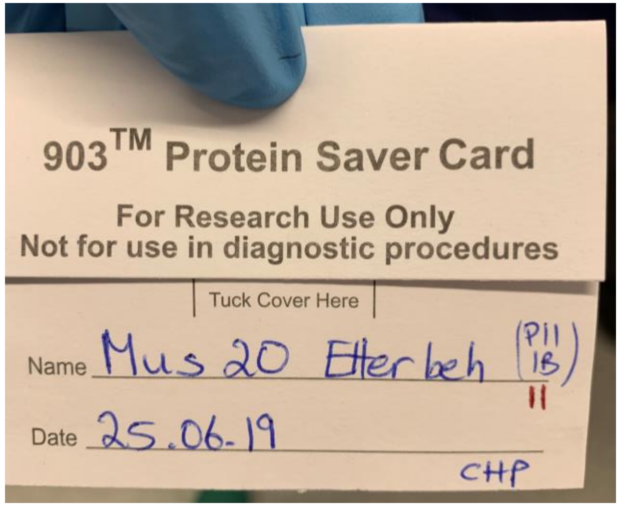

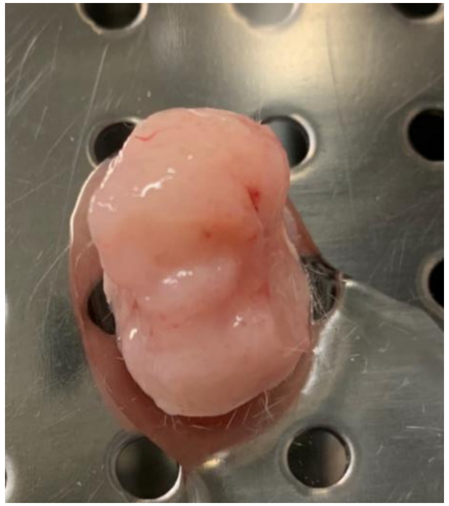

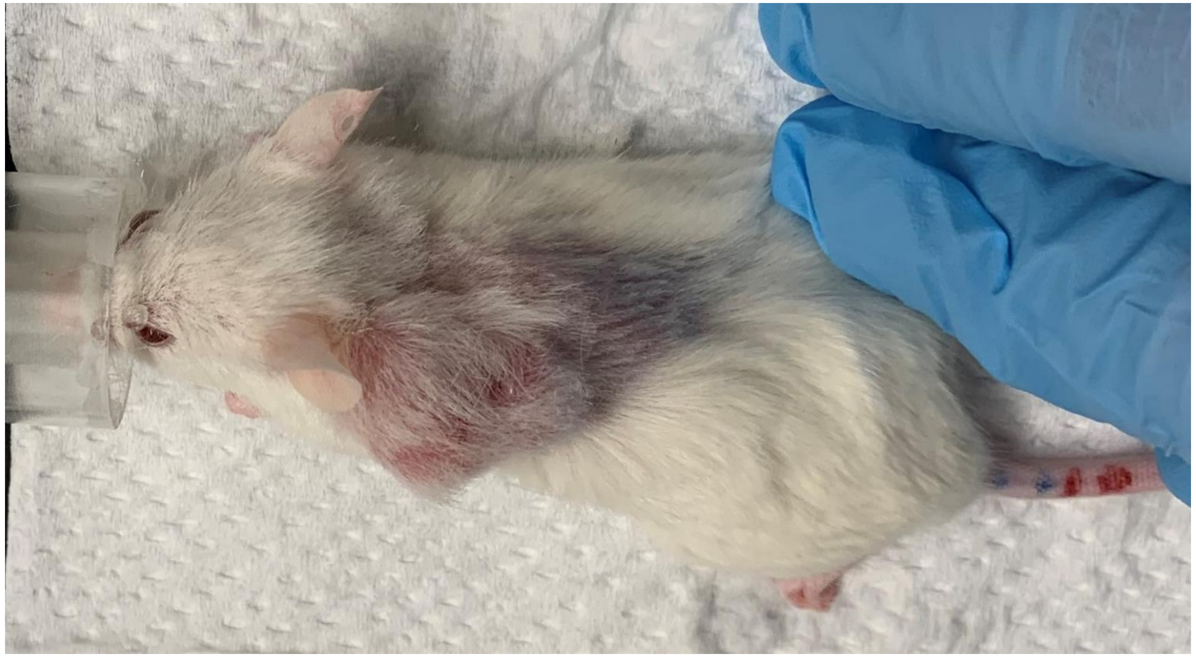

Supplementary figure 1-20-5. Picture of score sheet

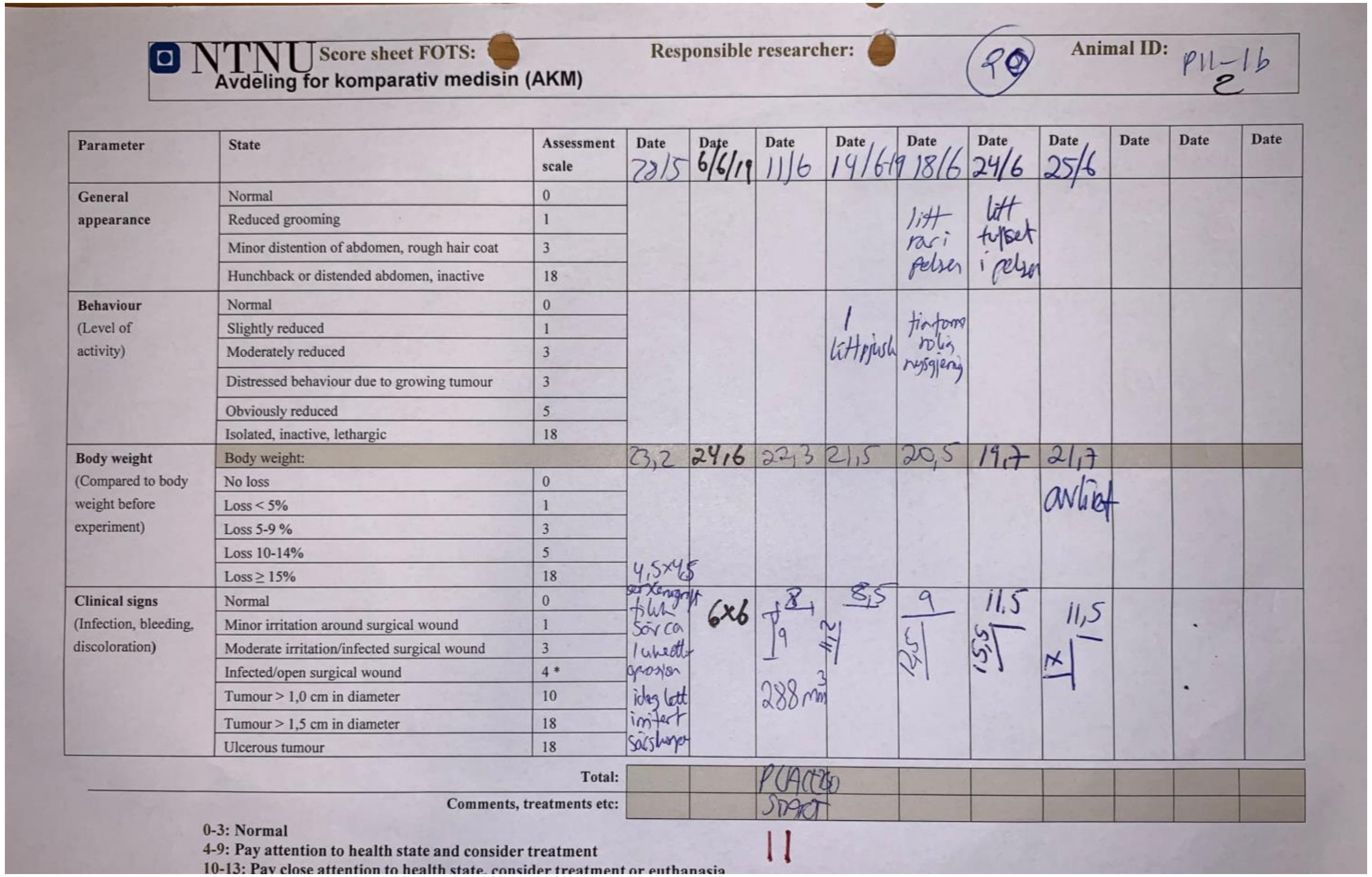

NTNU Score sheet FOTS: Responsible researcher: [illegible] Animal ID: P11-1b 2

Avdeling for komparativ medisin (AKM)

Parameter	State	Assessment scale	Date 28/5	Date 6/6/19	Date 11/6	Date 14/6-19	Date 18/6	Date 24/6	Date 25/6	Date	Date	Date
General appearance	Normal	0										
	Reduced grooming	1					litt rar i pelsen	litt tufset i pelsen				
	Minor distention of abdomen, rough hair coat	3										
	Hunchback or distended abdomen, inactive	18										
Behaviour (Level of activity)	Normal	0										
	Slightly reduced	1				1 litt pjusk	[illegible] rolig nysgjerrig					
	Moderately reduced	3										
	Distressed behaviour due to growing tumour	3										
	Obviously reduced	5										
	Isolated, inactive, lethargic	18										
Body weight (Compared to body weight before experiment)	Body weight:		23,2	24,6	22,3	21,5	20,5	19,7	21,7			
	No loss	0							avliv[illegible]			
	Loss < 5%	1										
	Loss 5-9 %	3										
	Loss 10-14%	5										
	Loss ≥ 15%	18	4,5x4,5									
Clinical signs (Infection, bleeding, discoloration)	Normal	0	[illegible]		8	8,5	9	11,5				
	Minor irritation around surgical wound	1	[illegible] sår ca	6x6	9	11,2			11,5			
	Moderate irritation/infected surgical wound	3	1 uke etter				12,5	15,5				
	Infected/open surgical wound	4 *	operasjon						17			
	Tumour > 1,0 cm in diameter	10	idag lett		288 mm³							
	Tumour > 1,5 cm in diameter	18	infisert									
	Ulcerous tumour	18	sårskorpe									
		Total:			[illegible]							
		Comments, treatments etc:			START							

11

0-3: Normal

4-9: Pay attention to health state and consider treatment

10-13: Pay close attention to health state, consider treatment or euthanasia

Supplementary figure 1-20-6. Mouse and xenograft growth curves

Weight curve

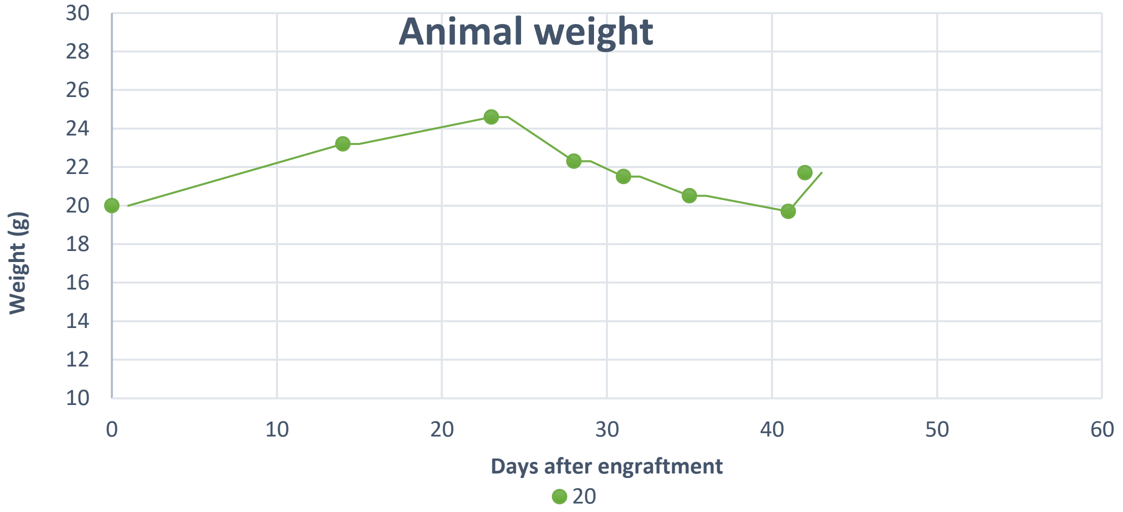

Xenograft growth curve

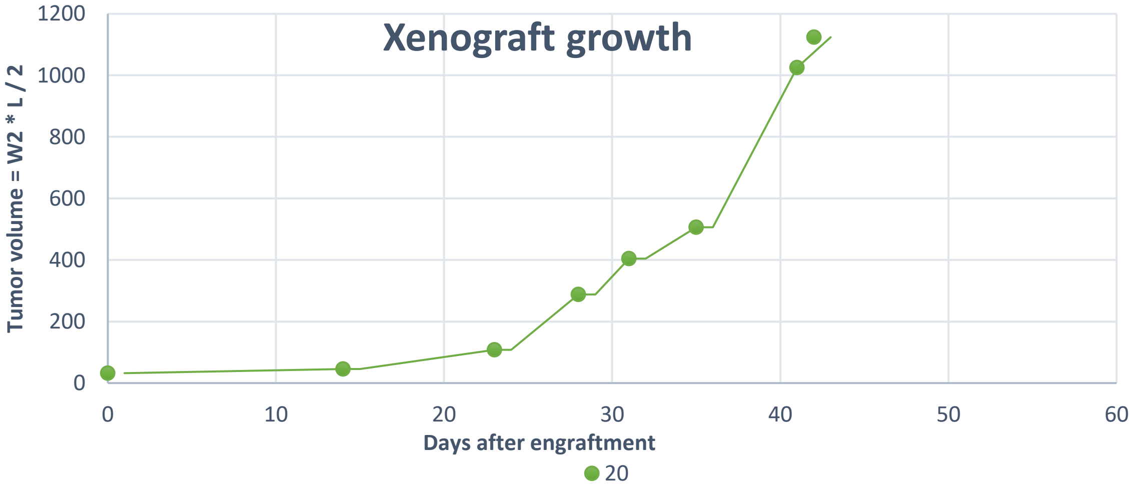

Supplementary figure 1-20-7.
Histology pictures

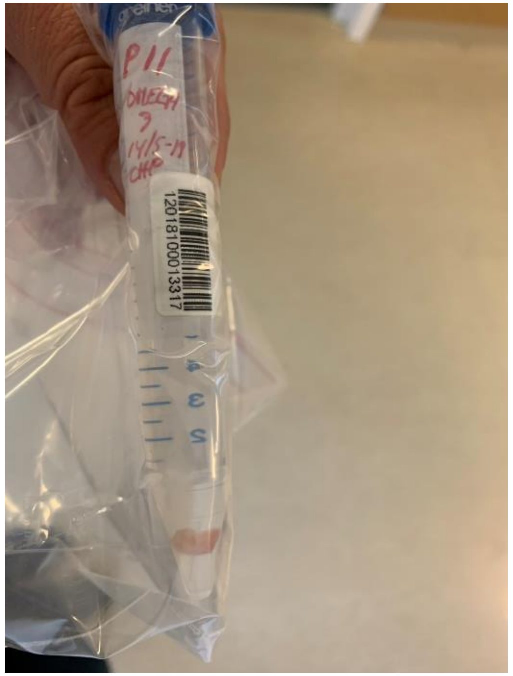

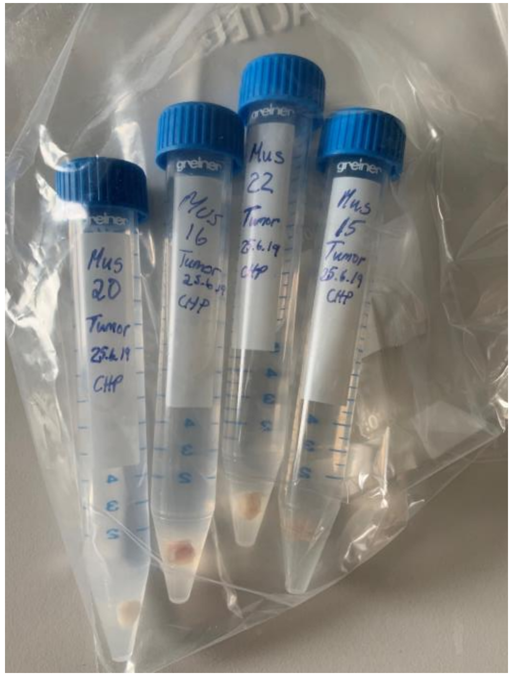

M20X

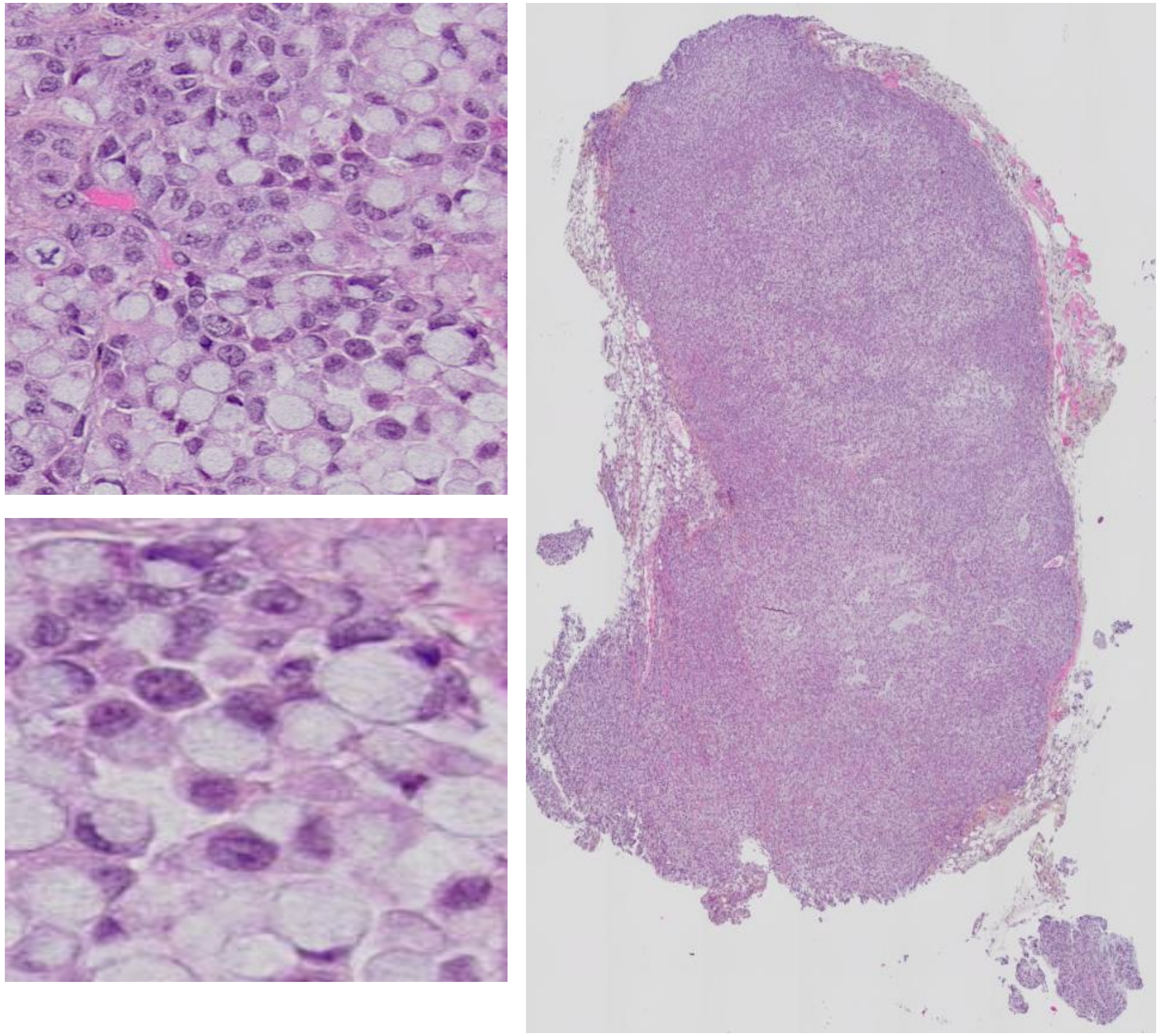

- Origin from signet ring cell adenocarcinoma
- Can see granulocytes in the blood vains getting attached to enter the tissue through the vain wall

Mouse 21

- Surgery date: 14.5.19
  - Age (weeks) 17
  - Weight (g) 20.3
  - Tumor fragment P11-2A
  - Fragment size 4*4
  - Ear marks 3

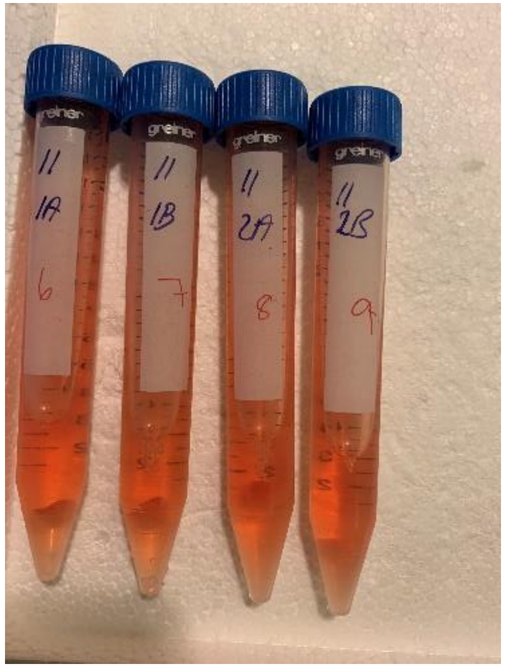

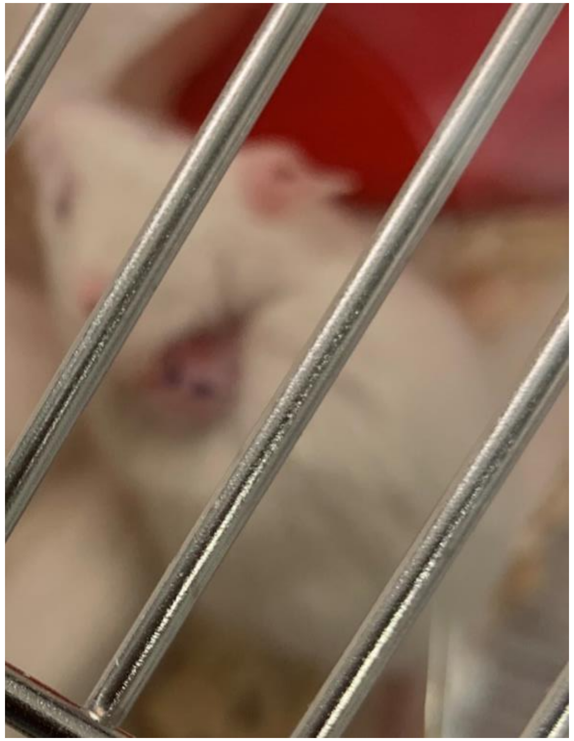

Supplementary figure 1-21-1. Pictures from the engraftment day

Supplementary figure 1-21-2. Pictures from euthanization (07.08.19)

- Xenograft size 7x9 mm
  - Not registered until 06.08.19
- Reduced health and weight
- Tumor tissue not sampled
- No treatment given
- Low blood volume; could not take blood sample

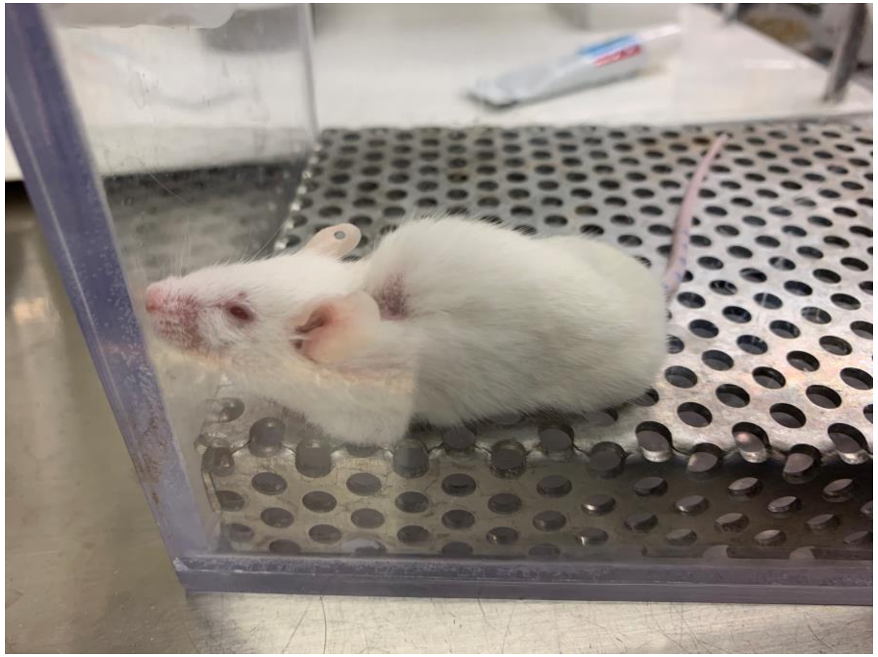

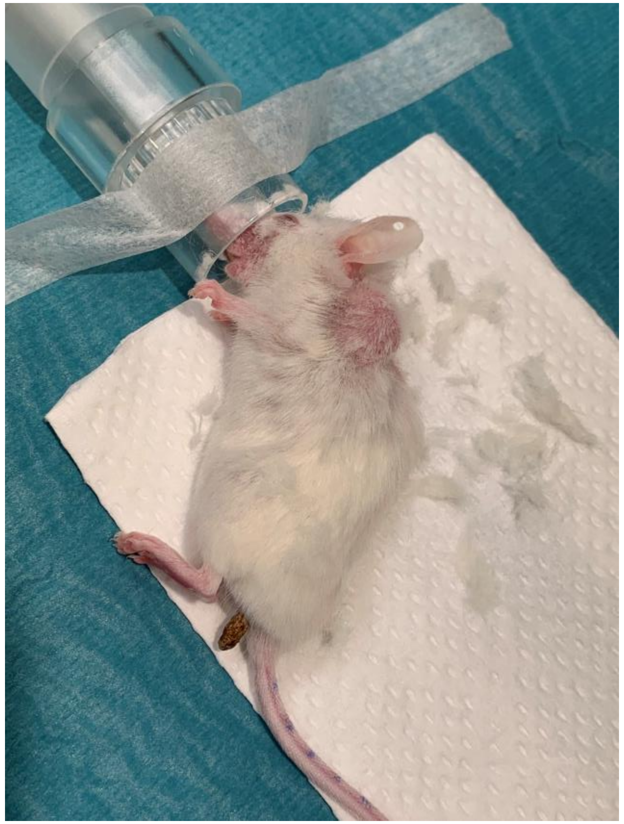

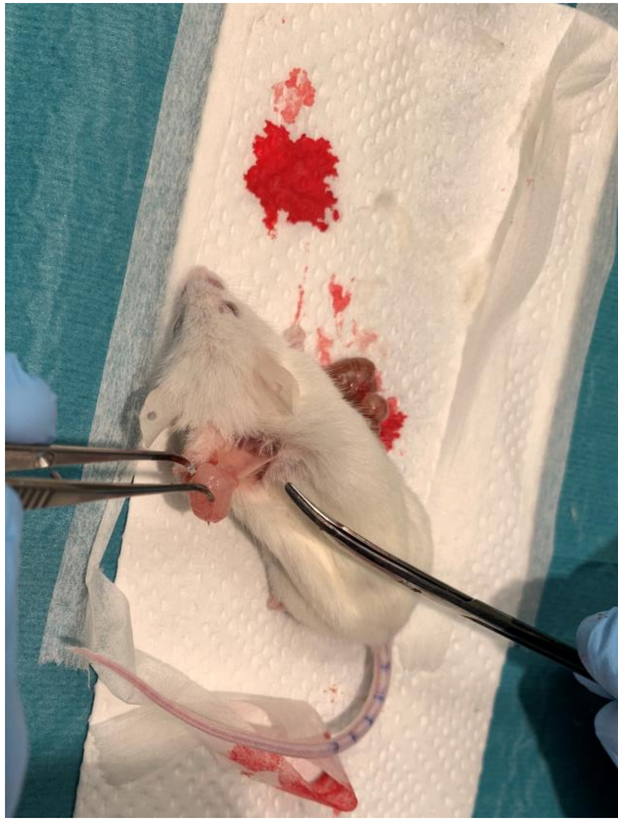

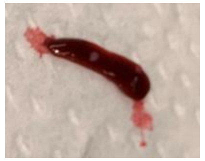

Normal spleen

Supplementary figure 1-21-3. Pictures of score sheets

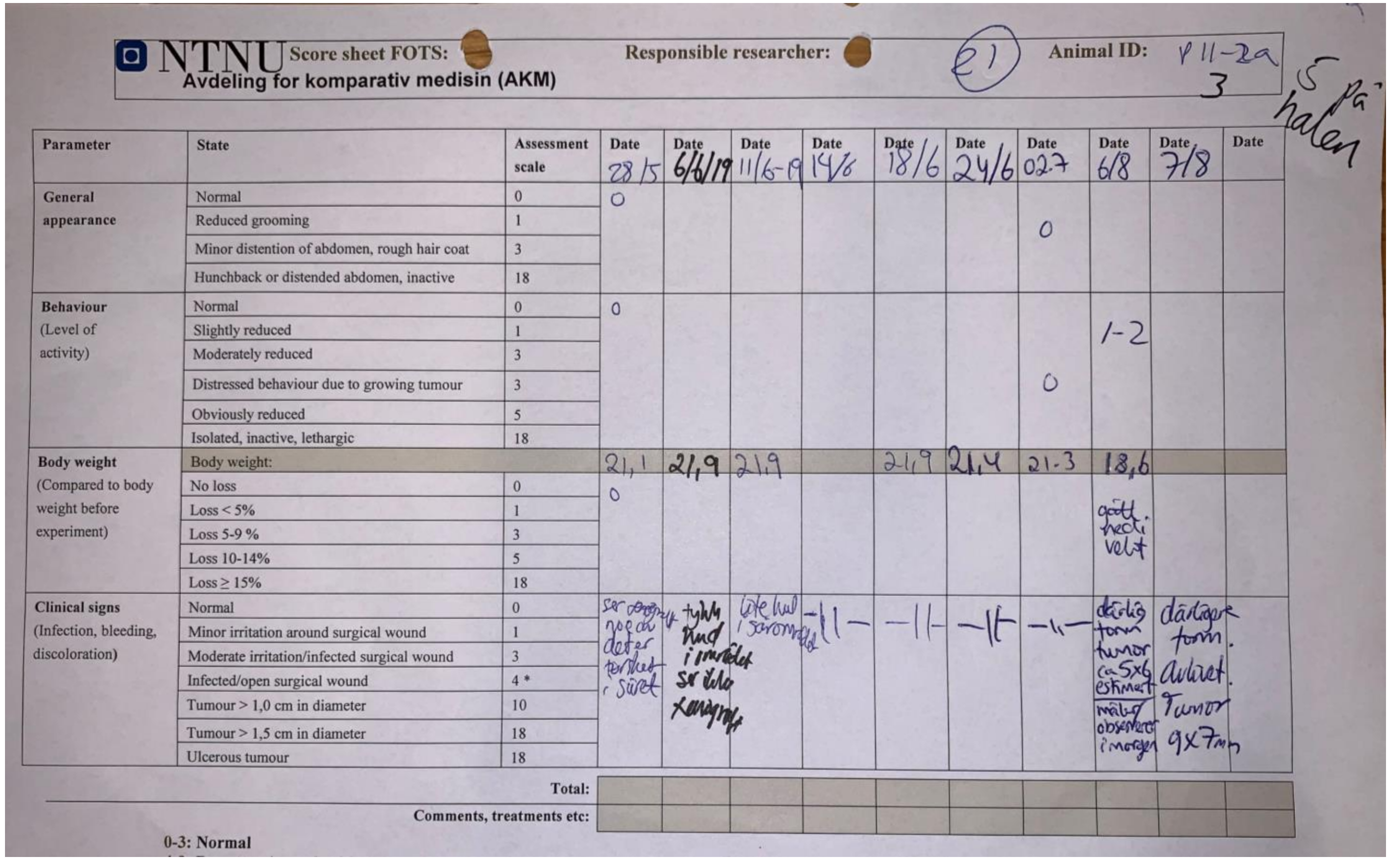

NTNU Score sheet FOTS: Responsible researcher: 21 Animal ID: ♀11-2a 3

Avdeling for komparativ medisin (AKM)

5 på halen

Parameter	State	Assessment scale	Date 28/5	Date 6/6/19	Date 11/6-19	Date 14/6	Date 18/6	Date 24/6	Date 02.7	Date 6/8	Date 7/8	Date
General appearance	Normal	0	0									
	Reduced grooming	1							0			
	Minor distention of abdomen, rough hair coat	3										
	Hunchback or distended abdomen, inactive	18										
Behaviour (Level of activity)	Normal	0	0									
	Slightly reduced	1								1-2		
	Moderately reduced	3										
	Distressed behaviour due to growing tumour	3							0			
	Obviously reduced	5										
	Isolated, inactive, lethargic	18										
Body weight (Compared to body weight before experiment)	Body weight:		21,1	21,9	21,9		21,9	21,4	21-3	18,6		
	No loss	0	0							[illegible]		
	Loss < 5%	1										
	Loss 5-9 %	3										
	Loss 10-14%	5										
	Loss ≥ 15%	18										
Clinical signs (Infection, bleeding, discoloration)	Normal	0	Sår [illegible] noe [illegible] der [illegible] i såret	tykk hud i området sår [illegible]	Lite hud i sårområde	-||-	-||-	-||-	-||-	dårlig form tumor ca 5x6 estimert / måling observeres i morgen	dårligere form. Avlivet. Tumor 9x7mm	
	Minor irritation around surgical wound	1										
	Moderate irritation/infected surgical wound	3										
	Infected/open surgical wound	4 *										
	Tumour > 1,0 cm in diameter	10										
	Tumour > 1,5 cm in diameter	18										
	Ulcerous tumour	18										
		Total:										
		Comments, treatments etc:										

0-3: Normal

Supplementary figure 1-21-4. Mouse and xenograft curve

Weight curve

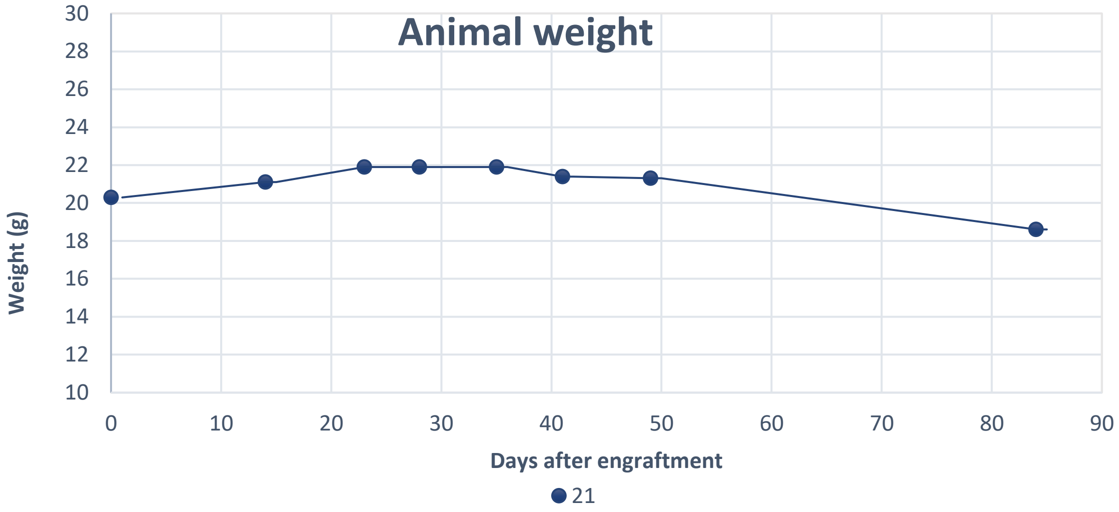

Xenograft growth curve

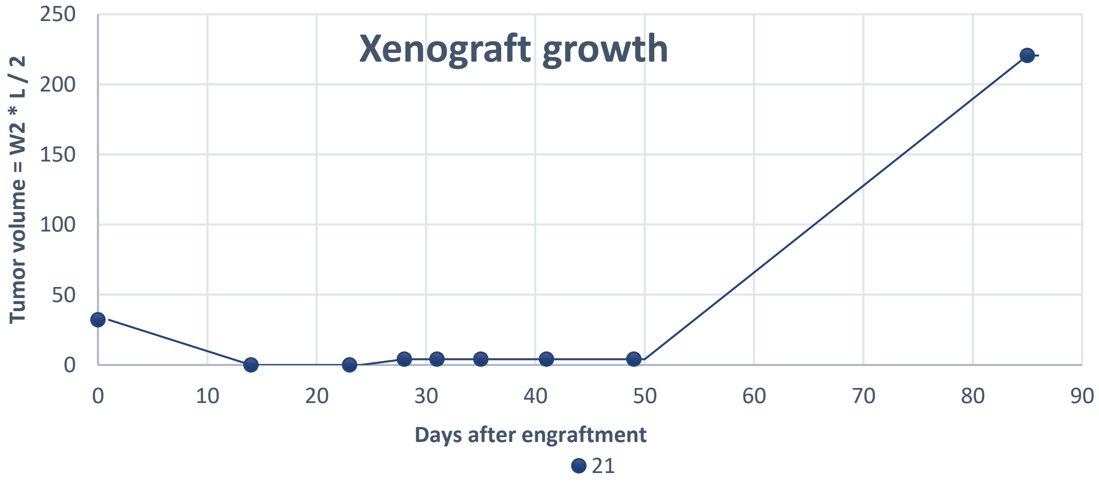

Mouse 22

- Surgery date: 14.5.19
    - Age (weeks) 17
    - Weight (g) 21.2
    - Tumor fragment P11-2B
    - Fragment size 4*4
    - Ear marks 4

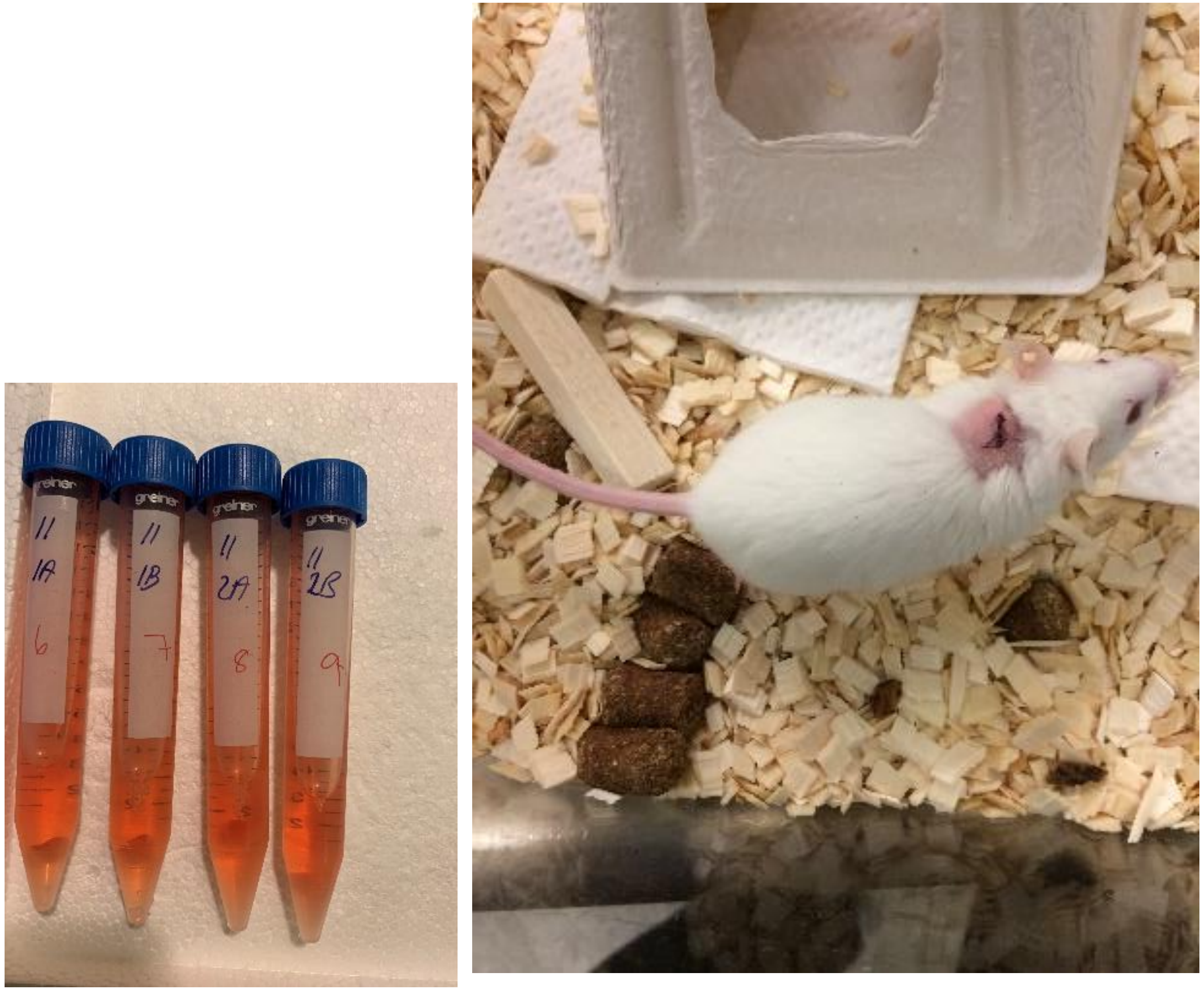

Supplementary figure 1-22-1. Pictures from the engraftment day

Supplementary figure 1-22-2. Pictures from follow up (14.06.19)

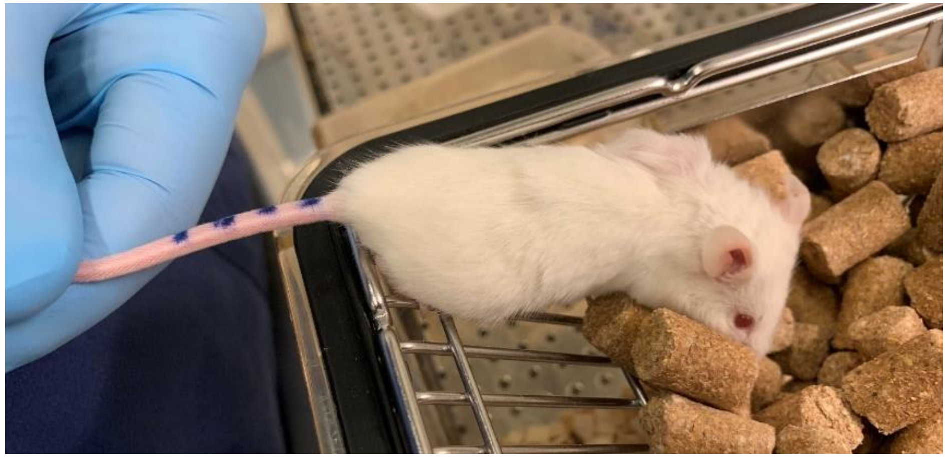

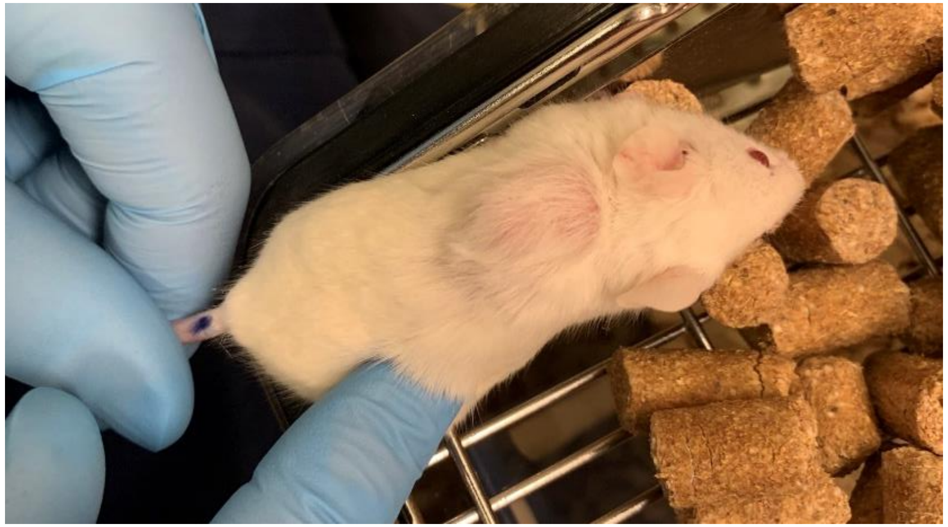

Blood sample

- Before: Vena Saphena 11.06.19
  - Hard to get blood sample from
- After treatment: Heart
- Treatment: Smartfish with fishoil (OMEGA-3)

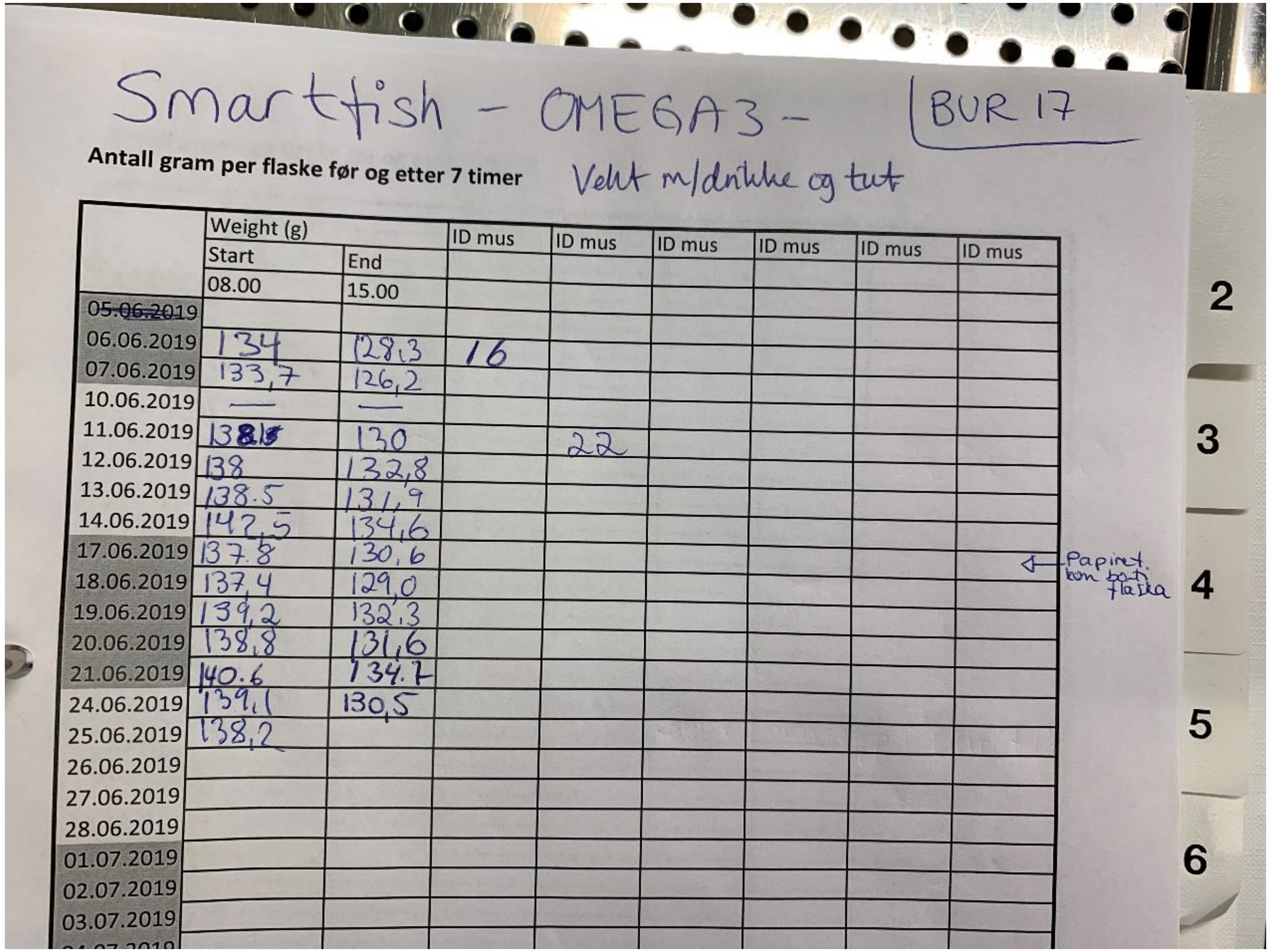

Supplementary figure 1-22-3. List over weight registrations of the drinking bottle (with Smartfish Remune Peach) before and after provided to the mice

Supplementary figure 1-22-4. Pictures from euthanization

- Xenograft size 17x11
- Blood sample (blood card and plasma)
- Tumor tissue for
  - Histology
  - RNA isolation
  - Protein isolation

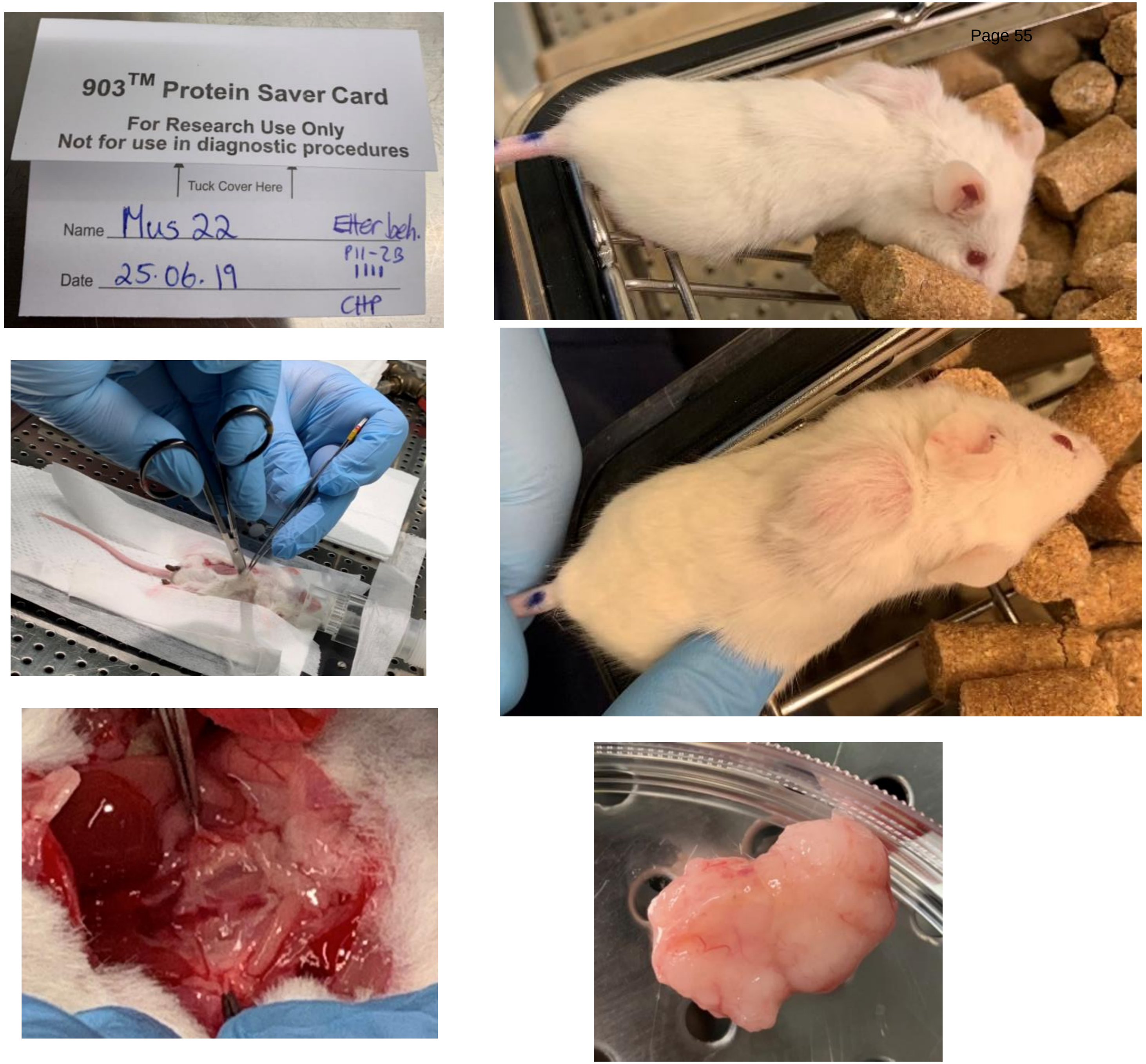

Supplementary figure 1-22-5. Picture of score sheet

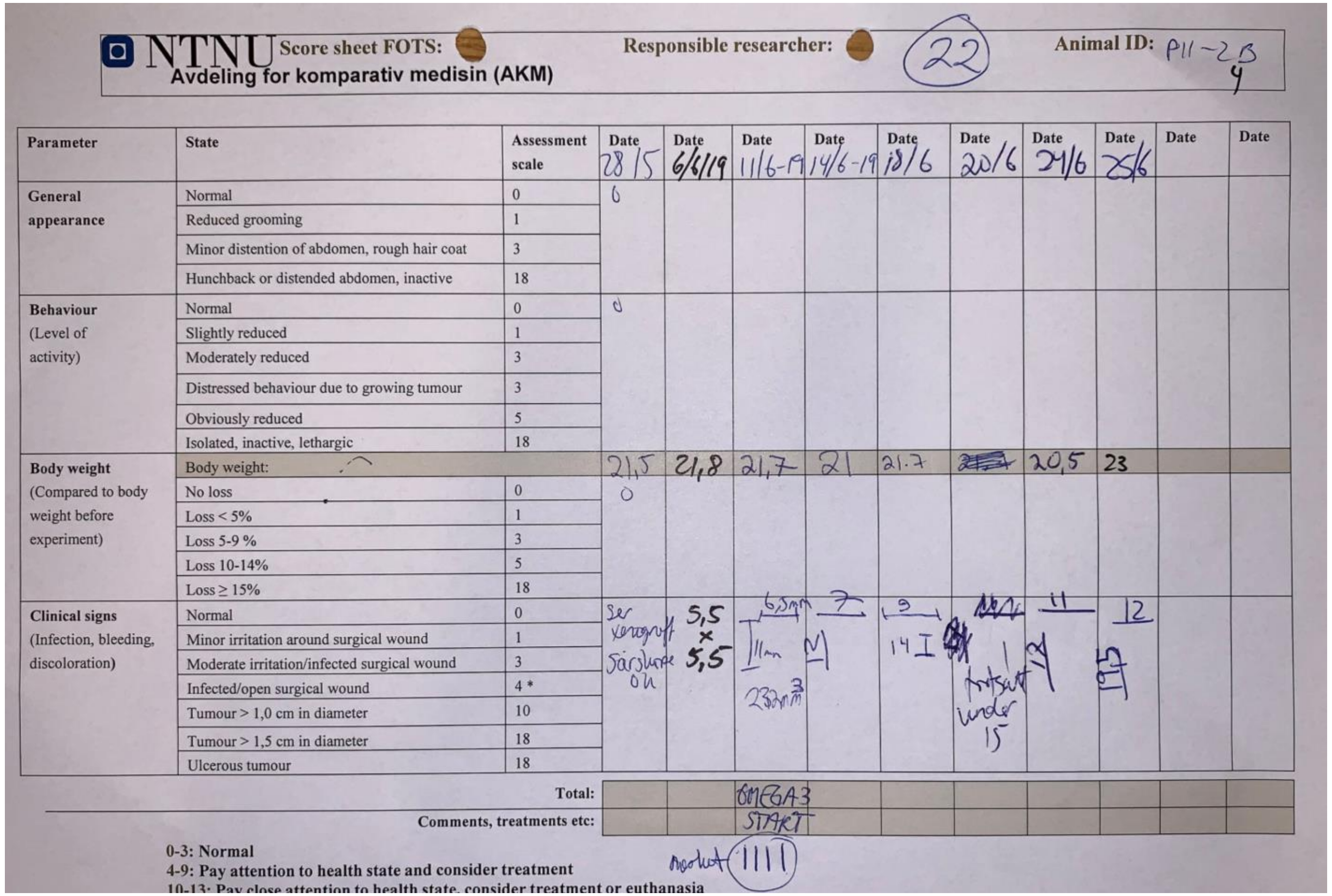

NTNU Score sheet FOTS: Responsible researcher: 22 Animal ID: P11-2B 4

Avdeling for komparativ medisin (AKM)

Parameter	State	Assessment scale	Date 28/5	Date 6/6/19	Date 11/6-19	Date 14/6-19	Date 18/6	Date 20/6	Date 24/6	Date 25/6	Date	Date
General appearance	Normal	0	0									
	Reduced grooming	1										
	Minor distention of abdomen, rough hair coat	3										
	Hunchback or distended abdomen, inactive	18										
Behaviour (Level of activity)	Normal	0	0									
	Slightly reduced	1										
	Moderately reduced	3										
	Distressed behaviour due to growing tumour	3										
	Obviously reduced	5										
	Isolated, inactive, lethargic	18										
Body weight (Compared to body weight before experiment)	Body weight:		21,5	21,8	21,7	21	21.7	[illegible]	20,5	23		
	No loss	0	0									
	Loss < 5%	1										
	Loss 5-9 %	3										
	Loss 10-14%	5										
	Loss ≥ 15%	18										
Clinical signs (Infection, bleeding, discoloration)	Normal	0	Ser xenograft Sårskorpe OK	5,5 x 5,5	6,5mm 11mm 233mm³	7 M	9 14	[illegible] fortsatt under 15	11 [illegible]	12 [illegible]		
	Minor irritation around surgical wound	1										
	Moderate irritation/infected surgical wound	3										
	Infected/open surgical wound	4 *										
	Tumour > 1,0 cm in diameter	10										
	Tumour > 1,5 cm in diameter	18										
	Ulcerous tumour	18										
		Total:			OMEGA3							
		Comments, treatments etc:			START							

[illegible] 1111

0-3: Normal

4-9: Pay attention to health state and consider treatment

10-13: Pay close attention to health state, consider treatment or euthanasia

Supplementary figure 1-22-6. Mouse and xenograft growth curves

Weight curve

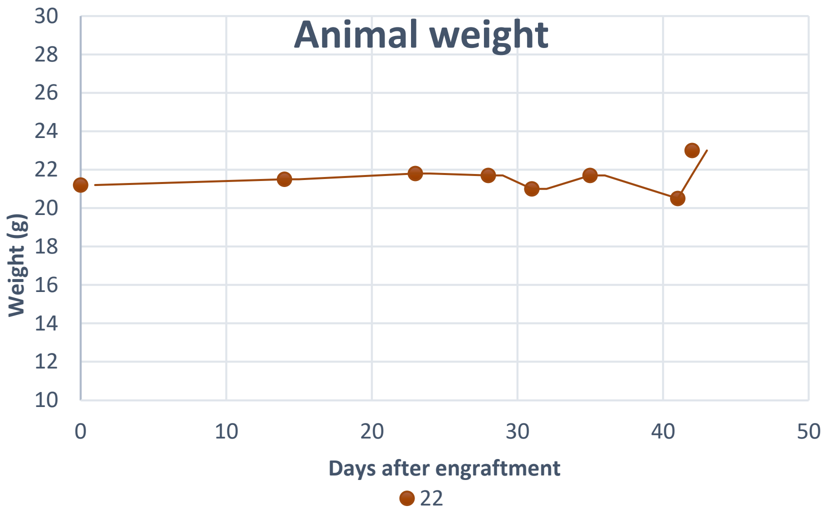

Xenograft growth curve

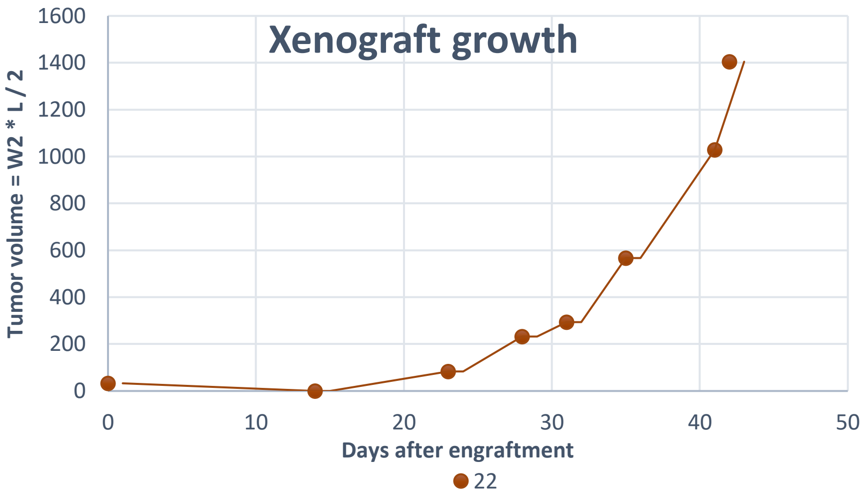

Supplementary figure 1-22-7. Histology pictures

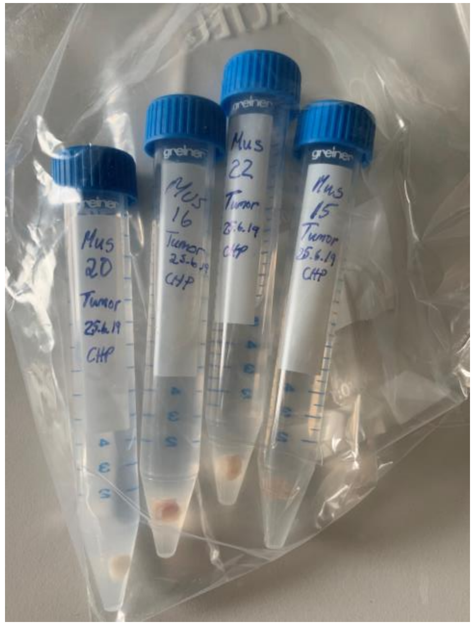

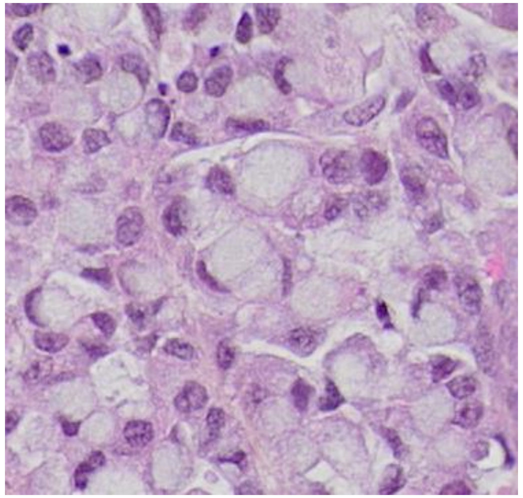

M22X

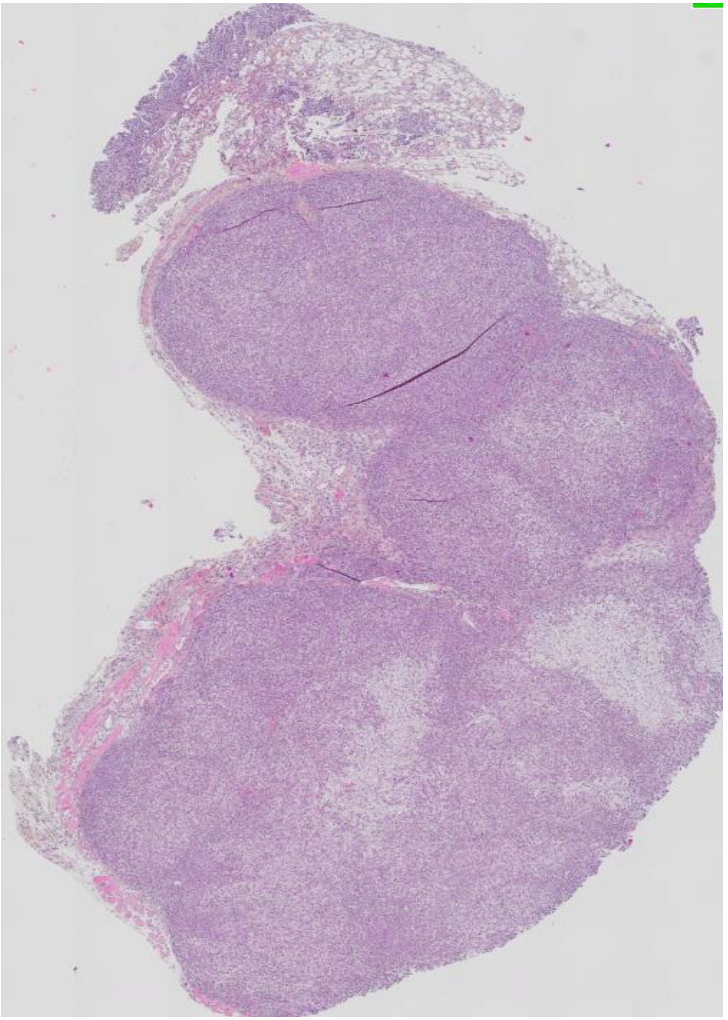

- Origin from signet ring cell adenocarcinoma
- Very simial to patient tumor 3
- Can see several mitotic cells, tumor tissue, connective tissue and some blood vains

Mouse 23

- Surgery date: 20.08.19
  - Age (weeks) 31
  - Weight (g) 24
  - Tumor fragment P17-1A
  - Fragment size 3 x 3 mm
  - Ear mark 1
- Note; opened 2 stiches after 1 h –> restiched

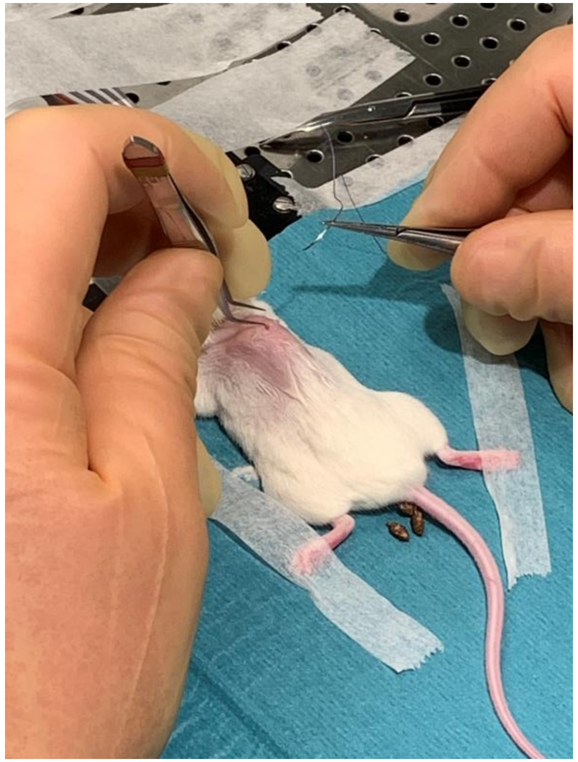

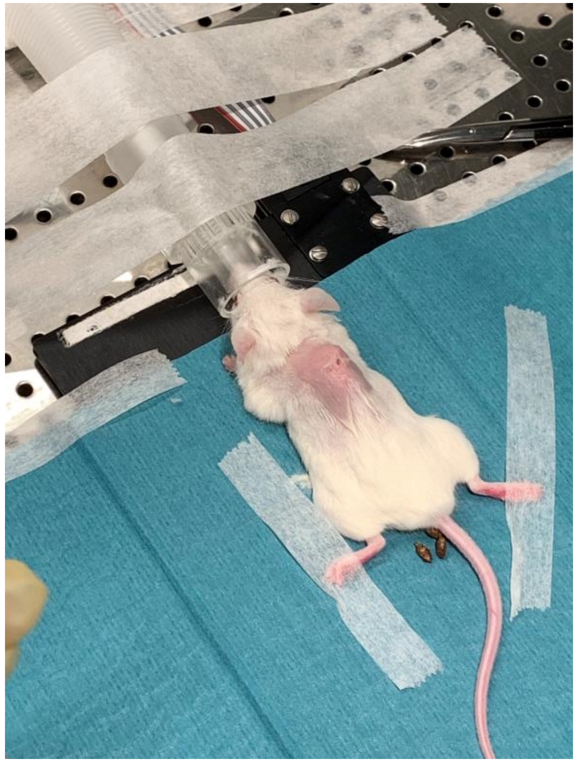

Supplementary figure 1-23-1.
Pictures from the engraftment day

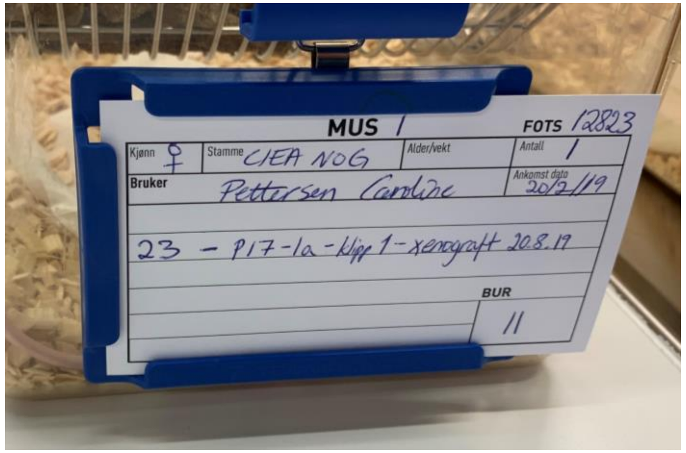

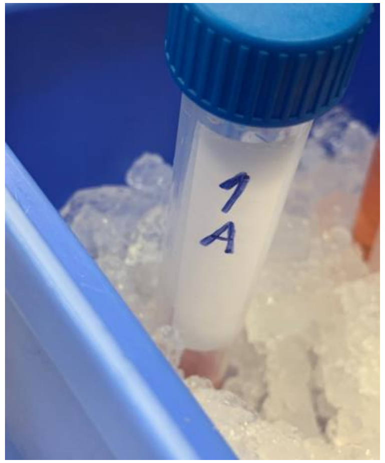

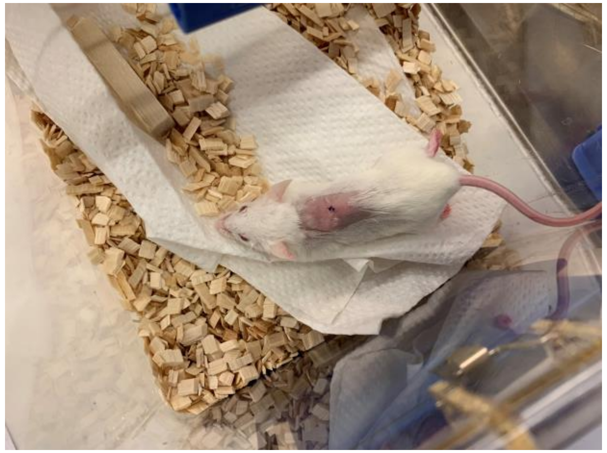

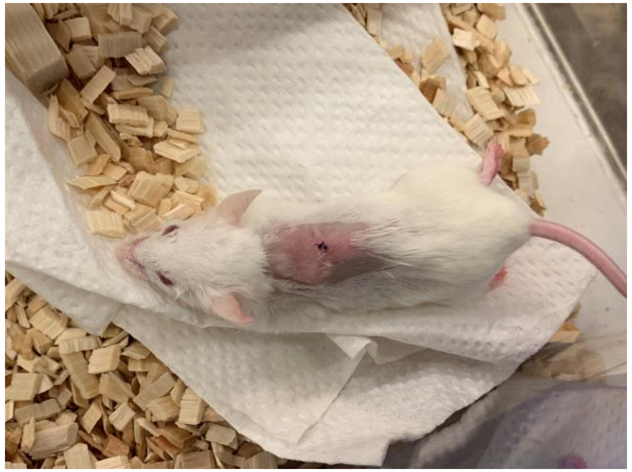

Supplementary figure 1-23-2. Pictures from follow up and tumor size measurement

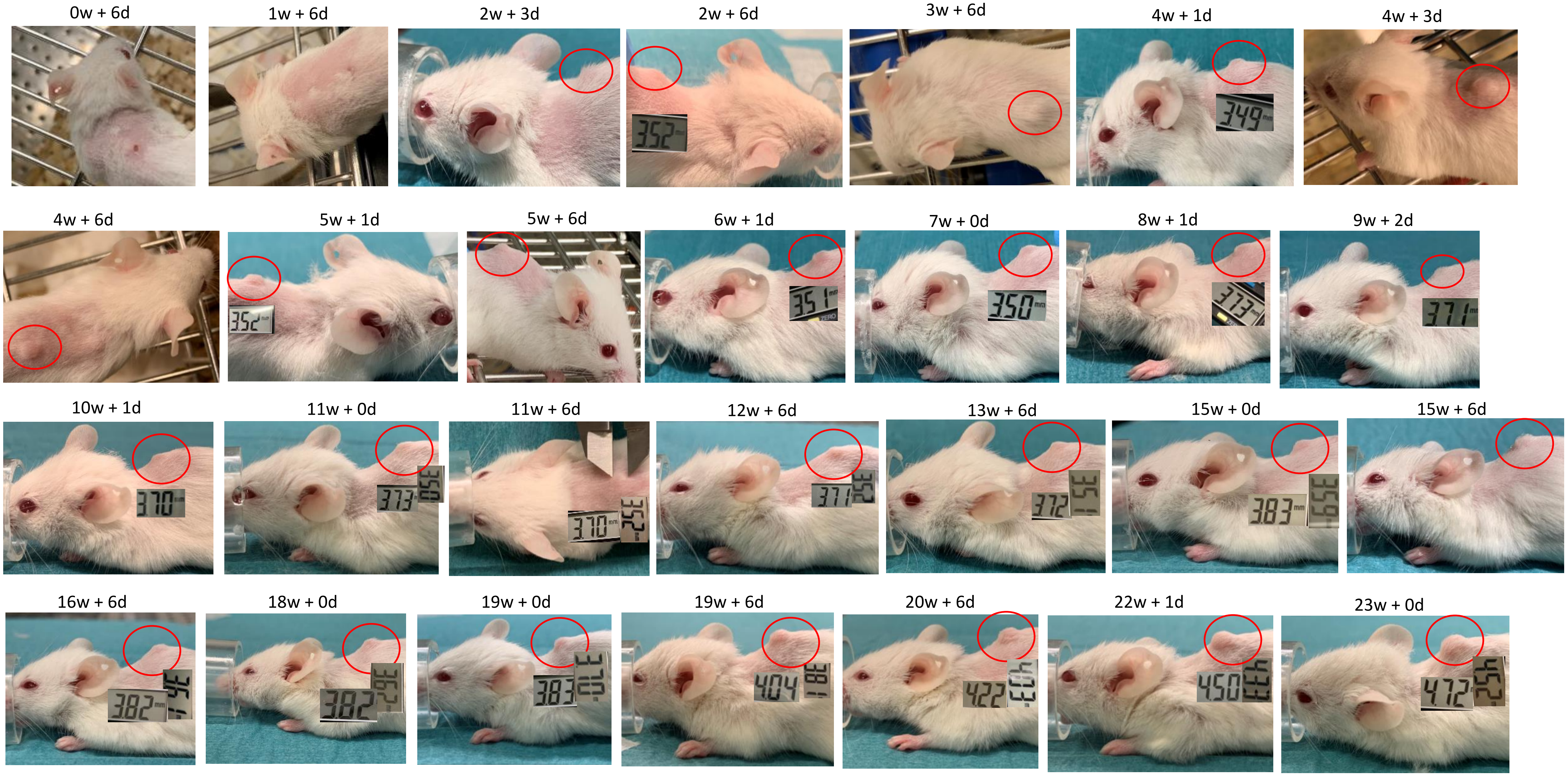

Supplementary figure 1-23-2. Pictures from follow up and tumor size measurement

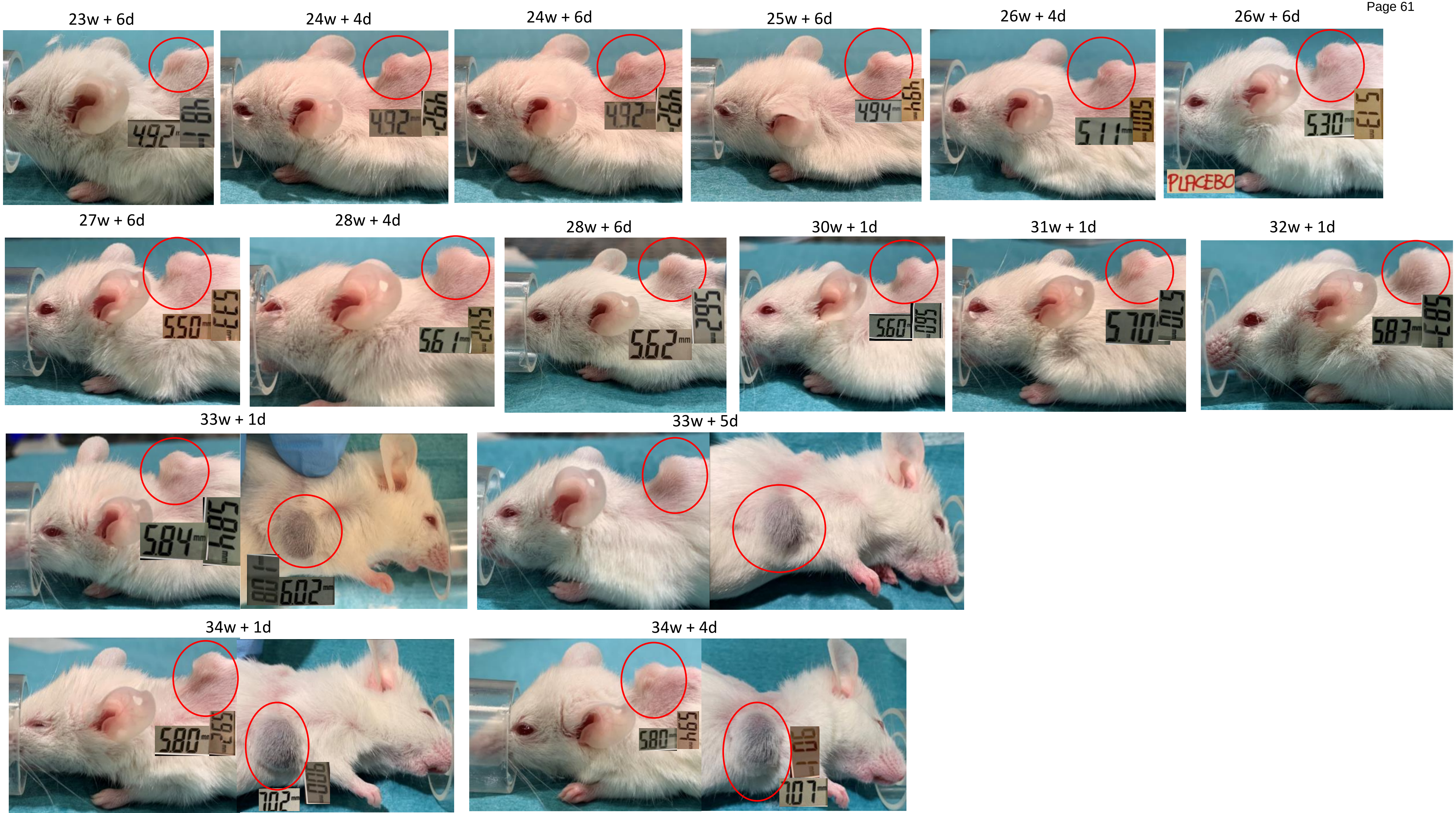

Blood sample (24.02.2020)

- Xenograft 5*5.1 mm
- Stabile weight
- Little blood, anemic?, hard to get blood sample.
- Only 40 µl was spotted on blood card instead of 50 µl.
    - Not blood for preparing plasma
- Starting rectal prolaps? Red in the rectum opening.
- Placebo start 25.02.20

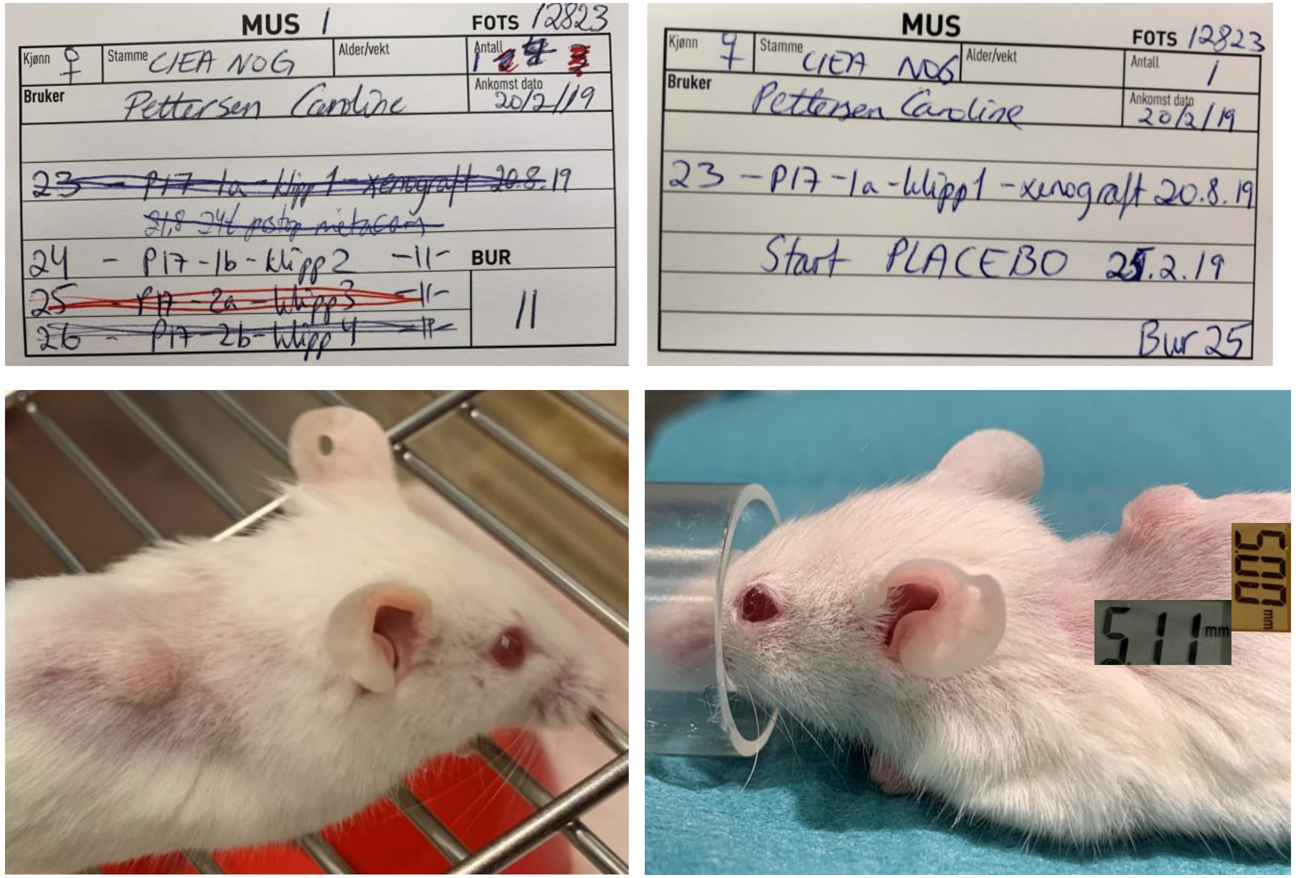

Supplementary figure 1-23-3. Pictures of mouse 23 at treatment startup

Supplementary figure 1-23-4. A) List over weight registrations of drinking bottle (with placebo) before and after provided to the mouse. B) Intake of placebo drink

- Placebo
- Treatment for 8 weeks
- Blood samples taken before/after treatment

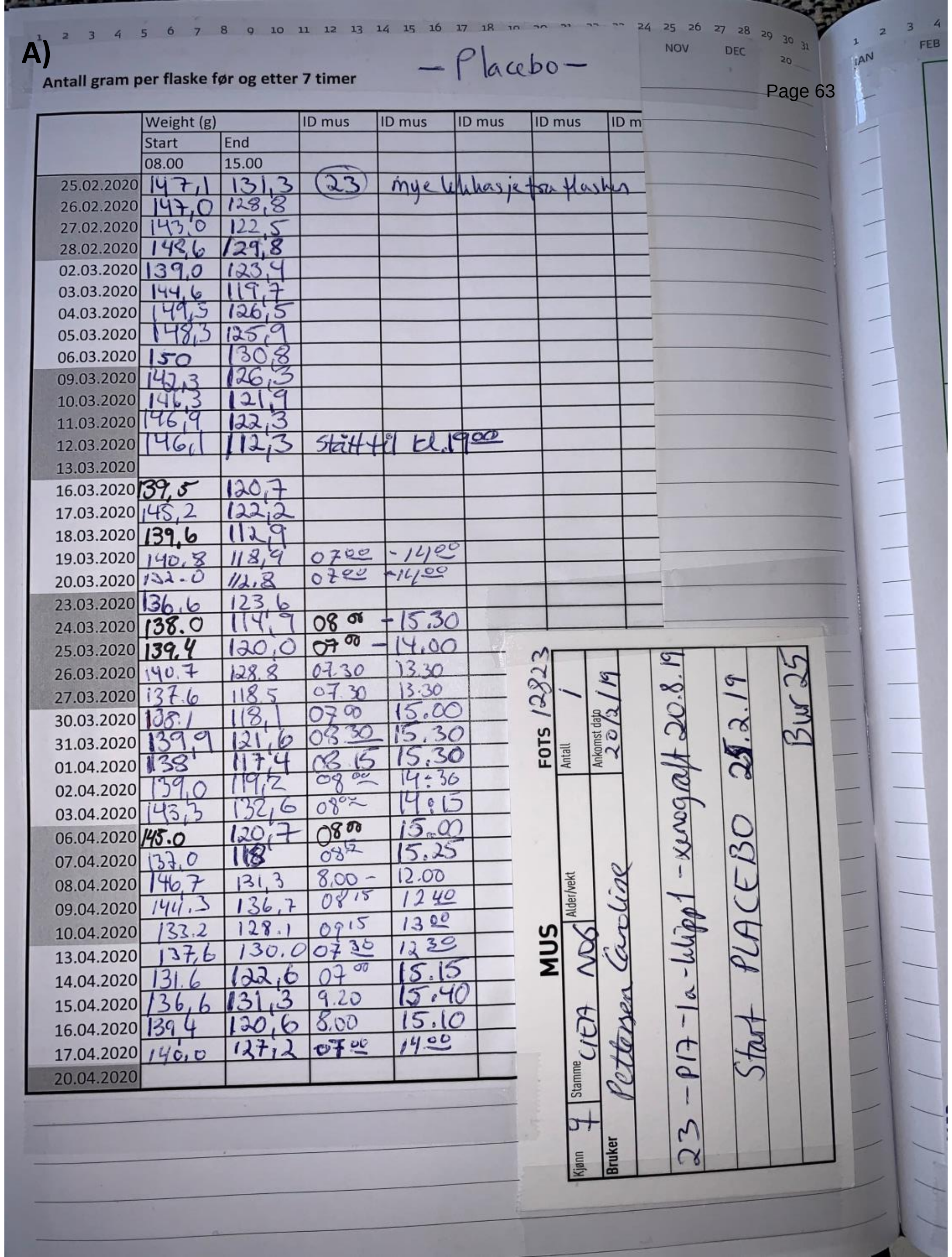

A)

Antall gram per flaske før og etter 7 timer — Placebo —

	Weight (g)		ID mus	ID mus	ID mus	ID mus	ID m
	Start	End					
	08.00	15.00					
25.02.2020	147,1	131,3	(23)	mye lekkasje fra flasken			
26.02.2020	147,0	128,8					
27.02.2020	143,0	122,5					
28.02.2020	148,6	129,8					
02.03.2020	139,0	123,4					
03.03.2020	144,6	119,7					
04.03.2020	149,5	126,5					
05.03.2020	148,3	125,9					
06.03.2020	150	130,8					
09.03.2020	142,3	126,3					
10.03.2020	146,3	121,9					
11.03.2020	146,9	122,3					
12.03.2020	146,1	112,3	Stått til kl. 19^{00}				
13.03.2020							
16.03.2020	139,5	120,7					
17.03.2020	145,2	122,2					
18.03.2020	139,6	112,9					
19.03.2020	140,8	118,9	07^{00}	- 14^{00}			
20.03.2020	132.0	112,8	07^{00}	- 14^{00}			
23.03.2020	136,6	123,6					
24.03.2020	138.0	114,9	08^{00}	- 15.30			
25.03.2020	139,4	120,0	07^{00} -	14.00			
26.03.2020	140.7	128.8	07.30	13.30			
27.03.2020	137.6	118.5	07.30	13.30			
30.03.2020	138.1	118,1	07^{00}	15.00			
31.03.2020	139,9	121,6	08.30	15.30			
01.04.2020	138	117,4	08.15	15.30			
02.04.2020	139,0	119,2	08^{00}	14:30			
03.04.2020	143,3	132,6	08^{00}	14:15			
06.04.2020	145.0	120,7	08^{00}	15.00			
07.04.2020	137,0	118	08^{15}	15.25			
08.04.2020	146,7	131,3	8.00 -	12.00			
09.04.2020	144,3	136,7	08^{15}	12^{40}			
10.04.2020	133.2	128.1	09^{15}	13^{00}			
13.04.2020	137,6	130.0	07^{30}	12^{30}			
14.04.2020	131.6	122,6	07^{00}	15.15			
15.04.2020	136,6	131,3	9.20	15.40			
16.04.2020	139,4	120,6	8.00	15.10			
17.04.2020	140,0	127,2	07^{00}	14^{00}			
20.04.2020							

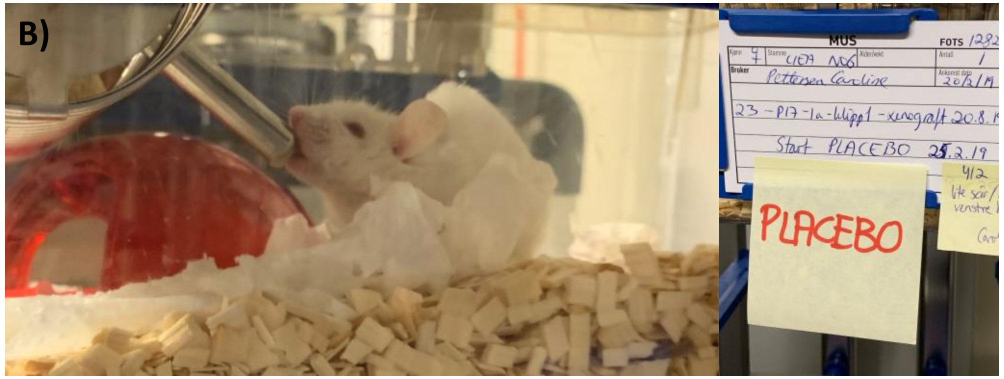

Supplementary figure 1-23-5.
Pictures from euthanization

- Reduced weight
- Necroscopy
  - Xenograft size 5.9 x 5.8 mm
  - Secondary tumor size 7.07 x 9.01 mm
  - Blood sample
  - Tumor tissue for
    - Histology
    - RNA isolation
    - Protein isolation
  - Blood card 40 µl

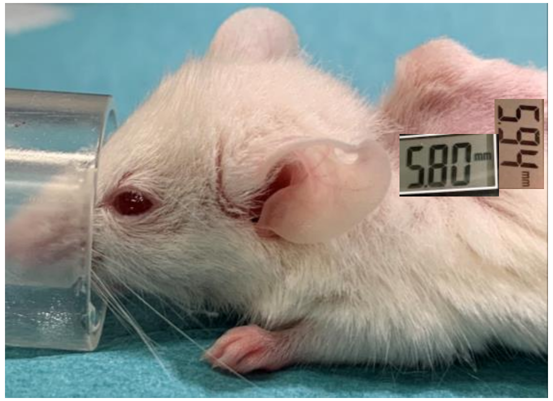

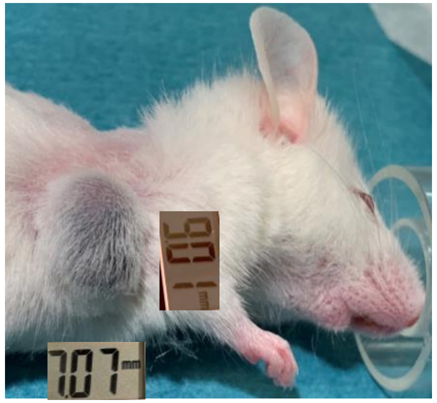

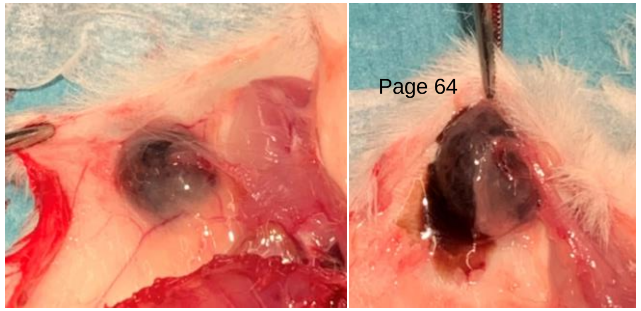

2nd tumor, behind right for leg, dark liquid coming out of it

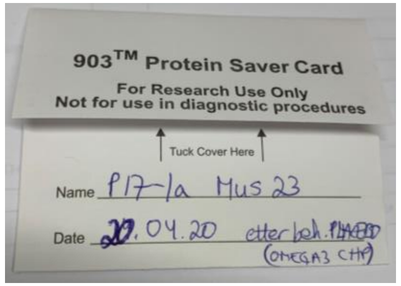
903™ Protein Saver Card
For Research Use Only
Not for use in diagnostic procedures
Tuck Cover Here
Name P17-1a Mus 23
Date 20.04.20 etter beh. PLACEBO (OMEGA3 CTR)

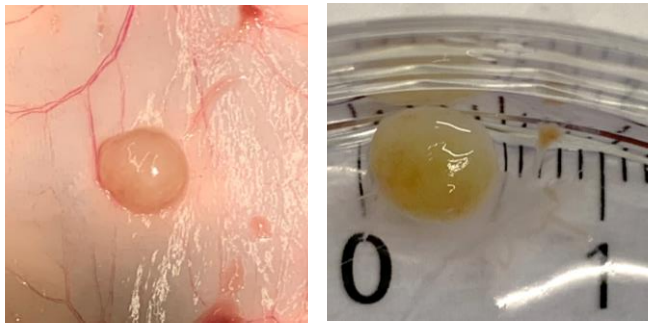

Xenograft attached to the skin

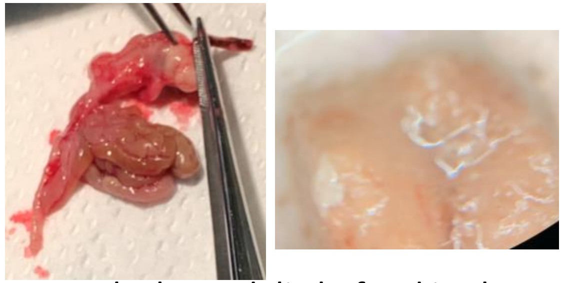

Stomach clogged, little food in the colon right; clog inspected in microscope (hair and food)

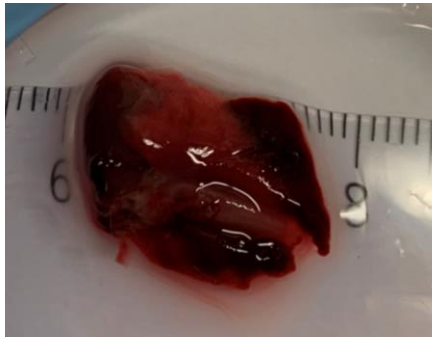

Lungs and heart.
Small lungs?

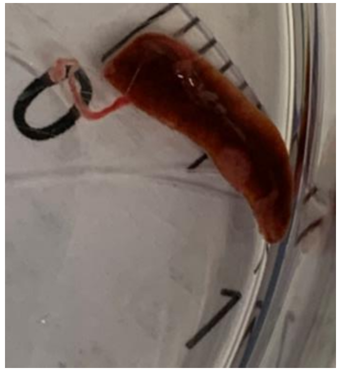

Spleen, small/normal, ca 1 cm

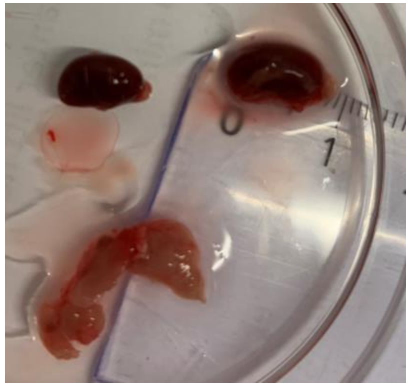

Kidneys and thymus

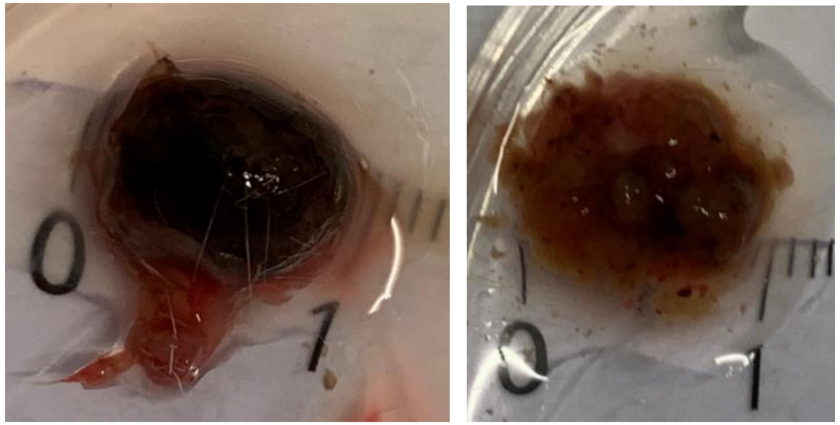

2nd tumor, before and after flushed with NaCl

Supplementary figure 1-23-6. Autopsy card filled out during necroscopy

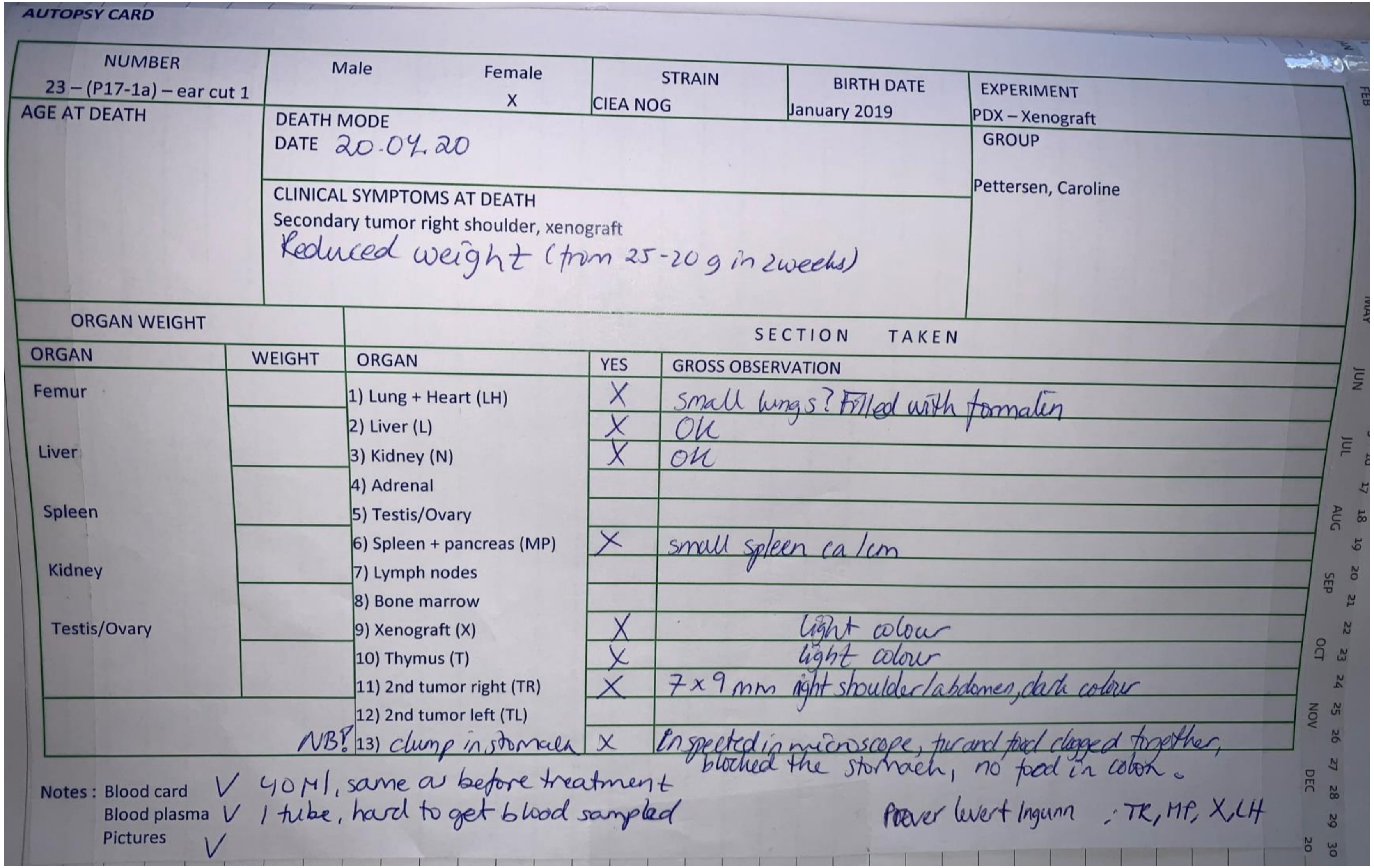

AUTOPSY CARD

NUMBER	Male	Female	STRAIN	BIRTH DATE	EXPERIMENT
23 – (P17-1a) – ear cut 1		X	CIEA NOG	January 2019	PDX – Xenograft

AGE AT DEATH

DEATH MODE

DATE 20.04.20

CLINICAL SYMPTOMS AT DEATH

Secondary tumor right shoulder, xenograft

Reduced weight (from 25-20 g in 2weeks)

GROUP

Pettersen, Caroline

ORGAN WEIGHT

ORGAN	WEIGHT
Femur	
Liver	
Spleen	
Kidney	
Testis/Ovary	

SECTION TAKEN

ORGAN	YES	GROSS OBSERVATION
1) Lung + Heart (LH)	X	small lungs? Filled with formalin
2) Liver (L)	X	OK
3) Kidney (N)	X	OK
4) Adrenal		
5) Testis/Ovary		
6) Spleen + pancreas (MP)	X	small spleen ca 1cm
7) Lymph nodes		
8) Bone marrow		
9) Xenograft (X)	X	light colour
10) Thymus (T)	X	light colour
11) 2nd tumor right (TR)	X	7 x 9 mm right shoulder/abdomen, dark colour
12) 2nd tumor left (TL)		
NB! 13) clump in stomach	X	Inspected in microscope, fur and food clogged together, blocked the stomach, no food in colon.

Notes: Blood card V 40 µl, same as before treatment

Blood plasma V 1 tube, hard to get blood sampled

Pictures V

Paever levert Ingunn : TR, MP, X, LH

Autopsy card modified from: http://www.pathbase.net/Necropsy_of_the_Mouse/

Supplementary figure 1-23-7. Score sheets

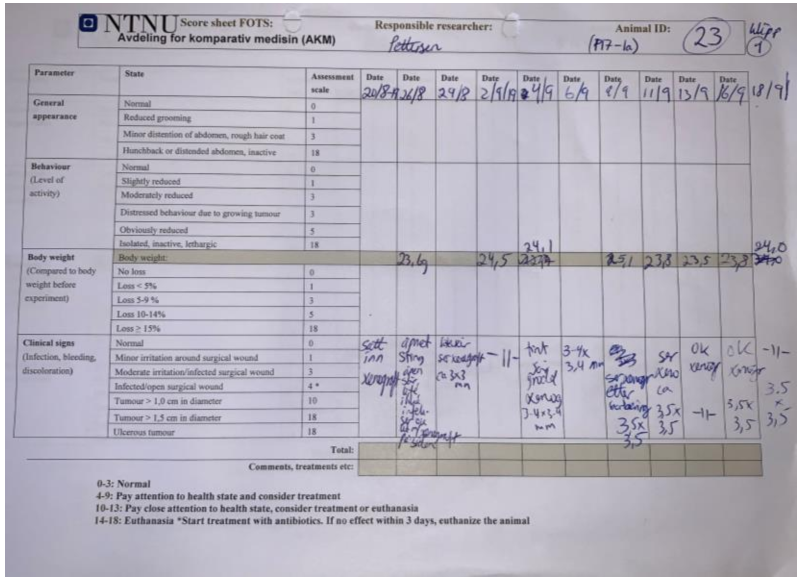

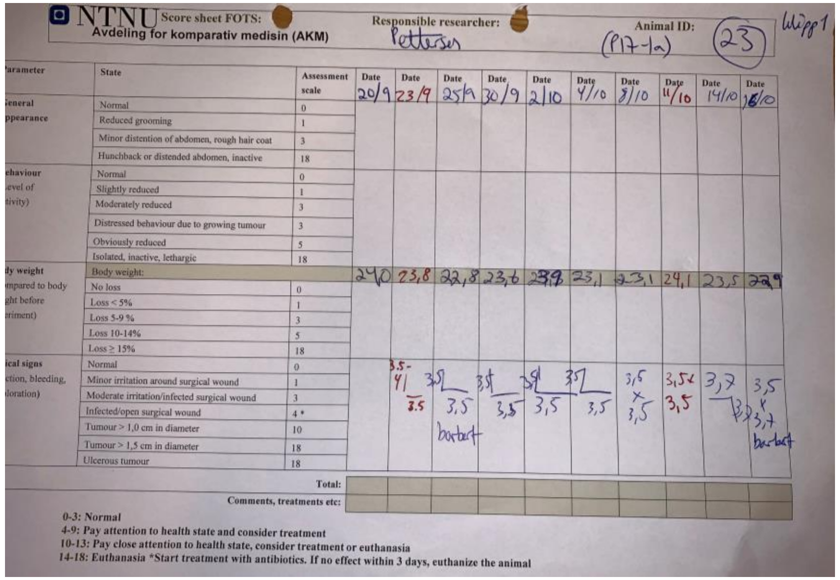

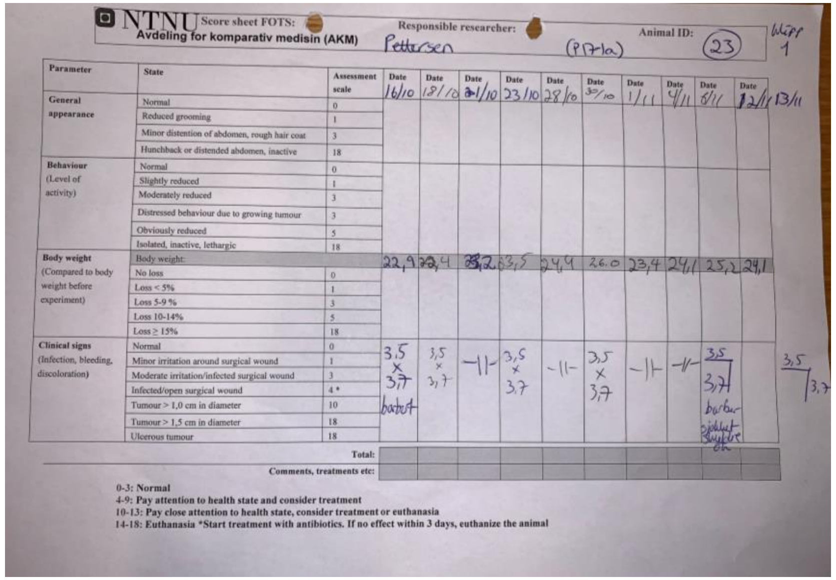

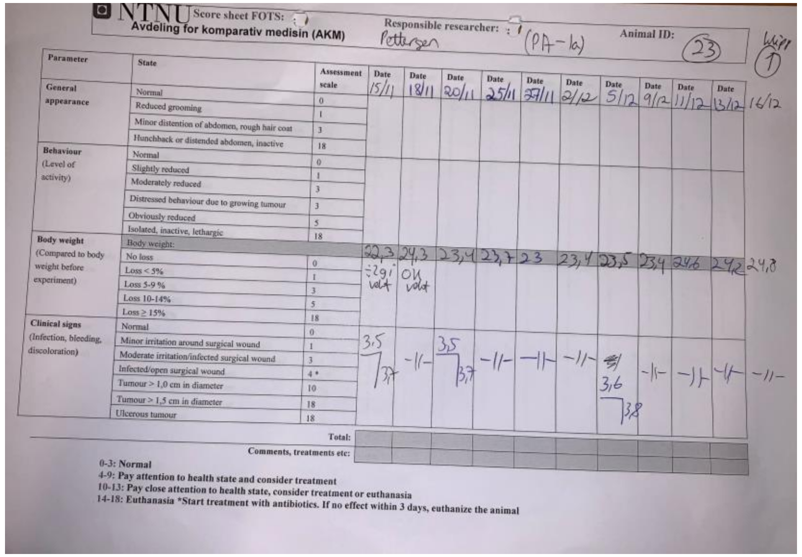

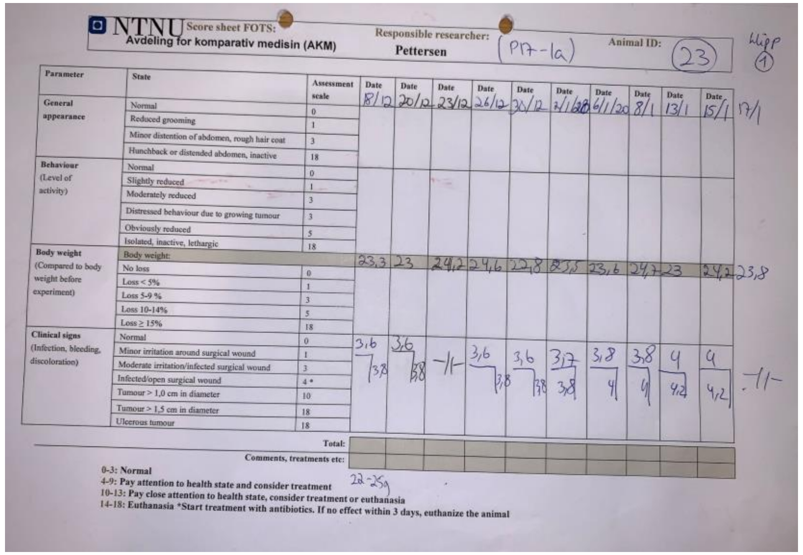

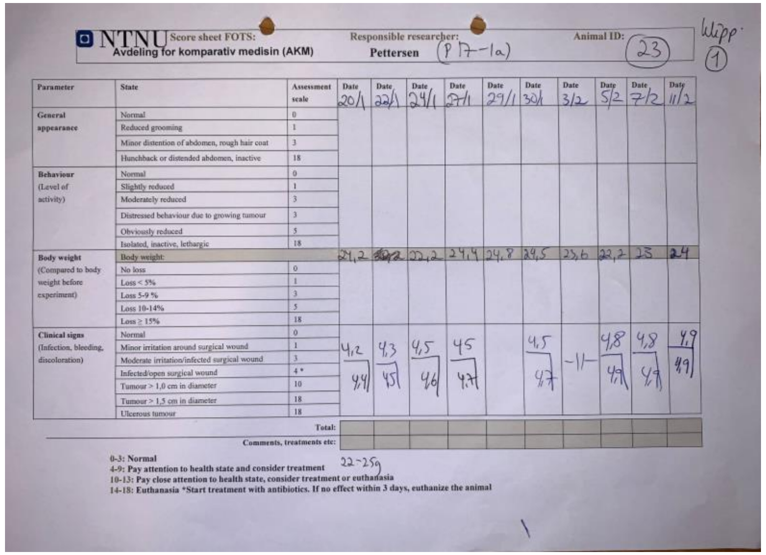

Supplementary figure 1-23-7. Score sheets

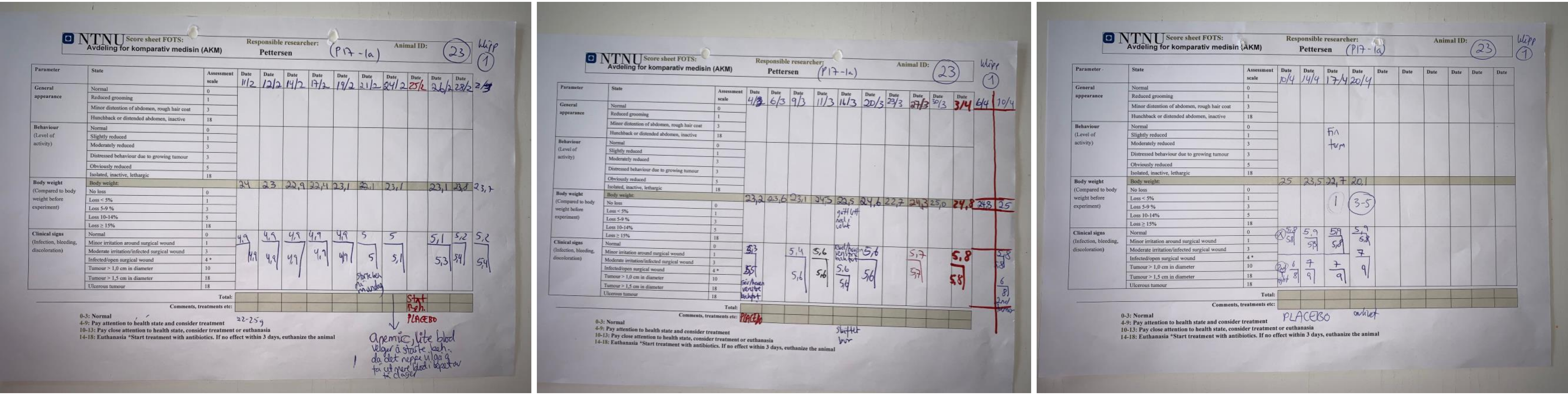

Supplementary figure 1-23-8.
Mouse, xenograft and secondary tumor growth curves

Weight curve

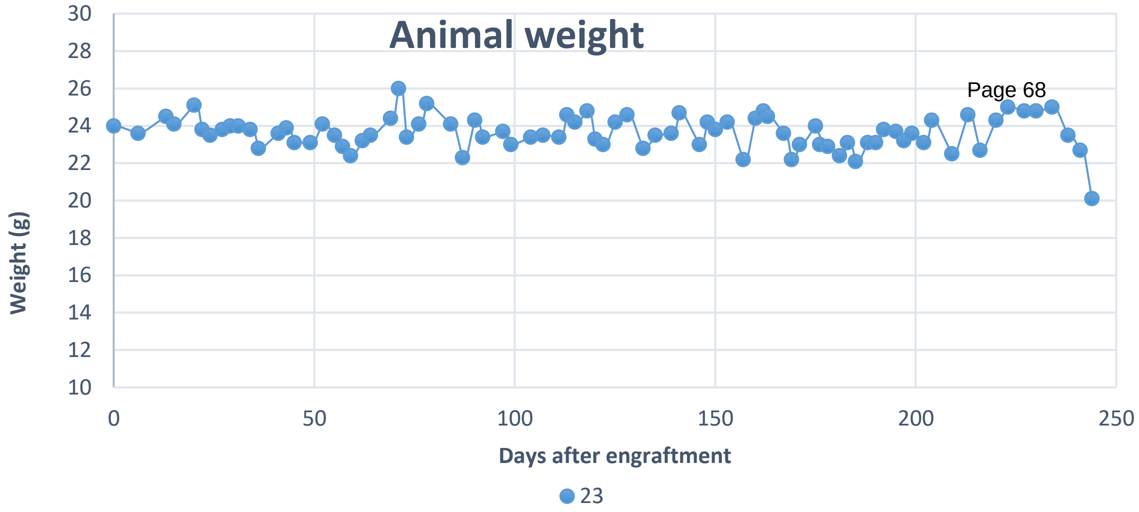

Xenograft growth curve

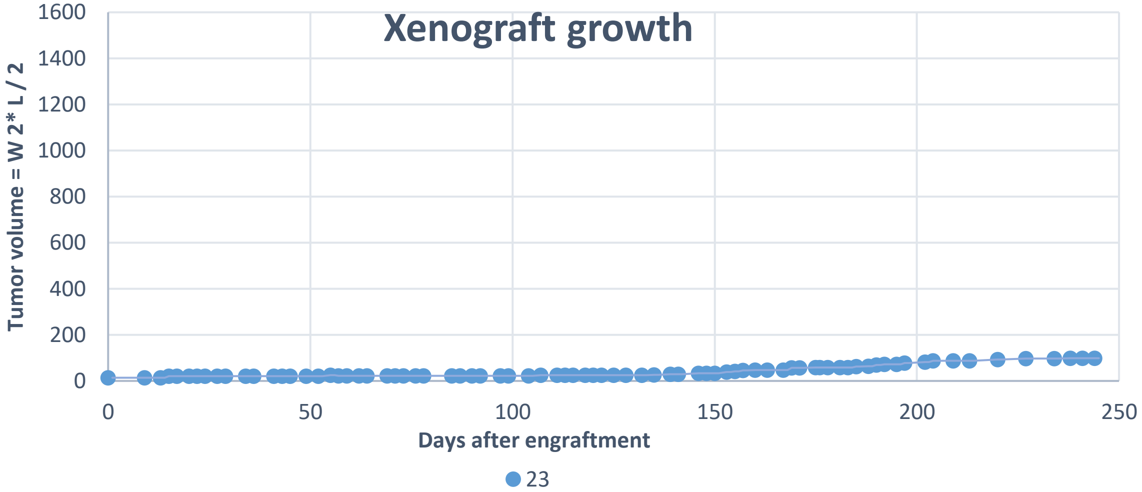

Secondary tumor growth curve

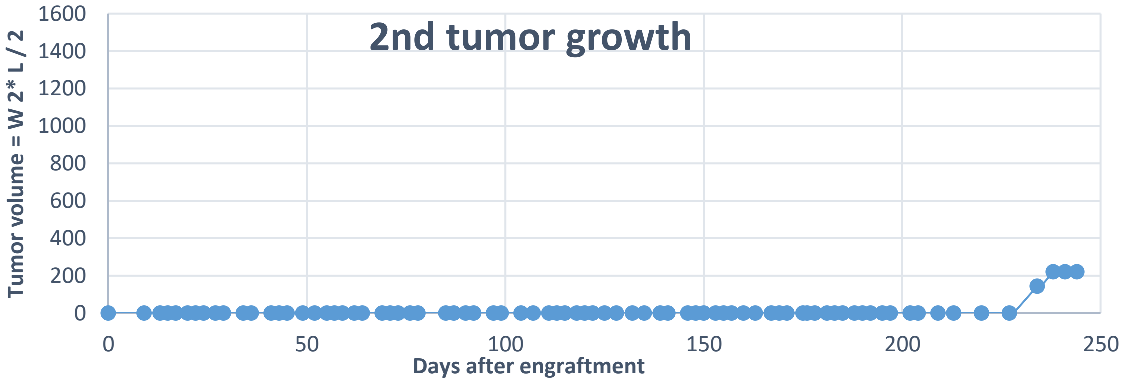

Supplementary figure 1-23-9. Histology pictures

X – xenograft

- CRC Tumor glandulas with necrotic debris in the lumen
- No lymphoma cells
- Mucus
- Necrotic tissue
- Connective tissue

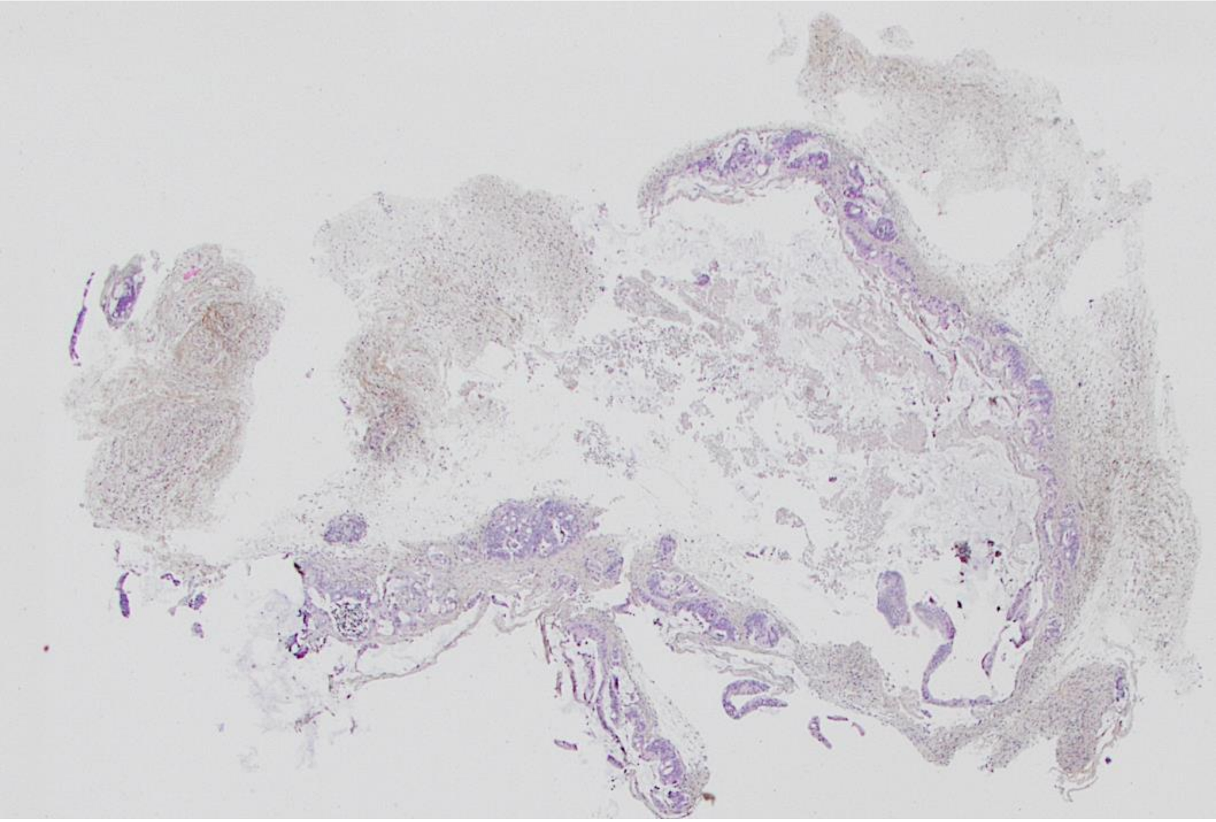

TR – tumor right side (dark bladder with liquid)

- Possible some kind of cyst
- Liquid filled structures
- Does not look malign
- Regularly epithelia

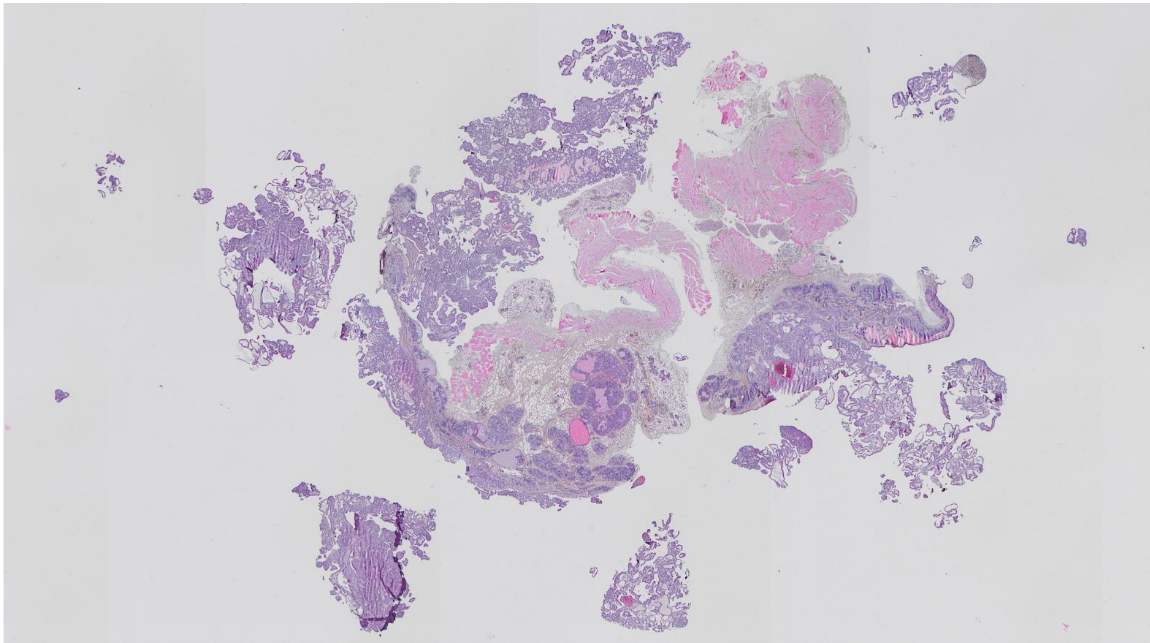

Supplementary figure 1-23-9. Histology pictures

Lungs and heart

- Look normal, however a bit compact lungs
- Possibly macrophages in the lungs

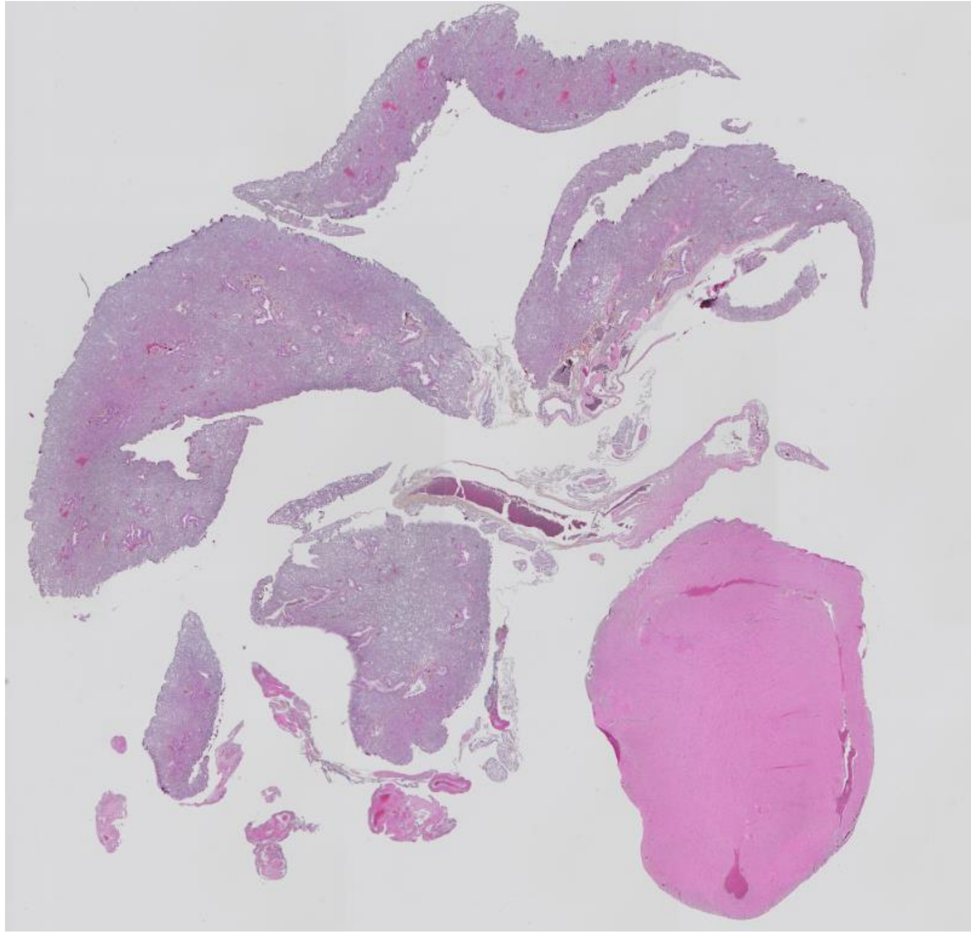

Spleen and pancreas

- pancreas looks normal
- no lyphoid cancer cells
- fibrotic field in the middle of the spleen

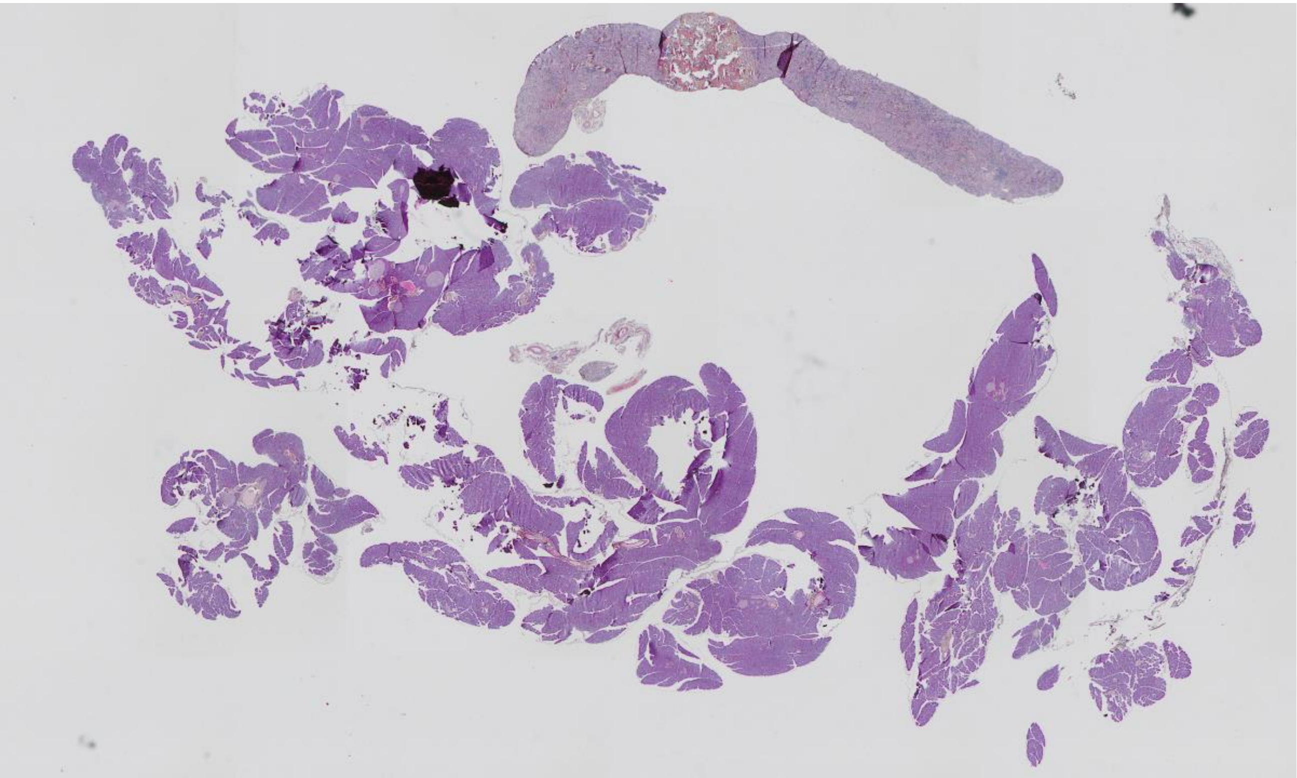

Mouse 24

- Surgery date: 20.08.19
    - Age (weeks) 31
    - Weight (g) 23.6
    - Tumor fragment P17-1B
    - Fragment size 3 x 3 mm
    - Ear mark 2

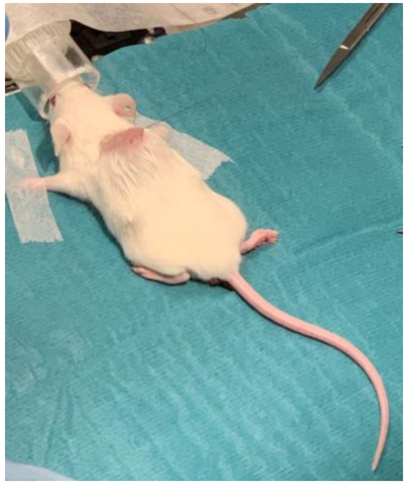
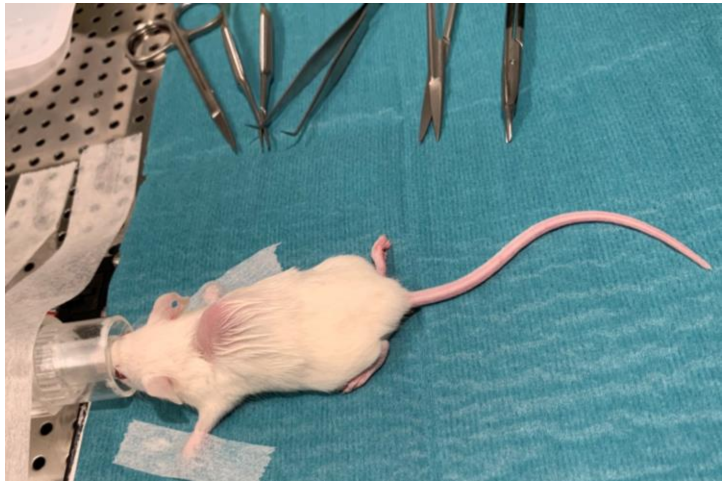
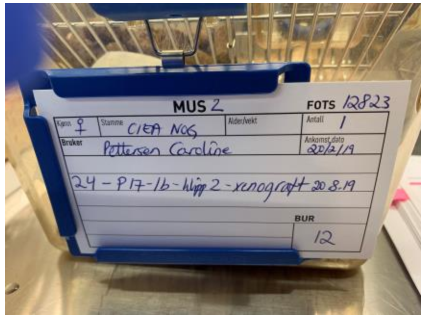

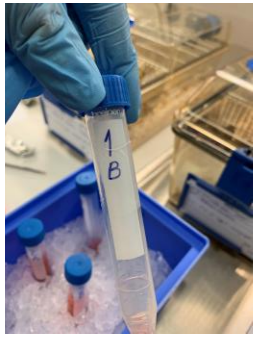

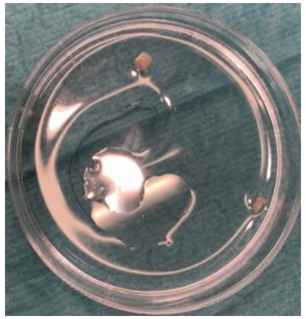
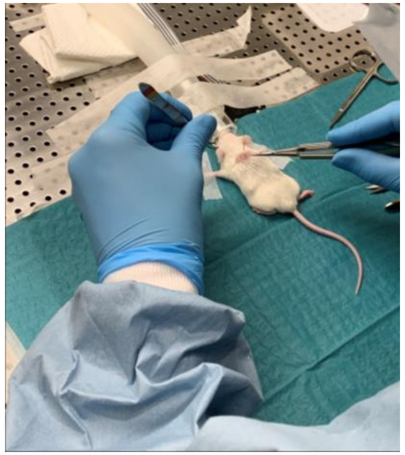
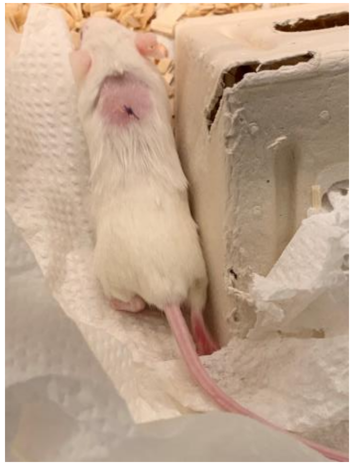
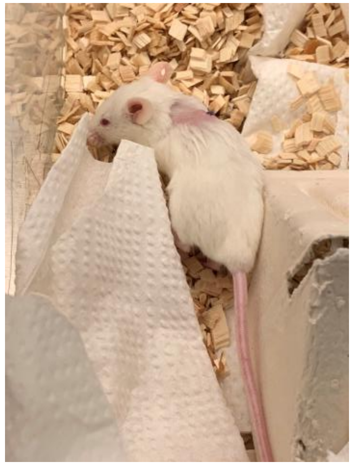

Supplementary figure 1-24-1. Pictures from the engraftment day

Supplementary figure 1-24-2. Pictures from follow up and tumor size measurement

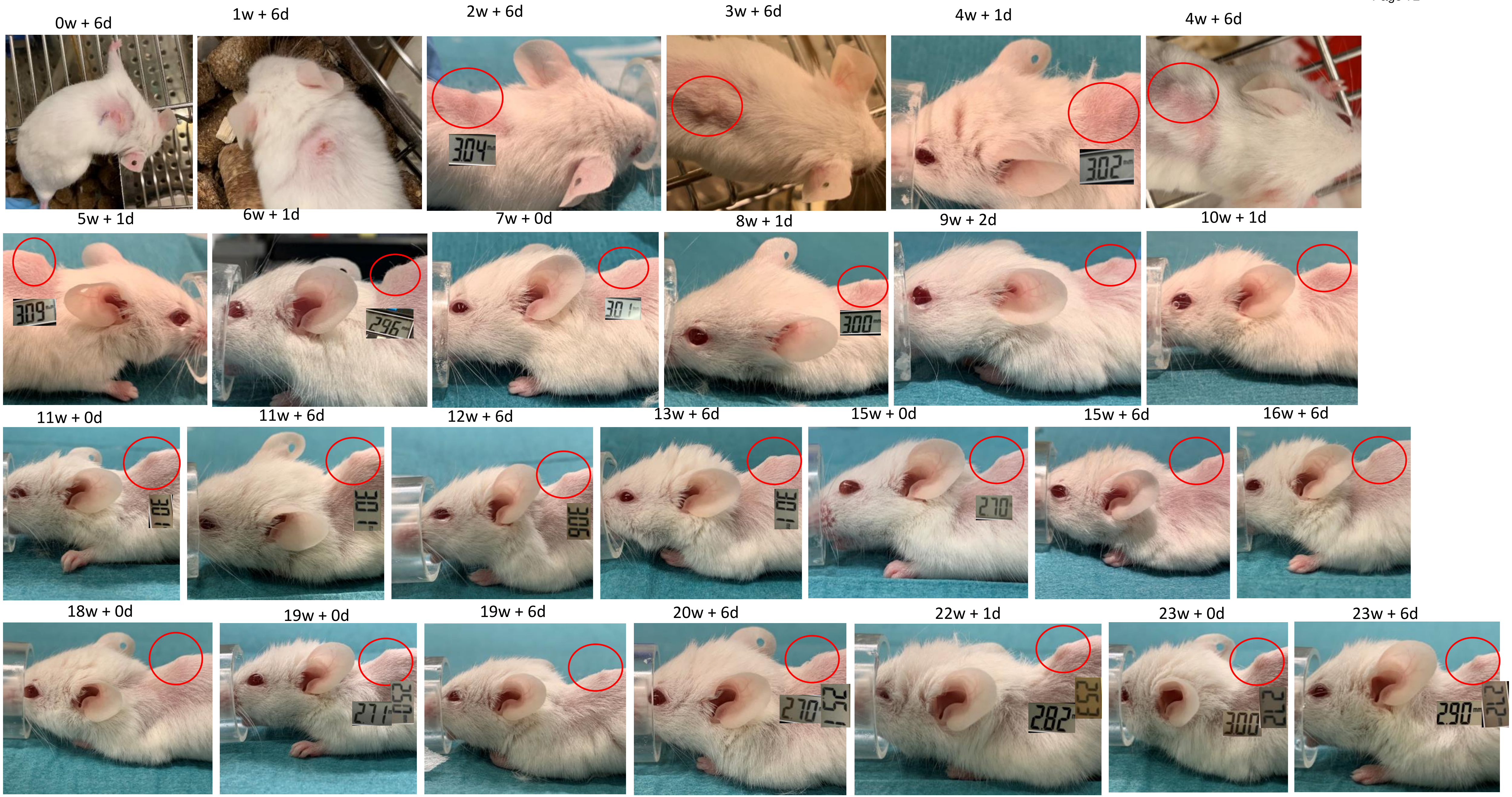

Supplementary figure 1-24-2. Pictures from follow up and tumor size measurement

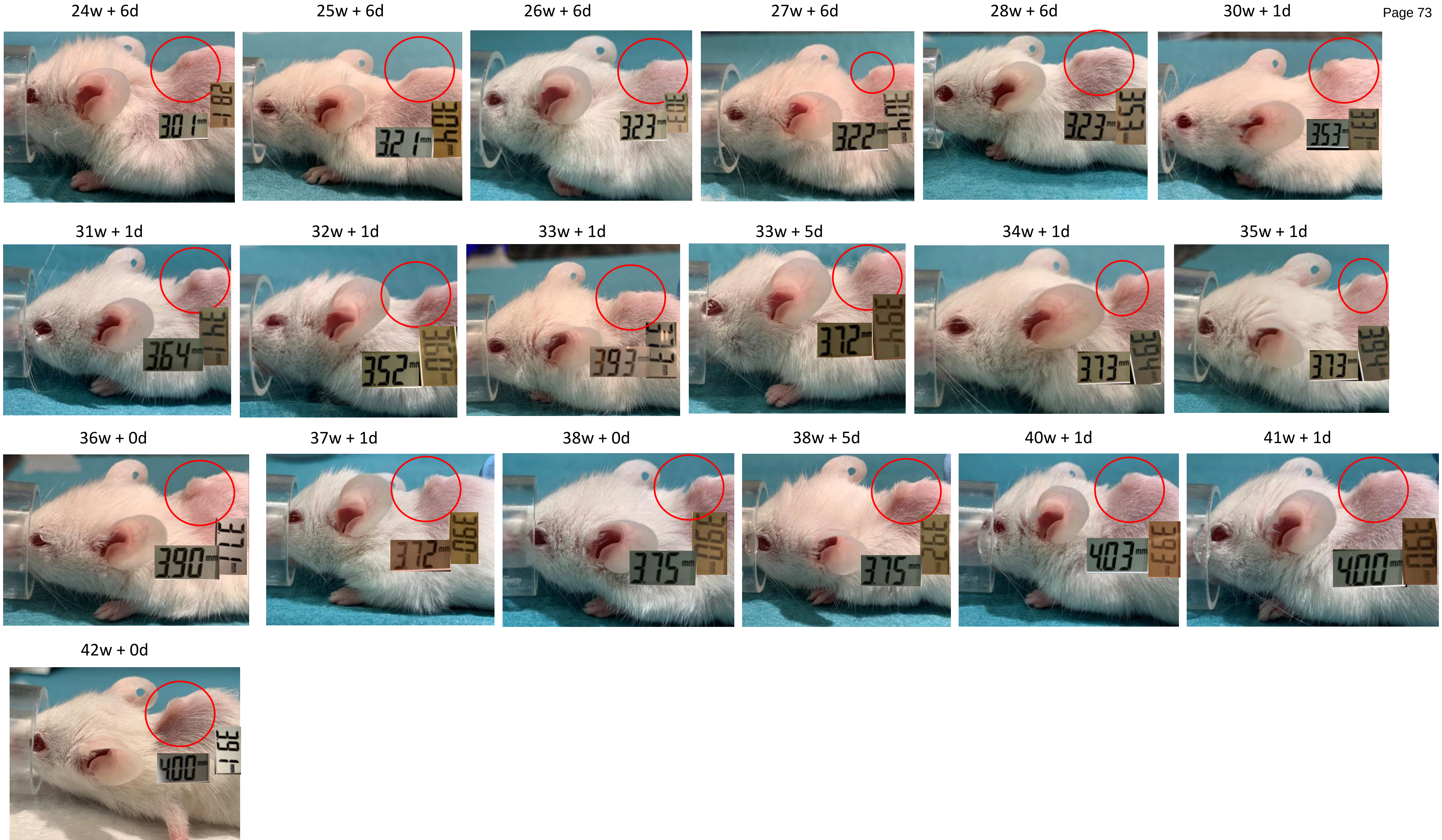

Supplementary figure 1-24-3. Pictures from euthanization

- No treatment given; Did not pass tumor size indicating treatment start
- Necroscopy
  - Xenograft size
  - Blood sample
    - Blood card and plasma
  - Tumor tissue for
    - Histology (only for histology due to small xenograft size)

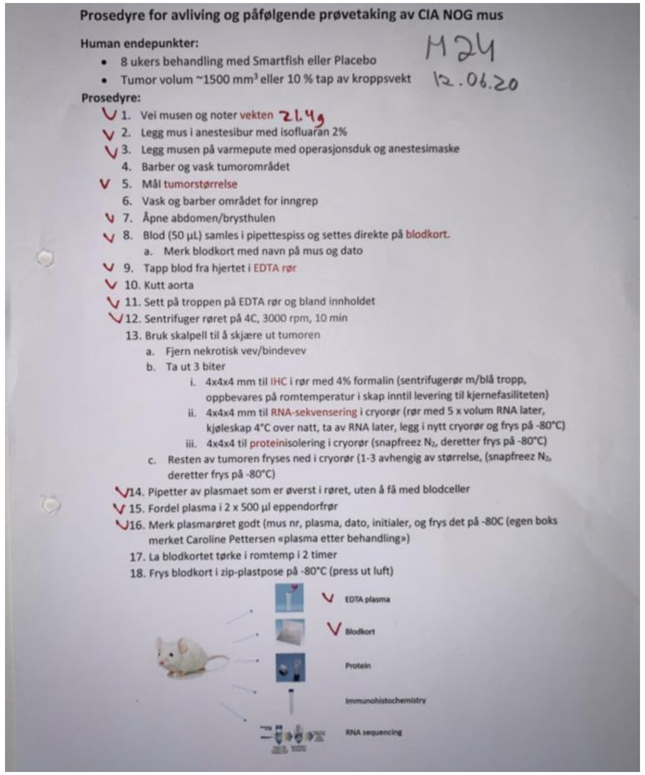

Prosedyre for avliving og påfølgende prøvetaking av CIA NOG mus

M24
12.06.20

Human endepunkter:
- 8 ukers behandling med Smartfish eller Placebo
- Tumor volum ~1500 mm³ eller 10 % tap av kroppsvekt

Prosedyre:
1. Vei musen og noter vekten 21.4g
2. Legg mus i anestesibur med isofluaran 2%
3. Legg musen på varmepute med operasjonsduk og anestesimaske
4. Barber og vask tumorområdet
5. Mål tumorstørrelse
6. Vask og barber området for inngrep
7. Åpne abdomen/brysthulen
8. Blod (50 µL) samles i pipettespiss og settes direkte på blodkort.
   a. Merk blodkort med navn på mus og dato
9. Tapp blod fra hjertet i EDTA rør
10. Kutt aorta
11. Sett på troppen på EDTA rør og bland innholdet
12. Sentrifuger røret på 4C, 3000 rpm, 10 min
13. Bruk skalpell til å skjære ut tumoren
    a. Fjern nekrotisk vev/bindevev
    b. Ta ut 3 biter
       i. 4x4x4 mm til IHC i rør med 4% formalin (sentrifugerør m/blå tropp, oppbevares på romtemperatur i skap inntil levering til kjernefasiliteten)
       ii. 4x4x4 mm til RNA-sekvensering i cryorør (rør med 5 x volum RNA later, kjøleskap 4°C over natt, ta av RNA later, legg i nytt cryorør og frys på -80°C)
       iii. 4x4x4 til proteinisolering i cryorør (snapfreez N_2, deretter frys på -80°C)
    c. Resten av tumoren fryses ned i cryorør (1-3 avhengig av størrelse, (snapfreez N_2, deretter frys på -80°C)
14. Pipetter av plasmaet som er øverst i røret, uten å få med blodceller
15. Fordel plasma i 2 x 500 µl eppendorfrør
16. Merk plasmarøret godt (mus nr, plasma, dato, initialer, og frys det på -80C (egen boks merket Caroline Pettersen «plasma etter behandling»)
17. La blodkortet tørke i romtemp i 2 timer
18. Frys blodkort i zip-plastpose på -80°C (press ut luft)

EDTA plasma
Blodkort
Protein
Immunohistochemistry
RNA sequencing

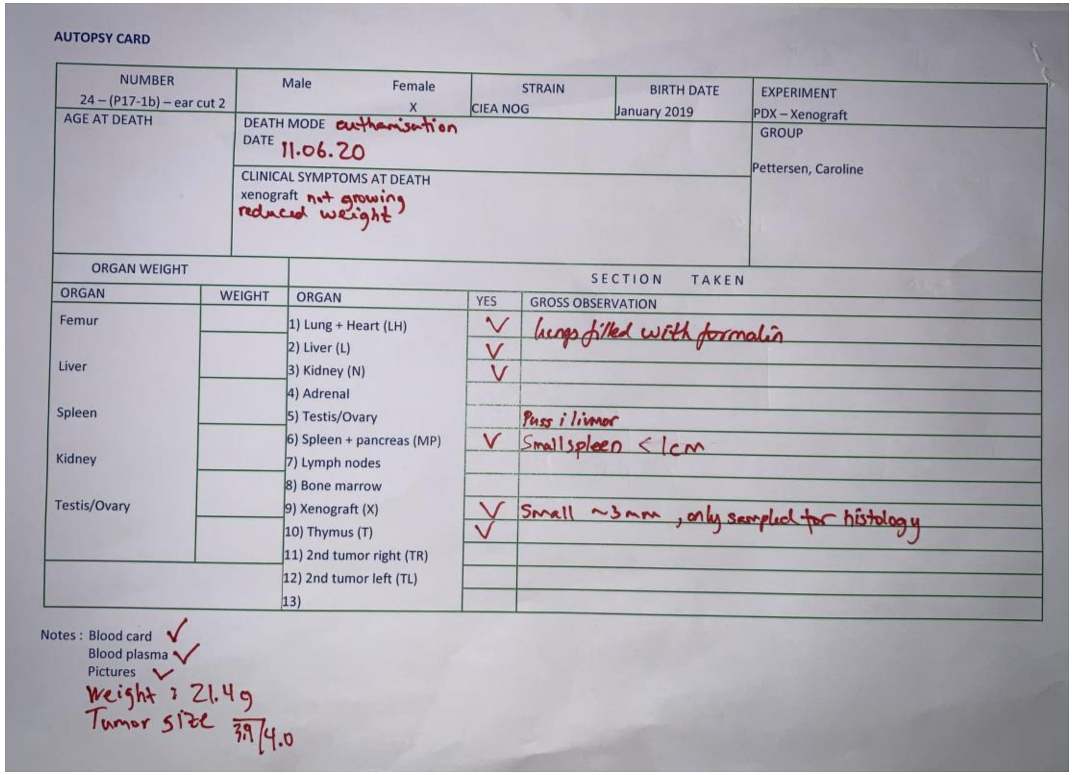

AUTOPSY CARD

NUMBER: 24 – (P17-1b) – ear cut 2
Male / Female: X (Female)
STRAIN: CIEA NOG
BIRTH DATE: January 2019
EXPERIMENT: PDX – Xenograft
AGE AT DEATH
DEATH MODE: euthanisation
DATE: 11.06.20
GROUP: Pettersen, Caroline
CLINICAL SYMPTOMS AT DEATH: xenograft not growing, reduced weight

ORGAN WEIGHT		SECTION TAKEN		
ORGAN	WEIGHT	ORGAN	YES	GROSS OBSERVATION
Femur		1) Lung + Heart (LH)	V	lungs filled with formalin
Liver		2) Liver (L)	V	
Spleen		3) Kidney (N)	V	
Kidney		4) Adrenal		
Testis/Ovary		5) Testis/Ovary		Puss i livmor
		6) Spleen + pancreas (MP)	V	Small spleen < 1cm
		7) Lymph nodes		
		8) Bone marrow		
		9) Xenograft (X)	V	Small ~3mm, only sampled for histology
		10) Thymus (T)	V	
		11) 2nd tumor right (TR)		
		12) 2nd tumor left (TL)		
		13)		

Notes: Blood card ✓
Blood plasma ✓
Pictures ✓
Weight: 21.4g
Tumor size 3.9/4.0

Autopsy card modified from: http://www.pathbase.net/Necropsy_of_the_Mouse/

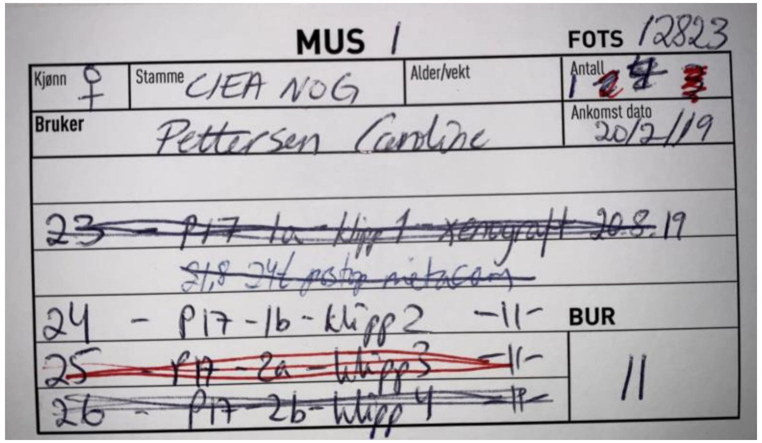

MUS 1 FOTS 12823
Kjønn: ♀ Stamme: CIEA NOG Alder/vekt: Antall: 1 2 3 4
Bruker: Pettersen Caroline Ankomst dato: 20/2/19
~~23 – P17-1a – klipp 1 – xenograft 20.8.19~~
~~31.8 24t postop metacam~~
24 – P17-1b – klipp 2 –"–
~~25 – P17-2a – klipp 3 –"–~~
~~26 – P17-2b – klipp 4 –"–~~
BUR 11

Supplementary figure 1-24-3. Pictures from euthanization (11.06.20)

*Xenograft slow growth

*3 months since animals with not growing xenografts were euthanized

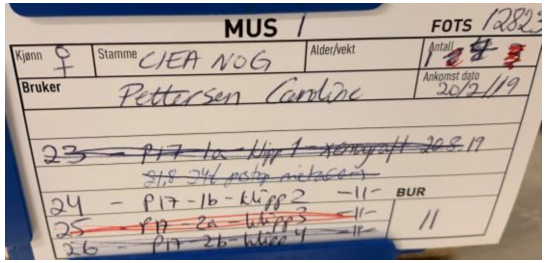

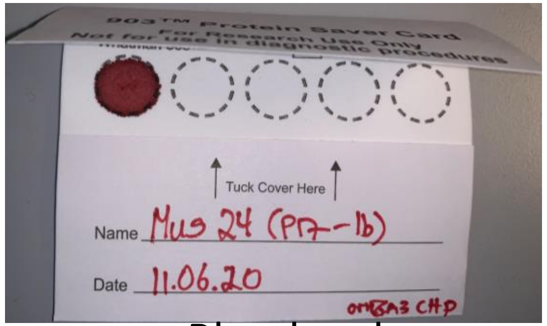

Bloodcard

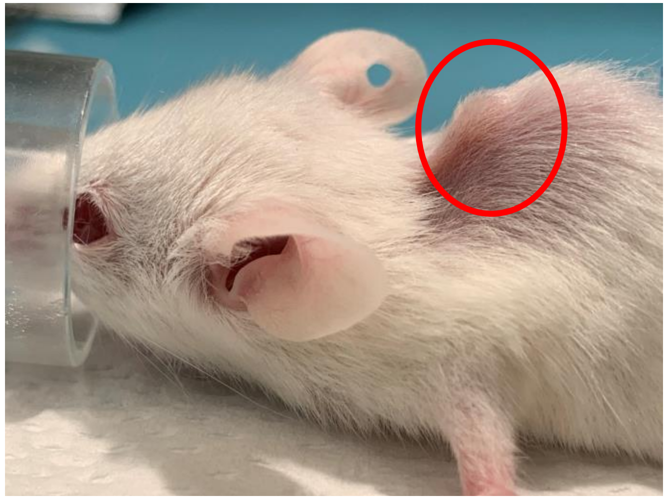
Xenograft (3.9 * 4.0 mm)

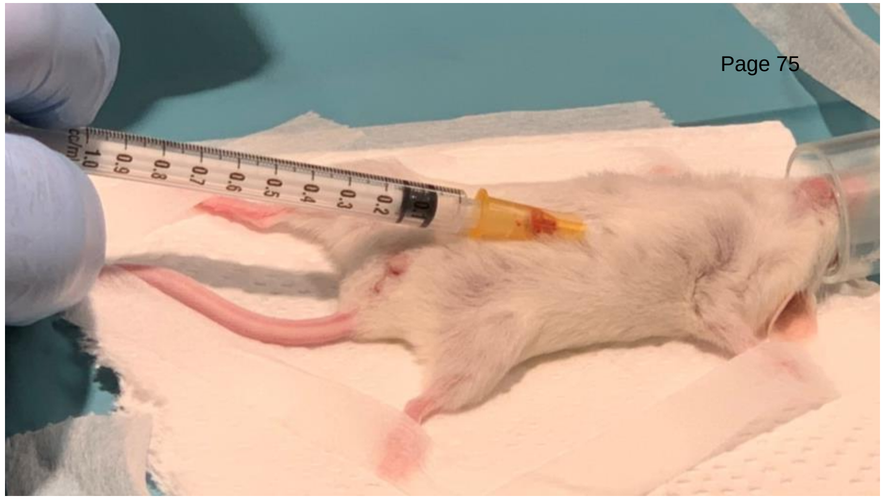
Blood draining

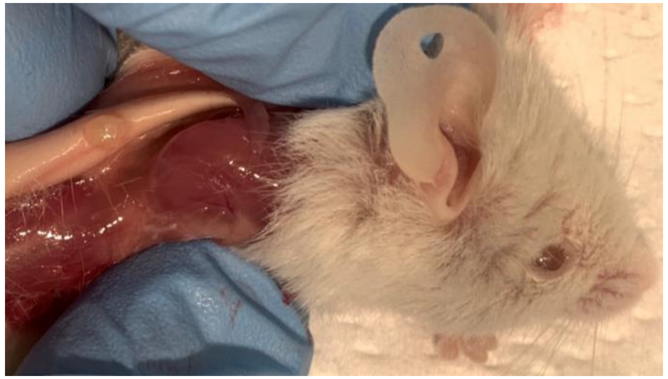
Xenograft

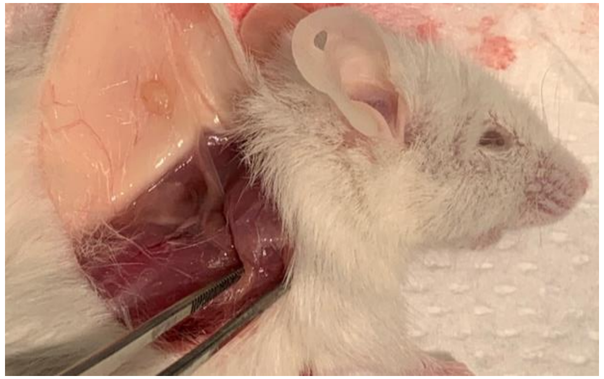
Xenograft

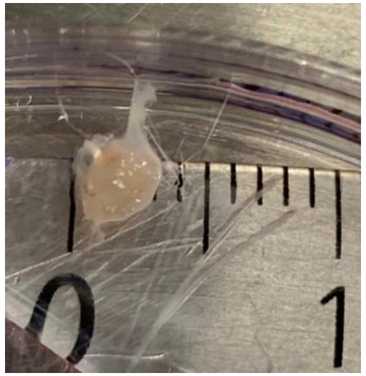
Xenograft

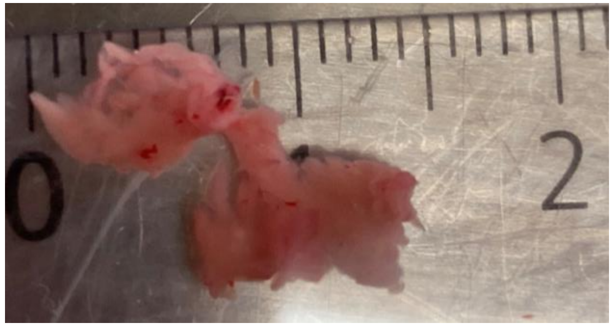
Pancreas

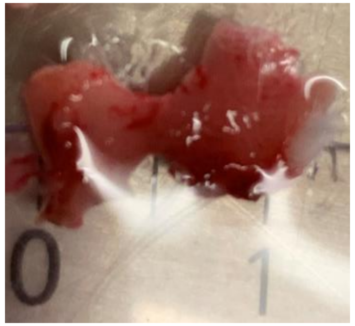
Thymus

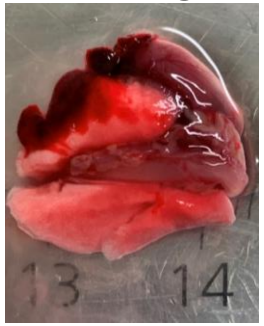
Lungs and heart

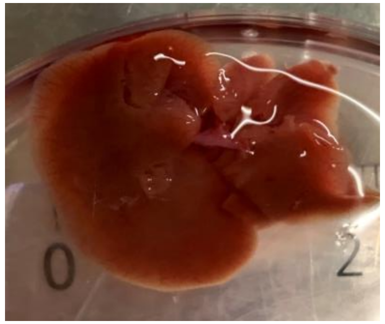
Liver

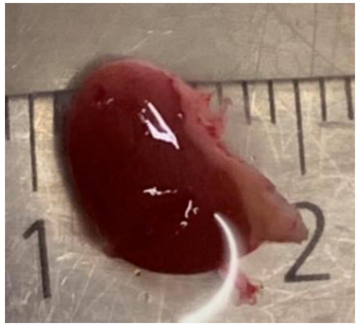
Kidney

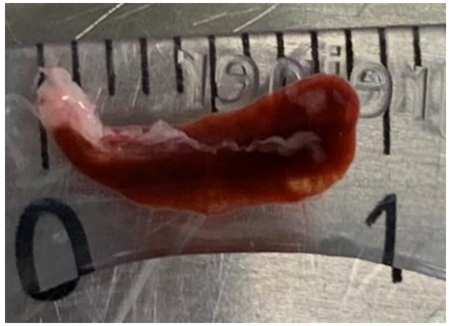
Spleen

Supplementary figure 1-24-4. Score sheets

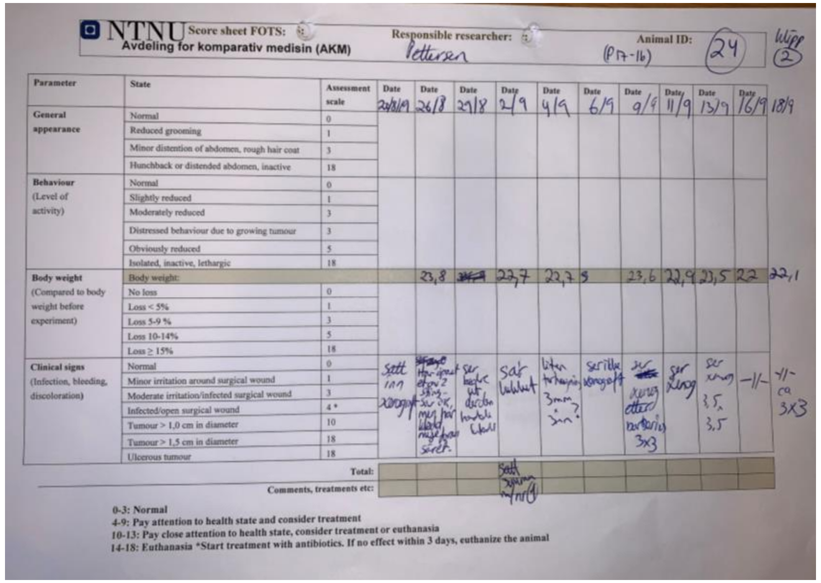

NTNU Score sheet FOTS: Avdeling for komparativ medisin (AKM) — Responsible researcher: Pettersen (P17-16) — Animal ID: 24 — Wipp 2

Parameter	State	Assessment scale
General appearance	Normal	0
	Reduced grooming	1
	Minor distention of abdomen, rough hair coat	3
	Hunchback or distended abdomen, inactive	18
Behaviour (Level of activity)	Normal	0
	Slightly reduced	1
	Moderately reduced	3
	Distressed behaviour due to growing tumour	3
	Obviously reduced	5
	Isolated, inactive, lethargic	18
Body weight (Compared to body weight before experiment)	Body weight:	
	No loss	0
	Loss < 5%	1
	Loss 5-9 %	3
	Loss 10-14%	5
	Loss ≥ 15%	18
Clinical signs (Infection, bleeding, discoloration)	Normal	0
	Minor irritation around surgical wound	1
	Moderate irritation/infected surgical wound	3
	Infected/open surgical wound	4 *
	Tumour > 1,0 cm in diameter	10
	Tumour > 1,5 cm in diameter	18
	Ulcerous tumour	18
	Total:	
	Comments, treatments etc:	

Date	24/8/19	26/8	29/8	2/9	4/9	6/9	9/9	11/9	13/9	16/9	18/9
Body weight		23,8	[illegible]	23,7	22,7	5	23,6	22,9	23,5	22	22,1
Clinical signs	[illegible]	[illegible]	[illegible]	Sår [illegible]	[illegible] 3mm	[illegible]	[illegible] 3x3	[illegible]	3,5 3,5	-//-	-//- ca 3x3

0-3: Normal
4-9: Pay attention to health state and consider treatment
10-13: Pay close attention to health state, consider treatment or euthanasia
14-18: Euthanasia *Start treatment with antibiotics. If no effect within 3 days, euthanize the animal

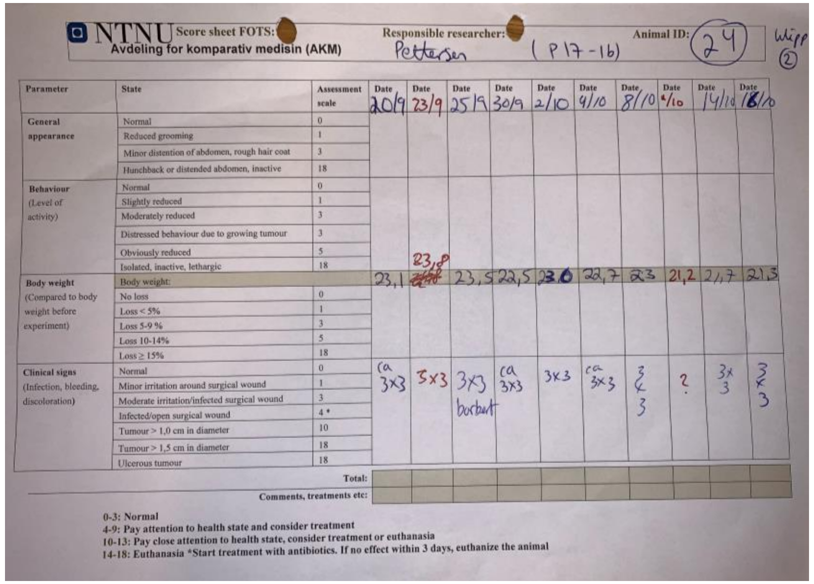

NTNU Score sheet FOTS: Avdeling for komparativ medisin (AKM) — Responsible researcher: Pettersen (P17-16) — Animal ID: 24 — Wipp 2

Date	20/9	23/9	25/9	30/9	2/10	4/10	8/10	11/10	14/10	16/10
Body weight	23,1	23,8	23,5	22,5	23,0	22,7	23	21,2	21,7	21,3
Clinical signs	ca 3x3	3x3	3x3 borbut	ca 3x3	3x3	ca 3x3	3x3	?	3x3	3x3

0-3: Normal
4-9: Pay attention to health state and consider treatment
10-13: Pay close attention to health state, consider treatment or euthanasia
14-18: Euthanasia *Start treatment with antibiotics. If no effect within 3 days, euthanize the animal

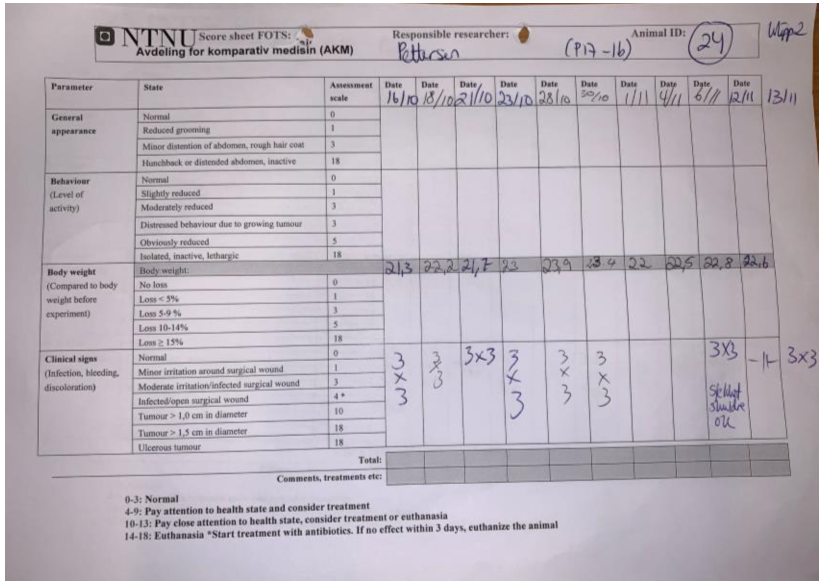

NTNU Score sheet FOTS: Avdeling for komparativ medisin (AKM) — Responsible researcher: Pettersen (P17-16) — Animal ID: 24 — Wipp 2

Date	16/10	18/10	21/10	23/10	28/10	30/10	1/11	4/11	6/11	12/11	13/11
Body weight	21,3	22,2	21,7	23	23,9	23,4	22	22,5	22,8	22,6	
Clinical signs	3x3	3x3	3x3	3x3	3x3	3x3			3x3 [illegible]	-//-	3x3

0-3: Normal
4-9: Pay attention to health state and consider treatment
10-13: Pay close attention to health state, consider treatment or euthanasia
14-18: Euthanasia *Start treatment with antibiotics. If no effect within 3 days, euthanize the animal

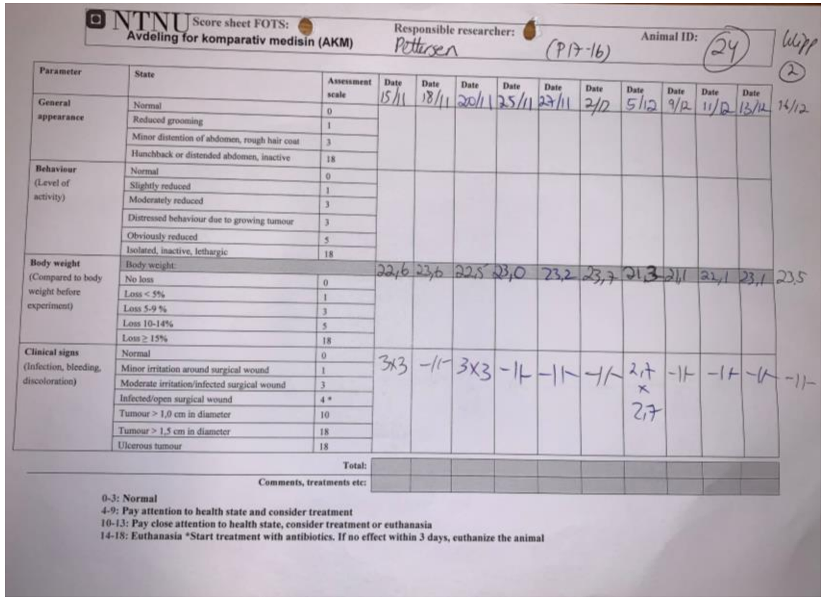

NTNU Score sheet FOTS: Avdeling for komparativ medisin (AKM) — Responsible researcher: Pettersen (P17-16) — Animal ID: 24 — Wipp 2

Date	15/11	18/11	20/11	25/11	27/11	2/12	5/12	9/12	11/12	13/12	16/12
Body weight	22,6	23,6	22,5	23,0	23,2	23,7	21,3	24,1	22,1	23,1	23,5
Clinical signs	3x3	-//-	3x3	-//-	-//-	-//-	2,7 x 2,7	-//-	-//-	-//-	-//-

0-3: Normal
4-9: Pay attention to health state and consider treatment
10-13: Pay close attention to health state, consider treatment or euthanasia
14-18: Euthanasia *Start treatment with antibiotics. If no effect within 3 days, euthanize the animal

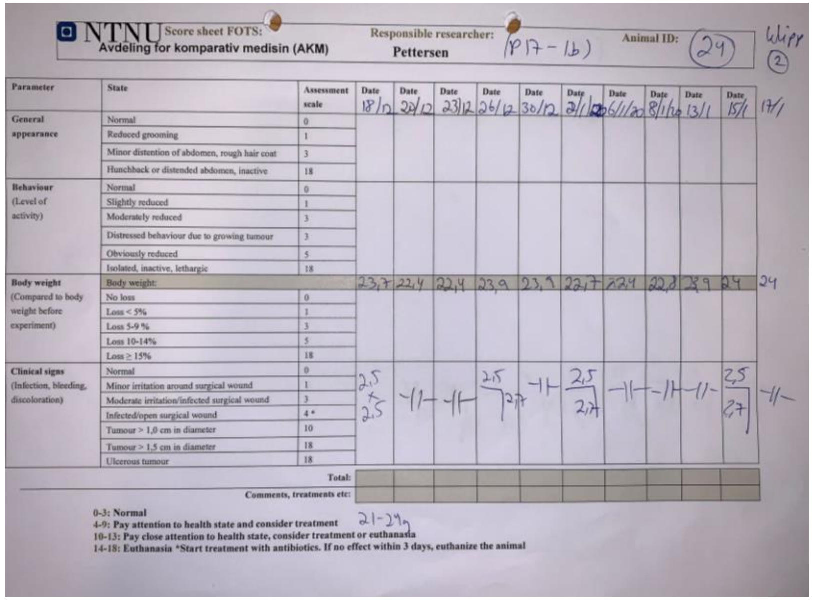

NTNU Score sheet FOTS: Avdeling for komparativ medisin (AKM) — Responsible researcher: Pettersen (P17-16) — Animal ID: 24 — Wipp 2

Date	18/12	20/12	23/12	26/12	30/12	2/1	6/1	8/1	13/1	15/1	17/1
Body weight	23,7	22,4	22,4	23,9	23,9	22,7	22,4	22,8	23,9	24	24
Clinical signs	2,5 x 2,5	-//-	-//-	2,5 2,7	-//-	2,5 2,7	-//-	-//-	-//-	2,5 2,7	-//-

0-3: Normal
4-9: Pay attention to health state and consider treatment 21-24g
10-13: Pay close attention to health state, consider treatment or euthanasia
14-18: Euthanasia *Start treatment with antibiotics. If no effect within 3 days, euthanize the animal

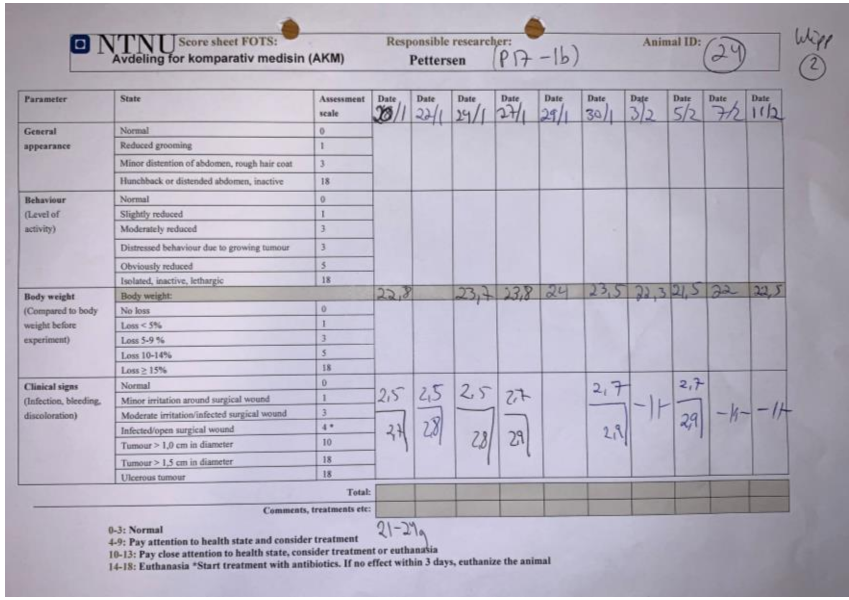

NTNU Score sheet FOTS: Avdeling for komparativ medisin (AKM) — Responsible researcher: Pettersen (P17-16) — Animal ID: 24 — Wipp 2

Date	20/1	22/1	24/1	27/1	29/1	30/1	3/2	5/2	7/2	11/2
Body weight	22,8		23,7	23,8	24	23,5	22,3	21,5	22	22,5
Clinical signs	2,5 2,7	2,5 2,8	2,5 2,8	2,7 2,9		2,7 2,9	-//-	2,7 2,9	-//-	-//-

0-3: Normal 21-24g
4-9: Pay attention to health state and consider treatment
10-13: Pay close attention to health state, consider treatment or euthanasia
14-18: Euthanasia *Start treatment with antibiotics. If no effect within 3 days, euthanize the animal

Supplementary figure 1-24-4. Score sheets

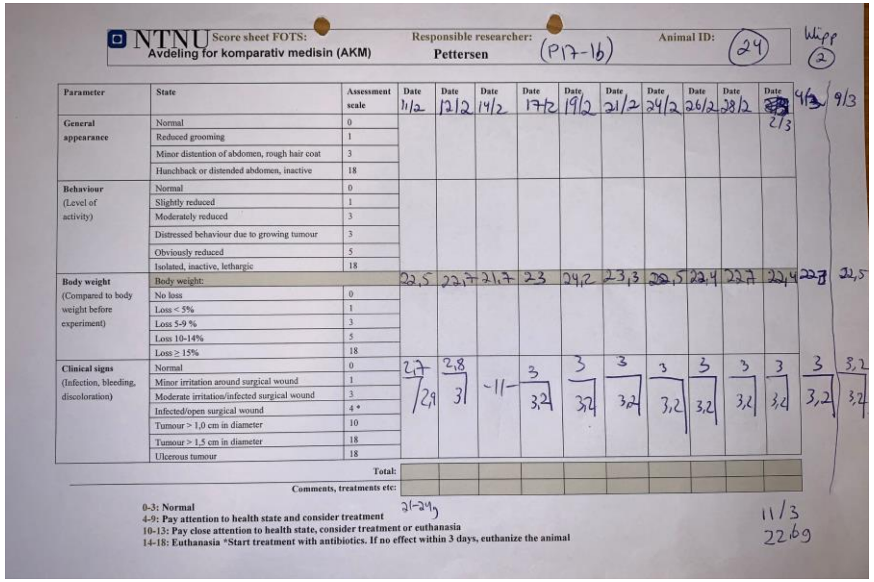

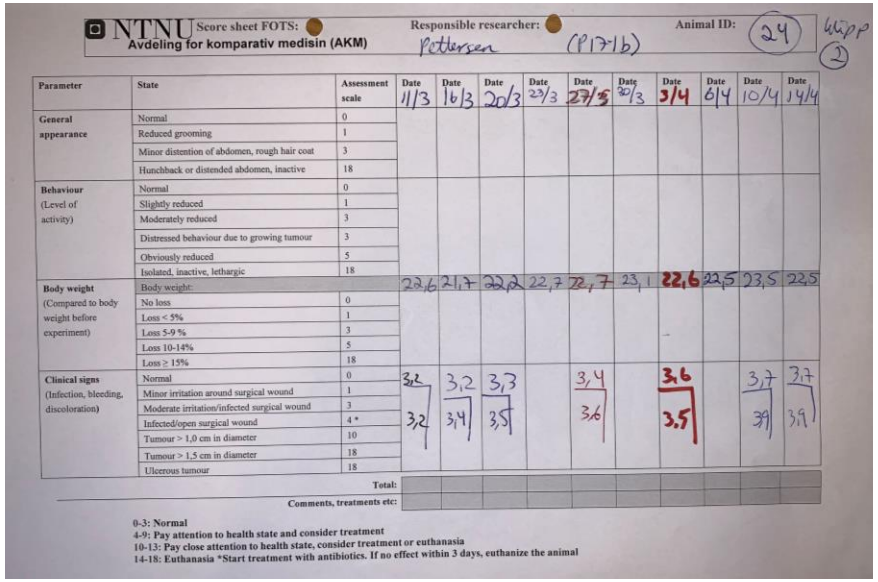

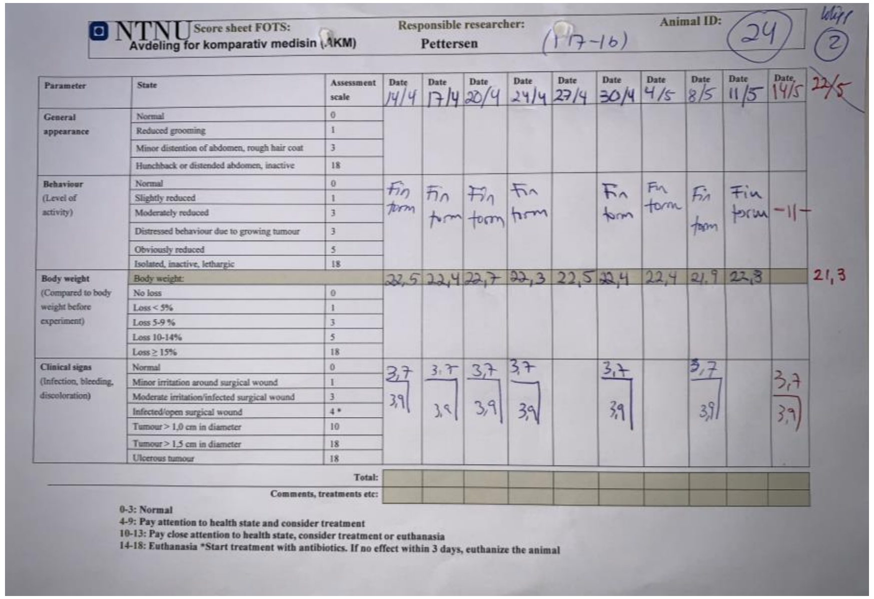

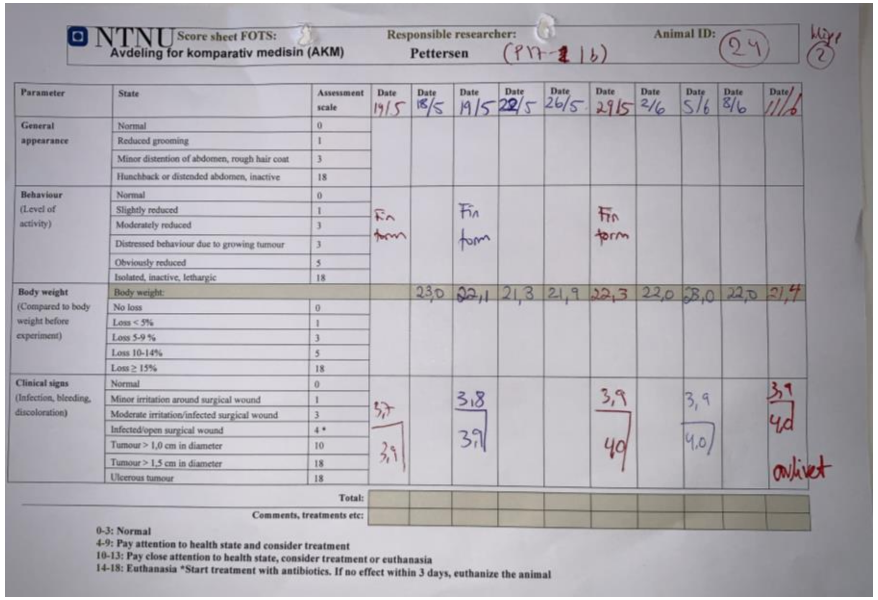

Supplementary figure 1-24-5. Mouse and xenograft growth curves

Weight curve

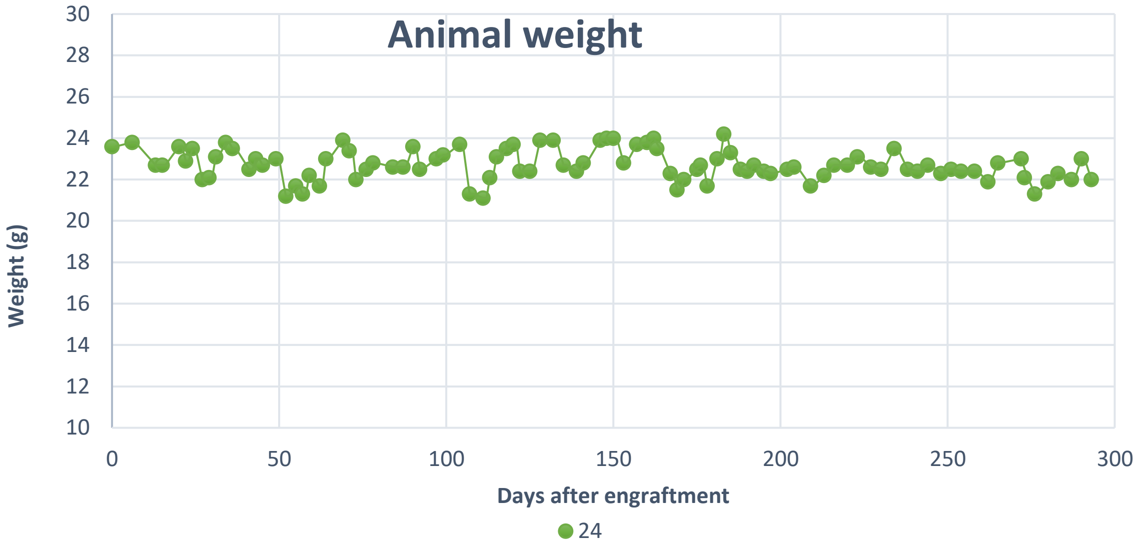

Xenograft growth curve

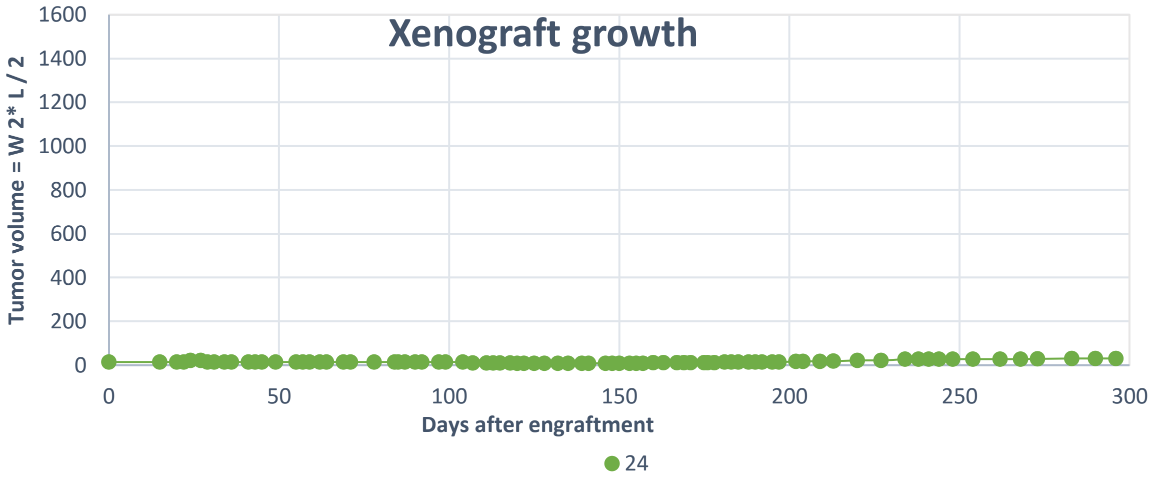

Supplementary figure 1-24-6. Histology pictures

M24X – Xenograft

- Typical colorectal adenocarcinoma
- Glandulas with necrotic debris in lumen
- Mucus producing adenocarcinoma
- Necotic tissue in the middle

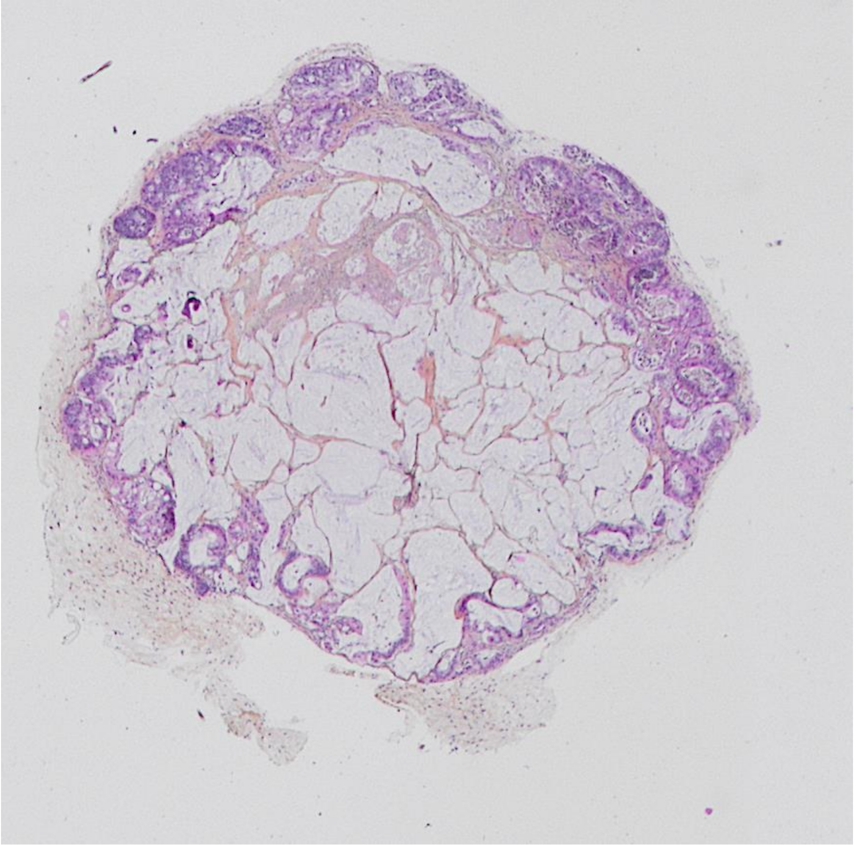

M24LH – Lungs and heart

- Looks normal

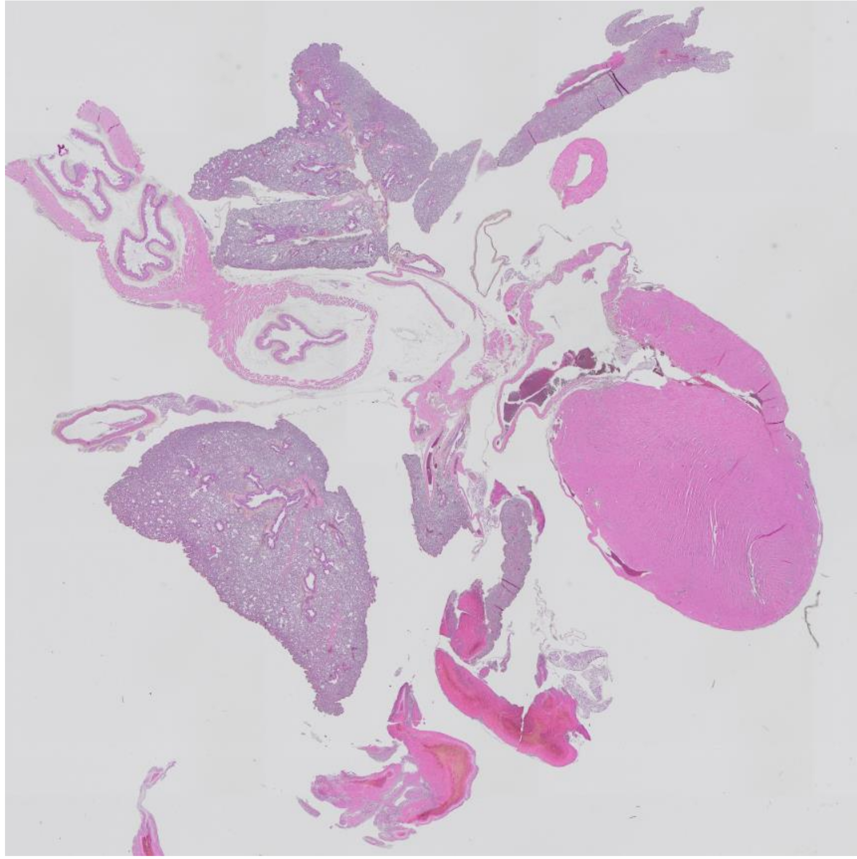

Supplementary figure 1-24-6. Histology pictures

M24MP – Spleen and pancreas
- Spleen looks normal
- Pancreas looks normal

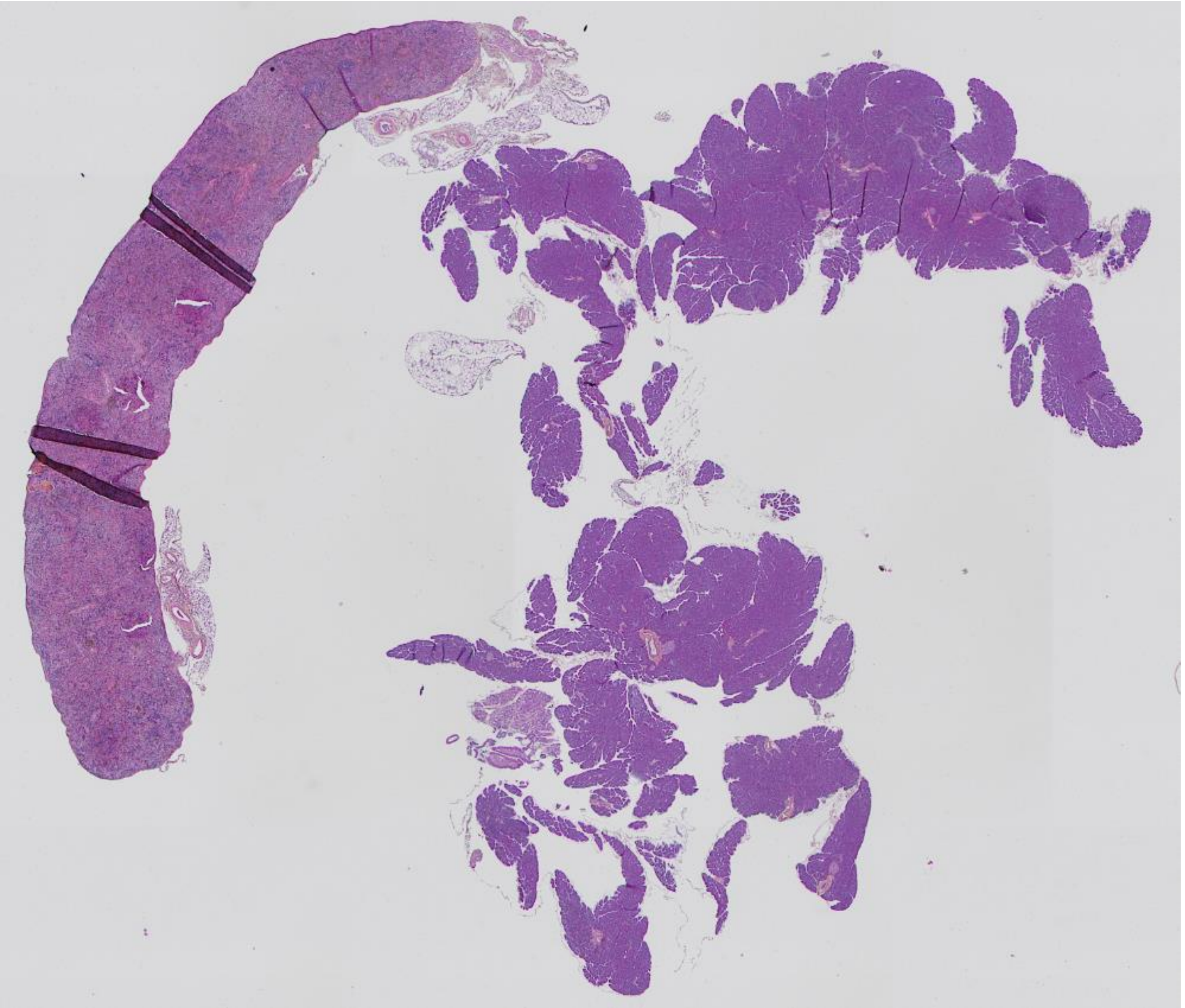

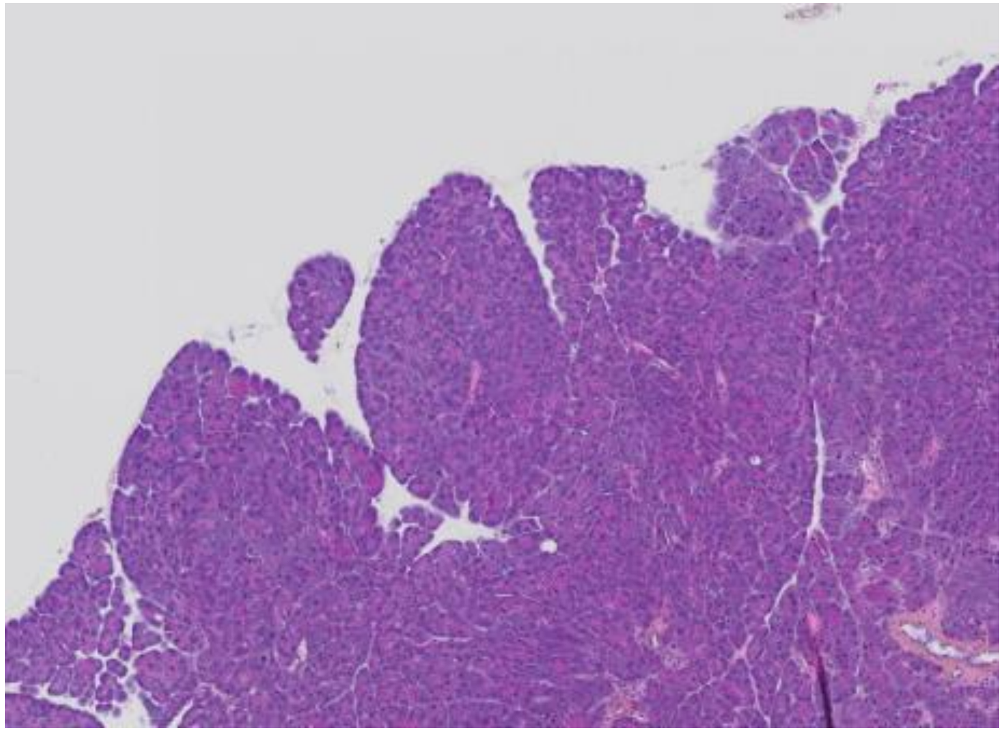

Mouse 25

- Surgery date: 20.08.19
  - Age (weeks) 31
  - Weight (g) 24.6
  - Tumor fragment P17-2A
  - Fragment size 3 x 3 mm
  - Ear mark 3

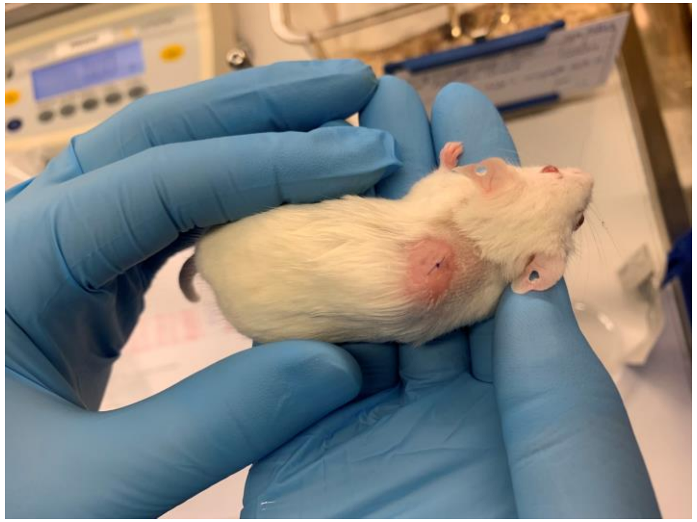

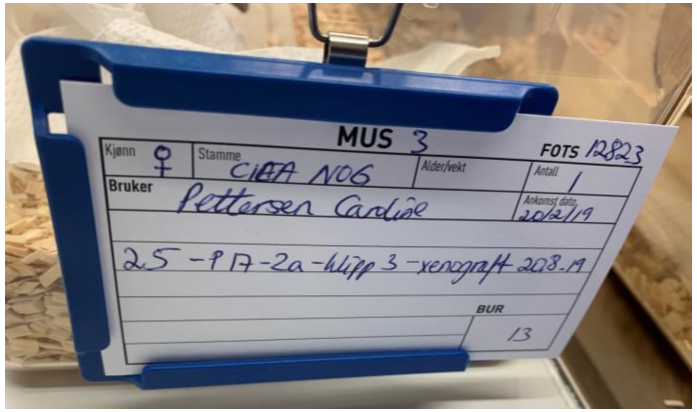

Supplementary figure 1-25-1.
Pictures from the engraftment day

Supplementary figure 1-25-2. Pictures from follow up and xenograft size measurement

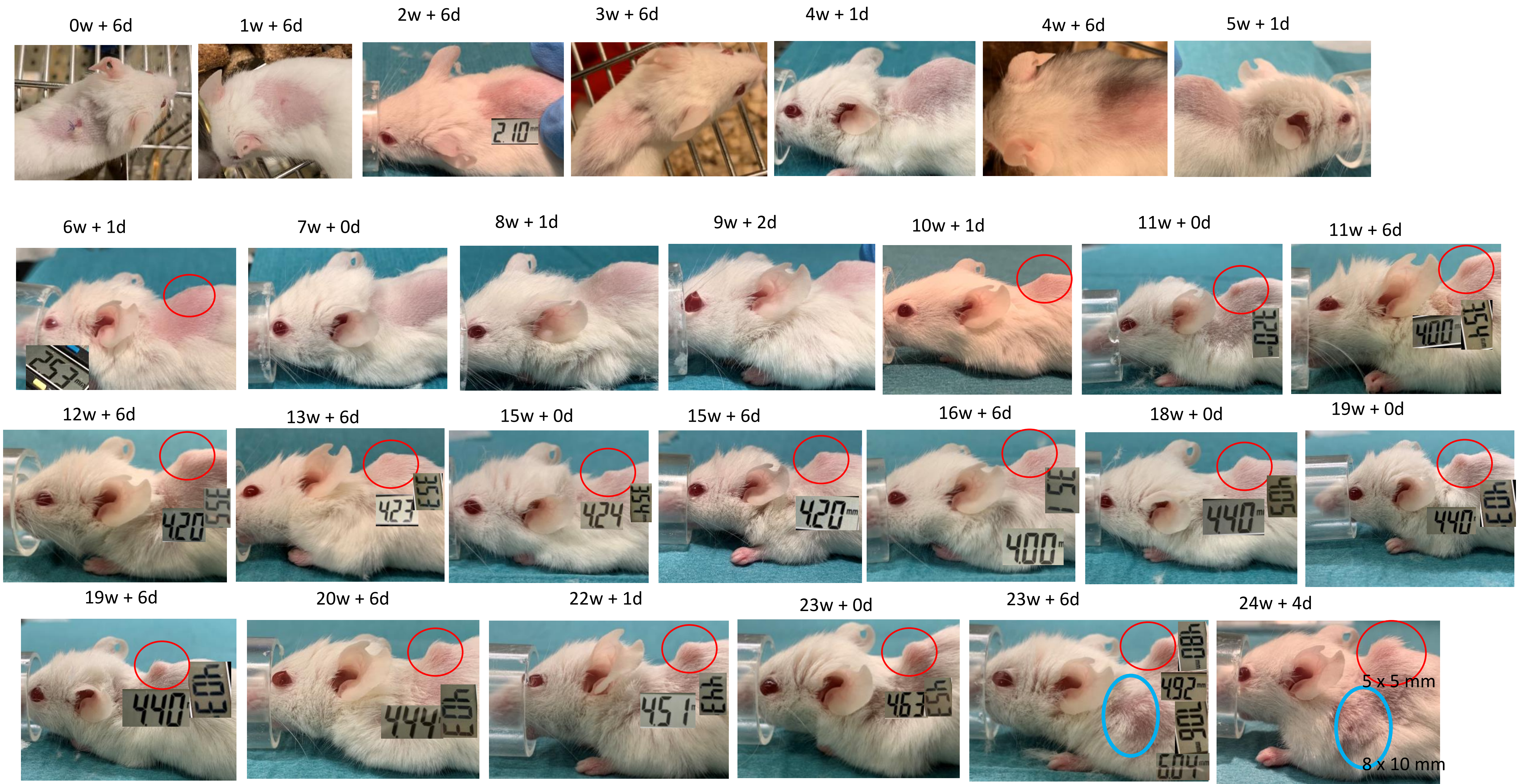

Supplementary figure 1-25-3. Picture from follow up

- Thick skin and a wound on the right shoulder at day 82, 11w + 5d (12.11.19)
- Looked bether 15.11.19
- Shaving machine checked for any defaults

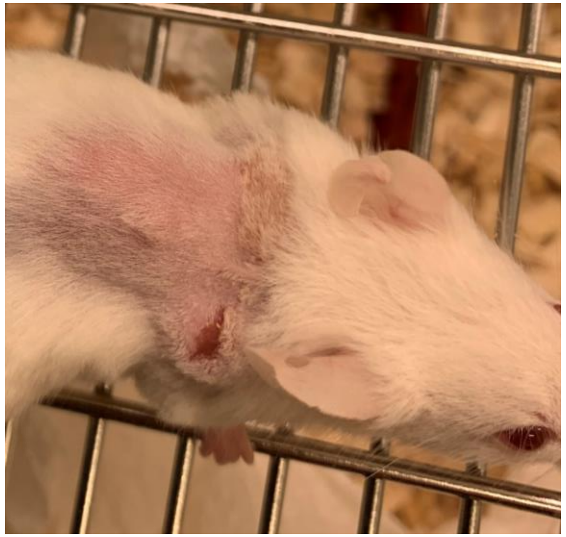

Supplementary figure 1-25-3. Pictures from euthanization 10.02.20

- Reduced general health
- Rapidly growing secondary tumor on the left shoulder
- Necroscopy
  - Xenograft size; 5 x 5 mm
  - Treatment not started due to reduced health
  - Secondary tumor at left shoulder 10 x 8 mm
  - Colon taken out and washed with sterile NaCl
    - To be used for testing antibodies for IHC
  - Blood sample
  - Tumor tissue for
    - Histology

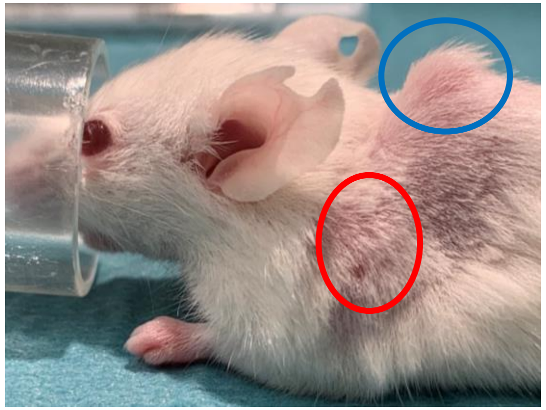

Blue; xenograft
Red; secondary tumor

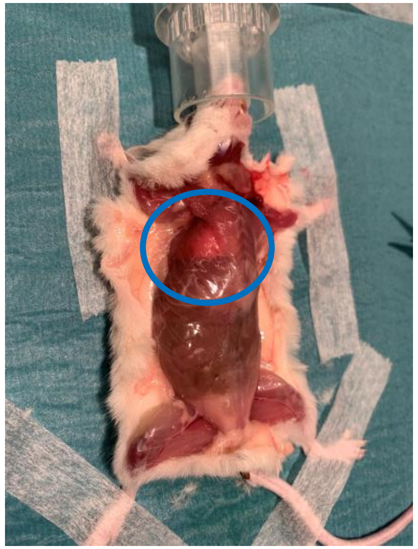

Whiteish colour on the lungs

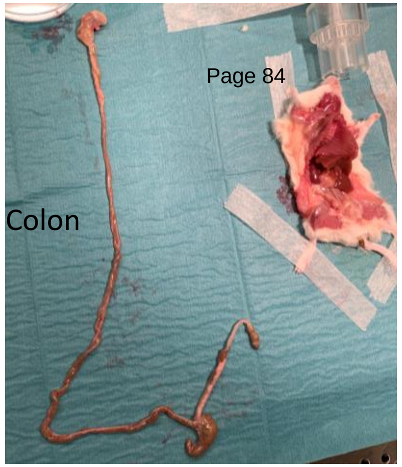

Colon taken out to use for antibody testing for IHC. Flushed with sterile NaCl.

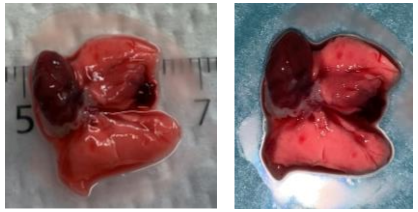

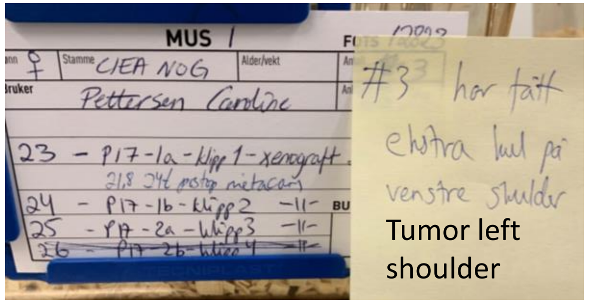

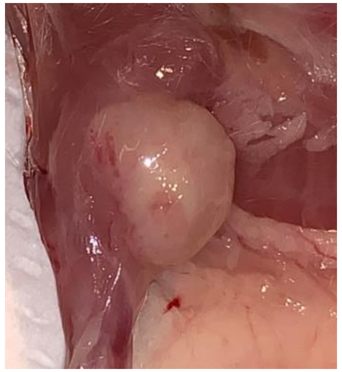

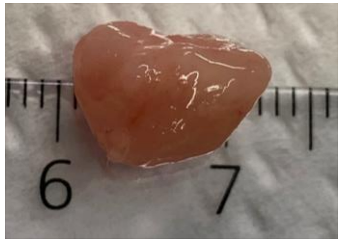

Tumor left shoulder

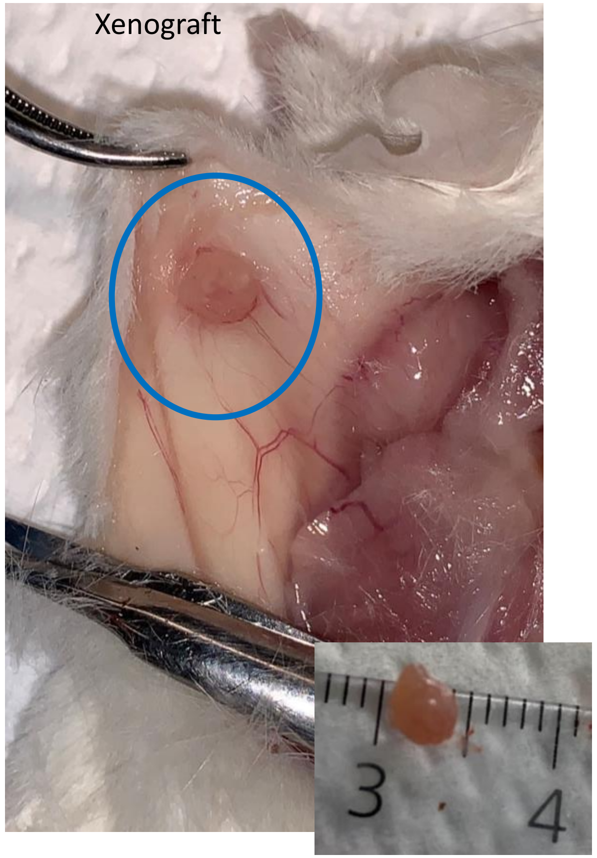

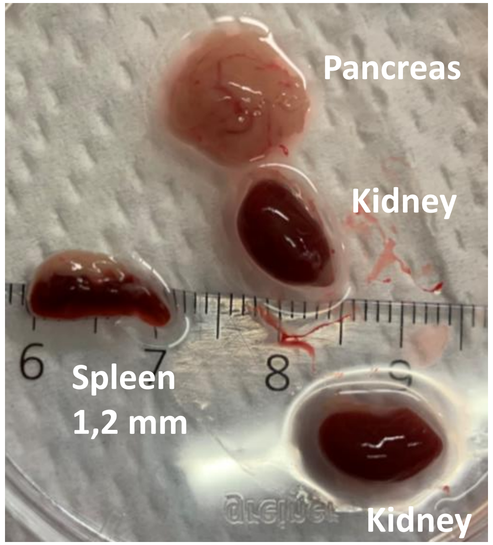

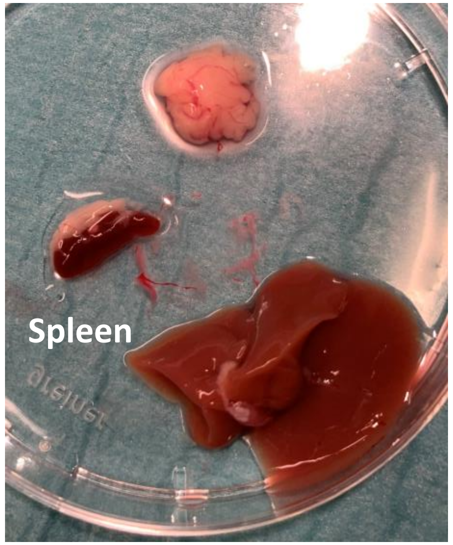

Supplementary figure 1-25-4. Score sheets

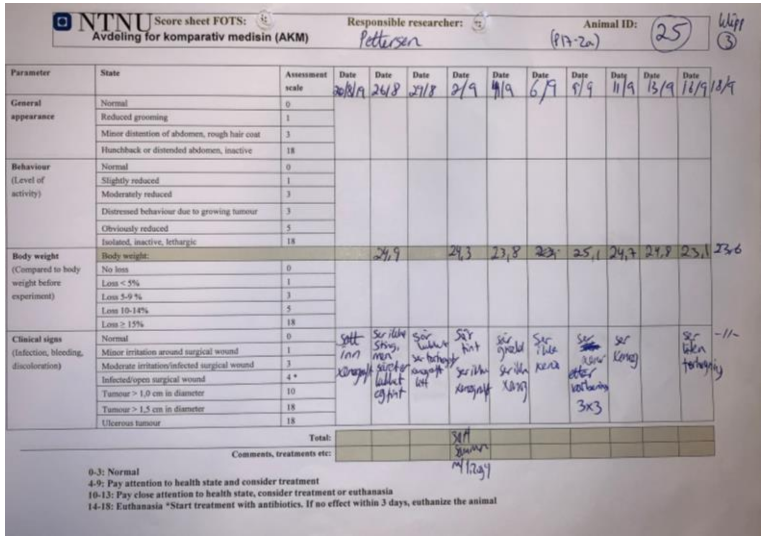

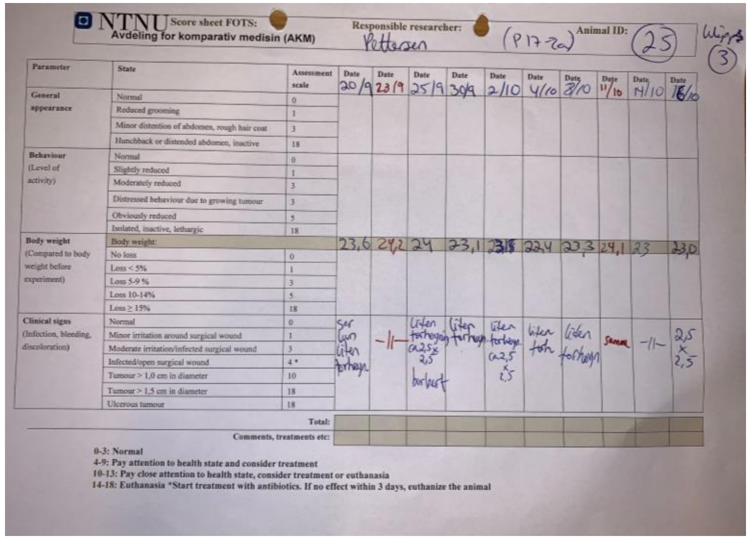

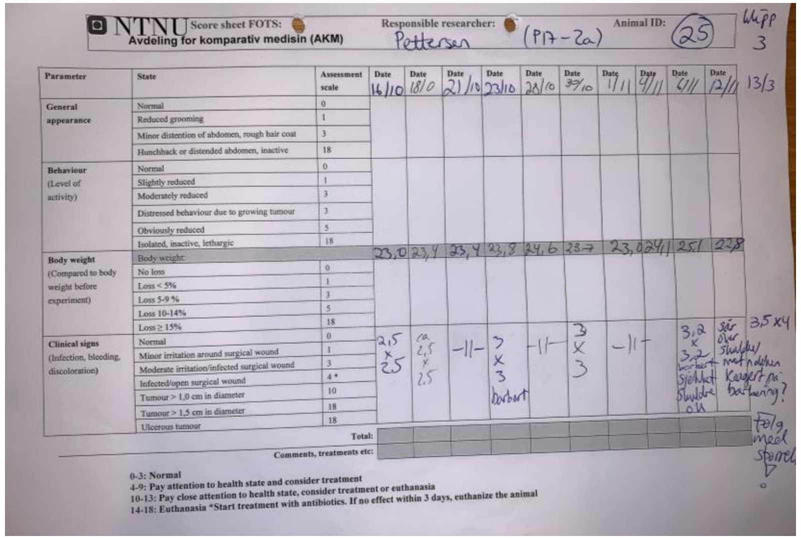

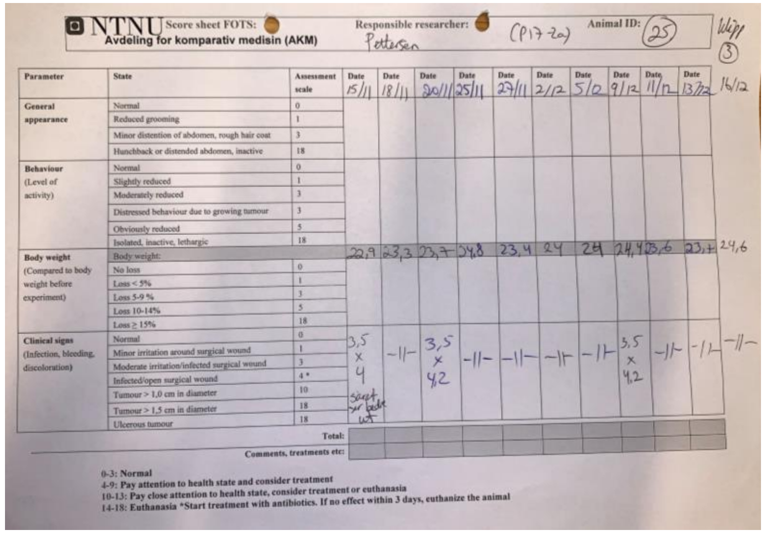

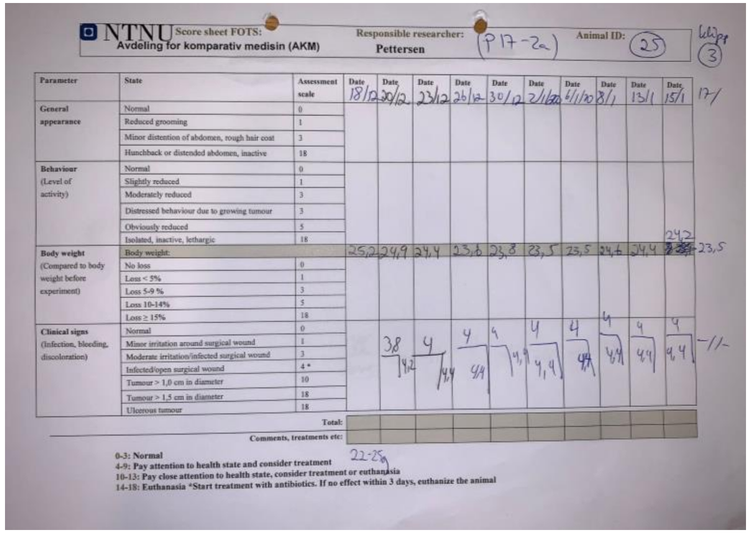

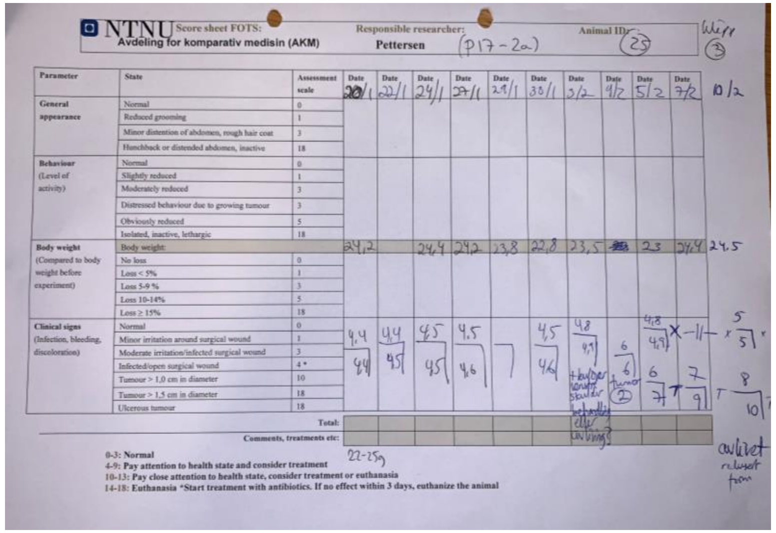

Supplementary figure 1-25-5. Mouse, xenograft and secondary tumor growrh curves

Weight curve

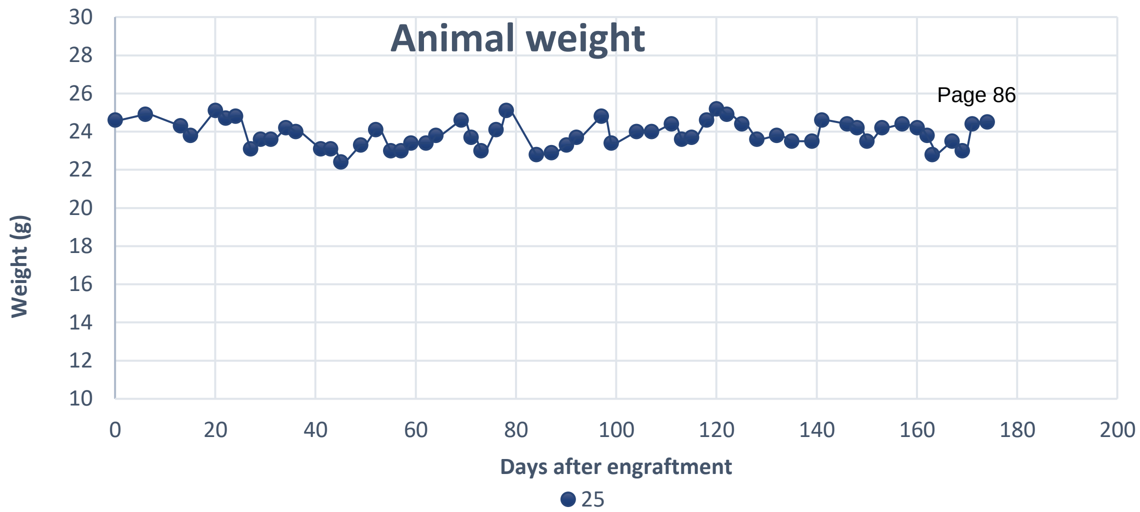

Xenograft growth curve

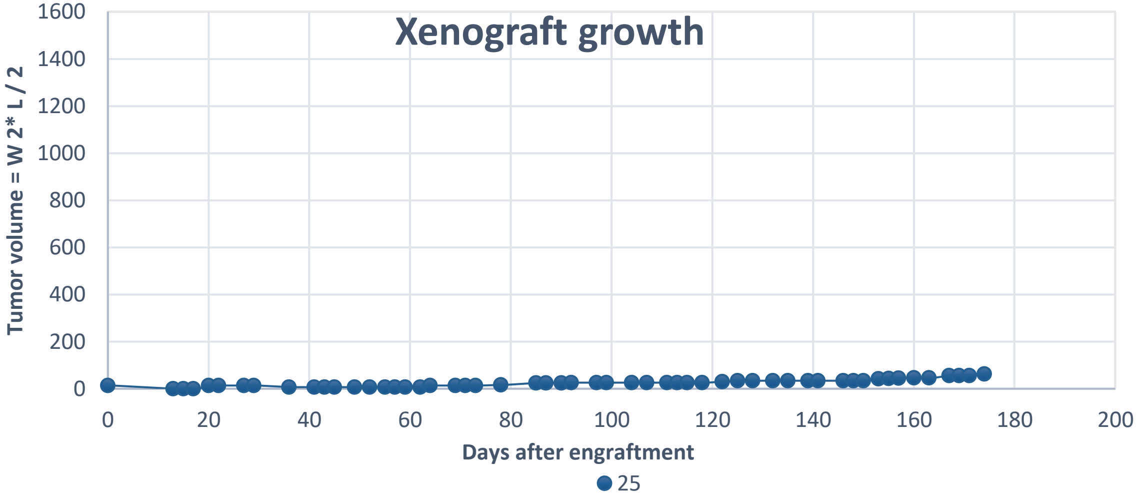

Secondary tumor growth curve

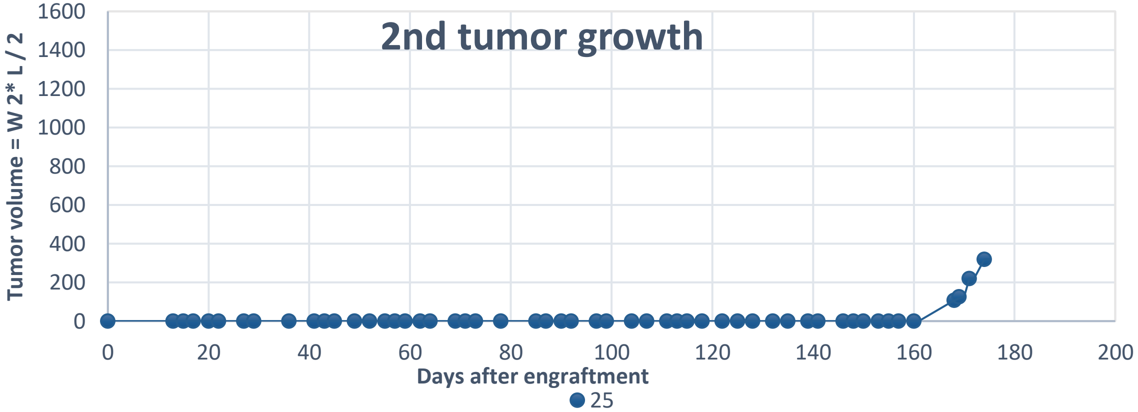

Supplementary figure 1-25-6. Histology pictures

M25X

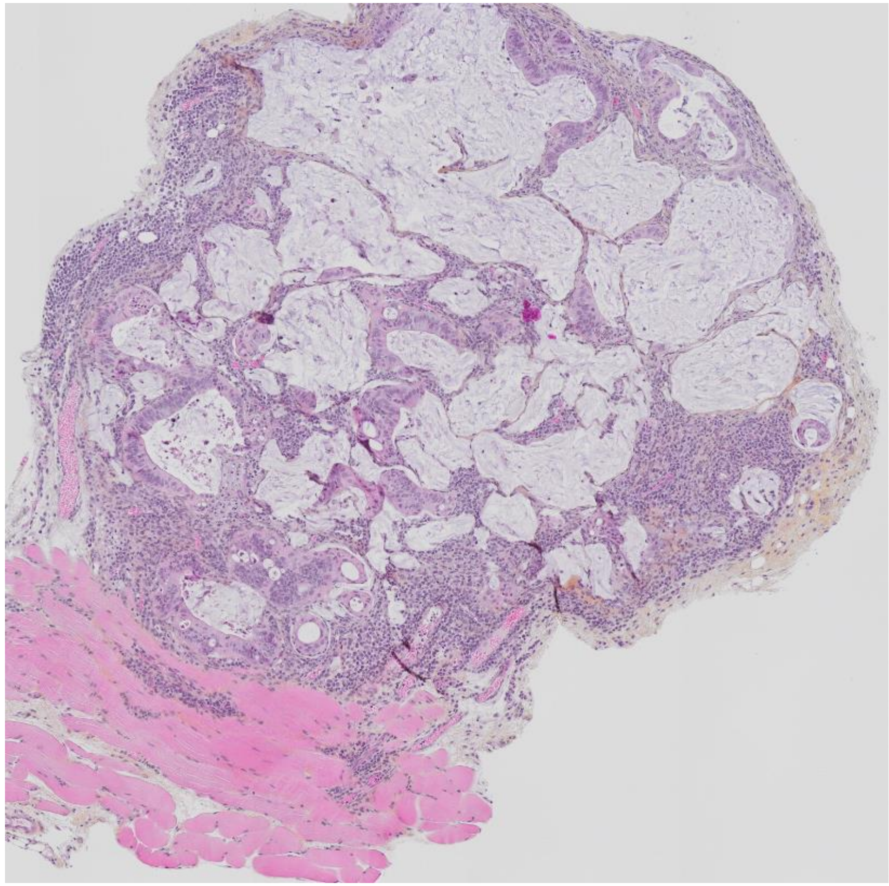

- Typical colorectal cancer tissue
- Aereas with glandulas
- Lymphoid cells at the border of the xenograft
- Mucus producing adenocarcinoma
- Unregular nuclear membranes might indicate stressed cells
  - Could be that the lymphoid cells are about taking over the xenograft

M25T

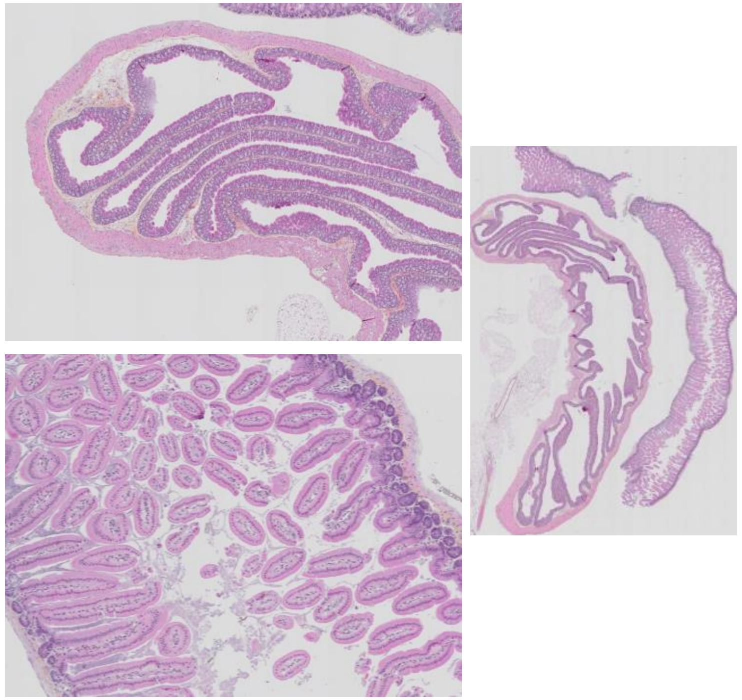

- Colon tissue; looks healthy
  - To be used for antibody testing

M25P

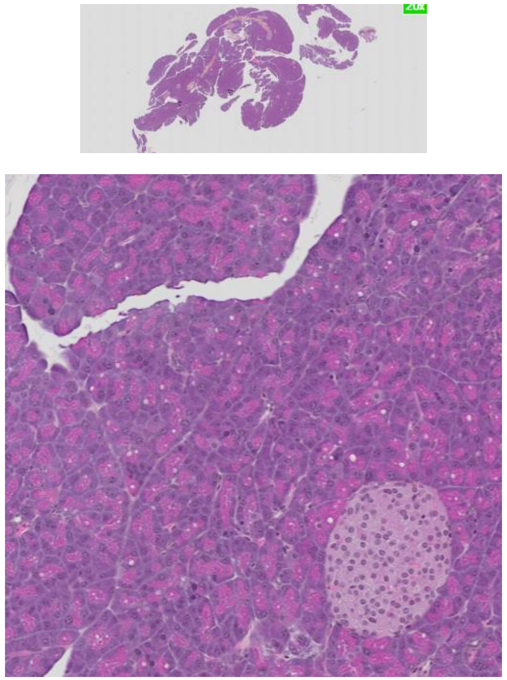

- Pancreas

Supplementary figure 1-25-6. Histology pictures

M25TV (tumor left shoulder)

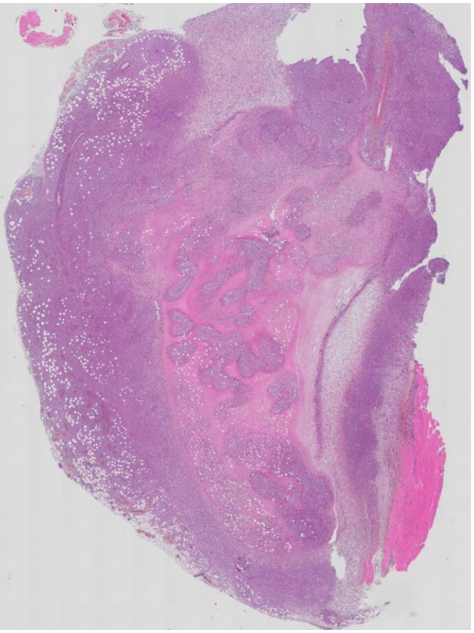

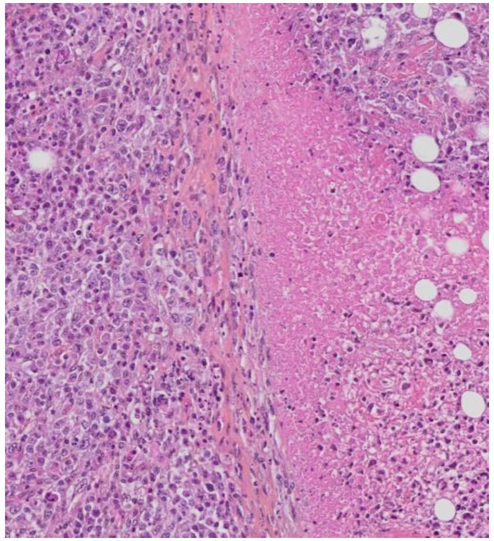

- Massive tumor
- Lymphoid cells
- Large light cells with spotted nuclei

M25MP (spleen and pancreas)

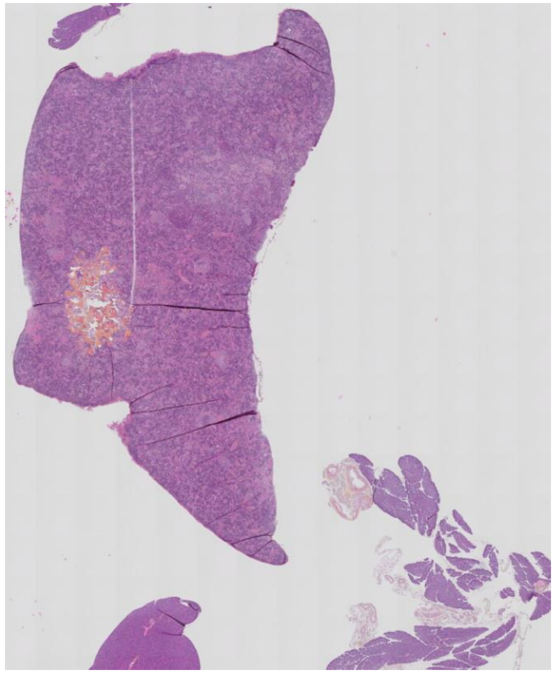

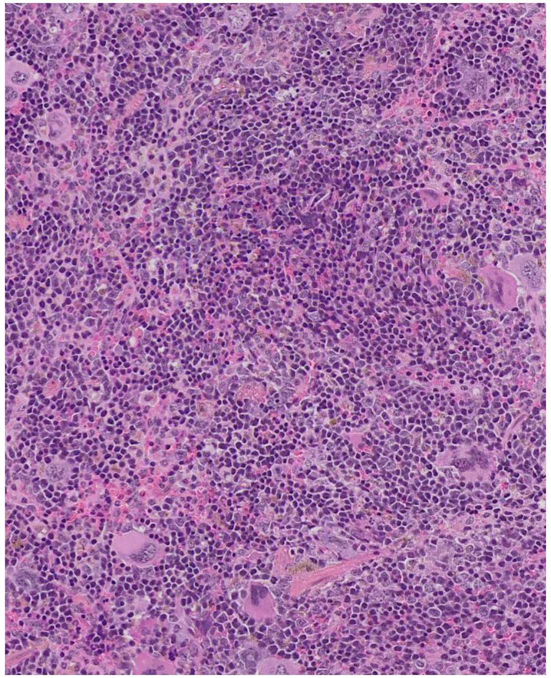

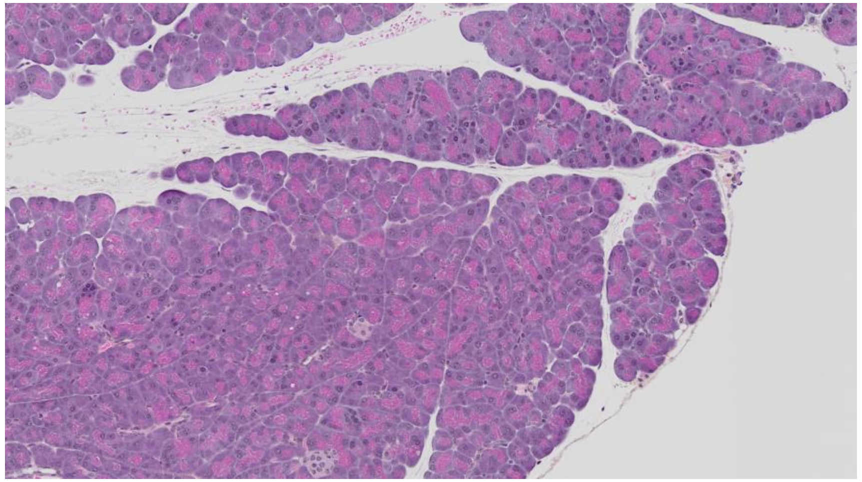

- Spleen with dark defined nuclei and megakaryocytes and red blodspots/vains
- Pancreas looks normal

Supplementary figure 1-25-6. Histology pictures

M25LH (Lungs and heart)

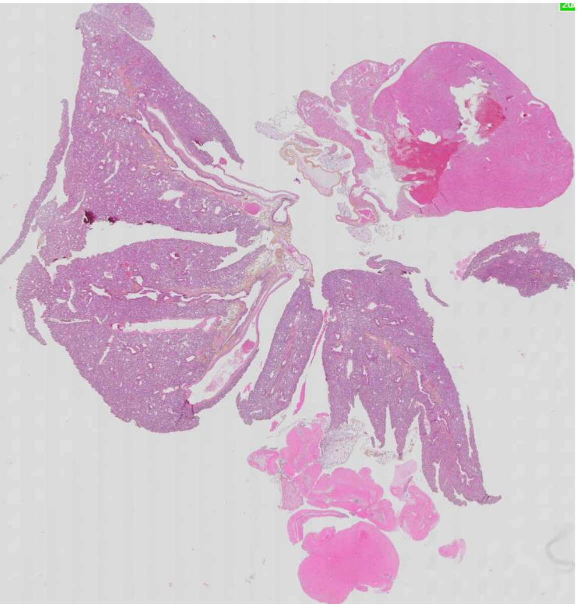

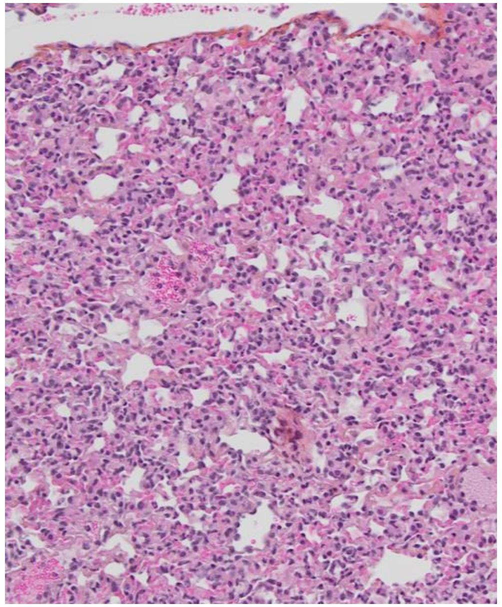

- Lungs looks collapsed, might be from the preservation, fill lungs with formalin hereafter
- Lungs look healthy but cell dense

Supplementary figure 1-25-7. Immunohistochemistry and EBV RNA staining

M25TV (tumor left shoulder) - lymphoma

- IHC staining of Ki67, and CD45
- EBER-ISH staining to detect EBV

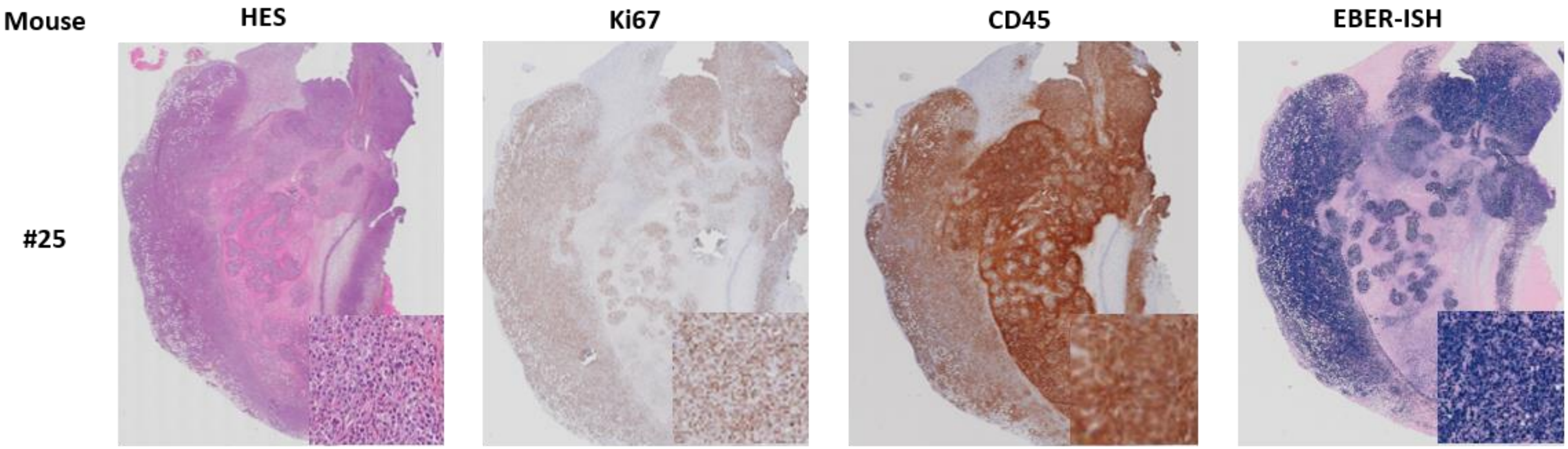

Mouse 26

- Surgery date: 20.08.19
  - Age (weeks) 31
  - Weight (g) 24.4
  - Tumor fragment P17-2B
  - Fragment size 3 x 3 mm
  - Ear mark 4

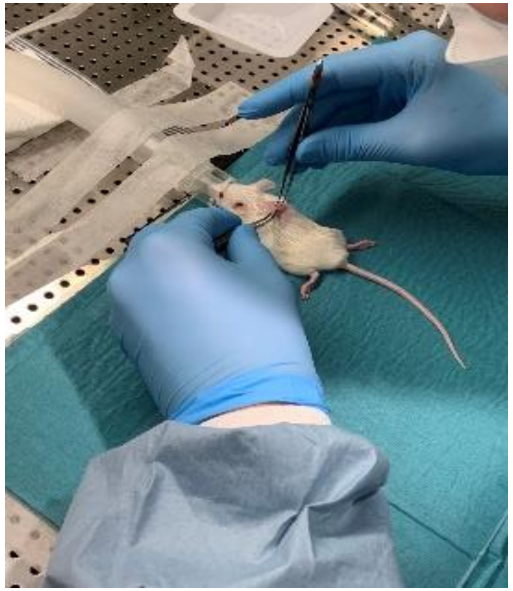
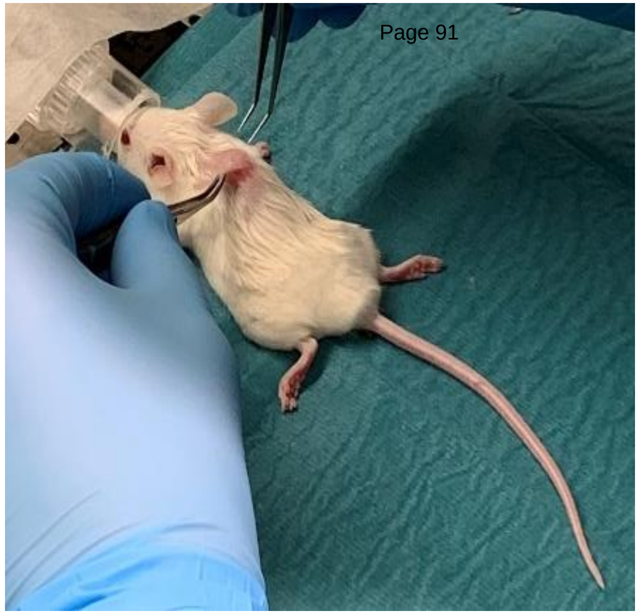
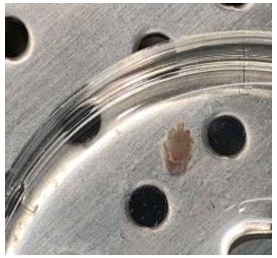
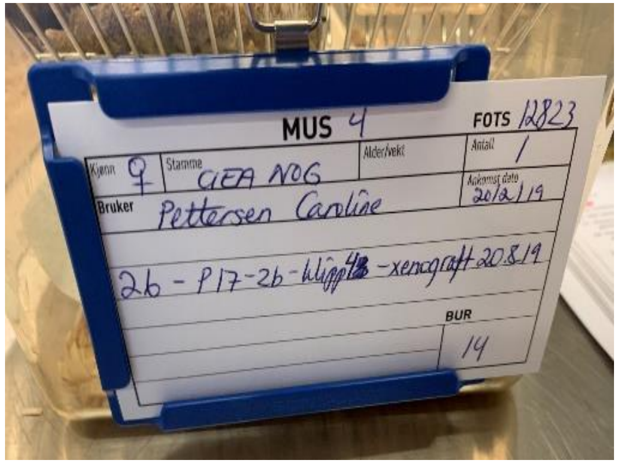

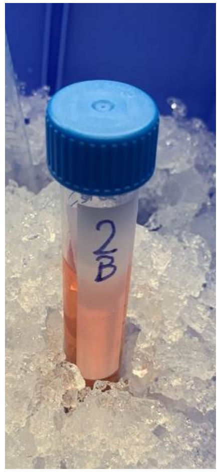

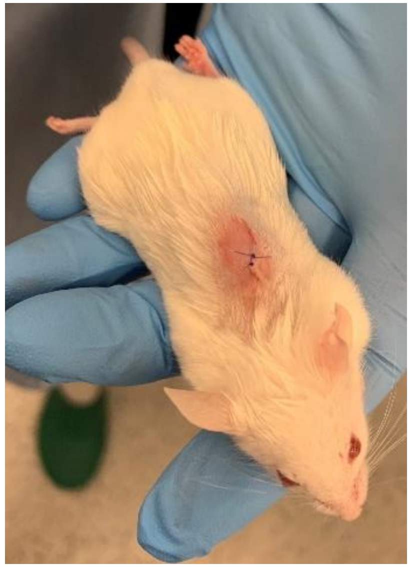
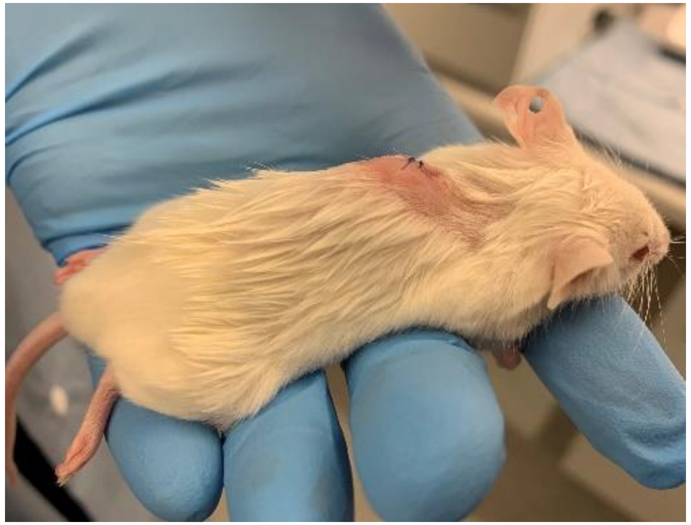

Supplementary figure 1-26-1. Pictures from the engraftment day

Supplementary figure 1-26-2. Pictures from follow up and tumor size measurement

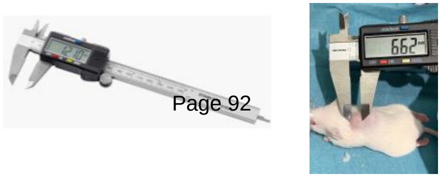

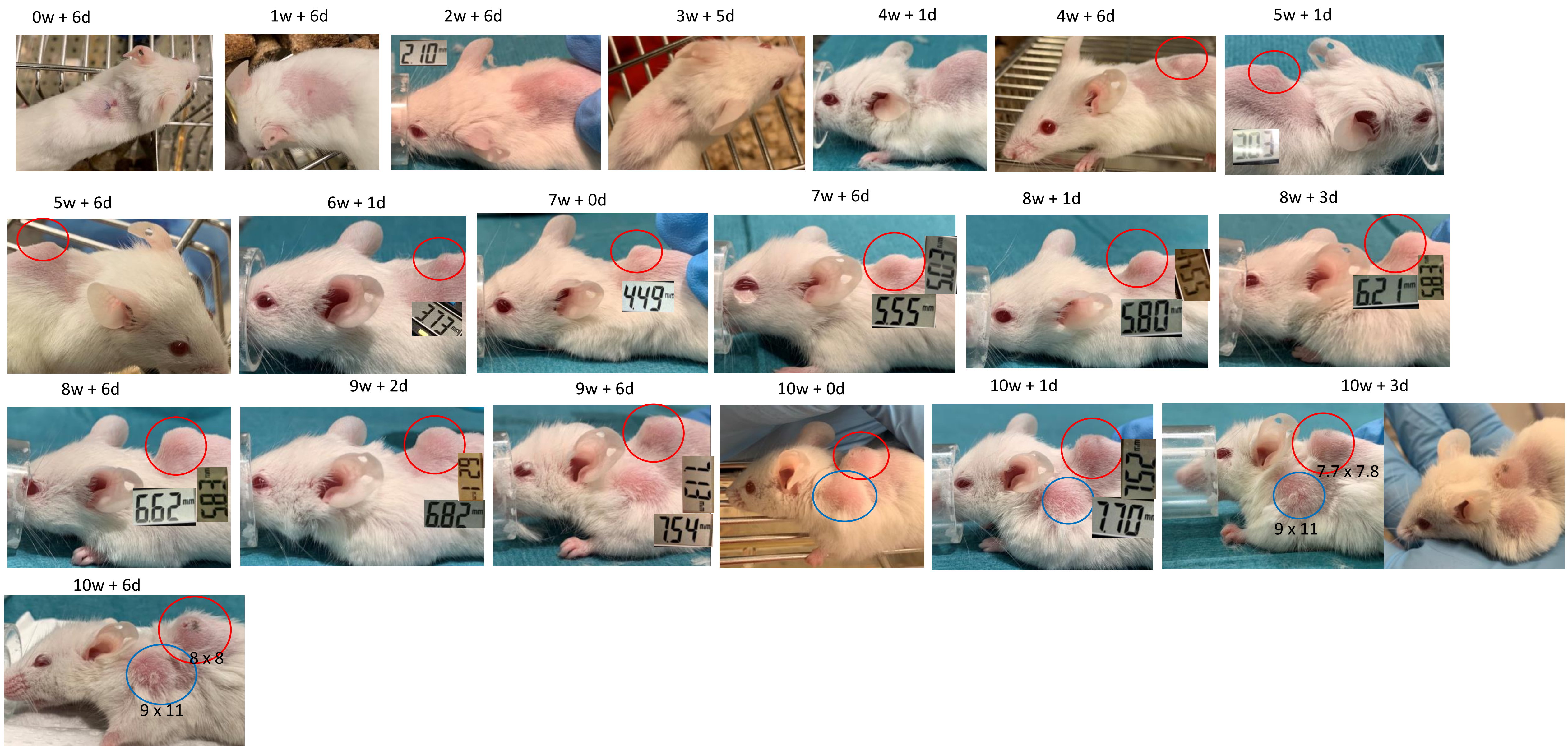

Treatment:

- Smartfish Remune with fish oil
- Days/weeks
- Blood samples before/after treatment

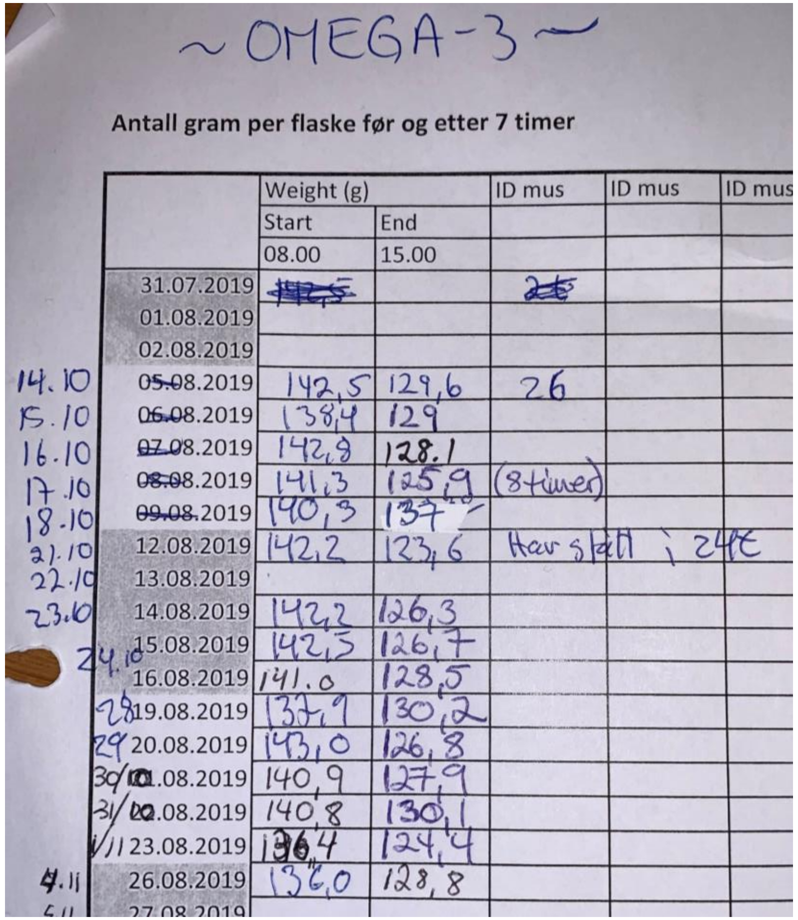

~ OMEGA-3 ~

Antall gram per flaske før og etter 7 timer

		Weight (g) Start 08.00	End 15.00	ID mus	ID mus	ID mus
	31.07.2019	~~142,5~~		~~25~~		
	01.08.2019					
	02.08.2019					
14.10	~~05.~~08.2019	142,5	129,6	26		
15.10	~~06.~~08.2019	138,4	129			
16.10	~~07.~~08.2019	142,8	128.1			
17.10	~~08.~~08.2019	141,3	125,9	(8 timer)		
18.10	~~09.08.~~2019	140,3	137			
21.10	12.08.2019	142,2	123,6	Har [illegible] i 24t		
22.10	13.08.2019					
23.10	14.08.2019	142,2	126,3			
	15.08.2019	142,5	126,7			
24.10	16.08.2019	141.0	128,5			
28	19.08.2019	137,9	130,2			
29	20.08.2019	143,0	126,8			
30/10	21.08.2019	140,9	127,9			
31/10	22.08.2019	140,8	130,1			
1/11	23.08.2019	136,4	124,4			
4.11	26.08.2019	136,0	128,8			
[illegible]	27.08.2019					

Supplementary figure 1-26-3. List over weight registrations of drinking bottle (with Smartfish Remune Peach) before and after treatment

Supplementary figure 1-26-4.

Pictures from euthanization 04.11.19

- Necroscopy
  - Xenograft size
  - Secondary tumor left shoulder
  - Secondary tumor right shoulder
  - Blood sample
  - Tumor tissue for
    - Histology
    - RNA isolation
    - Protein isolation

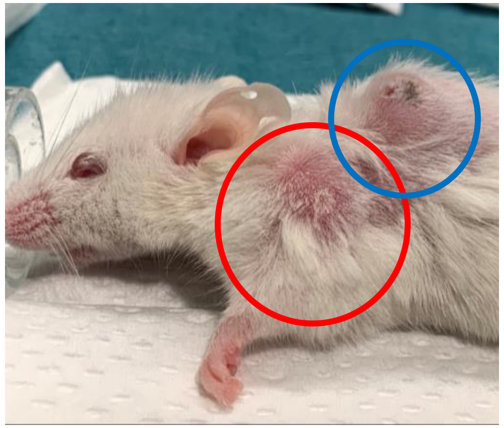
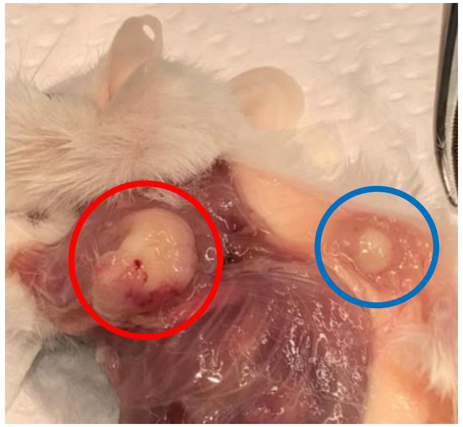
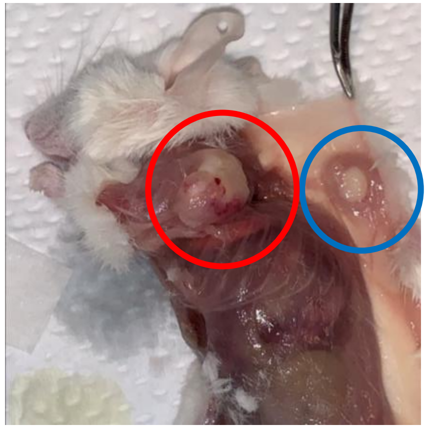
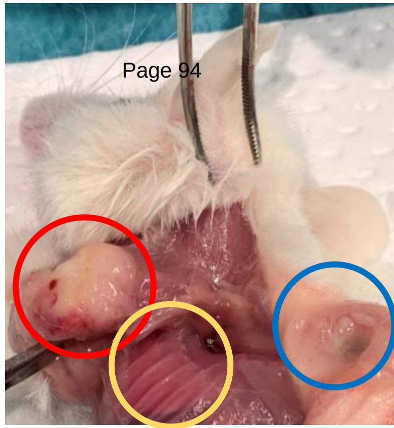

Blue; xenograft, Red; 2nd tumor

Whitish lungs

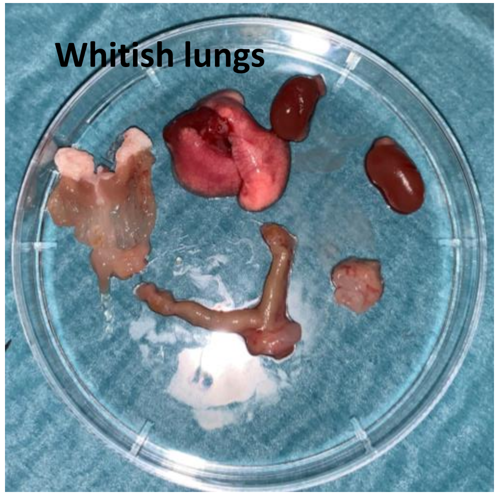

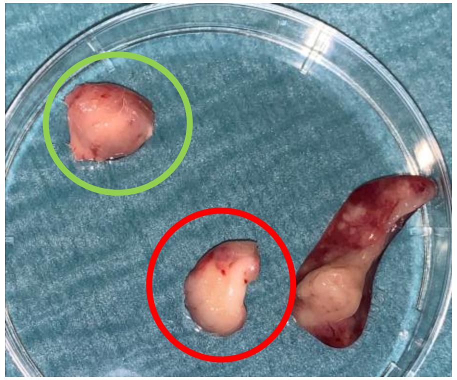

Enlarged spleen (2.2 cm) with white areas. Green; secondary tumor (right shoulder), Red; secondary tumor (left shoulder)

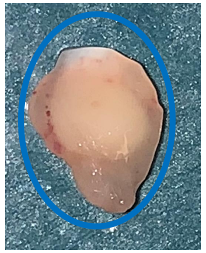

Xenograft

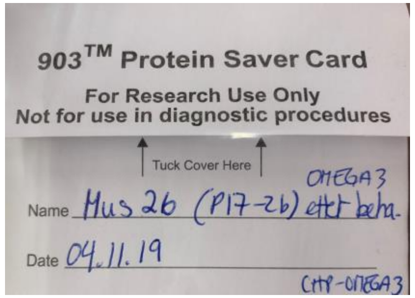

Bloodcard for fatty acid profiling

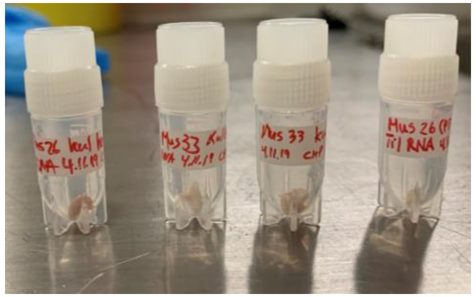

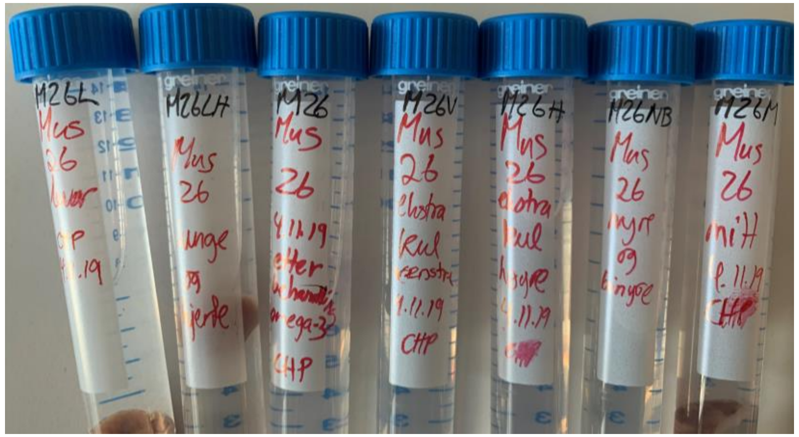

Samples collected for histology/HES staining

Supplementary figure 1-26-5. Score sheets

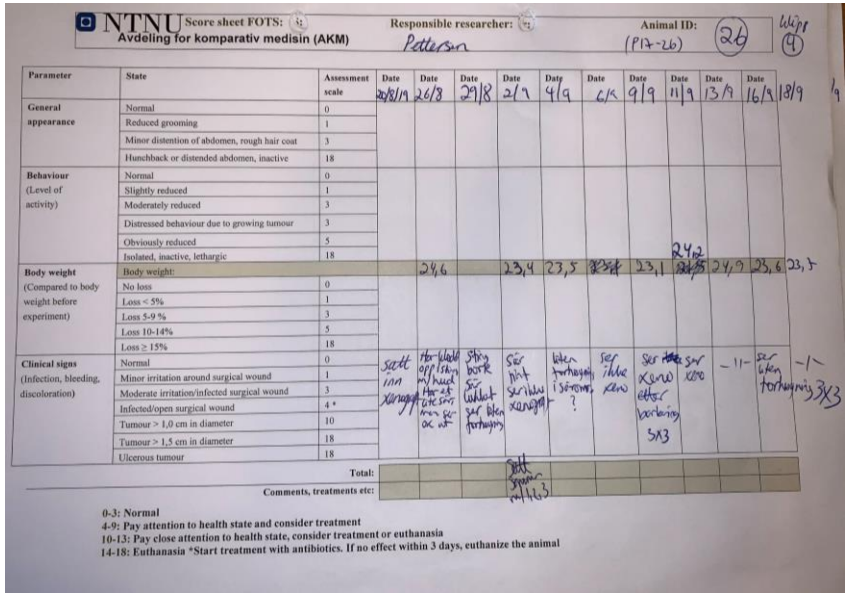
NTNU Score sheet FOTS: Avdeling for komparativ medisin (AKM)
Responsible researcher: Pettersen (P17-26)
Animal ID: 26

Parameter	State	Assessment scale
General appearance	Normal	0
	Reduced grooming	1
	Minor distention of abdomen, rough hair coat	3
	Hunchback or distended abdomen, inactive	18
Behaviour (Level of activity)	Normal	0
	Slightly reduced	1
	Moderately reduced	3
	Distressed behaviour due to growing tumour	3
	Obviously reduced	5
	Isolated, inactive, lethargic	18
Body weight (Compared to body weight before experiment)	Body weight:	
	No loss	0
	Loss < 5%	1
	Loss 5-9 %	3
	Loss 10-14%	5
	Loss ≥ 15%	18
Clinical signs (Infection, bleeding, discoloration)	Normal	0
	Minor irritation around surgical wound	1
	Moderate irritation/infected surgical wound	3
	Infected/open surgical wound	4 *
	Tumour > 1,0 cm in diameter	10
	Tumour > 1,5 cm in diameter	18
	Ulcerous tumour	18
Total:		
Comments, treatments etc:		

0-3: Normal
4-9: Pay attention to health state and consider treatment
10-13: Pay close attention to health state, consider treatment or euthanasia
14-18: Euthanasia *Start treatment with antibiotics. If no effect within 3 days, euthanize the animal

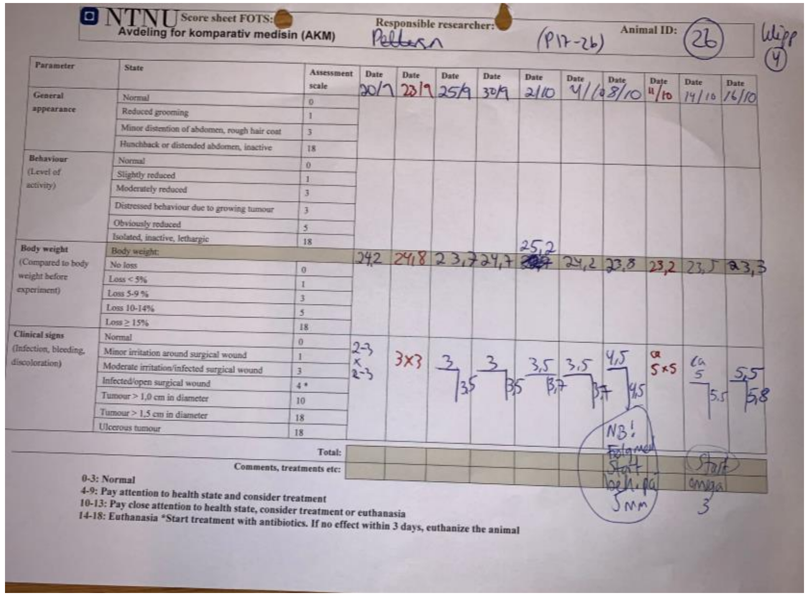
NTNU Score sheet FOTS: Avdeling for komparativ medisin (AKM)
Responsible researcher: Pettersen (P17-26)
Animal ID: 26

Parameter	State	Assessment scale
General appearance	Normal	0
	Reduced grooming	1
	Minor distention of abdomen, rough hair coat	3
	Hunchback or distended abdomen, inactive	18
Behaviour (Level of activity)	Normal	0
	Slightly reduced	1
	Moderately reduced	3
	Distressed behaviour due to growing tumour	3
	Obviously reduced	5
	Isolated, inactive, lethargic	18
Body weight (Compared to body weight before experiment)	Body weight:	
	No loss	0
	Loss < 5%	1
	Loss 5-9 %	3
	Loss 10-14%	5
	Loss ≥ 15%	18
Clinical signs (Infection, bleeding, discoloration)	Normal	0
	Minor irritation around surgical wound	1
	Moderate irritation/infected surgical wound	3
	Infected/open surgical wound	4 *
	Tumour > 1,0 cm in diameter	10
	Tumour > 1,5 cm in diameter	18
	Ulcerous tumour	18
Total:		
Comments, treatments etc:		

0-3: Normal
4-9: Pay attention to health state and consider treatment
10-13: Pay close attention to health state, consider treatment or euthanasia
14-18: Euthanasia *Start treatment with antibiotics. If no effect within 3 days, euthanize the animal

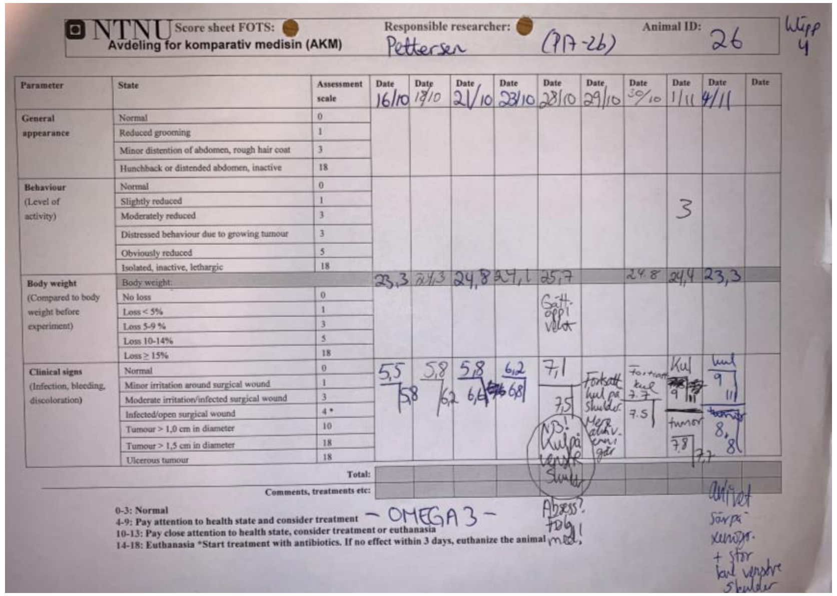
NTNU Score sheet FOTS: Avdeling for komparativ medisin (AKM)
Responsible researcher: Pettersen (P17-26)
Animal ID: 26

Parameter	State	Assessment scale
General appearance	Normal	0
	Reduced grooming	1
	Minor distention of abdomen, rough hair coat	3
	Hunchback or distended abdomen, inactive	18
Behaviour (Level of activity)	Normal	0
	Slightly reduced	1
	Moderately reduced	3
	Distressed behaviour due to growing tumour	3
	Obviously reduced	5
	Isolated, inactive, lethargic	18
Body weight (Compared to body weight before experiment)	Body weight:	
	No loss	0
	Loss < 5%	1
	Loss 5-9 %	3
	Loss 10-14%	5
	Loss ≥ 15%	18
Clinical signs (Infection, bleeding, discoloration)	Normal	0
	Minor irritation around surgical wound	1
	Moderate irritation/infected surgical wound	3
	Infected/open surgical wound	4 *
	Tumour > 1,0 cm in diameter	10
	Tumour > 1,5 cm in diameter	18
	Ulcerous tumour	18
Total:		
Comments, treatments etc:		

0-3: Normal
4-9: Pay attention to health state and consider treatment
10-13: Pay close attention to health state, consider treatment or euthanasia
14-18: Euthanasia *Start treatment with antibiotics. If no effect within 3 days, euthanize the animal

– OMEGA 3 –

Supplementary figure 1-26-6. Mouse, xenograft and secondary tumor growth curves

Weight curve

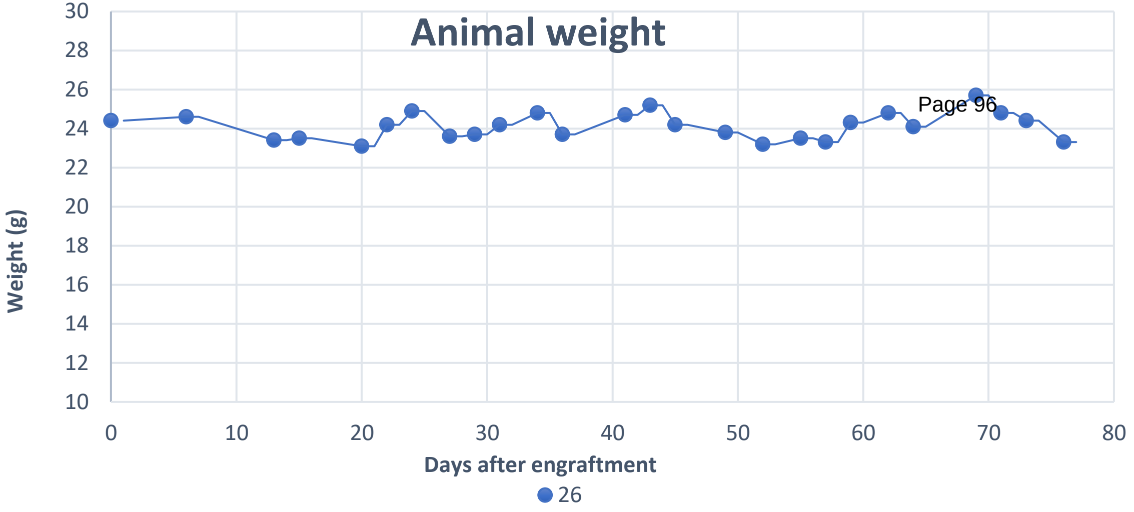

Xenograft growth curve

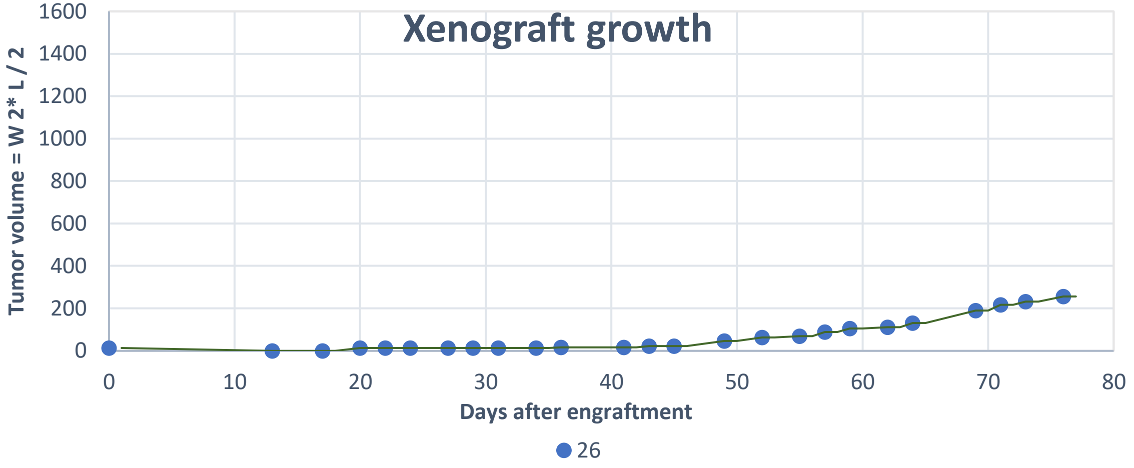

Secondary tumor growth curve

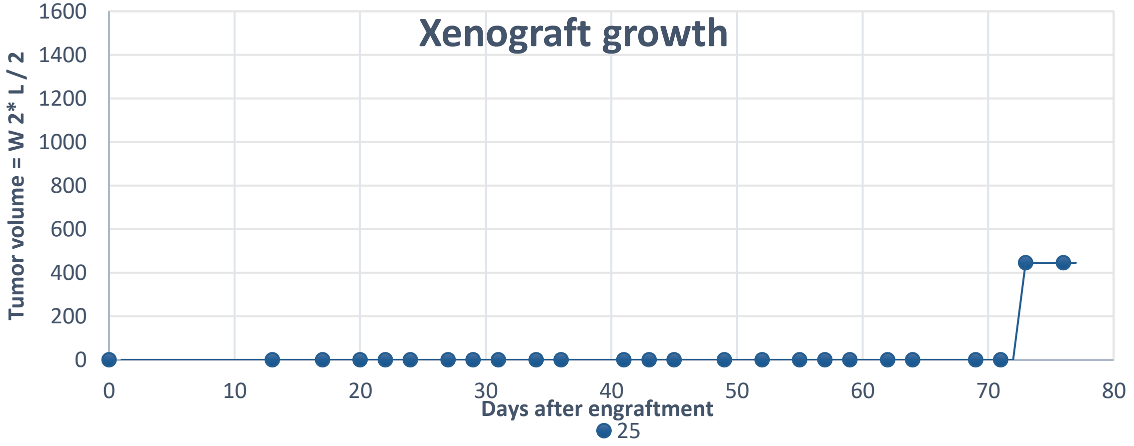

Supplementary figure 1-26-7. Histology pictures

- Two pathologists both confirmed that the secondary tumors consisted of lymphoid cells/lymphoma

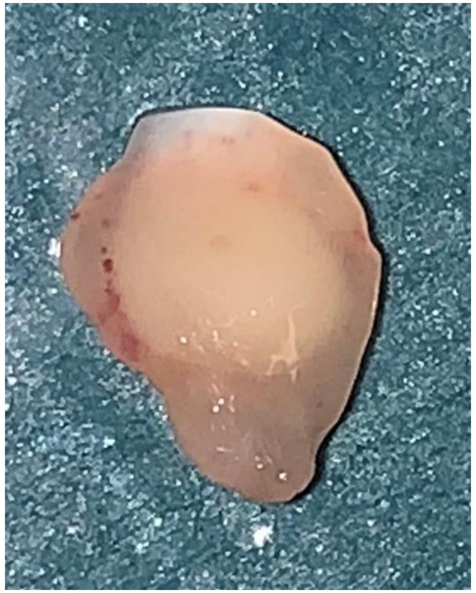

Xenograft

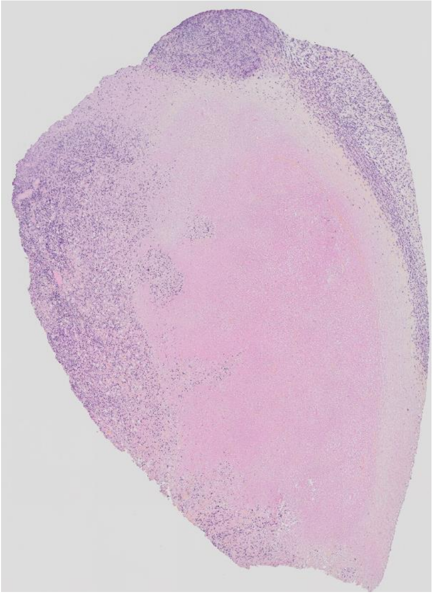

HES stained xenograft slide

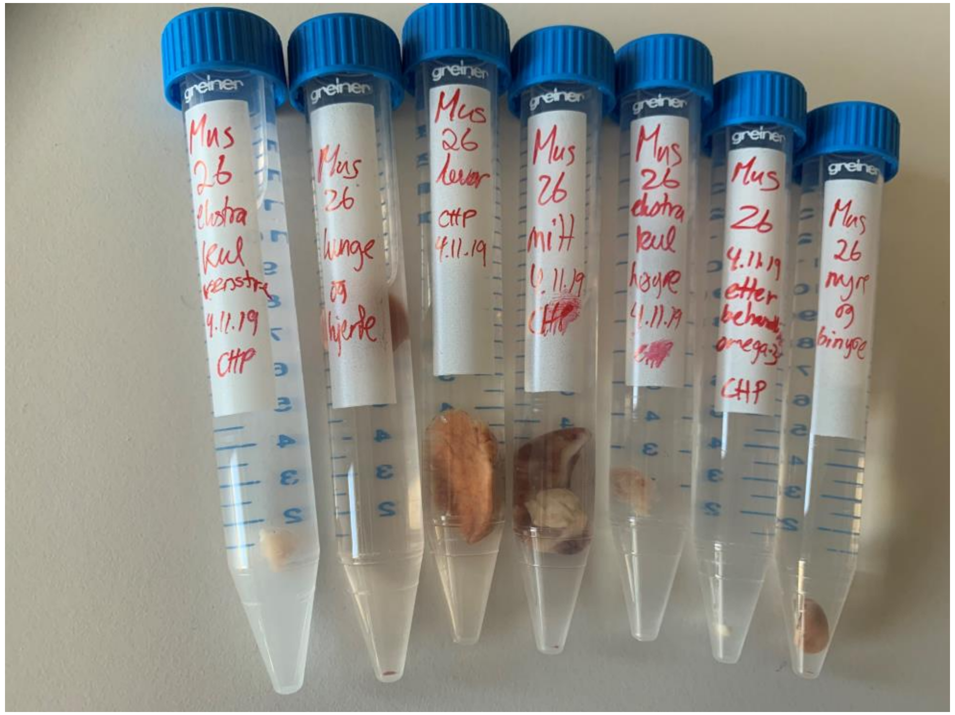

Supplementary figure 1-26-7. Histology pictures

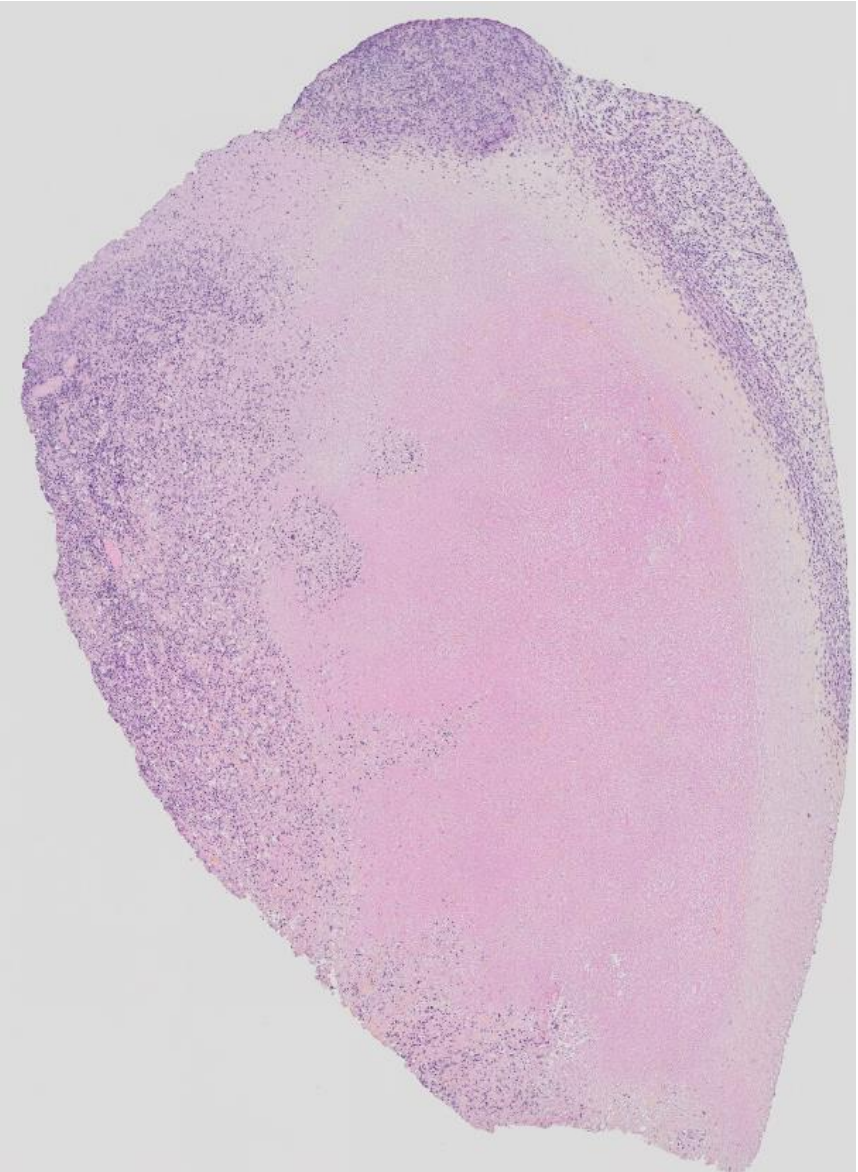

Mouse 26 X (xenograft)

- HES staining
    - Hematoxylin – blue – nuclear staining
    - Eosin – pink – Extracellular matrix and cytoplasma
    - Safran – yellow – stains collagen fibers/connective tissue
- Large necrotic aerea in the middle of the xenograft
- Lymphoid tumor cells surrounding the xenograftet
- Seems like the lyphoid cells have «strungled» the xenograft

Supplementary figure 1-26-7.
Histology pictures

Mouse 26 X (xenograft)

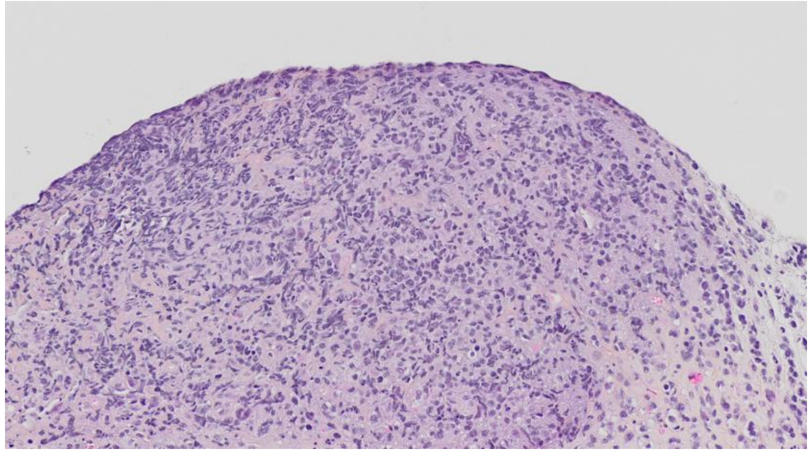

Compact tissue with tumor cells
- Some unregular nuclei
- Several mitoses

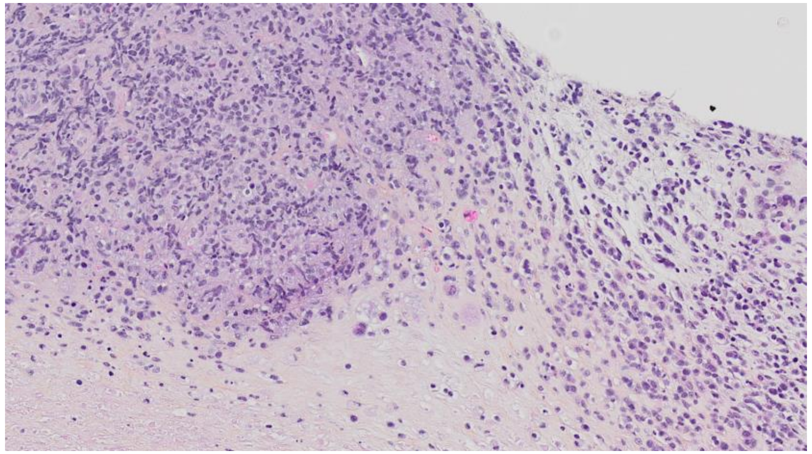

Border from lymphoid cancer cells to necrotic tissue

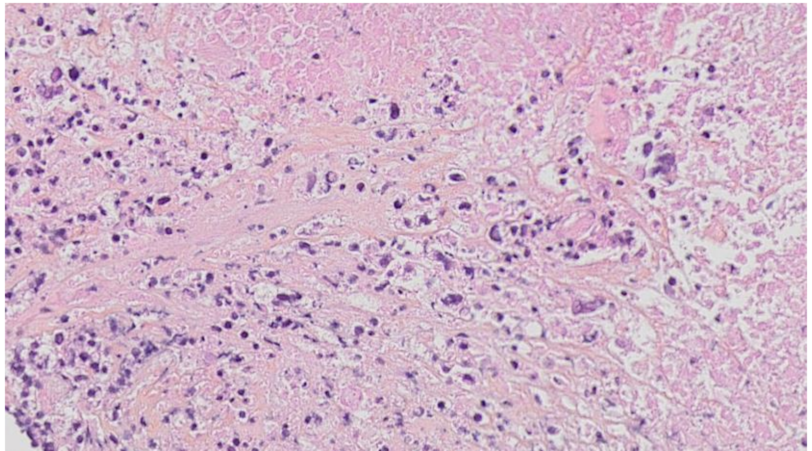

Necrotic tissue

Supplementary figure 1-26-7. Histology pictures

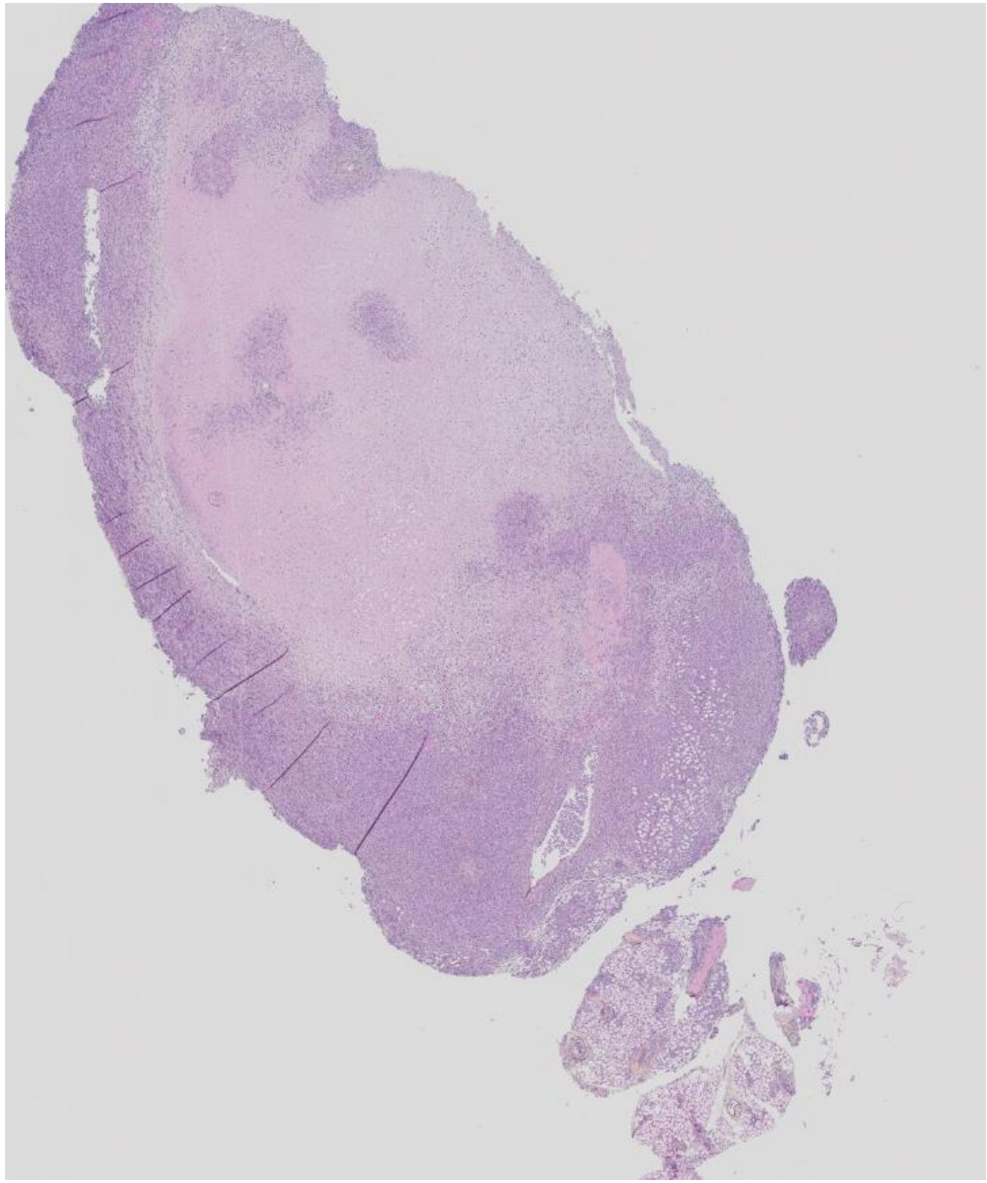

Mouse 26 H (tumor right shoulder)

- Necrotic tissue in the middle
- Lymphoid tumor cells

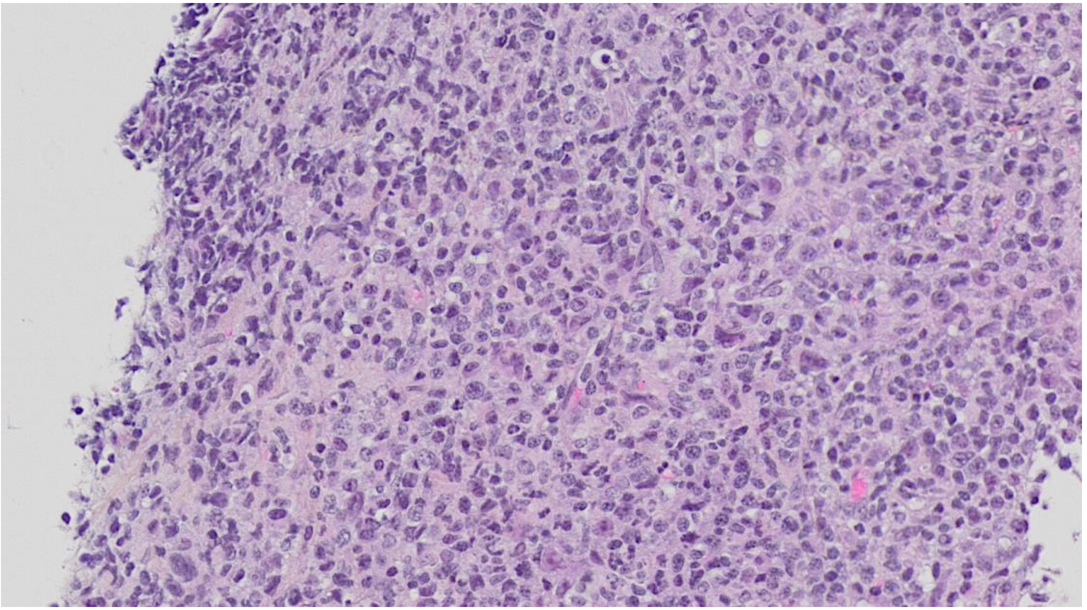

Supplementary figure 1-26-7. Histology pictures

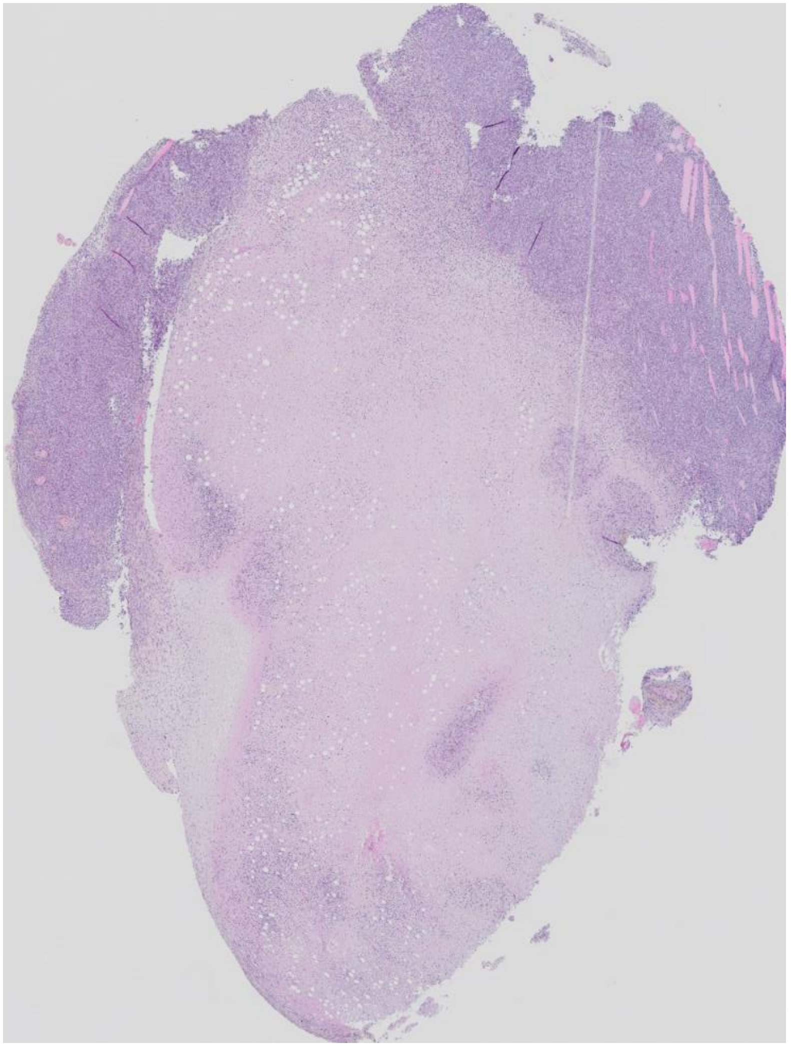

Mouse 26 V (tumor left shoulder)

- Lymphoid tumor cells
- Necrosis in the middle

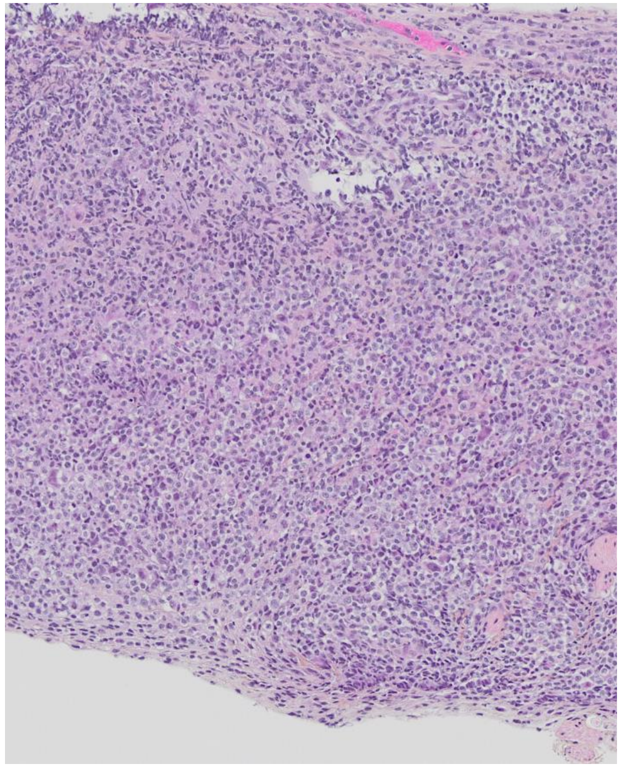

Supplementary figure 1-26-7. Histology pictures

Mouse 26 LH (Lung)

- Dense aereas of tumor tissue in the lungs
- Lymphoid tumor cells

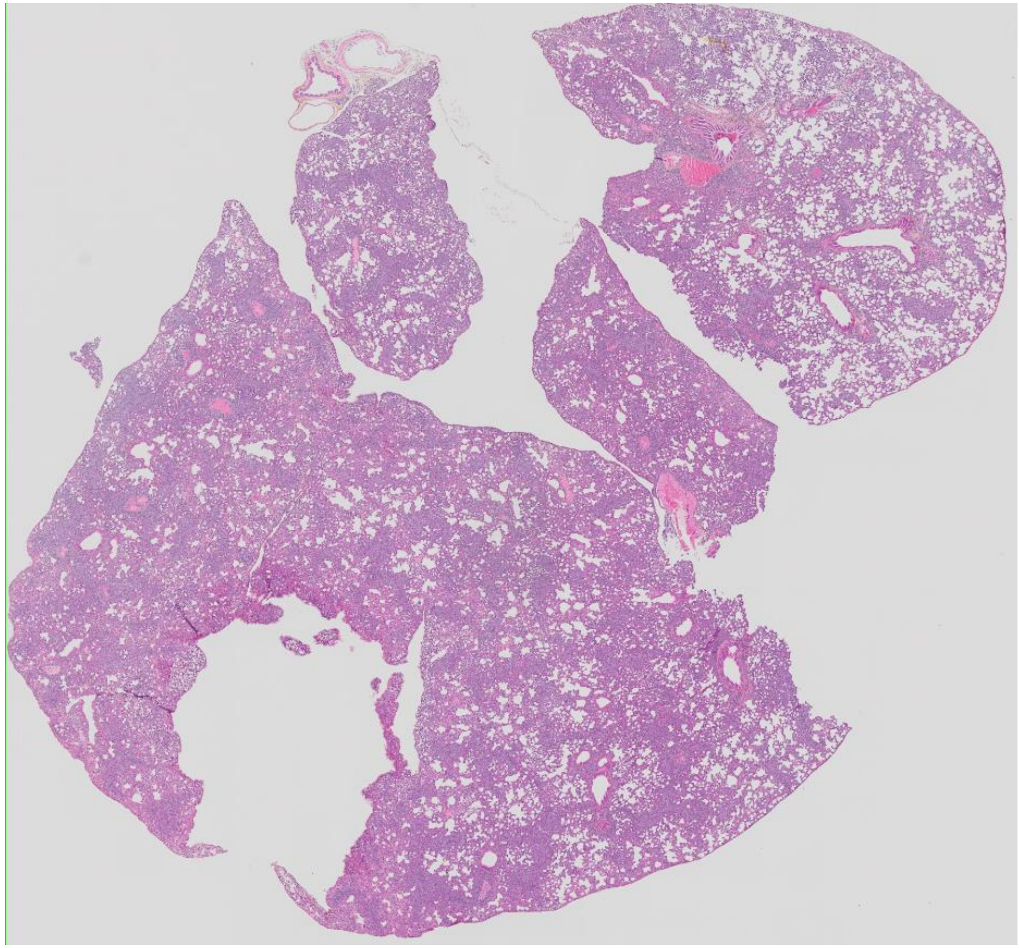

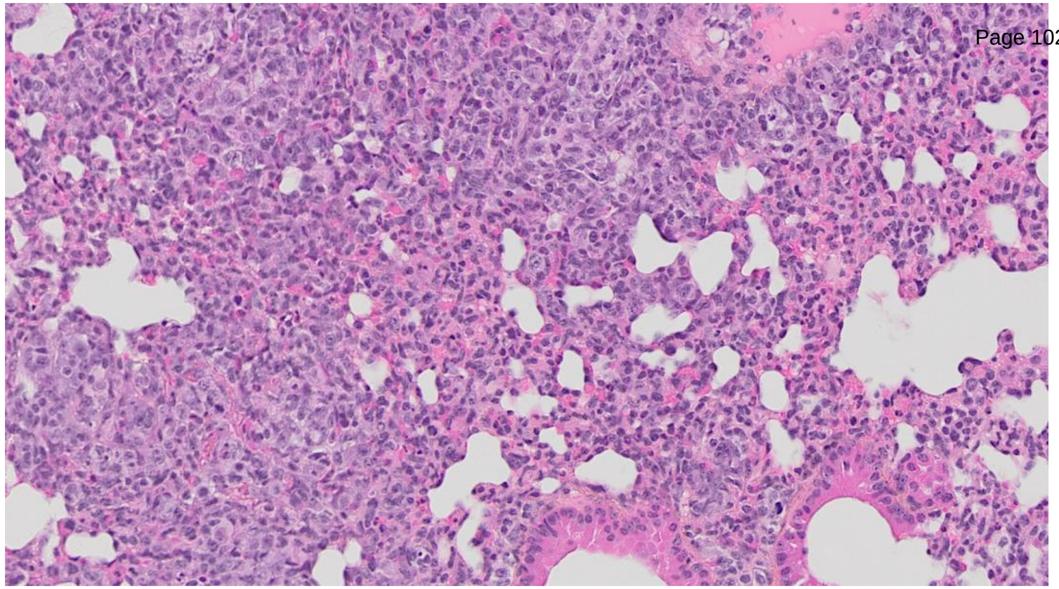

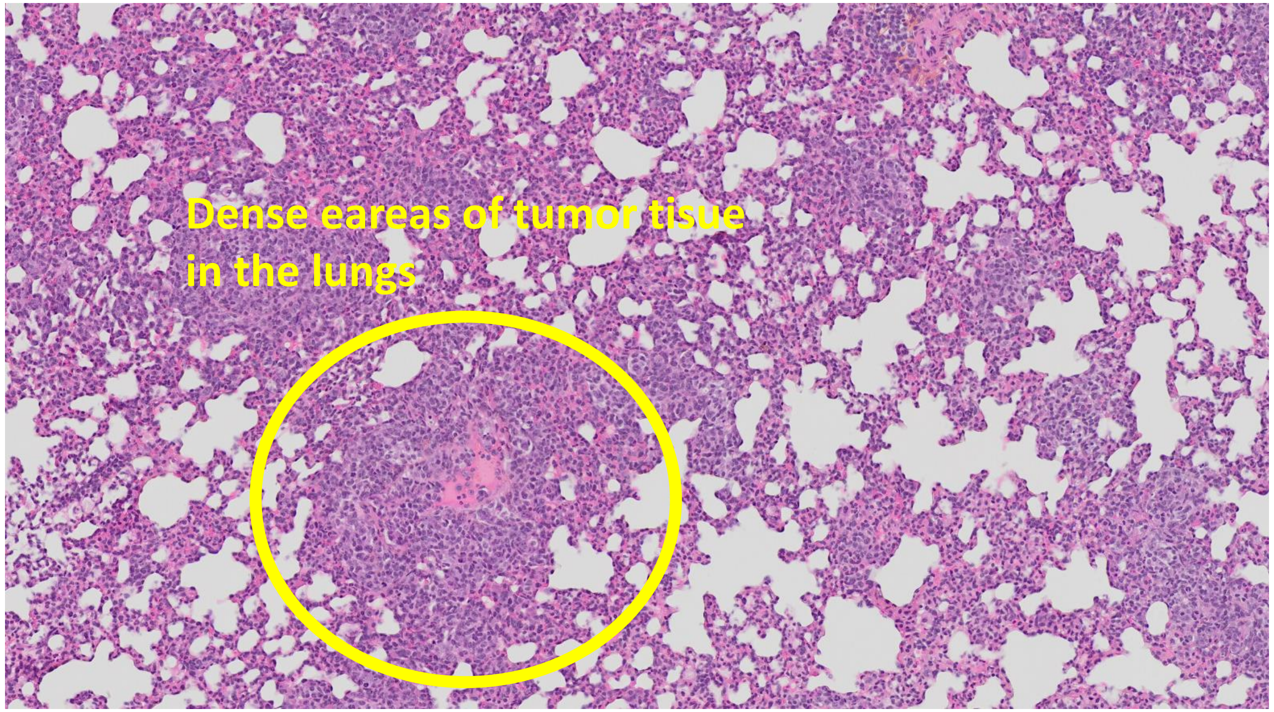

Supplementary figure 1-26-7. Histology pictures

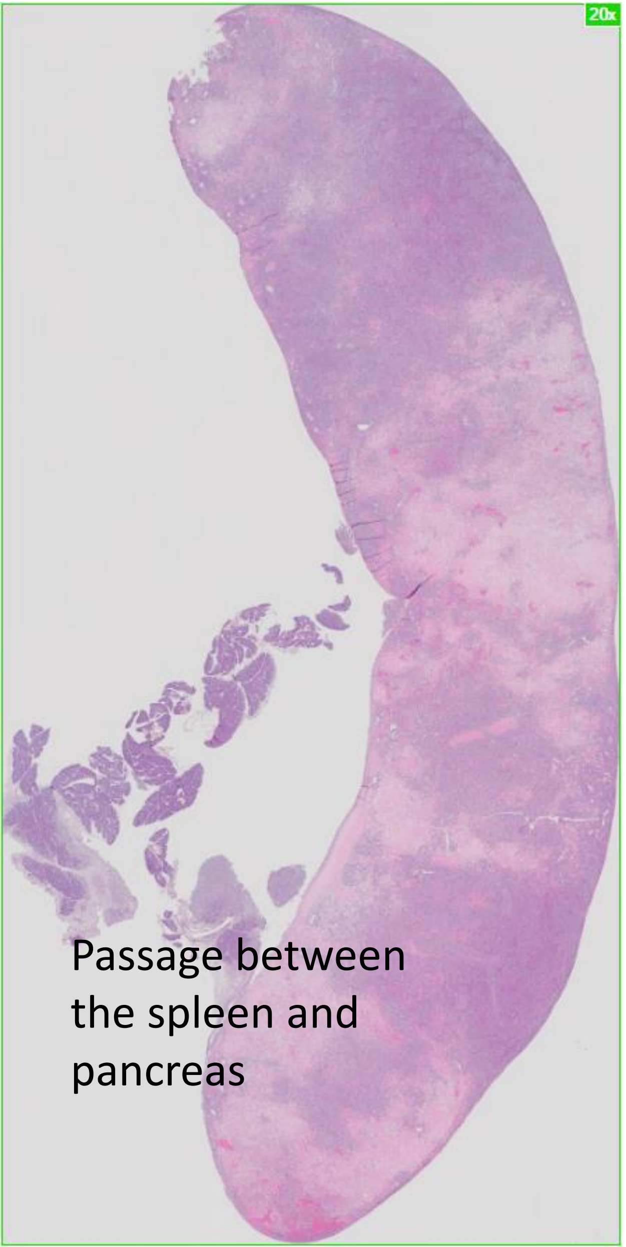

Mouse 26 M (Spleen)

- Large spleen (2.2 cm, ca 3 when not bent)
- Pancreas attached
- White necrotic fields
- Plenty tumor cells in the spleen
- Possible bleedings in the spleen

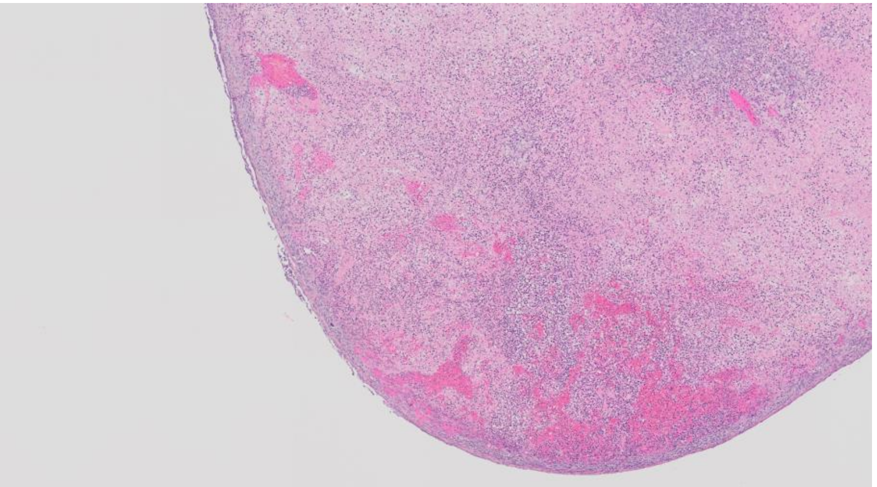

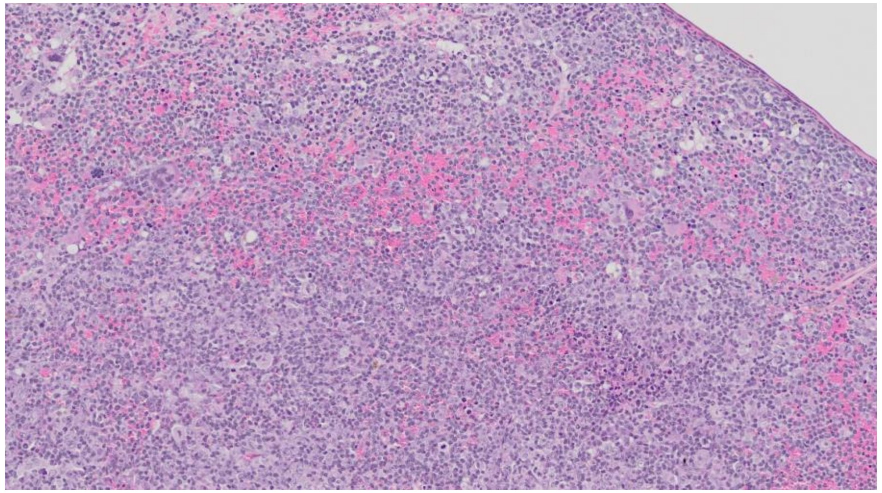

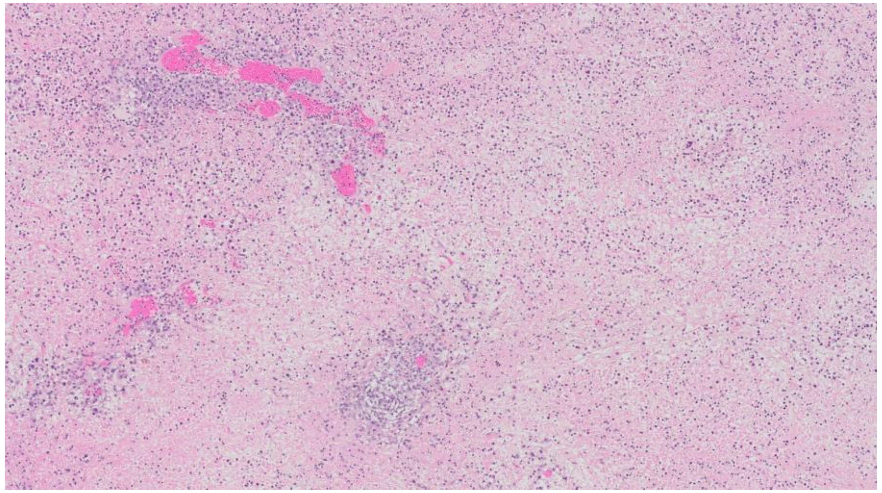

Supplementary figure 1-26-7. Histology pictures

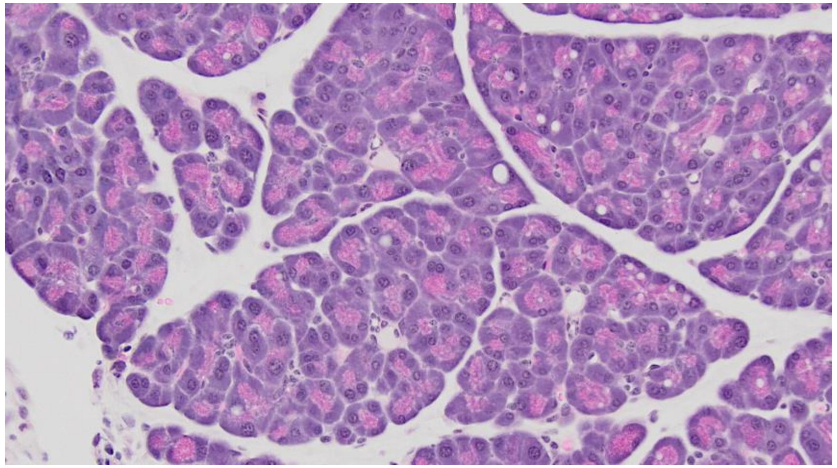

Pancreatic cells

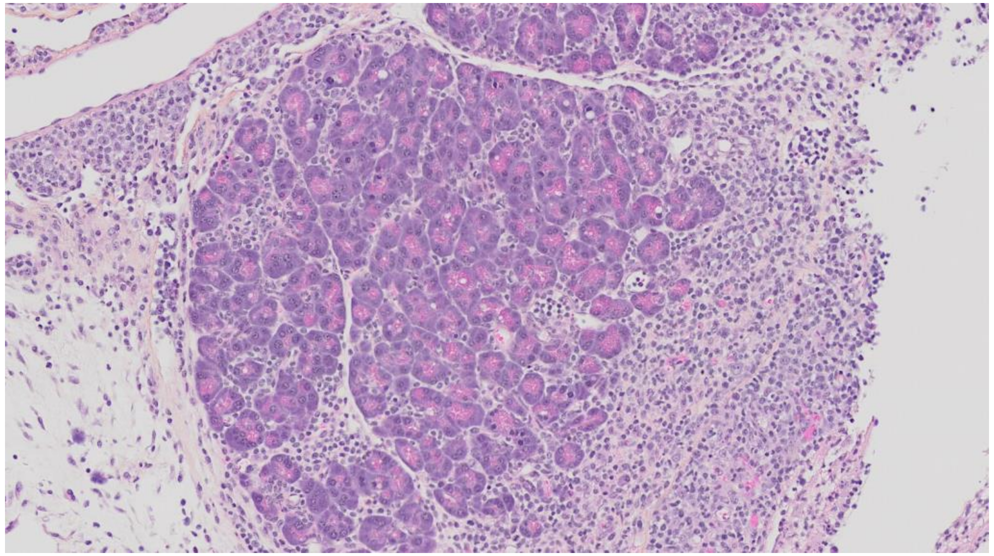

Pancreatic cells infiltrated by tumor cells

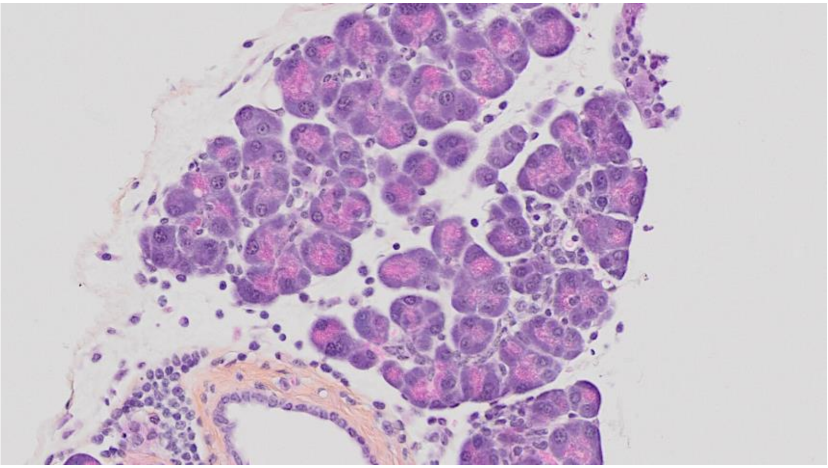

Pancreatic cells infiltrated by tumor cells

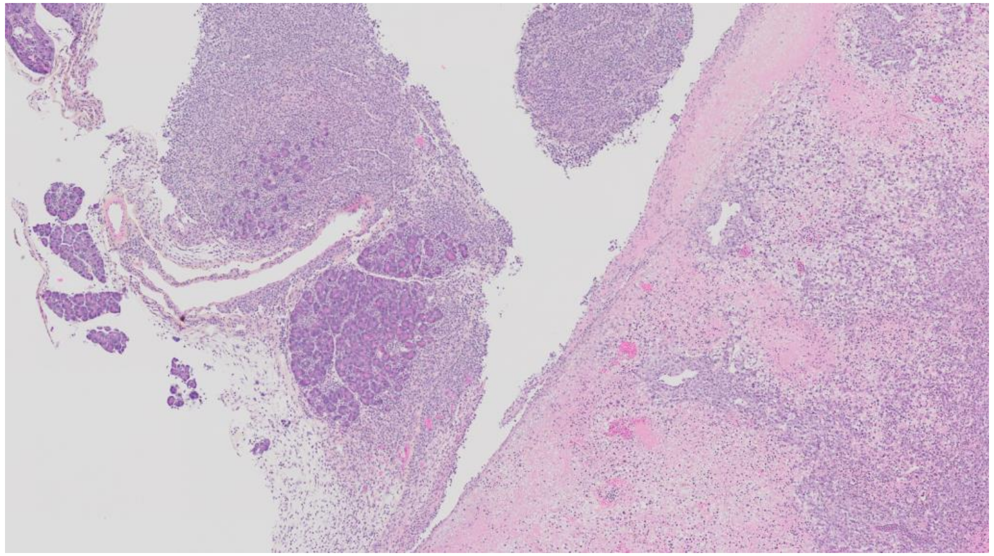

Passage between the spleen and pancreas

Supplementary figure 1-26-8. Histology and immunohistochemistry pictures of secondary tumor (left)

Mouse 26 V (tumor left shoulder)

- Lymphoma
- IHC staining of Ki67 and CD45
- EBER-ISH staining of EBV

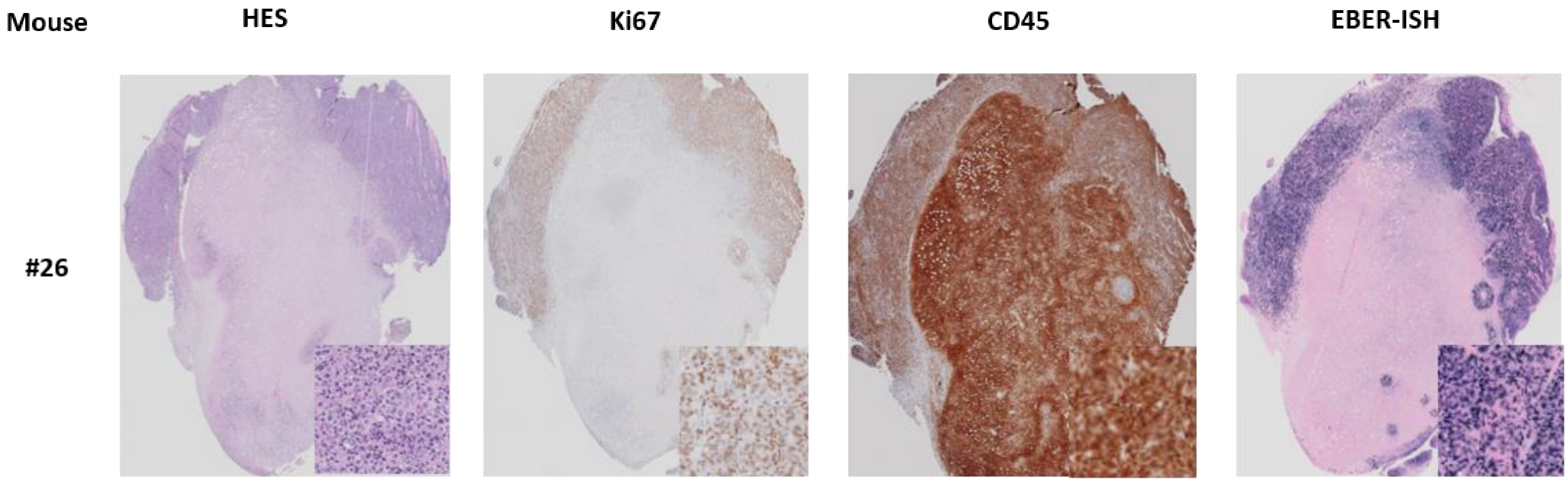

Mouse 27

- Surgery date: 21.08.19
    - Age (weeks) 31
    - Weight (g) 22
    - Tumor fragment P18-1a
    - Fragment size 3 x 3
    - Ear mark 1

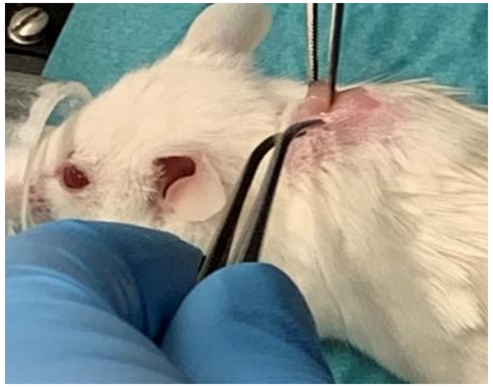

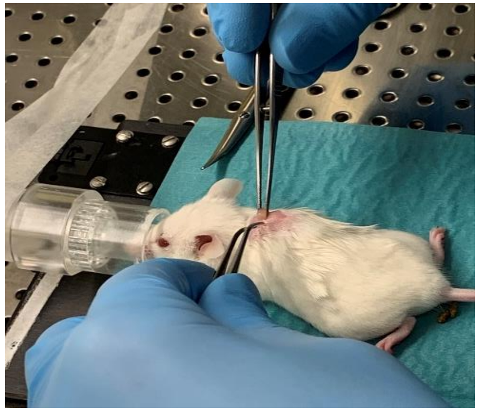

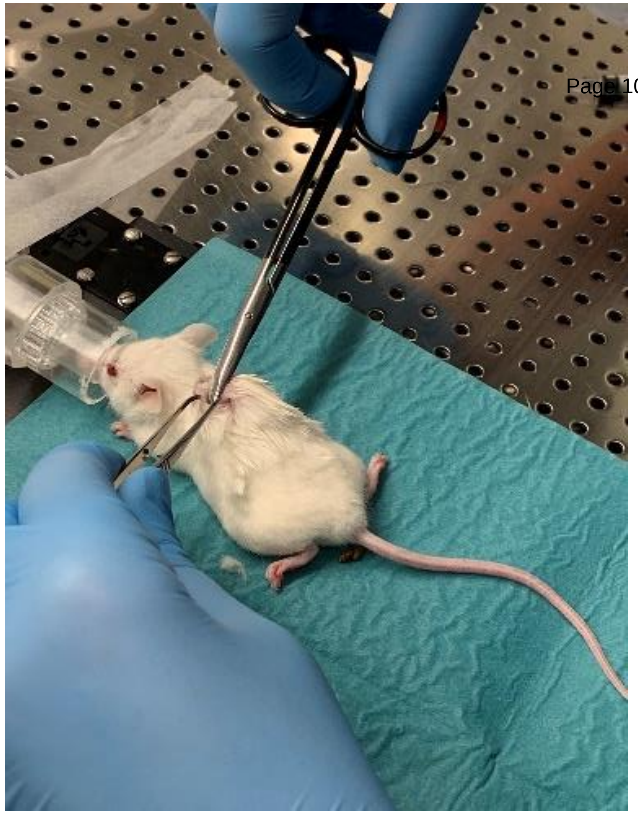

Supplementary figure 1-27-1.
Pictures from the engraftment day

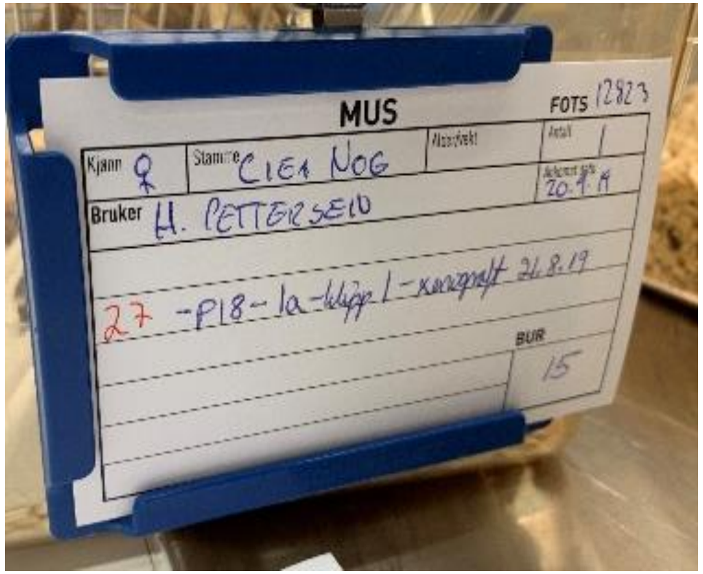

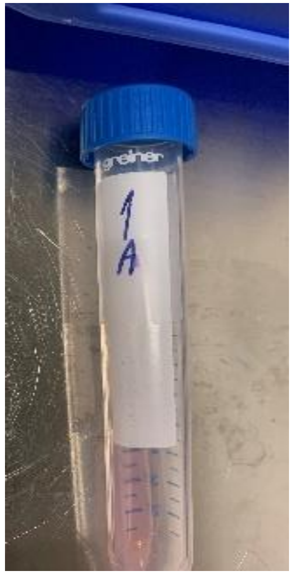

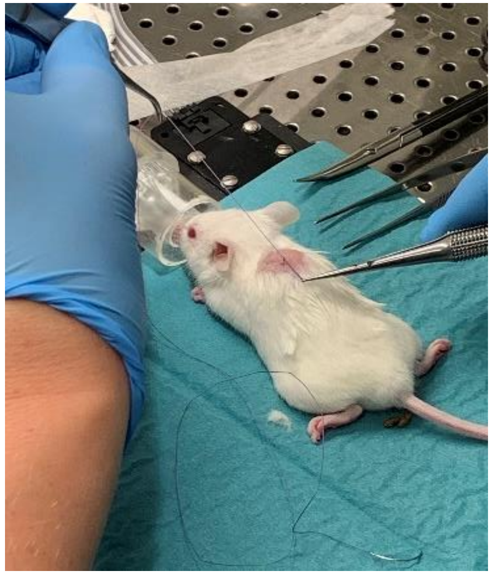

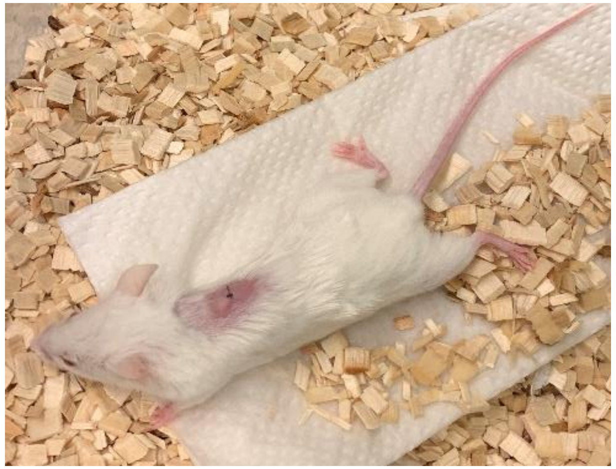

Supplementary figure 1-27-2. Pictures from follow up and xenograft size measurement

Mouse ID

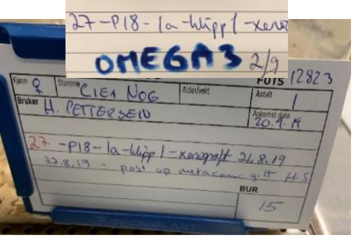

0w + 5d

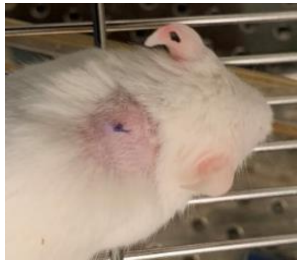

1w + 1d

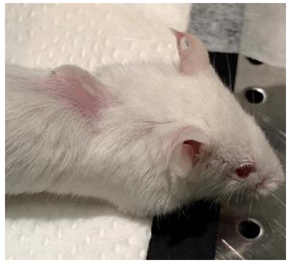

«Tumor» ca 5 x 5 mm

1w + 5d

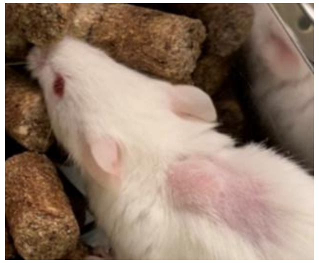

«Tumor» ca 6 x 7 mm

2w + 0d

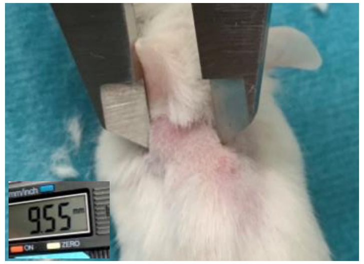

«Tumor» ca 9.5 x 5.5 mm

2w + 2d

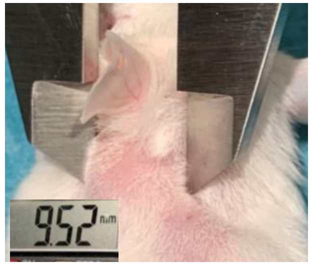

«Tumor» ca 9.5 mm

2w + 5d

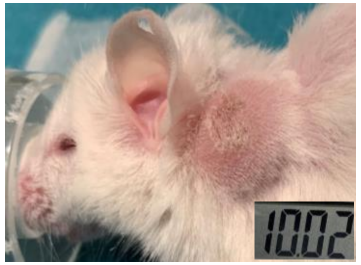

3w + 0d

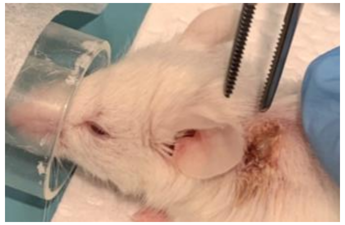

Treatment:

- Smartfish Remune with fish oil
- Blodsamples taken before and after treatment

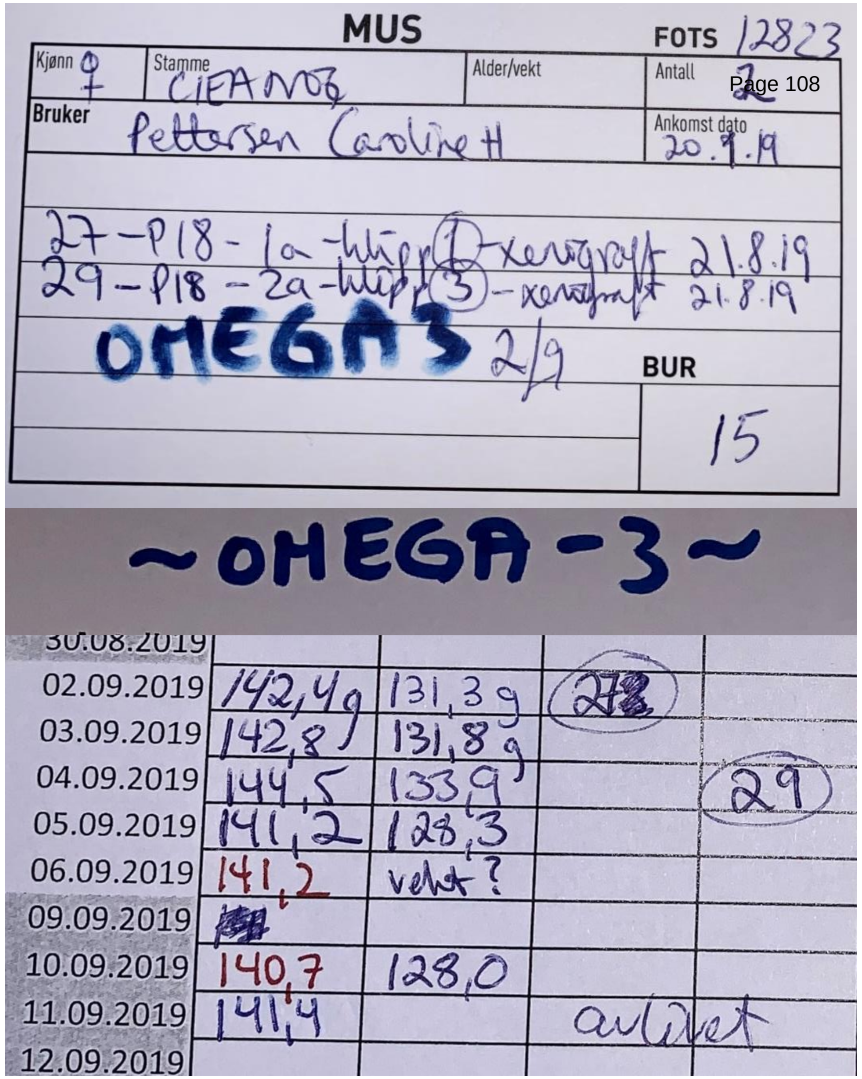

Supplementary figure 1-27-3. List over weight registrations of the drinking bottle (with Smartfish Remune Peach) before and after provided to the mice

Supplementary figure 1-27-4. Pictures from euthanization (11.09.19)

- Not the xenograft, but an abscess that was growing

Mus ID

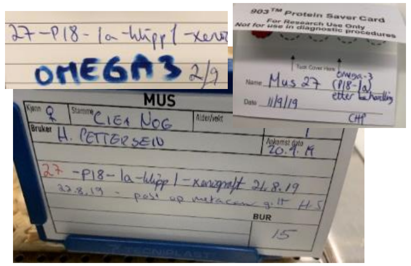

19 dager post op

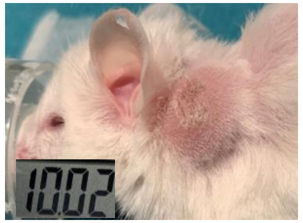

21 dager/avliving

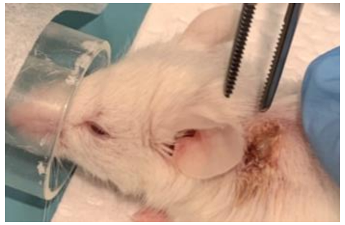

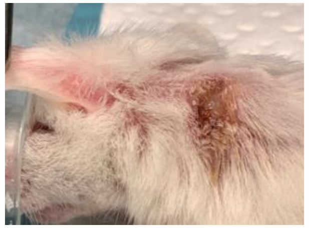

Scurvy wound, the «tumor» has bursted. Turned out it was an abscess and not the xenograft growing.

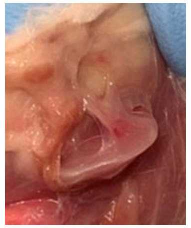

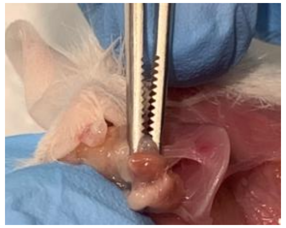

Small xenograft under the skin

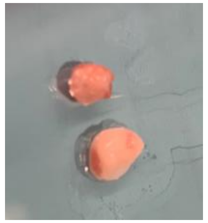

The largest fragment was devided into the 3 sample tubes, and the smallest fragment in a «left over» tube.

Supplementary figure 1-27-5. Score sheet

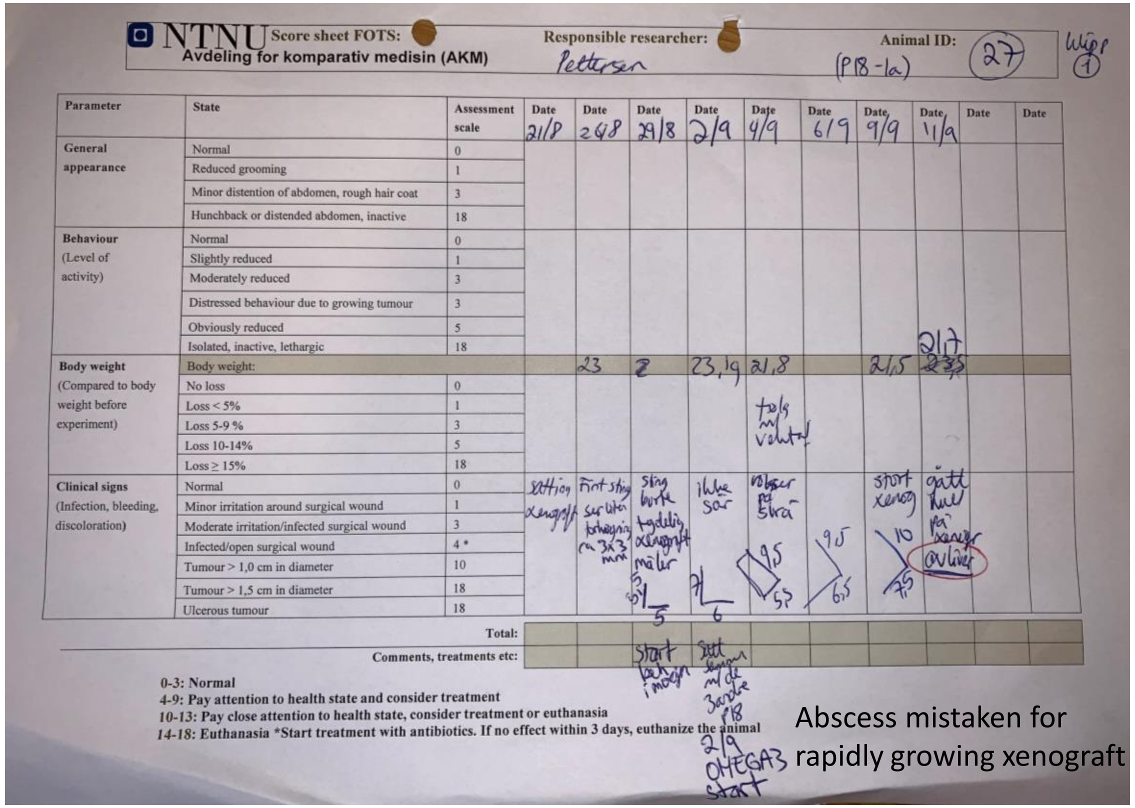

NTNU Score sheet FOTS: Responsible researcher: Pettersen Animal ID: (P18-1a) 27
Avdeling for komparativ medisin (AKM)

Parameter	State	Assessment scale	Date 21/8	Date 24/8	Date 29/8	Date 2/9	Date 4/9	Date 6/9	Date 9/9	Date 11/9	Date	Date
General appearance	Normal	0										
	Reduced grooming	1										
	Minor distention of abdomen, rough hair coat	3										
	Hunchback or distended abdomen, inactive	18										
Behaviour (Level of activity)	Normal	0										
	Slightly reduced	1										
	Moderately reduced	3										
	Distressed behaviour due to growing tumour	3										
	Obviously reduced	5										
	Isolated, inactive, lethargic	18										
Body weight (Compared to body weight before experiment)	Body weight:			23	[illegible]	23,19	21,8		21,5	21,7 / 23,5		
	No loss	0										
	Loss < 5%	1										
	Loss 5-9 %	3										
	Loss 10-14%	5										
	Loss ≥ 15%	18										
Clinical signs (Infection, bleeding, discoloration)	Normal	0	[illegible] xenograft	[illegible]	[illegible] 5	Ikke sår 6	[illegible] 9,5 / 5,5	9,5 / 6,5	Start xenog 10 / 7,5	[illegible]		
	Minor irritation around surgical wound	1										
	Moderate irritation/infected surgical wound	3										
	Infected/open surgical wound	4 *										
	Tumour > 1,0 cm in diameter	10										
	Tumour > 1,5 cm in diameter	18										
	Ulcerous tumour	18										
		Total:										
		Comments, treatments etc:			Start [illegible]	[illegible]						

0-3: Normal
4-9: Pay attention to health state and consider treatment
10-13: Pay close attention to health state, consider treatment or euthanasia
14-18: Euthanasia *Start treatment with antibiotics. If no effect within 3 days, euthanize the animal

2/9 OMEGA3 start

Abscess mistaken for rapidly growing xenograft

Supplementary figure 1-27-6.

Mouse and abscess growth curves

Weight curve

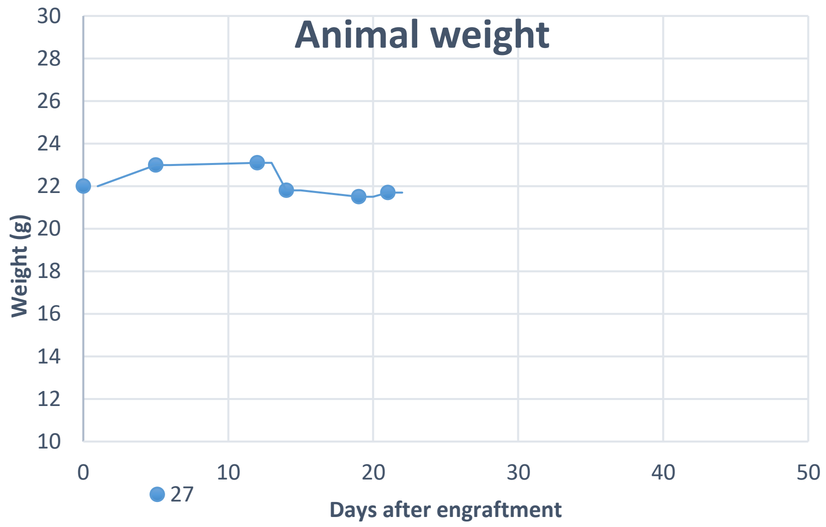

Size of abscess

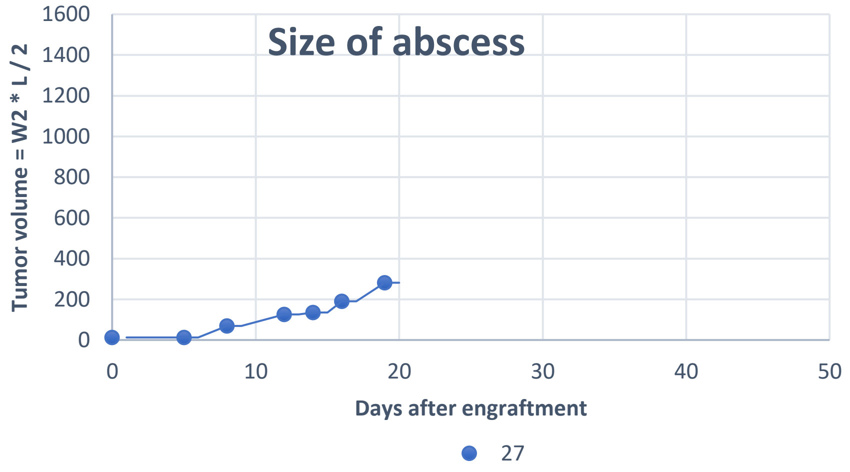

Mouse 28

- Surgery date: 21.08.19
  - Age (weeks) 31
  - Weight (g) 22.6
  - Tumor fragment P18-1b
  - Fragment size 3 x 3 mm
  - Ear mark 2

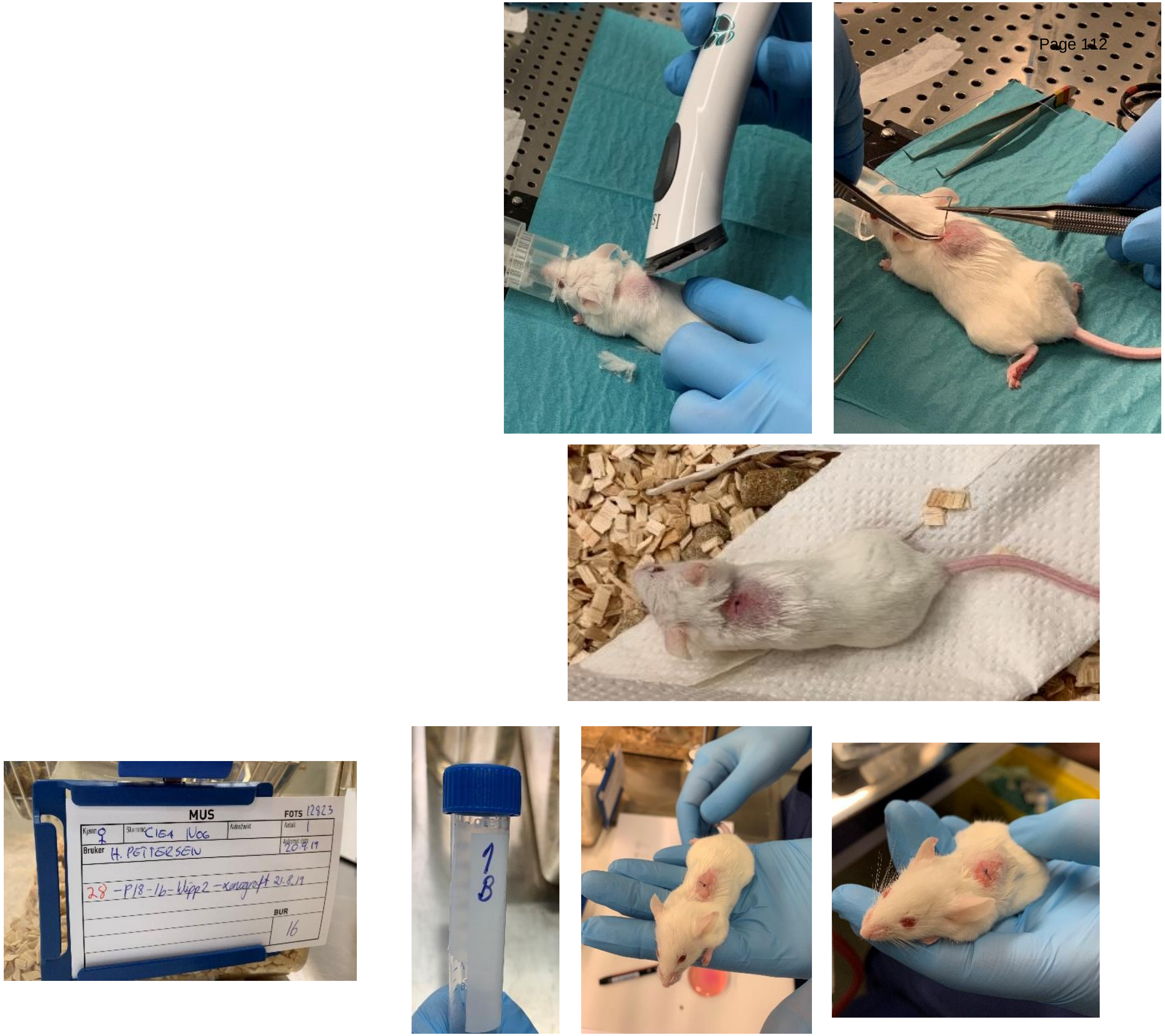

Supplementary figure 1-28-1.
Pictures from the engraftment day

Supplementary figure 1-28-2. Pictures from follow up and xenograft size measurement

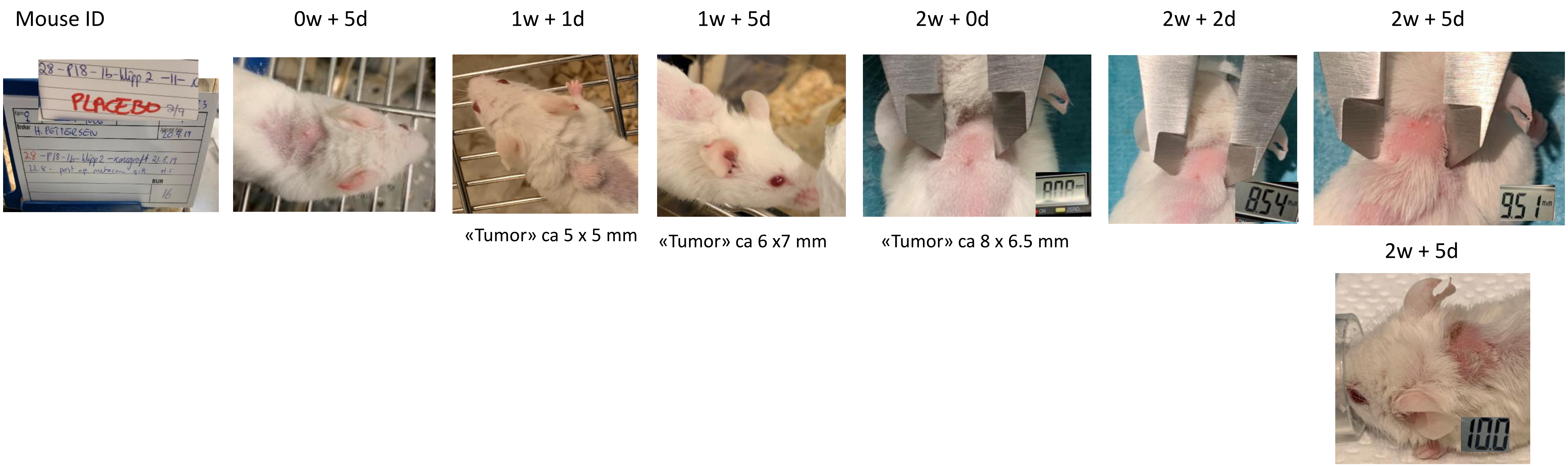

Treatment:

- Placebo (Smartfish Remune with Rapseed oil)
- Blodsamples taken before and after treatment

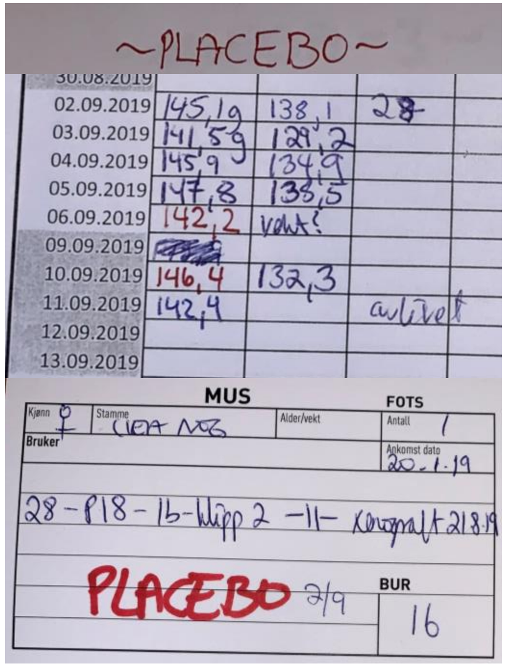

Supplementary figure 1-28-3. List over weight registrations of the drinking bottle (with placebo) before and after provided to the mice

Supplementary figure 1-28-4. Pictures from euthanization (11.09.19)

- Not xenograft but an abscess growing
- Euthanized due to two other mice with bursting abscesses (mouse 27 and 29)

Mus ID | 19 dager post op | 21 dager/avliving

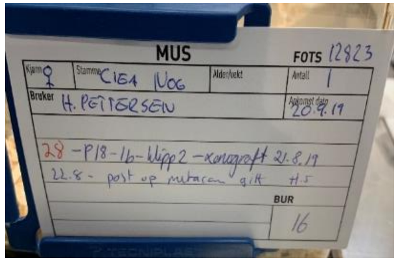

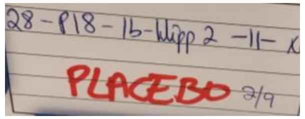

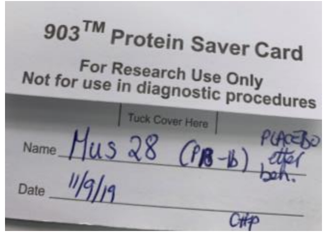

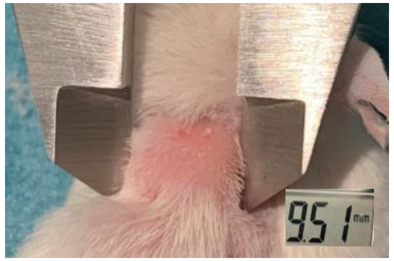

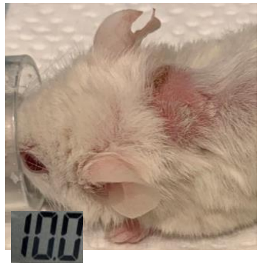

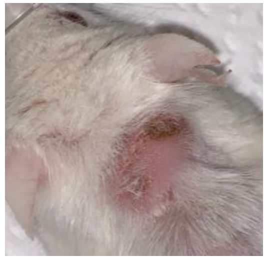

Brown fields/scurvy forming on the skin over the «tumor» (abscess)

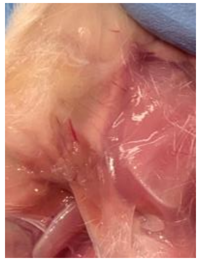

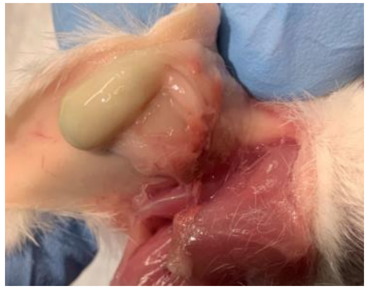

Green abscess. Green liquid coming out when pinched. Clearly not the xenograft.

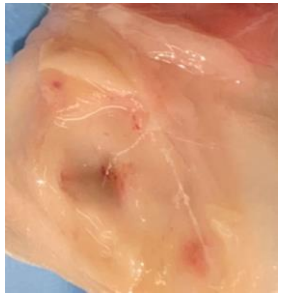

Almost no xenograft visiable, nothing to sample

Supplementary figure 1-28-5. Score sheet

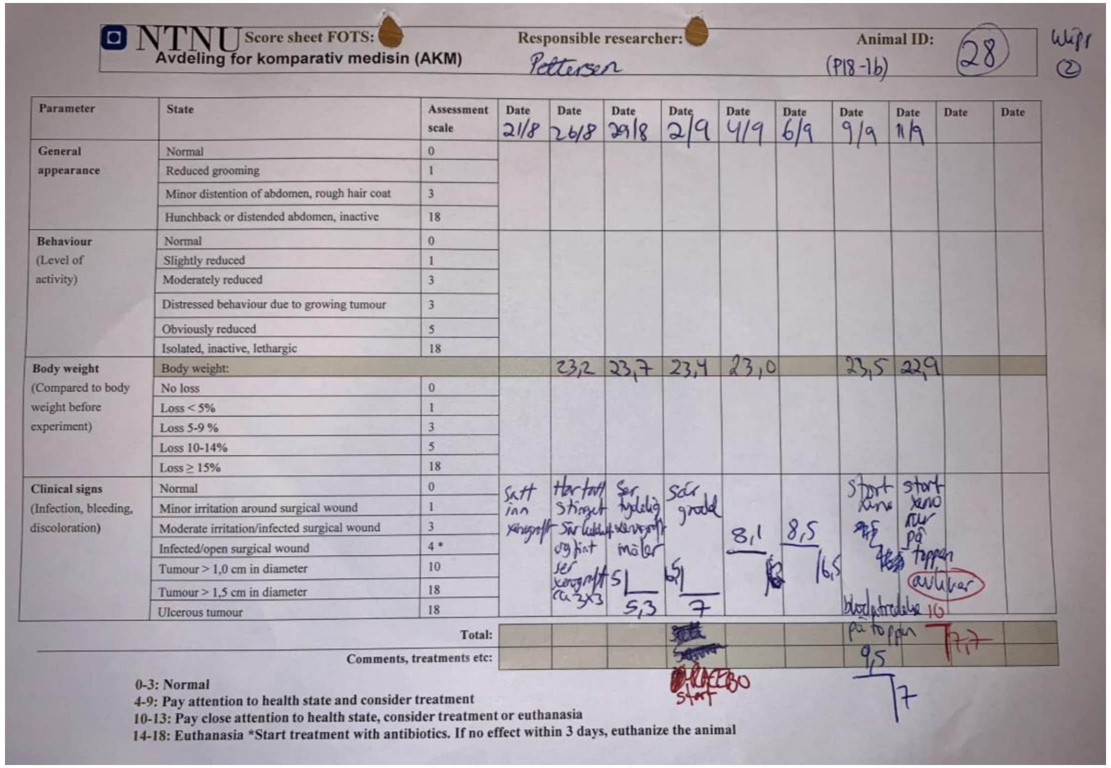

NTNU Score sheet FOTS: Responsible researcher: Pettersen Animal ID: 28 (P18-1b) Wipr ②

Avdeling for komparativ medisin (AKM)

Parameter	State	Assessment scale	Date 21/8	Date 26/8	Date 29/8	Date 2/9	Date 4/9	Date 6/9	Date 9/9	Date 11/9	Date	Date
General appearance	Normal	0										
	Reduced grooming	1										
	Minor distention of abdomen, rough hair coat	3										
	Hunchback or distended abdomen, inactive	18										
Behaviour (Level of activity)	Normal	0										
	Slightly reduced	1										
	Moderately reduced	3										
	Distressed behaviour due to growing tumour	3										
	Obviously reduced	5										
	Isolated, inactive, lethargic	18										
Body weight (Compared to body weight before experiment)	Body weight:			23,2	23,7	23,4	23,0		23,5	22,9		
	No loss	0										
	Loss < 5%	1										
	Loss 5-9 %	3										
	Loss 10-14%	5										
	Loss ≥ 15%	18										
Clinical signs (Infection, bleeding, discoloration)	Normal	0	Satt inn xenograft	Har tatt stinget Sår lukket og fint ser xenograft ca 3x3	Ser tydelig xenograft måler 5 / 5,3	Sår grodd 5 / 7	8,1 / [illegible]	8,5 / 16,5	Stort xeno [illegible] blodutredelse på toppen	stort xeno nå på toppen avslutter 16		
	Minor irritation around surgical wound	1										
	Moderate irritation/infected surgical wound	3										
	Infected/open surgical wound	4 *										
	Tumour > 1,0 cm in diameter	10										
	Tumour > 1,5 cm in diameter	18										
	Ulcerous tumour	18										
		Total:				[illegible]				7,7		
		Comments, treatments etc:				PLACEBO start			9,5 / 7			

0-3: Normal
4-9: Pay attention to health state and consider treatment
10-13: Pay close attention to health state, consider treatment or euthanasia
14-18: Euthanasia *Start treatment with antibiotics. If no effect within 3 days, euthanize the animal

Note: numbers represent abscess size, not tumor growth.

Supplementary figure 1-28-6.

Mouse and abscess growth curve

Weight curve

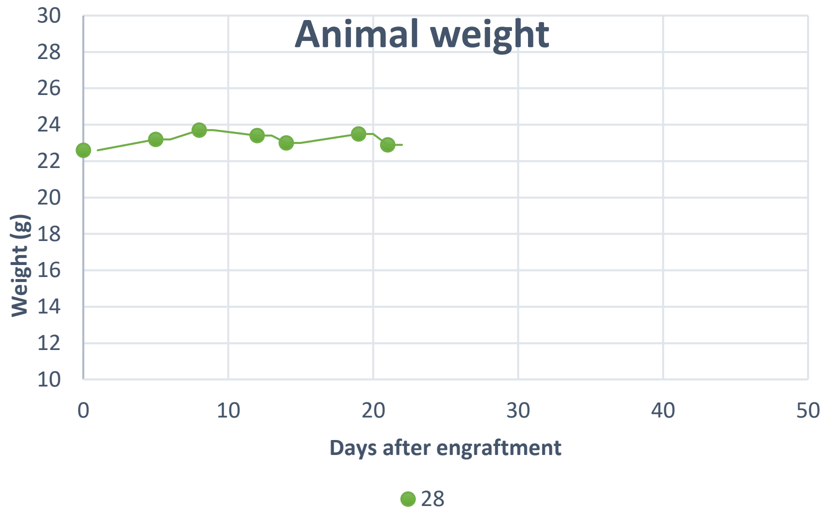

Size of abscess

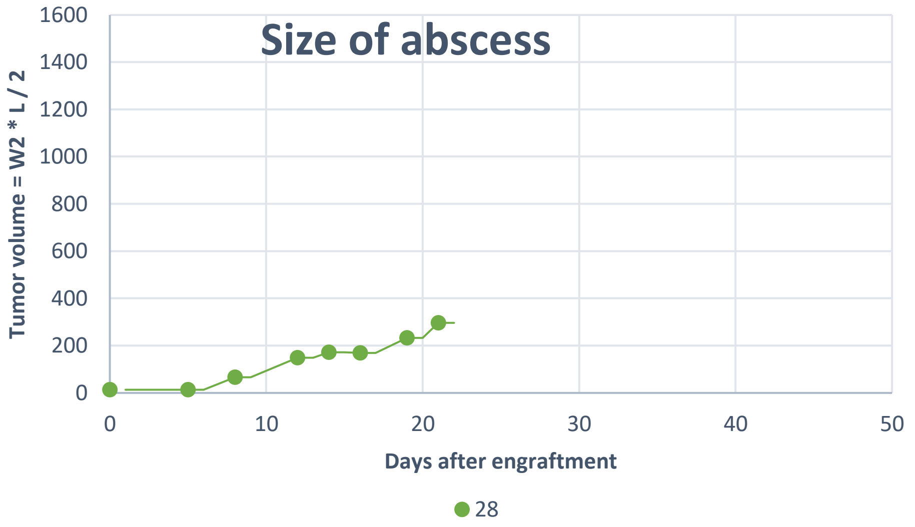

Mouse 29

- Surgery date: 21.08.19
    - Age (weeks) 31
    - Weight (g) 22.82
    - Tumor fragment P18-2a
    - Fragment size 3 x 3 mm
    - Ear mark 3
    - One stich was opened by the mouse after 1 hour. Restiched.

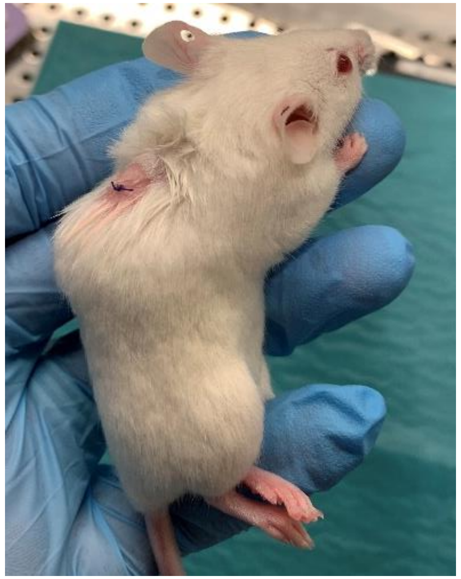

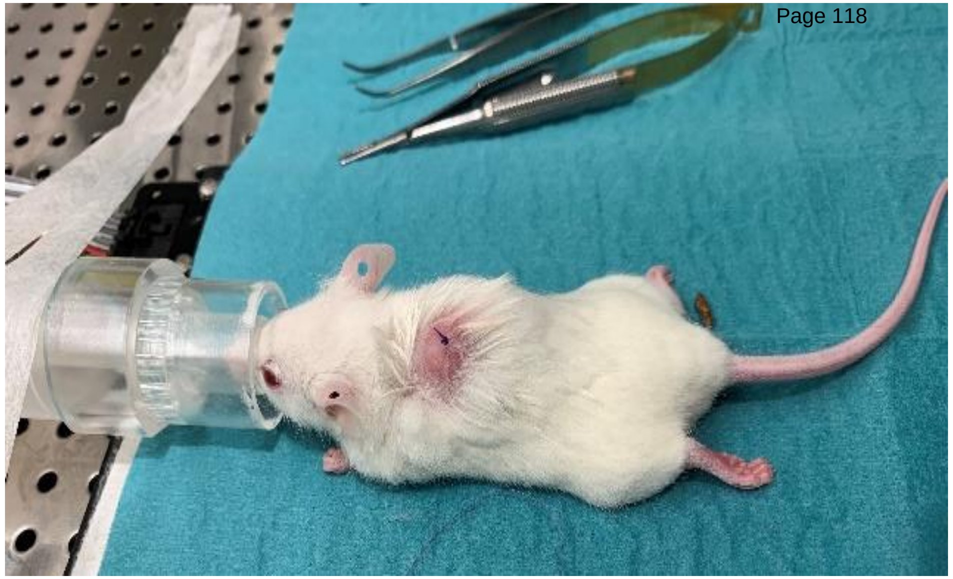

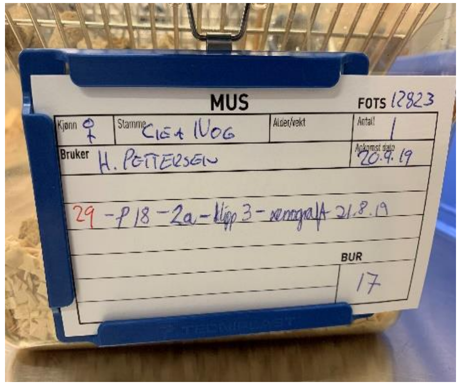

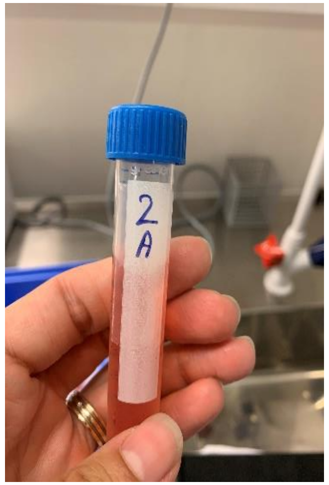

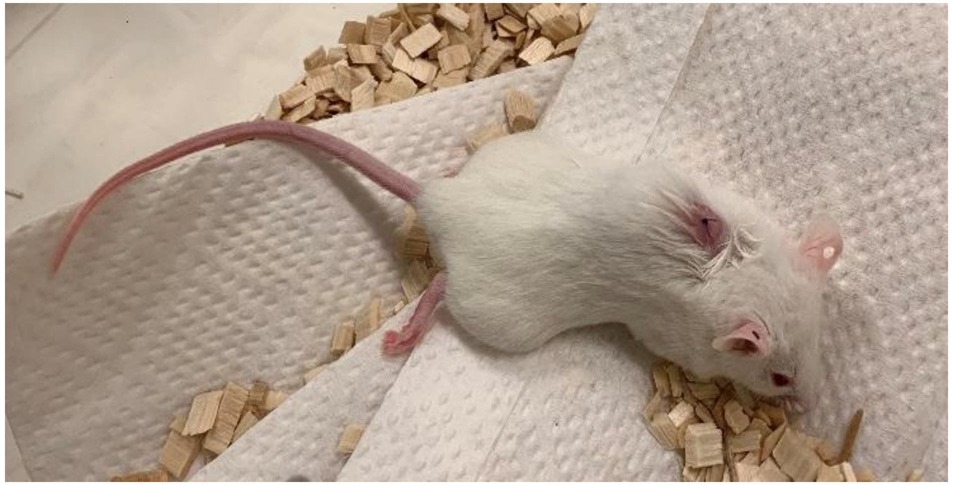

Supplementary figure 1-29-1. Pictures from the engraftment day

Supplementary figure 1-29-2. Pictures from follow up and «xenograft» size measurement

Pictures from follow up

Mouse ID

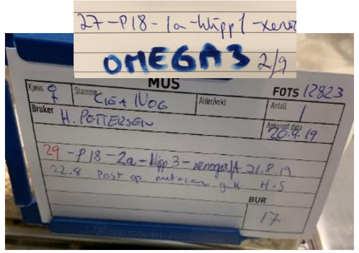

0w + 5d

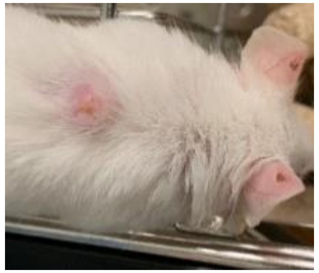

1w + 1d

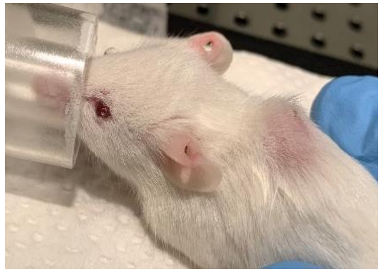

«tumor» ca 5 x 5 mm

1w + 5d

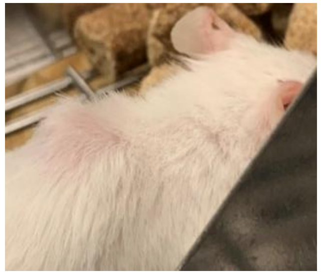

«Tumor» ca 5 x 5 mm

2w + 0d

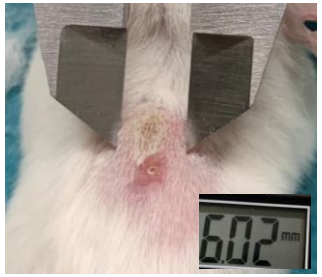

«Tumor» ca 6 x 5,5 mm

2w + 2d

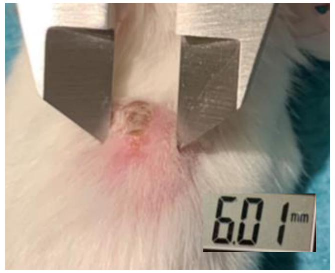

«Tumor» ca 6 mm

2w + 5d

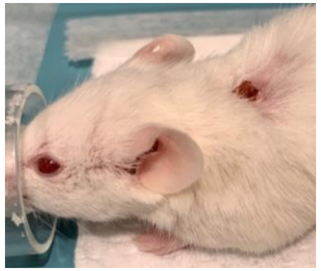

Wound with «crater»

Treatment:

- Smartfish Remune with fishoil
- Blodsamples before/after

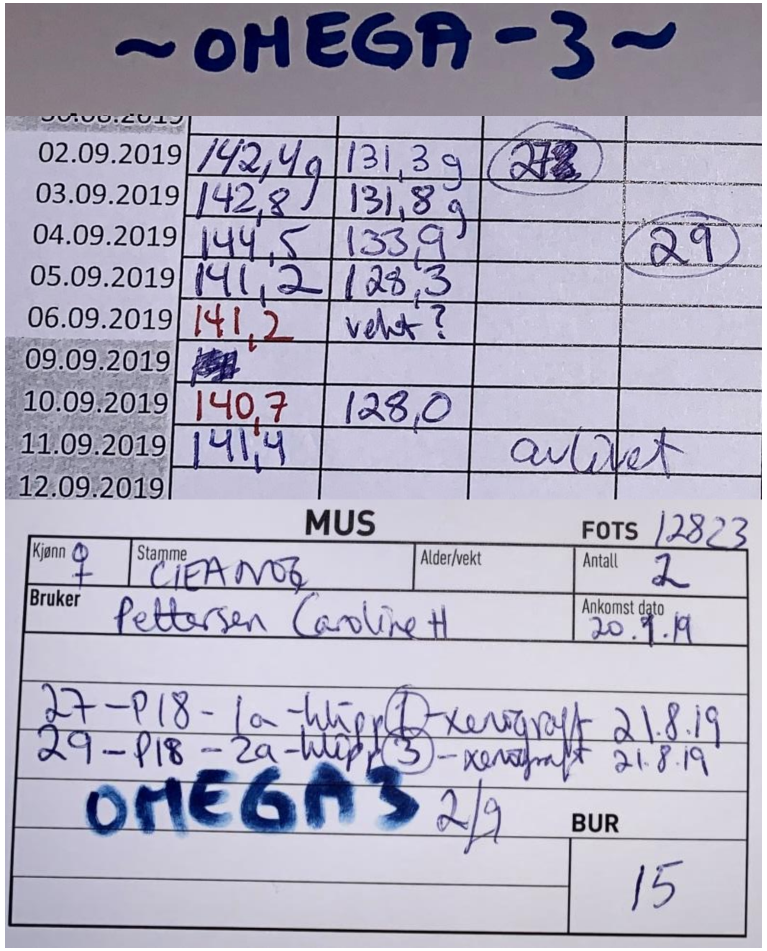

Supplementary figure 1-29-3. List over weight registrations of the drinking bottle (with Smartfish Remune Peach) before and after provided to the mice

Supplementary figure 1-29-4. Pictures from euthanization (11.09.19)

- It was not the xenograft, but an abscess growing.
  Abscess bursted and left a wound in the xenograft aerea

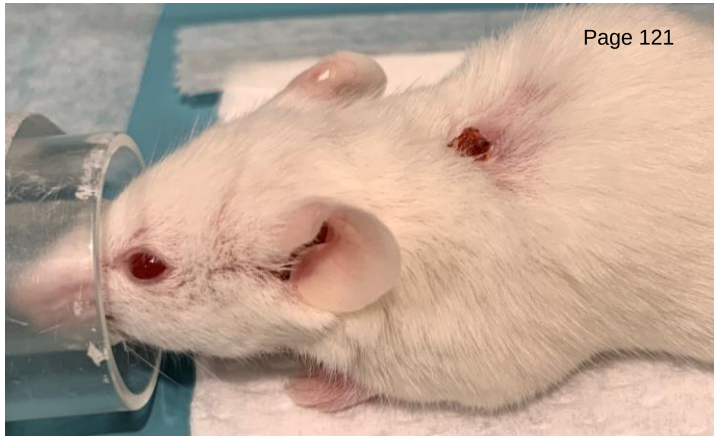

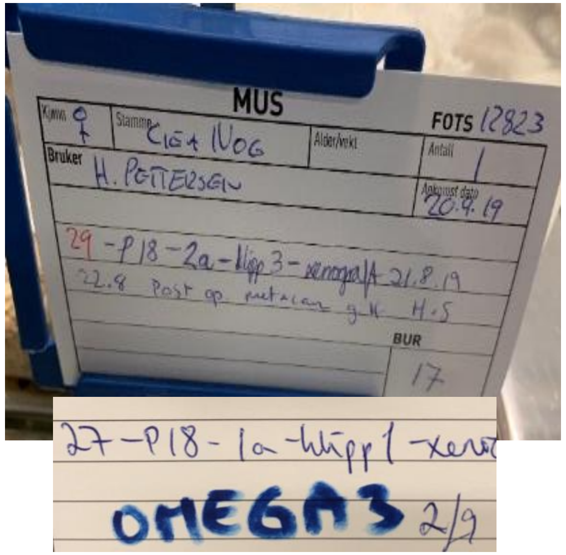

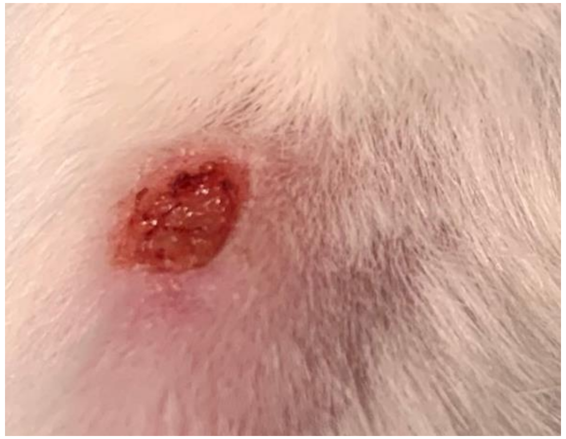

Wound with a «crater». The xenograft seems to be gone.

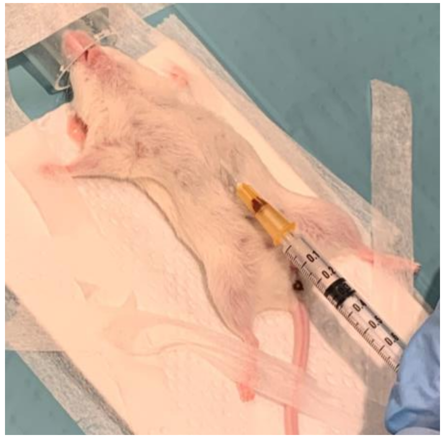

Blood drained from the heart before opening the chest and cutting the aorta. Gave a higher volume for the blood sample compared to sample from the back vain.

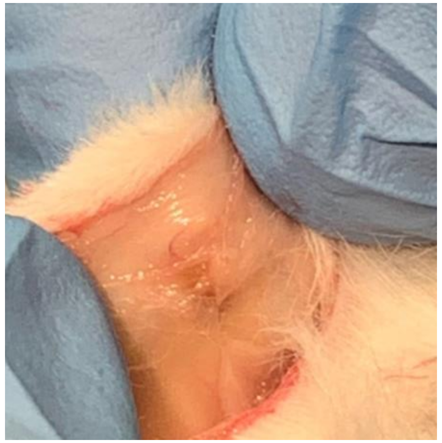

Only remainings of the xenograft was found.

Supplementary figure 1-29-5. Score sheet

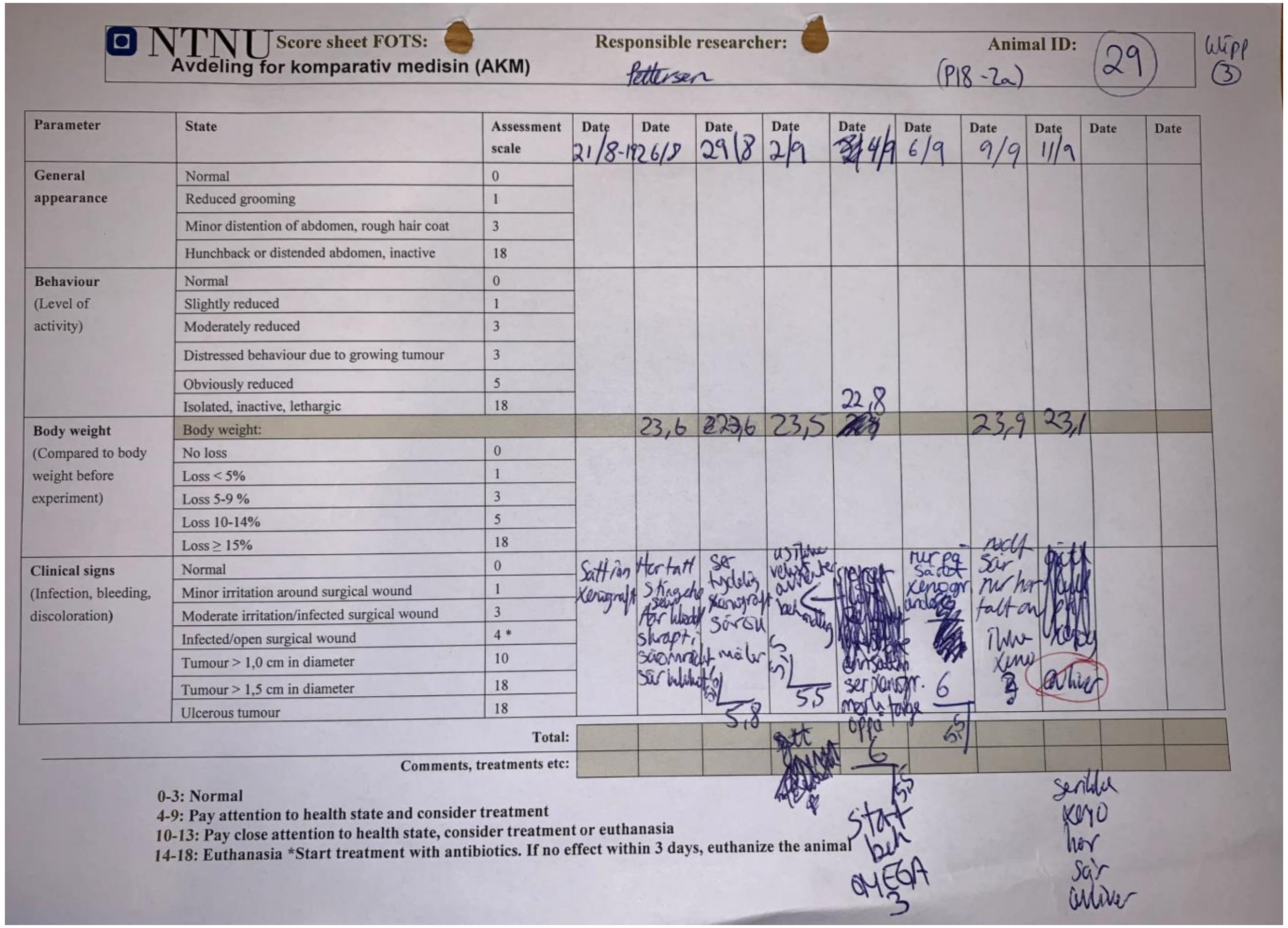

NTNU Score sheet FOTS: Responsible researcher: Pettersen Animal ID: (P18-2a) 29

Avdeling for komparativ medisin (AKM)

Parameter	State	Assessment scale	Date 21/8-19	Date 26/8	Date 29/8	Date 2/9	Date 4/9	Date 6/9	Date 9/9	Date 11/9	Date	Date
General appearance	Normal	0										
	Reduced grooming	1										
	Minor distention of abdomen, rough hair coat	3										
	Hunchback or distended abdomen, inactive	18										
Behaviour (Level of activity)	Normal	0										
	Slightly reduced	1										
	Moderately reduced	3										
	Distressed behaviour due to growing tumour	3										
	Obviously reduced	5										
	Isolated, inactive, lethargic	18										
Body weight (Compared to body weight before experiment)	Body weight:			23,6	23,6	23,5	22,8		23,9	23,1		
	No loss	0										
	Loss < 5%	1										
	Loss 5-9 %	3										
	Loss 10-14%	5										
	Loss ≥ 15%	18										
Clinical signs (Infection, bleeding, discoloration)	Normal	0	Satt inn xenograft	[illegible]	[illegible] 5,8	[illegible] 5,5	[illegible]	[illegible] 6	[illegible]	[illegible]		
	Minor irritation around surgical wound	1										
	Moderate irritation/infected surgical wound	3										
	Infected/open surgical wound	4 *										
	Tumour > 1,0 cm in diameter	10										
	Tumour > 1,5 cm in diameter	18										
	Ulcerous tumour	18										
Total:												
Comments, treatments etc:												

0-3: Normal
4-9: Pay attention to health state and consider treatment
10-13: Pay close attention to health state, consider treatment or euthanasia
14-18: Euthanasia *Start treatment with antibiotics. If no effect within 3 days, euthanize the animal

Start bruk OMEGA 3

Sendte KEYO her sår [illegible]

Abscess mistaken for rapidly growing xenograft

Supplementary figure 1-29-6.

Mouse and abscess growth curves

Weight curve

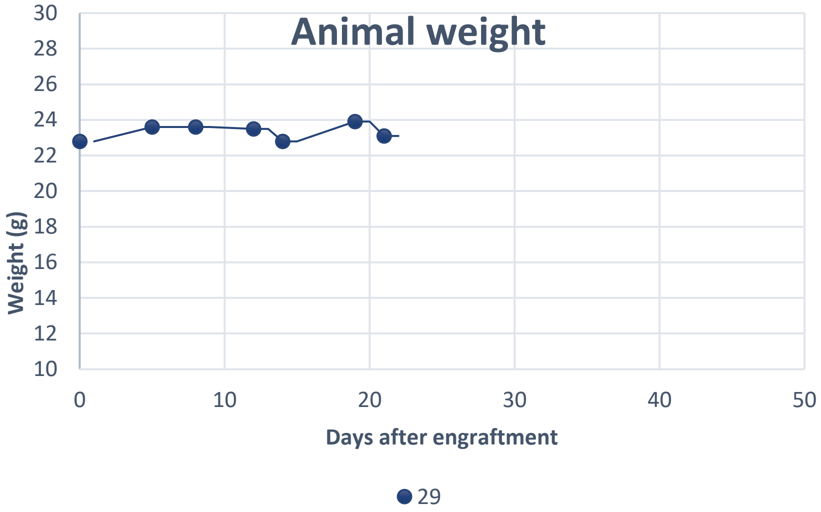

Size of abscess

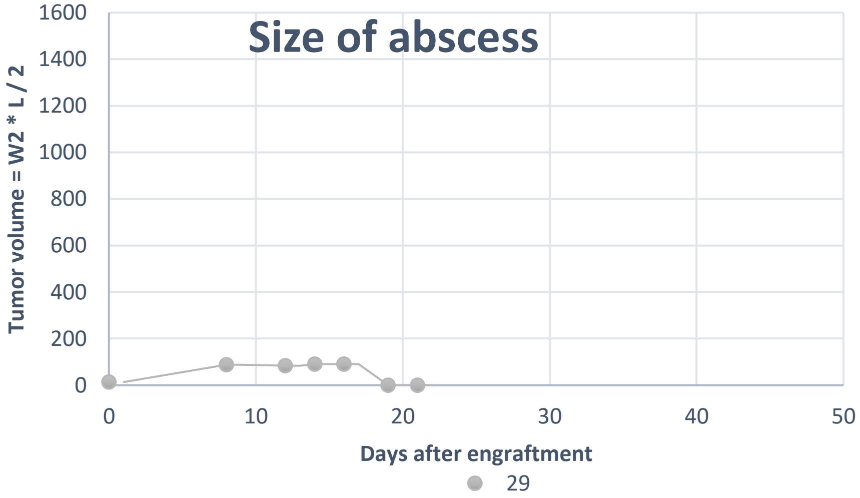

Mouse 30

- Surgery date: 21.08.19
  - Age (weeks) 31
  - Weight (g) 22.1
  - Tumor fragment P18-2b
  - Fragment size 3 x 3 mm
  - Ear mark 4

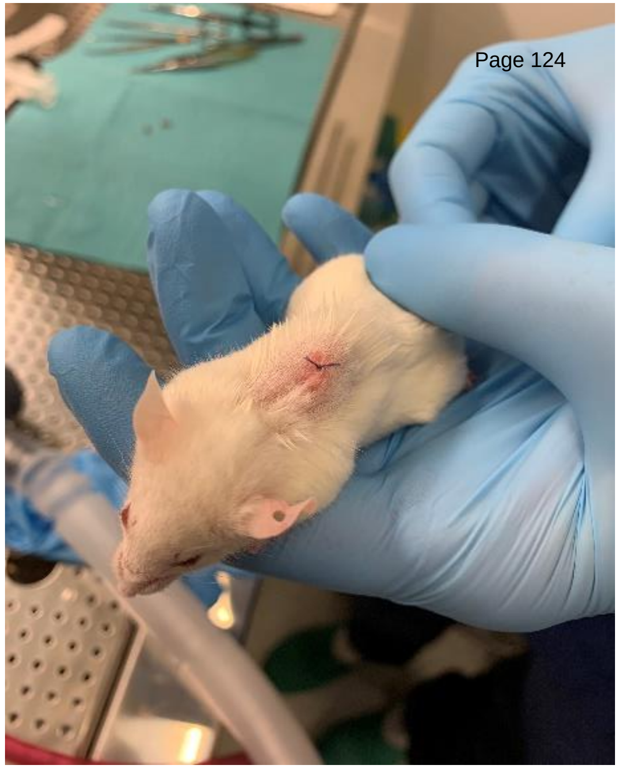

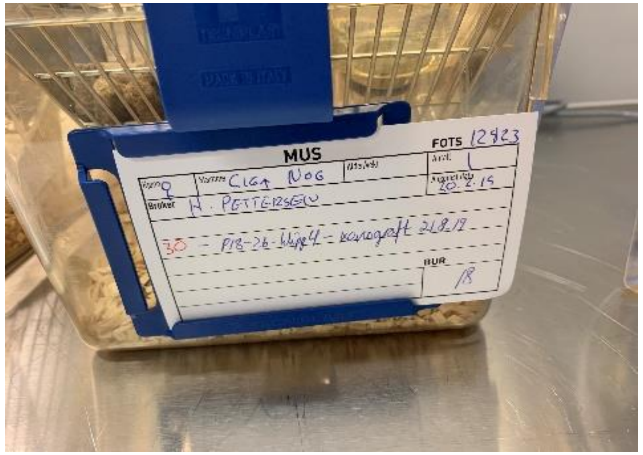

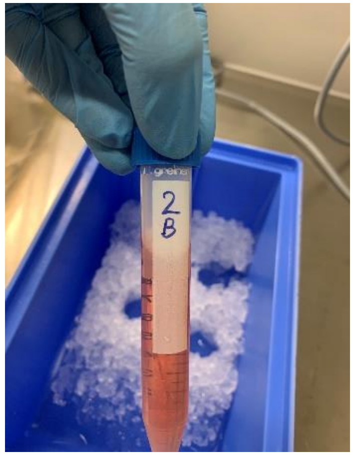

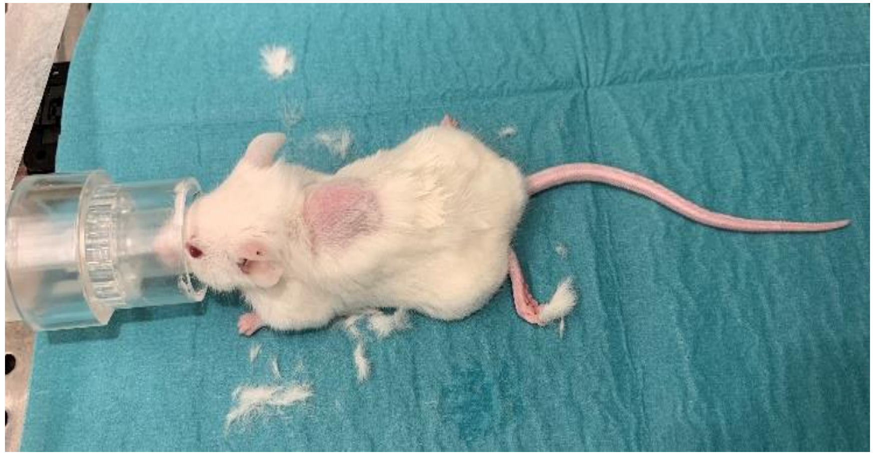

Supplementary figure 1-30-1. Pictures from the engraftment day

Supplementary figure 1-30-2. Pictures from follow up and xenograft size measurement

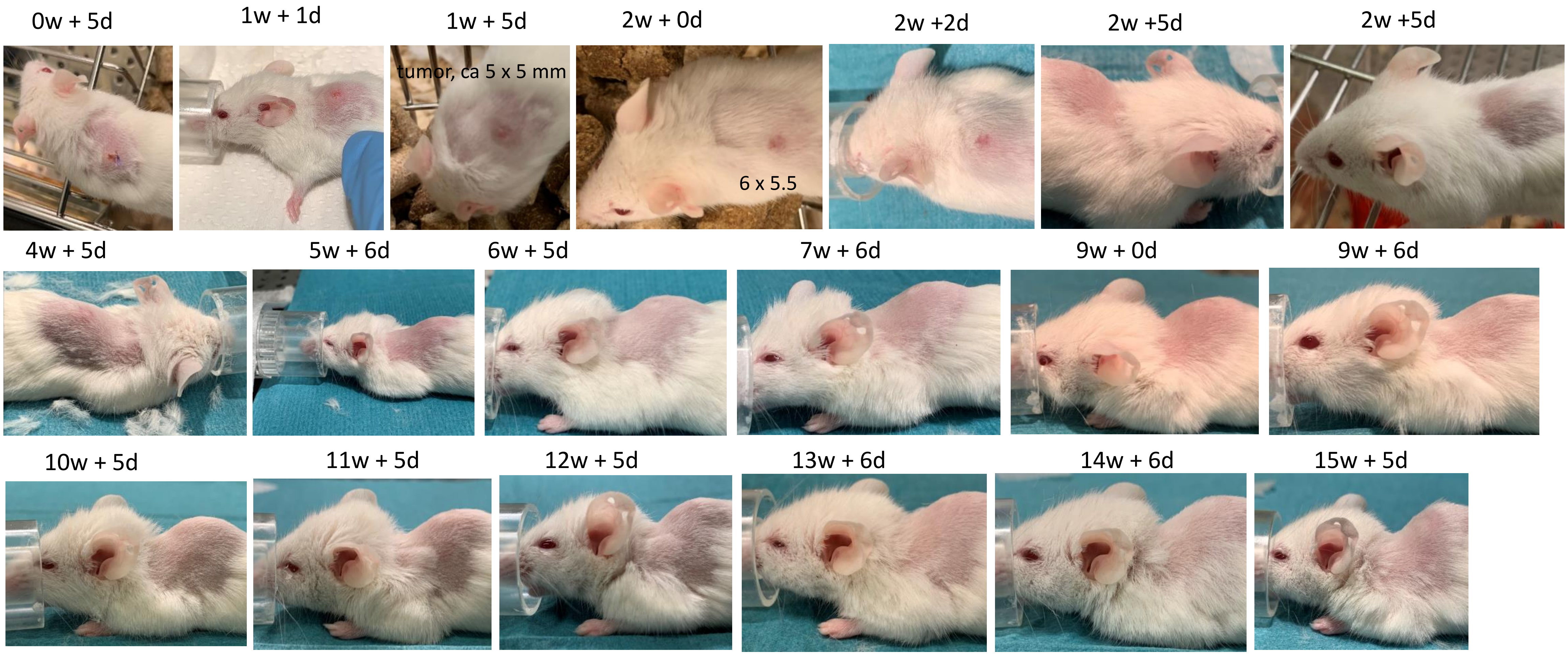

Supplementary figure 1-30-2. Pictures from follow up and xenograft size measurement

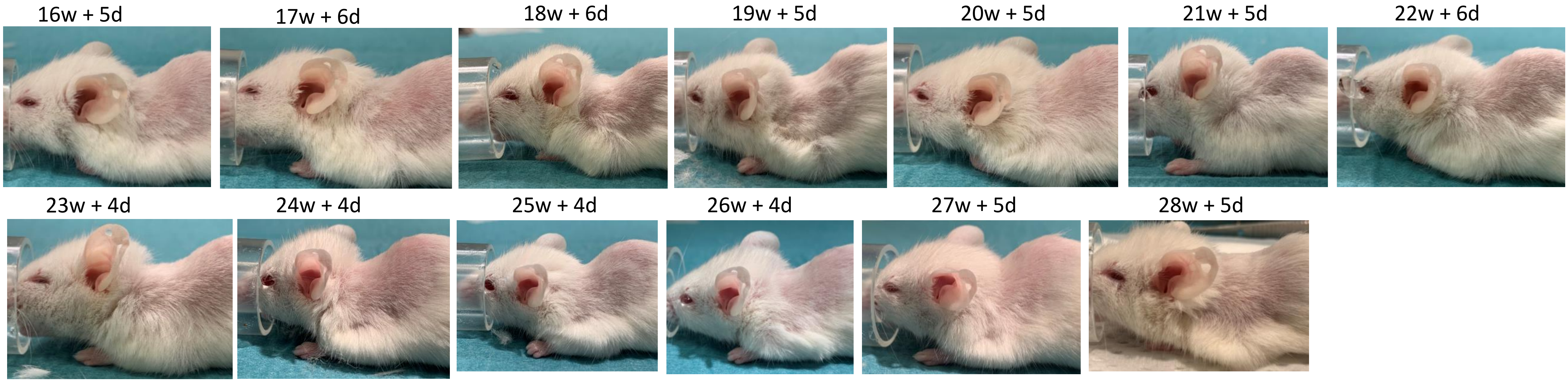

Supplementary figure 1-30-3. Pictures from euthanization (11.03.20)

- Xenograft size – not visiable
- No treatment given
- Small/normal spleen
- Possibly small tumor on liver
- Euthanized due to xenograft not growing after 6 months

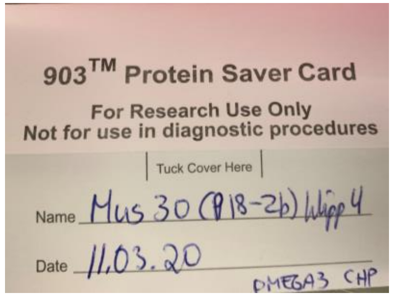

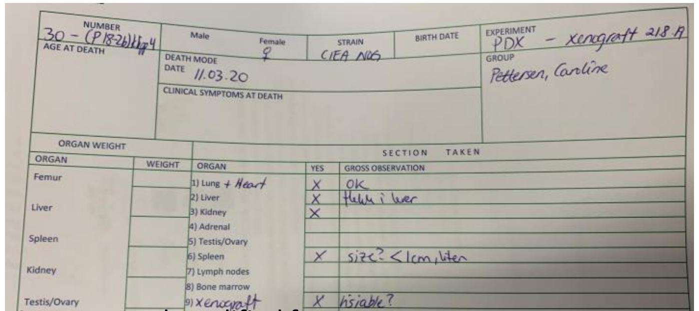

Autopsy card modified from:
http://www.pathbase.net/Necropsy_of_the_Mouse/

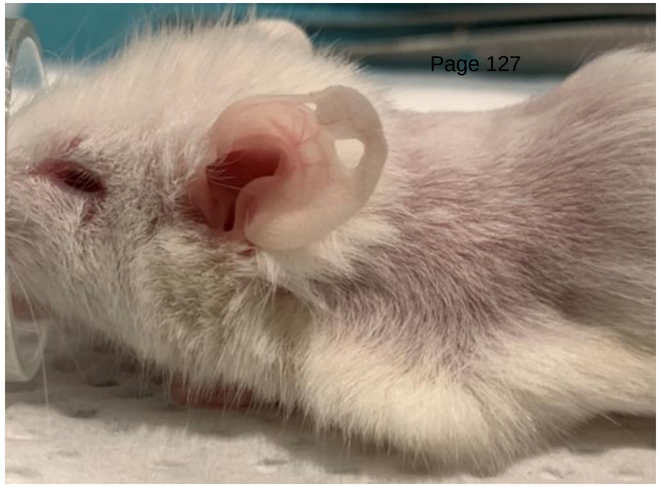

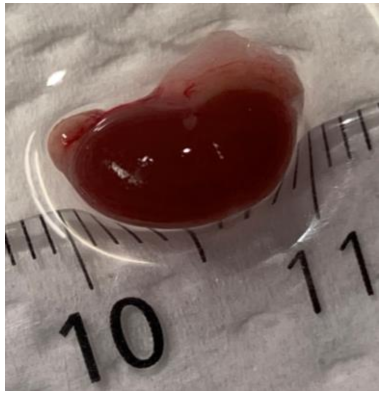

Normal kidney

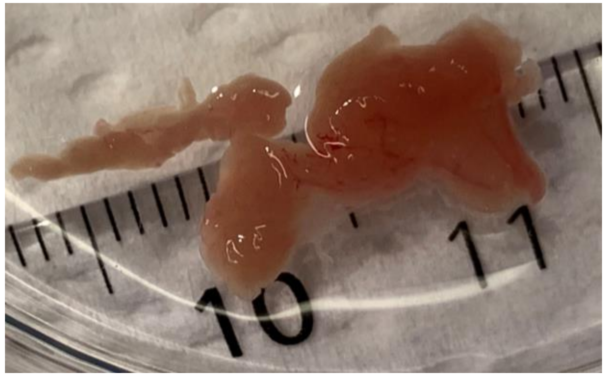

Pancreas

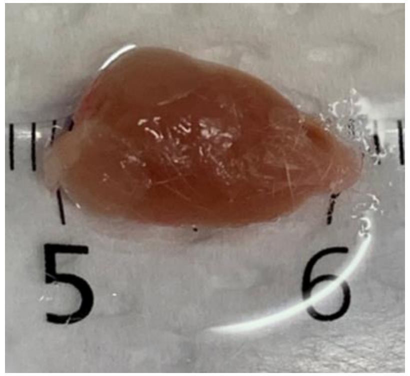

Thymus

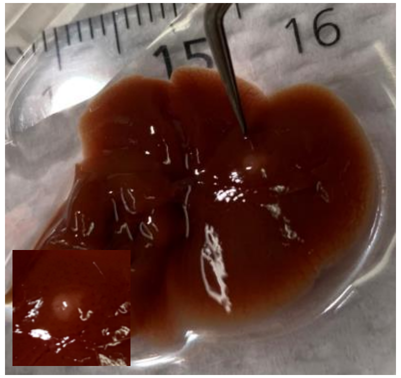

Liver with «bump» (tumor?)

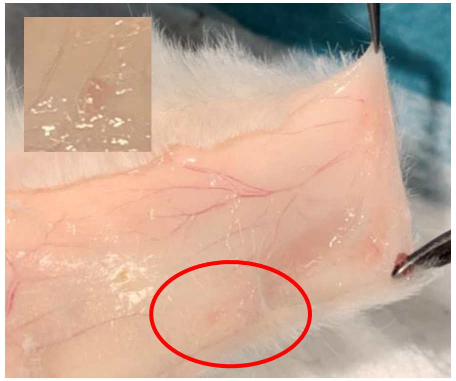

Only a small «bump» of xenograft

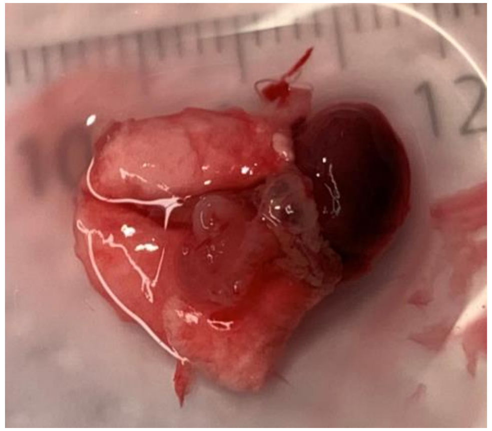

Normal heart and lungs

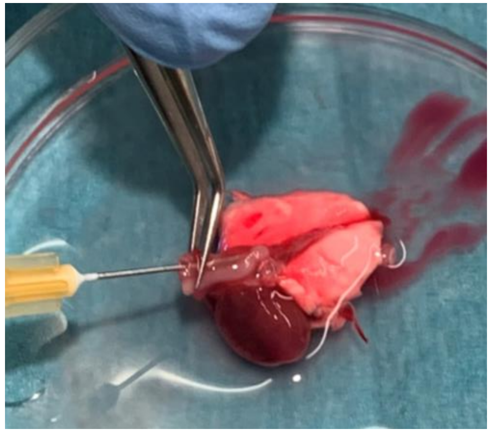

Lungs filled with formalin

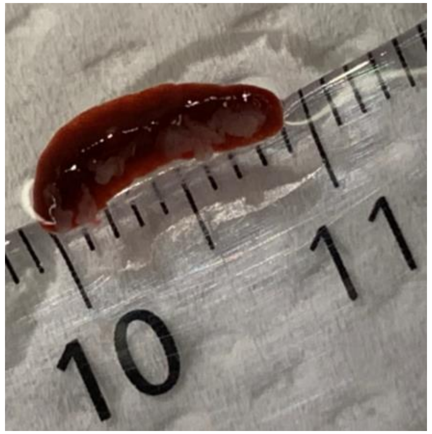

Normal spleen

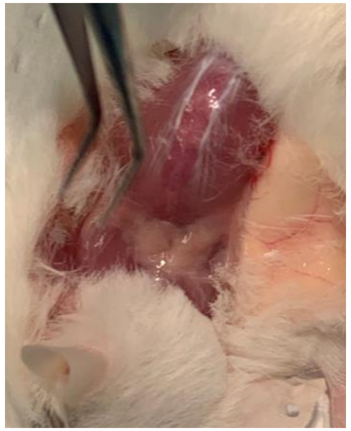

No tumors on shoulders

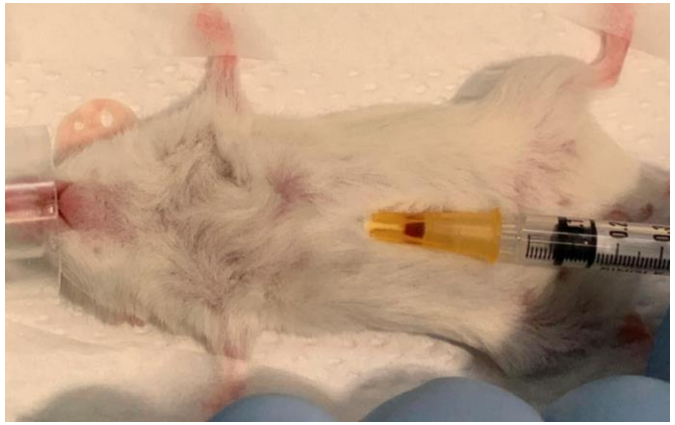

Blood sampling

Supplementary figure 1-30-4. Score sheets

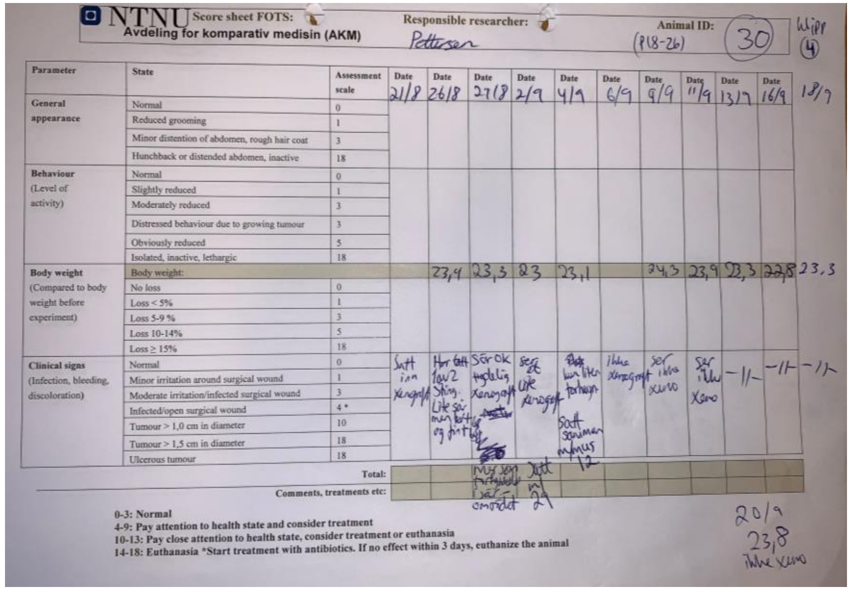

NTNU Score sheet FOTS: Avdeling for komparativ medisin (AKM) — Responsible researcher: Pettersen — Animal ID: (P18-26) 30 — Klipp (4)

Parameter	State	Assessment scale	Date 21/8	Date 26/8	Date 27/8	Date 2/9	Date 4/9	Date 6/9	Date 9/9	Date 11/9	Date 13/9	Date 16/9	18/9
General appearance	Normal	0											
	Reduced grooming	1											
	Minor distention of abdomen, rough hair coat	3											
	Hunchback or distended abdomen, inactive	18											
Behaviour (Level of activity)	Normal	0											
	Slightly reduced	1											
	Moderately reduced	3											
	Distressed behaviour due to growing tumour	3											
	Obviously reduced	5											
	Isolated, inactive, lethargic	18											
Body weight (Compared to body weight before experiment)	Body weight:			23,4	23,3	23	23,1		24,3	23,9	23,3	22,8	23,3
	No loss	0											
	Loss < 5%	1											
	Loss 5-9 %	3											
	Loss 10-14%	5											
	Loss ≥ 15%	18											
Clinical signs (Infection, bleeding, discoloration)	Normal	0	Satt inn xenograft	Har fått [illegible] sting. Lite sår men [illegible] og fint	Sår ok tydelig xenograft [illegible]	Ser ut lite xenograft	[illegible] litt forhøyn. Satt sammen m/mus 12	Ikke xenograft	Ser ikke xeno	Ser ikke Xeno	-//-	-//-	-//-
	Minor irritation around surgical wound	1											
	Moderate irritation/infected surgical wound	3											
	Infected/open surgical wound	4 *											
	Tumour > 1,0 cm in diameter	10											
	Tumour > 1,5 cm in diameter	18											
	Ulcerous tumour	18											
	Total:				Mus som [illegible] [illegible] området	Satt inn 29							
	Comments, treatments etc:												

0-3: Normal
4-9: Pay attention to health state and consider treatment
10-13: Pay close attention to health state, consider treatment or euthanasia
14-18: Euthanasia *Start treatment with antibiotics. If no effect within 3 days, euthanize the animal

20/9 23,8 Ikke xeno

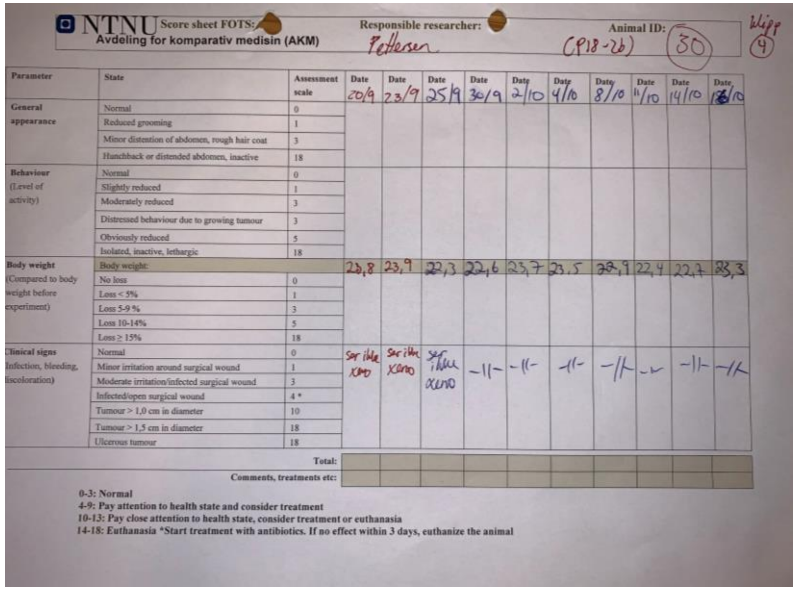

NTNU Score sheet FOTS: Avdeling for komparativ medisin (AKM) — Responsible researcher: Pettersen — Animal ID: (P18-26) 30 — Klipp (4)

Parameter	State	Assessment scale	Date 20/9	Date 23/9	Date 25/9	Date 30/9	Date 2/10	Date 4/10	Date 8/10	Date 11/10	Date 14/10	Date 16/10
General appearance	Normal	0										
	Reduced grooming	1										
	Minor distention of abdomen, rough hair coat	3										
	Hunchback or distended abdomen, inactive	18										
Behaviour (Level of activity)	Normal	0										
	Slightly reduced	1										
	Moderately reduced	3										
	Distressed behaviour due to growing tumour	3										
	Obviously reduced	5										
	Isolated, inactive, lethargic	18										
Body weight (Compared to body weight before experiment)	Body weight:		23,8	23,9	22,3	22,6	23,7	23,5	22,9	22,4	22,7	23,3
	No loss	0										
	Loss < 5%	1										
	Loss 5-9 %	3										
	Loss 10-14%	5										
	Loss ≥ 15%	18										
Clinical signs (Infection, bleeding, discoloration)	Normal	0	Ser ikke xeno	Ser ikke xeno	Ser ikke xeno	-//-	-//-	-//-	-//-	-//-	-//-	-//-
	Minor irritation around surgical wound	1										
	Moderate irritation/infected surgical wound	3										
	Infected/open surgical wound	4 *										
	Tumour > 1,0 cm in diameter	10										
	Tumour > 1,5 cm in diameter	18										
	Ulcerous tumour	18										
	Total:											
	Comments, treatments etc:											

0-3: Normal
4-9: Pay attention to health state and consider treatment
10-13: Pay close attention to health state, consider treatment or euthanasia
14-18: Euthanasia *Start treatment with antibiotics. If no effect within 3 days, euthanize the animal

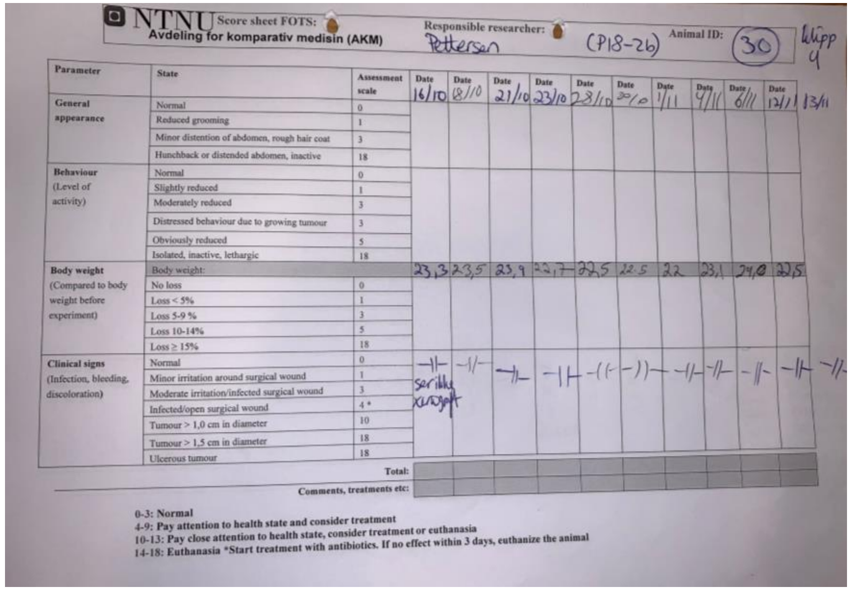

NTNU Score sheet FOTS: Avdeling for komparativ medisin (AKM) — Responsible researcher: Pettersen — (P18-26) Animal ID: 30 — Klipp 4

Parameter	State	Assessment scale	Date 16/10	Date 18/10	Date 21/10	Date 23/10	Date 28/10	Date 30/10	Date 1/11	Date 4/11	Date 6/11	Date 12/11	13/11
General appearance	Normal	0											
	Reduced grooming	1											
	Minor distention of abdomen, rough hair coat	3											
	Hunchback or distended abdomen, inactive	18											
Behaviour (Level of activity)	Normal	0											
	Slightly reduced	1											
	Moderately reduced	3											
	Distressed behaviour due to growing tumour	3											
	Obviously reduced	5											
	Isolated, inactive, lethargic	18											
Body weight (Compared to body weight before experiment)	Body weight:		23,3	23,5	23,9	22,7	22,5	22.5	22	23,1	24,0	22,5	
	No loss	0											
	Loss < 5%	1											
	Loss 5-9 %	3											
	Loss 10-14%	5											
	Loss ≥ 15%	18											
Clinical signs (Infection, bleeding, discoloration)	Normal	0	-//- ser ikke xenograft	-//-	-//-	-//-	-//-	-//-	-//-	-//-	-//-	-//-	-//-
	Minor irritation around surgical wound	1											
	Moderate irritation/infected surgical wound	3											
	Infected/open surgical wound	4 *											
	Tumour > 1,0 cm in diameter	10											
	Tumour > 1,5 cm in diameter	18											
	Ulcerous tumour	18											
	Total:												
	Comments, treatments etc:												

0-3: Normal
4-9: Pay attention to health state and consider treatment
10-13: Pay close attention to health state, consider treatment or euthanasia
14-18: Euthanasia *Start treatment with antibiotics. If no effect within 3 days, euthanize the animal

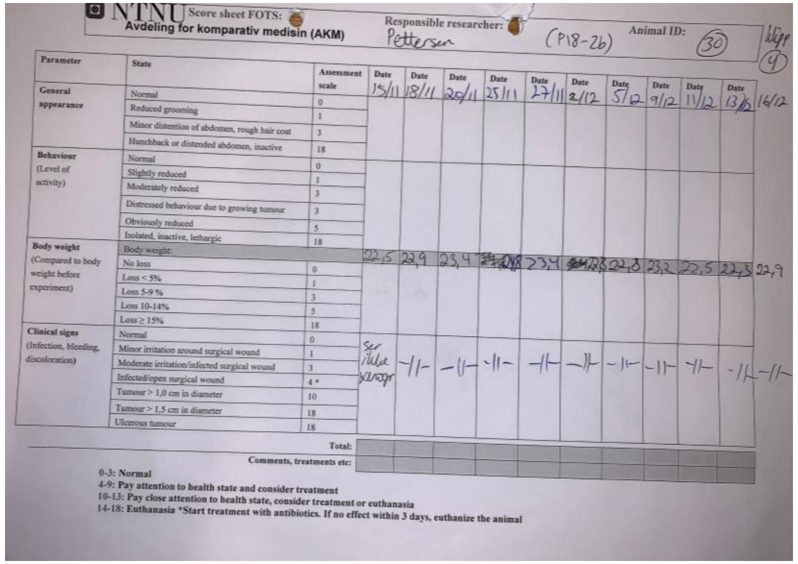

NTNU Score sheet FOTS: Avdeling for komparativ medisin (AKM) — Responsible researcher: Pettersen — (P18-26) Animal ID: 30 — Klipp (4)

Parameter	State	Assessment scale	Date 15/11	Date 18/11	Date 20/11	Date 25/11	Date 27/11	Date 2/12	Date 5/12	Date 9/12	Date 11/12	Date 13/12	16/12
General appearance	Normal	0											
	Reduced grooming	1											
	Minor distention of abdomen, rough hair coat	3											
	Hunchback or distended abdomen, inactive	18											
Behaviour (Level of activity)	Normal	0											
	Slightly reduced	1											
	Moderately reduced	3											
	Distressed behaviour due to growing tumour	3											
	Obviously reduced	5											
	Isolated, inactive, lethargic	18											
Body weight (Compared to body weight before experiment)	Body weight:		22,5	22,9	23,4	[illegible]	23,4	[illegible]	22,8	23,2	22,5	22,3	22,9
	No loss	0											
	Loss < 5%	1											
	Loss 5-9 %	3											
	Loss 10-14%	5											
	Loss ≥ 15%	18											
Clinical signs (Infection, bleeding, discoloration)	Normal	0	Ser ikke xenogr	-//-	-//-	-//-	-//-	-//-	-//-	-//-	-//-	-//-	-//-
	Minor irritation around surgical wound	1											
	Moderate irritation/infected surgical wound	3											
	Infected/open surgical wound	4 *											
	Tumour > 1,0 cm in diameter	10											
	Tumour > 1,5 cm in diameter	18											
	Ulcerous tumour	18											
	Total:												
	Comments, treatments etc:												

0-3: Normal
4-9: Pay attention to health state and consider treatment
10-13: Pay close attention to health state, consider treatment or euthanasia
14-18: Euthanasia *Start treatment with antibiotics. If no effect within 3 days, euthanize the animal

Supplementary figure 1-30-4. Score sheets

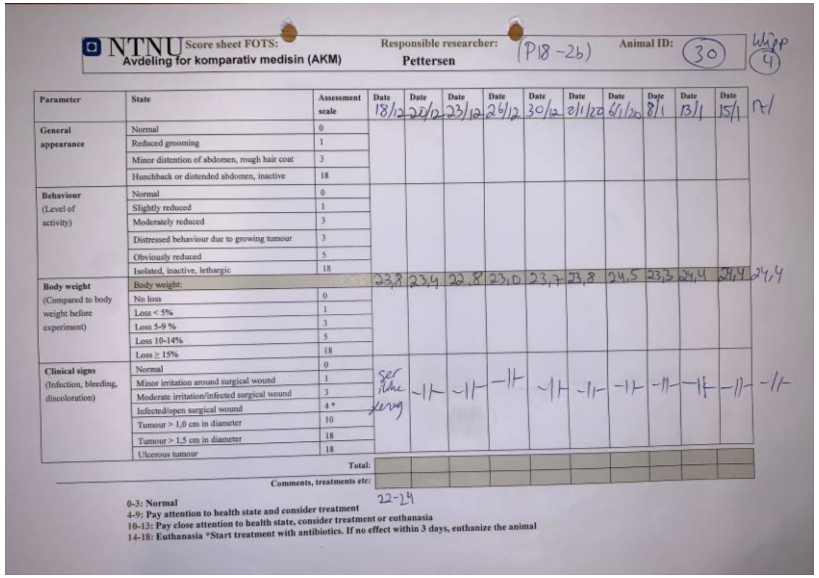

NTNU Score sheet FOTS: Avdeling for komparativ medisin (AKM) — Responsible researcher: Pettersen (P18-2b) — Animal ID: 30 — Klipp 4

Parameter	State	Assessment scale	Date 18/12	Date 20/12	Date 23/12	Date 26/12	Date 30/12	Date 2/1/20	Date 6/1/20	Date 8/1	Date 13/1	Date 15/1	[illegible]
General appearance	Normal	0											
	Reduced grooming	1											
	Minor distention of abdomen, rough hair coat	3											
	Hunchback or distended abdomen, inactive	18											
Behaviour (Level of activity)	Normal	0											
	Slightly reduced	1											
	Moderately reduced	3											
	Distressed behaviour due to growing tumour	3											
	Obviously reduced	5											
	Isolated, inactive, lethargic	18											
Body weight (Compared to body weight before experiment)	Body weight:		23,8	23,4	22,8	23,0	23,7	23,8	24,5	23,3	24,4	24,4	24,4
	No loss	0											
	Loss < 5%	1											
	Loss 5-9 %	3											
	Loss 10-14%	5											
	Loss ≥ 15%	18											
Clinical signs (Infection, bleeding, discoloration)	Normal	0	ser ikke xenog	-ll-	-ll-	-ll-	-ll-	-ll-	-ll-	-ll-	-ll-	-ll-	-ll-
	Minor irritation around surgical wound	1											
	Moderate irritation/infected surgical wound	3											
	Infected/open surgical wound	4 *											
	Tumour > 1,0 cm in diameter	10											
	Tumour > 1,5 cm in diameter	18											
	Ulcerous tumour	18											
		Total:											
		Comments, treatments etc:											

22-24

0-3: Normal
4-9: Pay attention to health state and consider treatment
10-13: Pay close attention to health state, consider treatment or euthanasia
14-18: Euthanasia *Start treatment with antibiotics. If no effect within 3 days, euthanize the animal

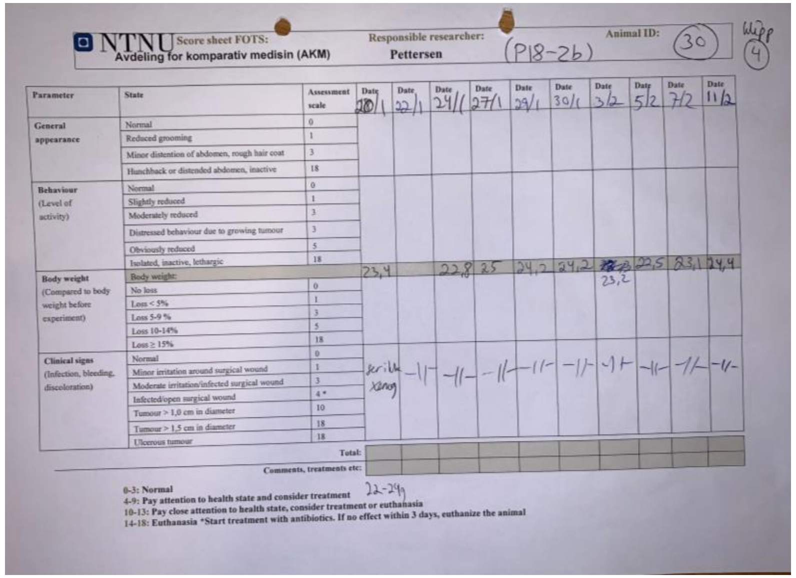

NTNU Score sheet FOTS: Avdeling for komparativ medisin (AKM) — Responsible researcher: Pettersen (P18-2b) — Animal ID: 30 — Klipp 4

Parameter	State	Assessment scale	Date 20/1	Date 22/1	Date 24/1	Date 27/1	Date 29/1	Date 30/1	Date 3/2	Date 5/2	Date 7/2	Date 11/2
General appearance	Normal	0										
	Reduced grooming	1										
	Minor distention of abdomen, rough hair coat	3										
	Hunchback or distended abdomen, inactive	18										
Behaviour (Level of activity)	Normal	0										
	Slightly reduced	1										
	Moderately reduced	3										
	Distressed behaviour due to growing tumour	3										
	Obviously reduced	5										
	Isolated, inactive, lethargic	18										
Body weight (Compared to body weight before experiment)	Body weight:		23,4		22,8	25	24,2	24,2	~~[illegible]~~ 23,2	22,5	23,1	24,4
	No loss	0										
	Loss < 5%	1										
	Loss 5-9 %	3										
	Loss 10-14%	5										
	Loss ≥ 15%	18										
Clinical signs (Infection, bleeding, discoloration)	Normal	0	ser ikke xenog	-ll-	-ll-	-ll-	-ll-	-ll-	-ll-	-ll-	-ll-	-ll-
	Minor irritation around surgical wound	1										
	Moderate irritation/infected surgical wound	3										
	Infected/open surgical wound	4 *										
	Tumour > 1,0 cm in diameter	10										
	Tumour > 1,5 cm in diameter	18										
	Ulcerous tumour	18										
		Total:										
		Comments, treatments etc:										

22-24g

0-3: Normal
4-9: Pay attention to health state and consider treatment
10-13: Pay close attention to health state, consider treatment or euthanasia
14-18: Euthanasia *Start treatment with antibiotics. If no effect within 3 days, euthanize the animal

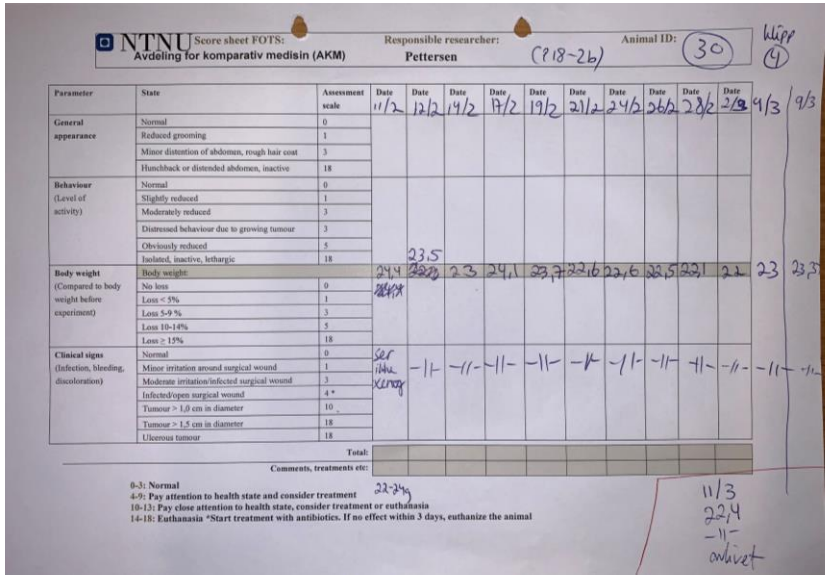

NTNU Score sheet FOTS: Avdeling for komparativ medisin (AKM) — Responsible researcher: Pettersen (P18-2b) — Animal ID: 30 — Klipp 4

Parameter	State	Assessment scale	Date 11/2	Date 12/2	Date 14/2	Date 17/2	Date 19/2	Date 21/2	Date 24/2	Date 26/2	Date 28/2	Date 2/3	4/3	9/3
General appearance	Normal	0												
	Reduced grooming	1												
	Minor distention of abdomen, rough hair coat	3												
	Hunchback or distended abdomen, inactive	18												
Behaviour (Level of activity)	Normal	0												
	Slightly reduced	1												
	Moderately reduced	3												
	Distressed behaviour due to growing tumour	3												
	Obviously reduced	5												
	Isolated, inactive, lethargic	18		23,5										
Body weight (Compared to body weight before experiment)	Body weight:		24,4 ~~[illegible]~~	~~[illegible]~~	23	24,1	23,7	22,6	22,6	22,5	22,1	22	23	23,5
	No loss	0												
	Loss < 5%	1												
	Loss 5-9 %	3												
	Loss 10-14%	5												
	Loss ≥ 15%	18												
Clinical signs (Infection, bleeding, discoloration)	Normal	0	ser ikke xenog	-ll-	-ll-	-ll-	-ll-	-ll-	-ll-	-ll-	-ll-	-ll-	-ll-	-ll-
	Minor irritation around surgical wound	1												
	Moderate irritation/infected surgical wound	3												
	Infected/open surgical wound	4 *												
	Tumour > 1,0 cm in diameter	10												
	Tumour > 1,5 cm in diameter	18												
	Ulcerous tumour	18												
		Total:												
		Comments, treatments etc:												

22-24g

0-3: Normal
4-9: Pay attention to health state and consider treatment
10-13: Pay close attention to health state, consider treatment or euthanasia
14-18: Euthanasia *Start treatment with antibiotics. If no effect within 3 days, euthanize the animal

11/3
22,4
-ll-
avlivet

Supplementary figure 1-30-5.

Mouse and xenograft growth curves

Weight curve

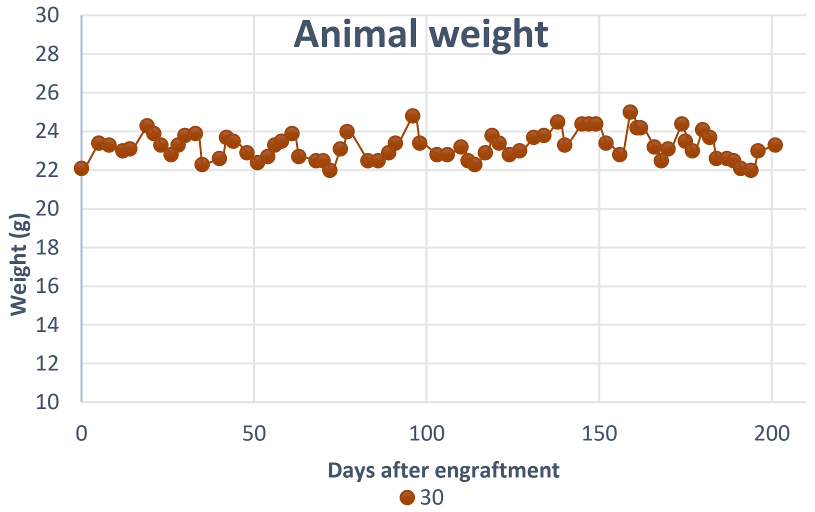

Xenograft growth curve

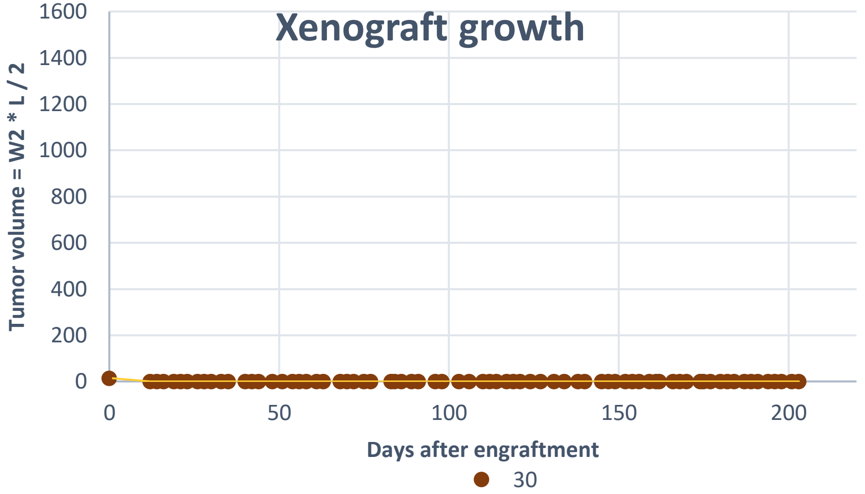

Supplementary figure 1-30-6. Histology pictures

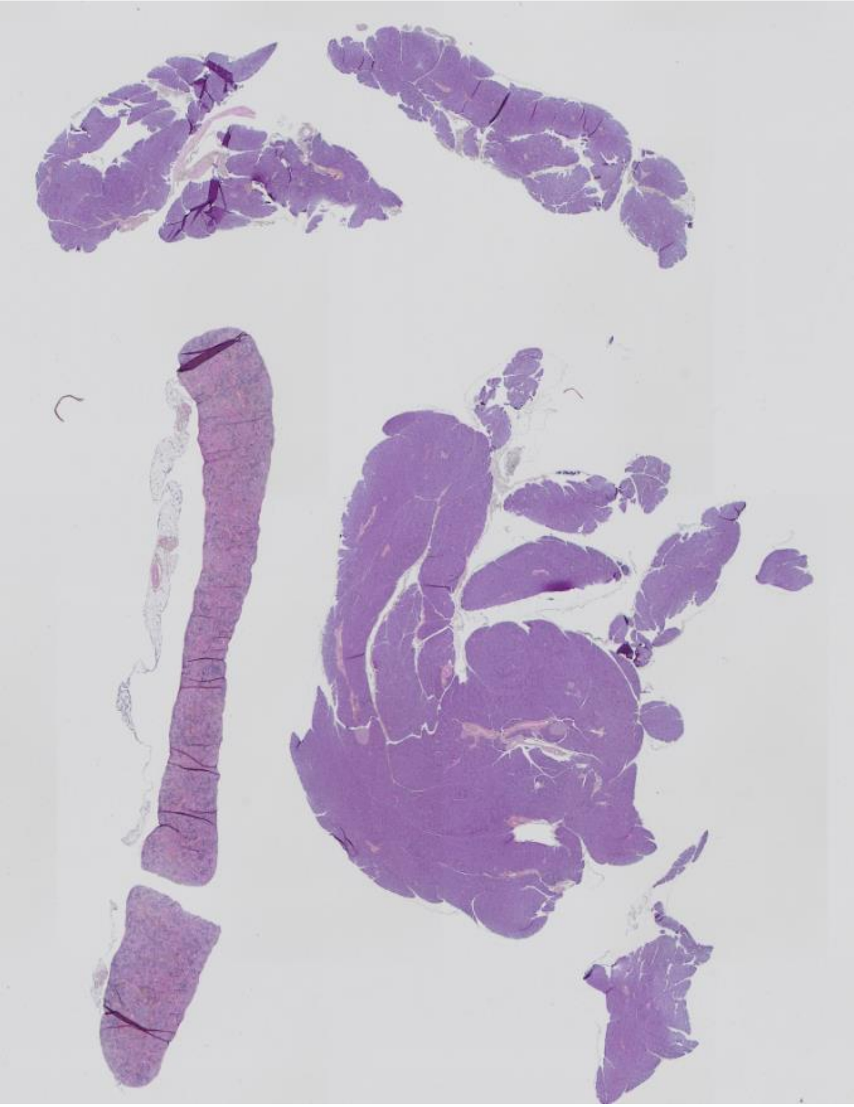

M30MP – Spleen and pancreas

- Look normal

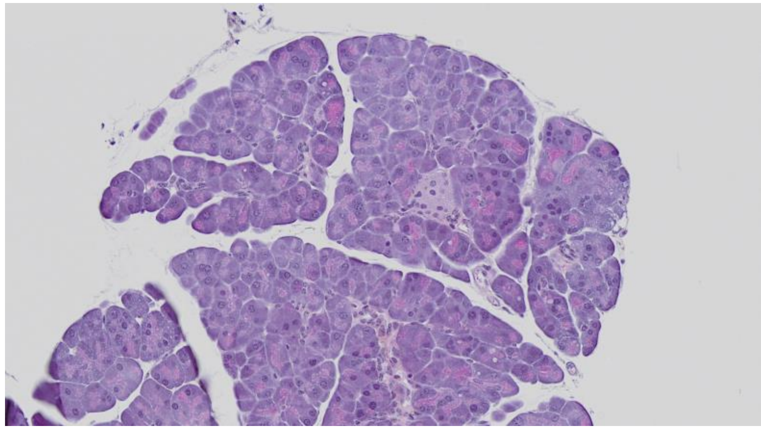

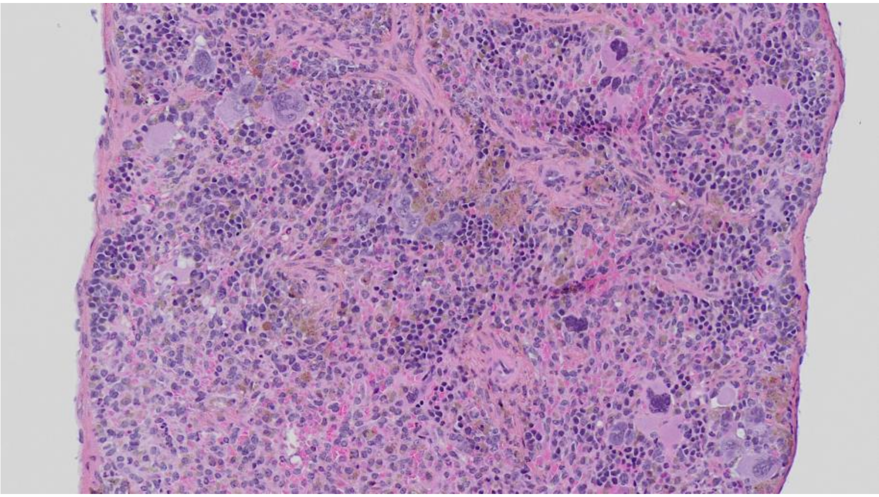

Supplementary figure 1-30-6. Histology pictures

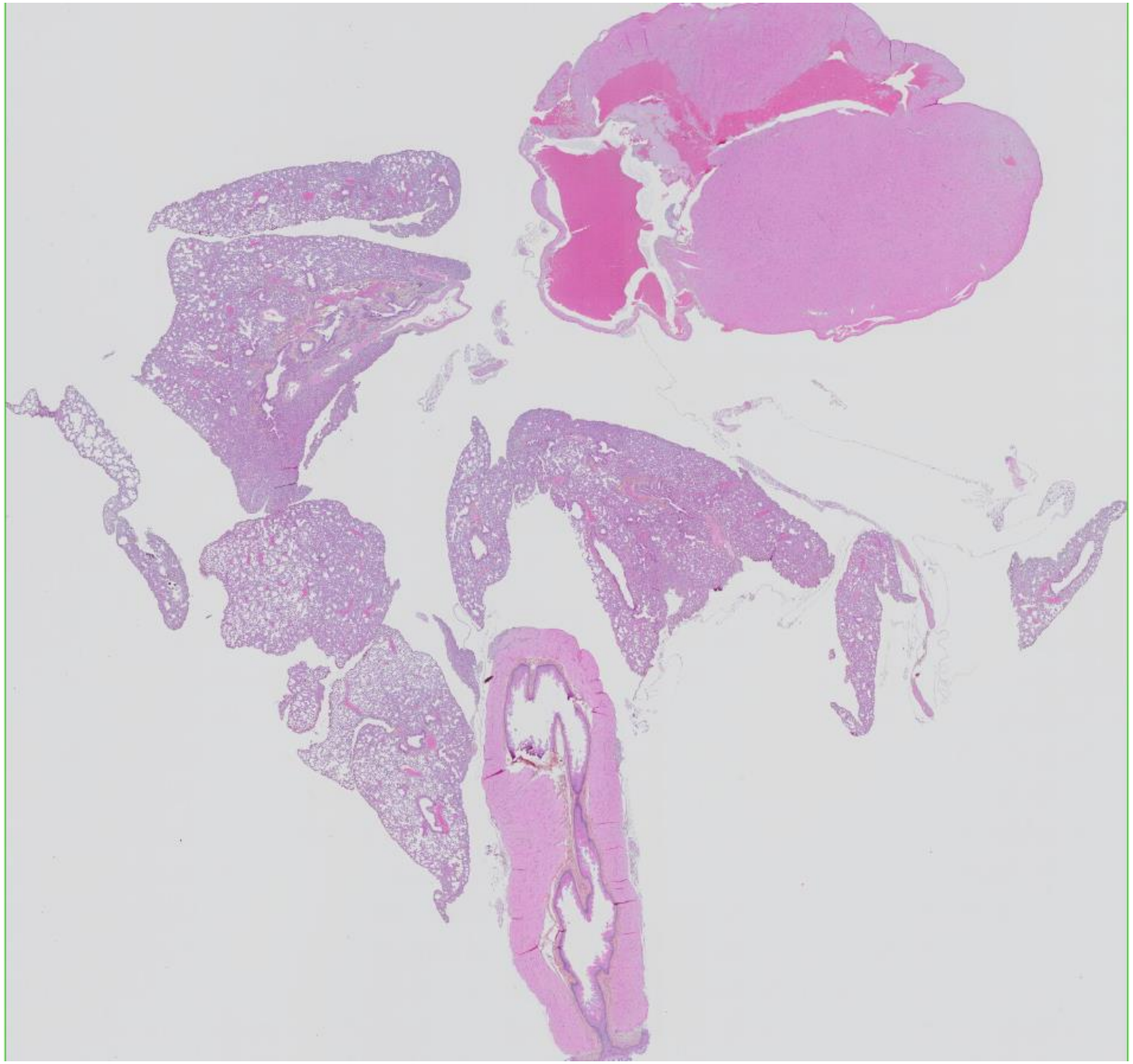

M30LH – Lungs and heart

- Look normal

Mouse 31

- Surgery date: 03.09.19
  - Age (weeks) 33
  - Weight (g) 23.8
  - Tumor fragment P19-1a
  - Fragment size 3 x 3 mm
  - Ear marks 1

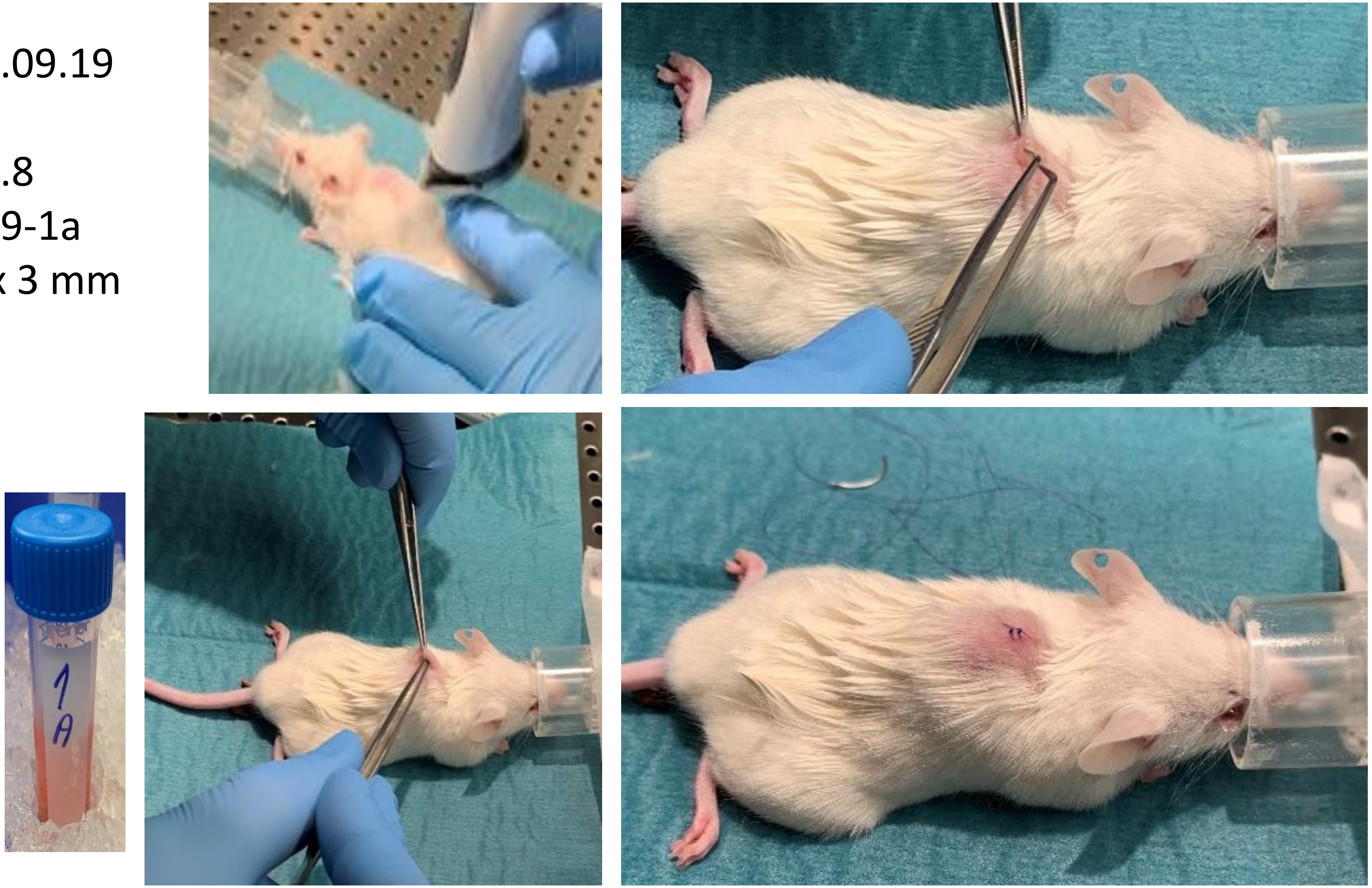

Supplementary figure 1-31-1. Pictures from the engraftment day

Supplementary figure 1-31-2. Pictures from follow up and xenograft size measurement

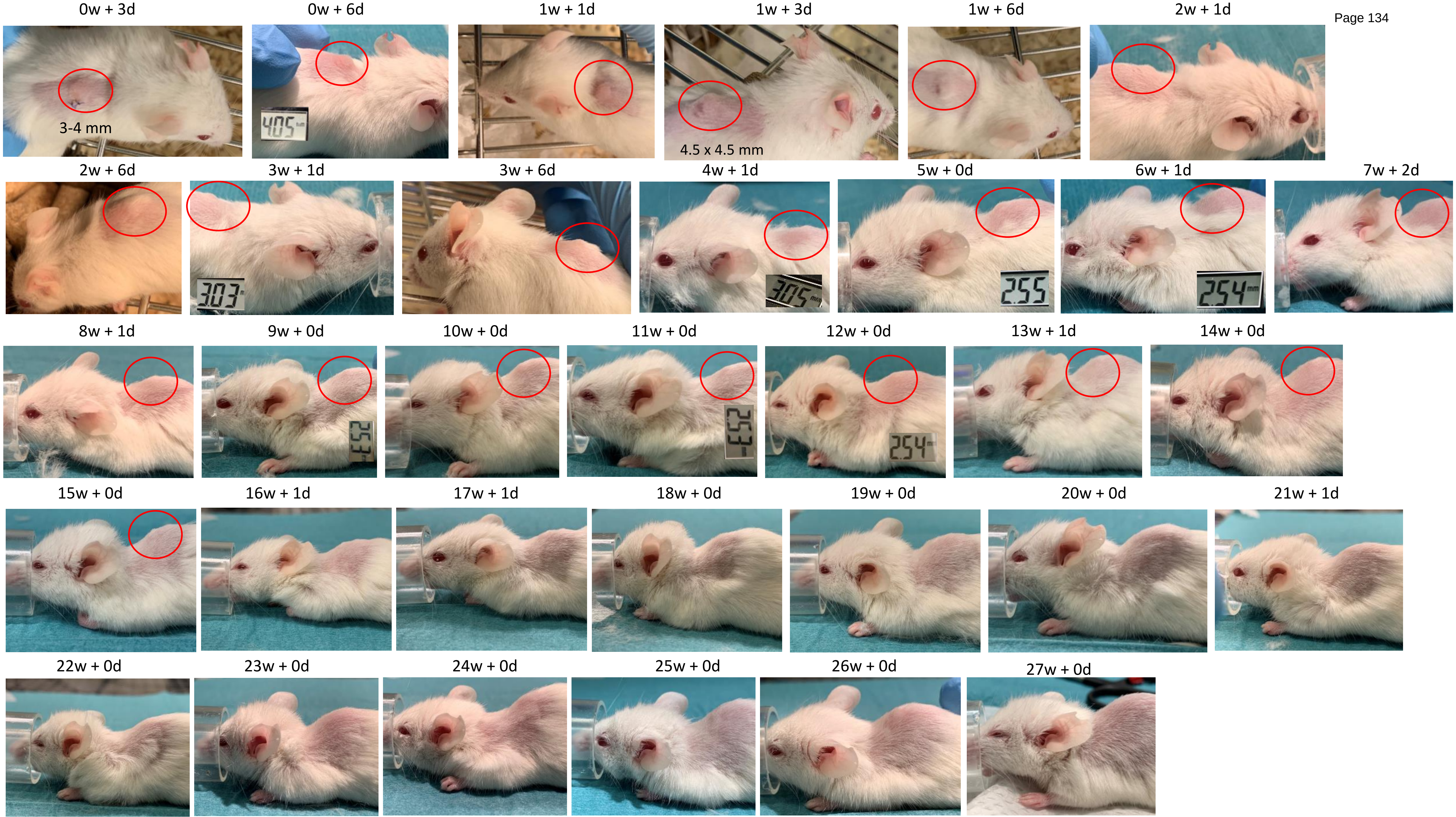

Supplementary figure 1-31-3. Pictures from euthanization (11.03.20)

- No treatment given (no zenograft growth)
- Xenograft size – not visiable
- Small/normal spleen
- Euthanized due to xenograft not growing after 6 months

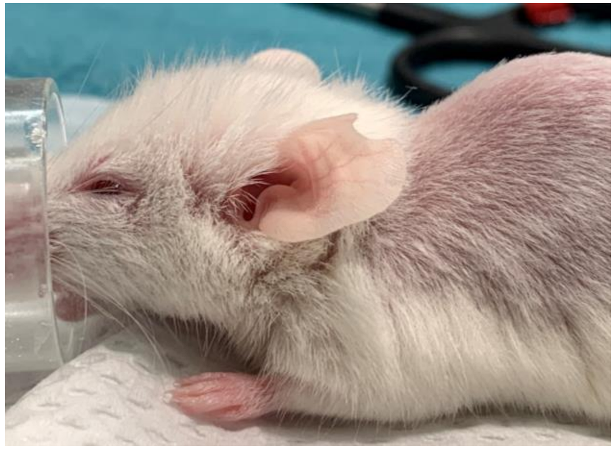

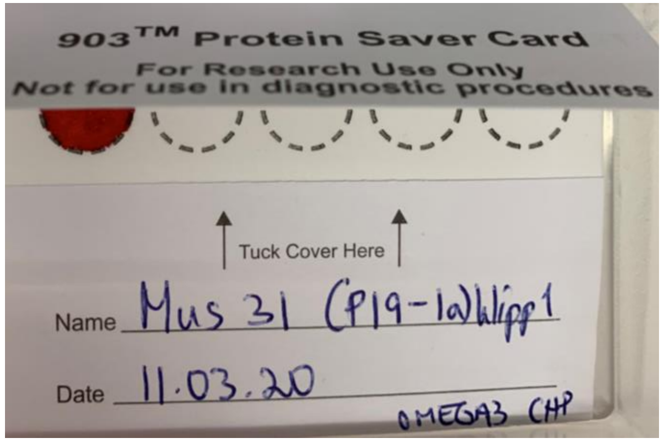

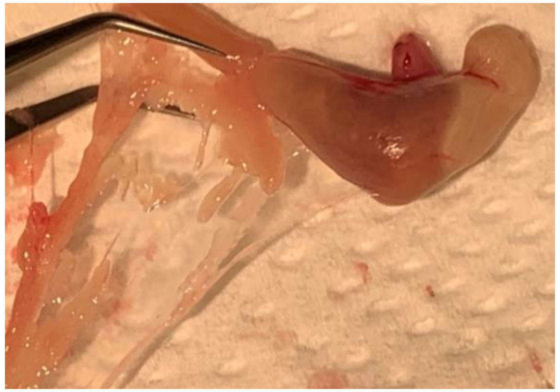
Stomach and pancreatic tissue

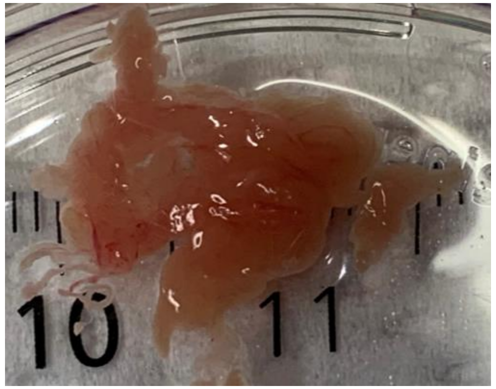
Pancreas

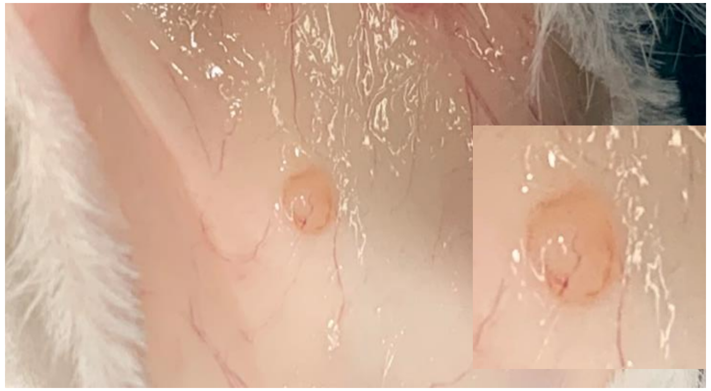
Small xenograft under the skin

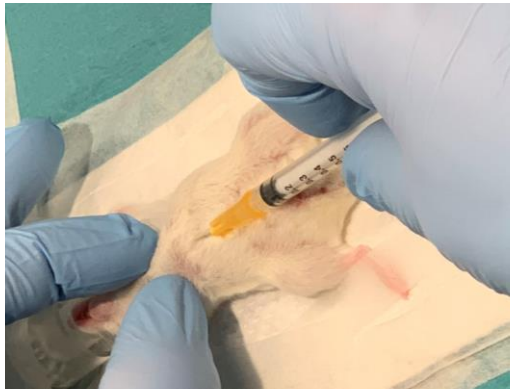
Blood sampling

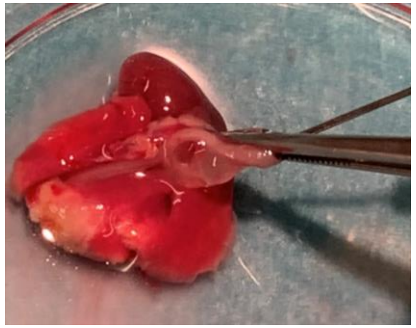
Lungs filled with formalin

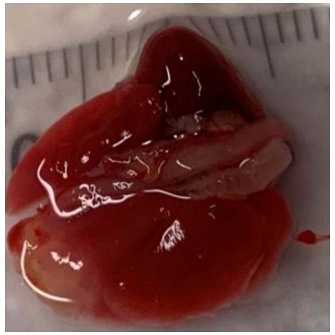
Normal lungs and heart

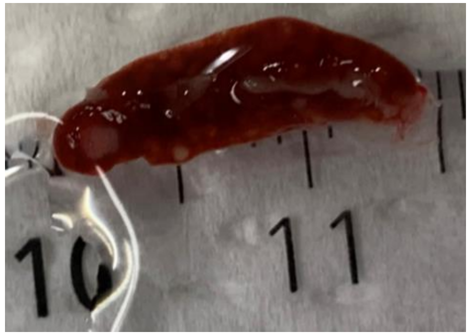
Normal spleen

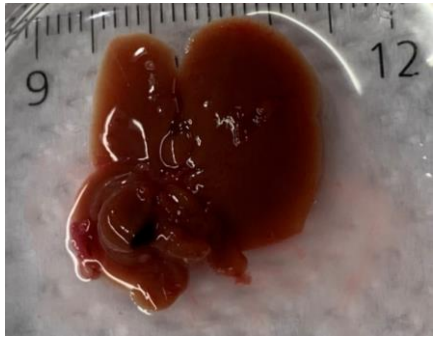
Normal liver

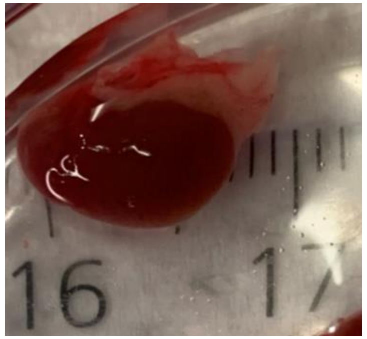
Normal kidney

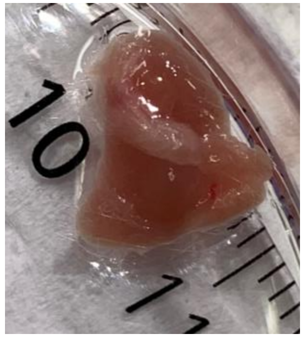
Thymus

Supplementary figure 1-31-4. Autopsy card filled out during necroscopy

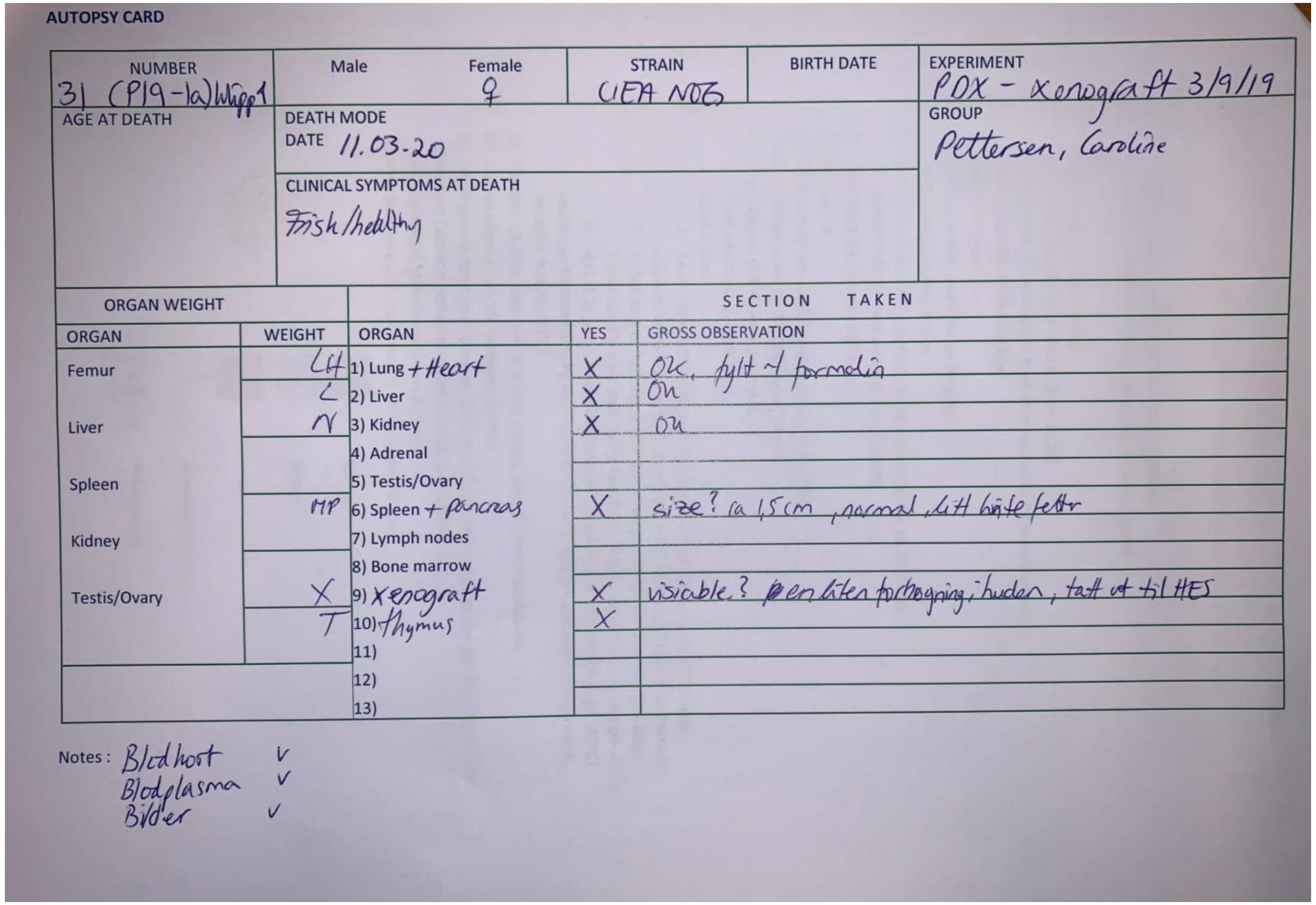

AUTOPSY CARD

NUMBER	Male	Female	STRAIN	BIRTH DATE	EXPERIMENT
31 (P19-1a) Whipp1		♀	UEA NOG		PDX - Xenograft 3/9/19

AGE AT DEATH

DEATH MODE
DATE 11.03.20

CLINICAL SYMPTOMS AT DEATH
Frisk/healthy

GROUP
Pettersen, Caroline

ORGAN WEIGHT	
ORGAN	WEIGHT
Femur	LH
	L
Liver	N
Spleen	MP
Kidney	
	X
Testis/Ovary	T

SECTION TAKEN

ORGAN	YES	GROSS OBSERVATION
1) Lung + Heart	X	OK, fylt m/ formalin
2) Liver	X	OK
3) Kidney	X	OK
4) Adrenal		
5) Testis/Ovary		
6) Spleen + pancreas	X	size? ca 1,5 cm, normal, litt hvite felter
7) Lymph nodes		
8) Bone marrow		
9) Xenograft	X	visiable? en liten forhøyning i huden, tatt ut til HES
10) thymus	X	
11)		
12)		
13)		

Notes : Blodkort ✓
Blodplasma ✓
Bilder ✓

Autopsy card modified from: http://www.pathbase.net/Necropsy_of_the_Mouse/

Supplementary figure 1-31-5. Score sheets

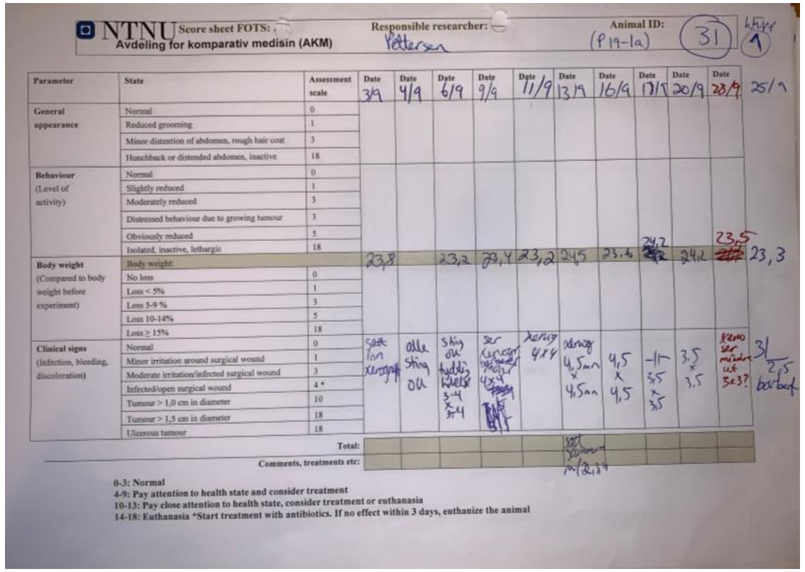

NTNU Score sheet FOTS: Avdeling for komparativ medisin (AKM) — Responsible researcher: Pettersen — Animal ID: 31 (P19-1a) — Wipp 1

Date	3/9	4/9	6/9	9/9	11/9	13/9	16/9	17/9	20/9	23/9	25/9
Body weight	23,8		23,2	23,4	23,2	24,5	23,6	24,2	24,2	23,5	23,3

0-3: Normal
4-9: Pay attention to health state and consider treatment
10-13: Pay close attention to health state, consider treatment or euthanasia
14-18: Euthanasia *Start treatment with antibiotics. If no effect within 3 days, euthanize the animal

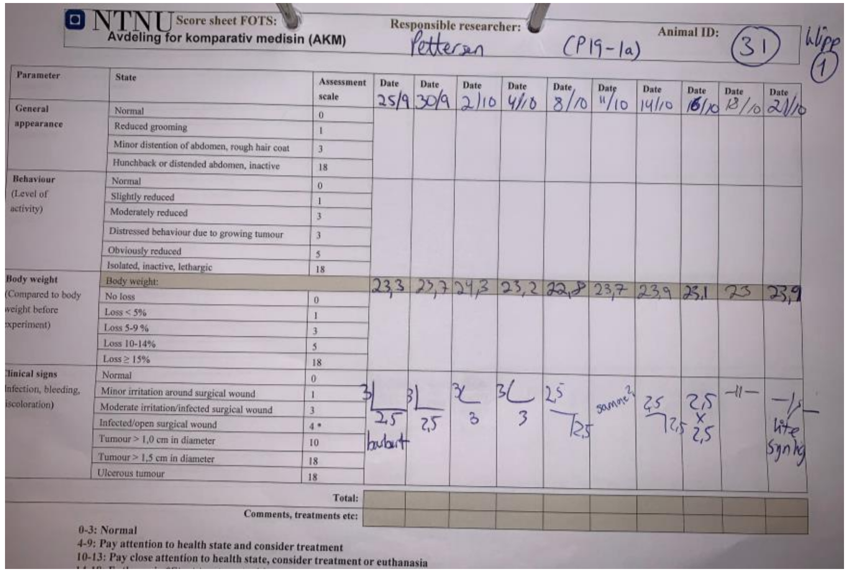

NTNU Score sheet FOTS: Avdeling for komparativ medisin (AKM) — Responsible researcher: Pettersen — Animal ID: 31 (P19-1a) — Wipp 1

Date	25/9	30/9	2/10	4/10	8/10	11/10	14/10	16/10	18/10	21/10
Body weight	23,3	23,7	24,3	23,2	22,8	23,7	23,9	25,1	23	23,9

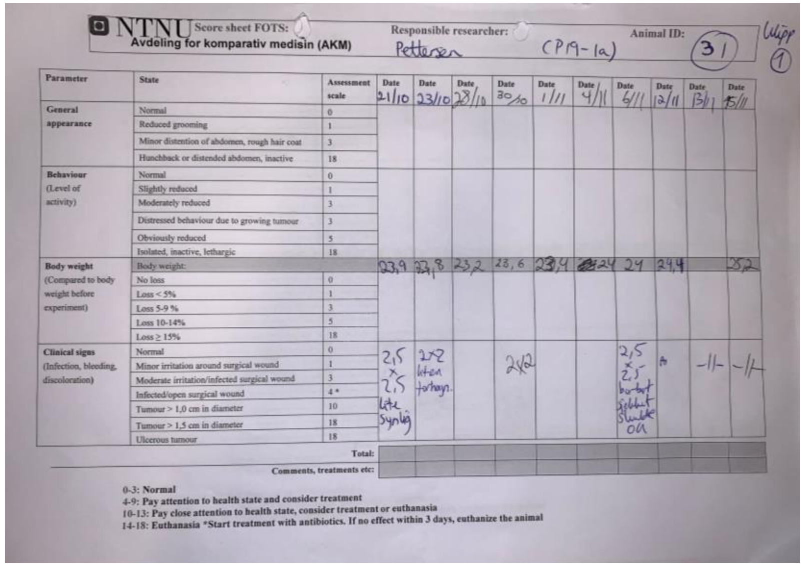

NTNU Score sheet FOTS: Avdeling for komparativ medisin (AKM) — Responsible researcher: Pettersen — Animal ID: 31 (P19-1a) — Wipp 1

Date	21/10	23/10	28/10	30/10	1/11	4/11	6/11	12/11	13/11	15/11
Body weight	23,9	23,8	23,2	23,6	23,4	24	24	24,4		25,2

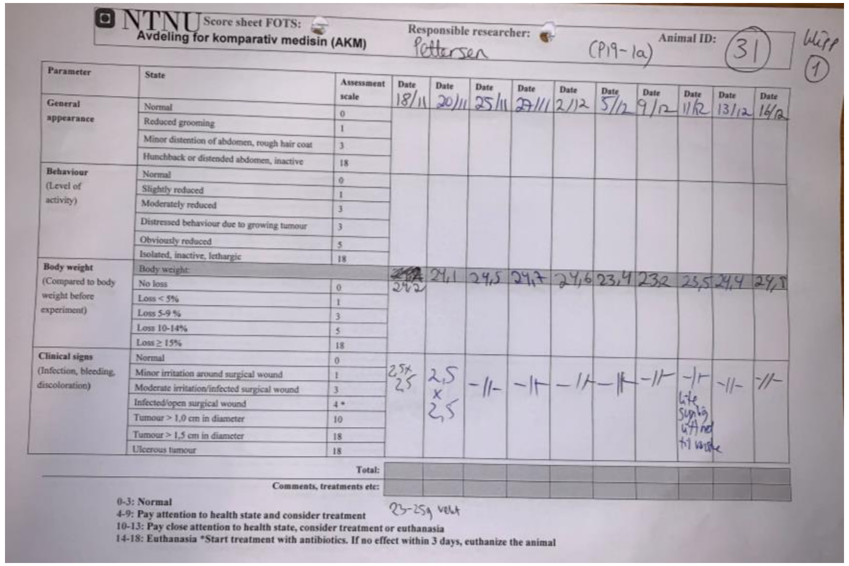

NTNU Score sheet FOTS: Avdeling for komparativ medisin (AKM) — Responsible researcher: Pettersen — Animal ID: 31 (P19-1a) — Wipp 1

Date	18/11	20/11	25/11	27/11	2/12	5/12	9/12	11/12	13/12	16/12
Body weight	24,2	24,1	24,5	24,7	24,6	23,4	23,2	23,5	24,4	24,5

23-25g vekt

Supplementary figure 1-31-5. Score sheets

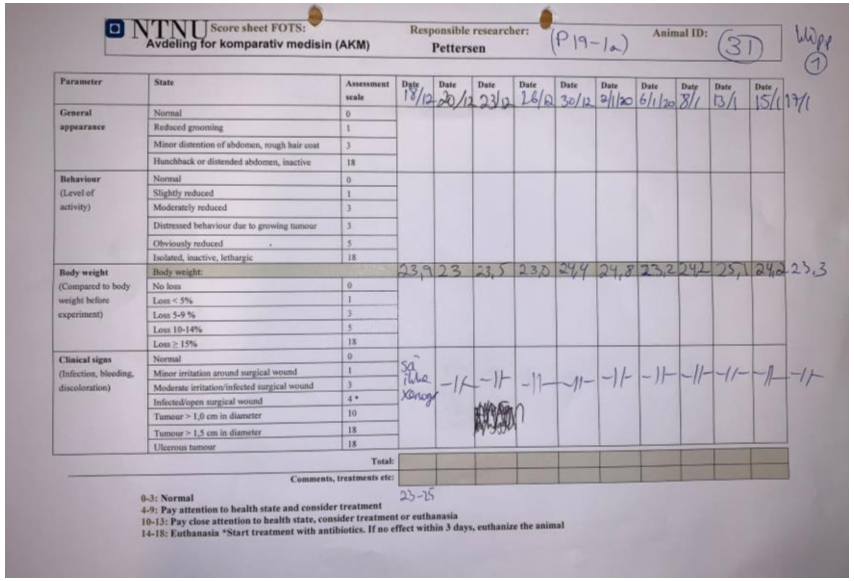

NTNU Score sheet FOTS: Avdeling for komparativ medisin (AKM) — Responsible researcher: Pettersen (P19-1a) — Animal ID: (31) — klipp (1)

Parameter	State	Assessment scale	Date 18/12	Date 20/12	Date 23/12	Date 26/12	Date 30/12	Date 2/1/20	Date 6/1/20	Date 8/1	Date 13/1	Date 15/1	17/1
General appearance	Normal	0											
	Reduced grooming	1											
	Minor distention of abdomen, rough hair coat	3											
	Hunchback or distended abdomen, inactive	18											
Behaviour (Level of activity)	Normal	0											
	Slightly reduced	1											
	Moderately reduced	3											
	Distressed behaviour due to growing tumour	3											
	Obviously reduced	5											
	Isolated, inactive, lethargic	18											
Body weight (Compared to body weight before experiment)	Body weight:		23,9	23	23,5	23,0	24,4	24,8	23,2	24,2	25,1	24,2	23,3
	No loss	0											
	Loss < 5%	1											
	Loss 5-9 %	3											
	Loss 10-14%	5											
	Loss ≥ 15%	18											
Clinical signs (Infection, bleeding, discoloration)	Normal	0	så ikke xenograft	-II-	-II-	-II-	-II-	-II-	-II-	-II-	-II-	-II-	-II-
	Minor irritation around surgical wound	1											
	Moderate irritation/infected surgical wound	3											
	Infected/open surgical wound	4*											
	Tumour > 1,0 cm in diameter	10											
	Tumour > 1,5 cm in diameter	18											
	Ulcerous tumour	18											
	Total:												
	Comments, treatments etc:		23-25										

0-3: Normal
4-9: Pay attention to health state and consider treatment
10-13: Pay close attention to health state, consider treatment or euthanasia
14-18: Euthanasia *Start treatment with antibiotics. If no effect within 3 days, euthanize the animal

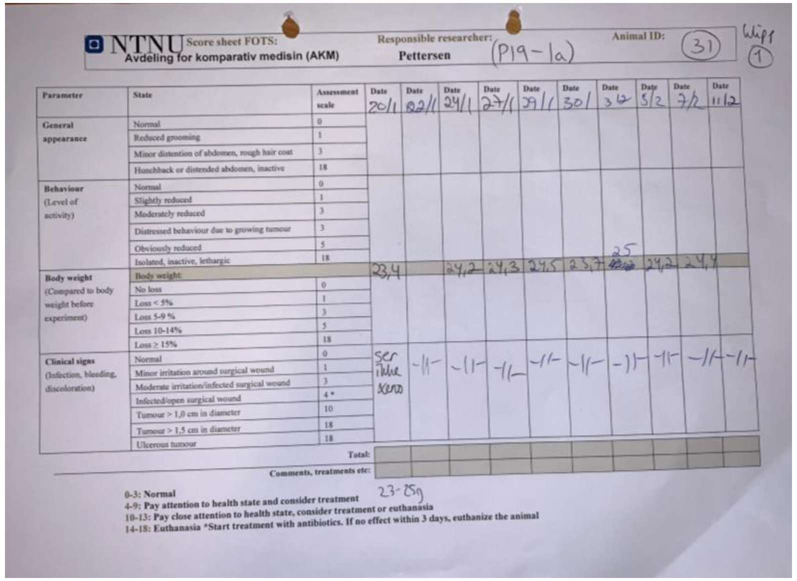

NTNU Score sheet FOTS: Avdeling for komparativ medisin (AKM) — Responsible researcher: Pettersen (P19-1a) — Animal ID: (31) — klipp (1)

Parameter	State	Assessment scale	Date 20/1	Date 22/1	Date 24/1	Date 27/1	Date 29/1	Date 30/1	Date 3/2	Date 5/2	Date 7/2	Date 11/2
General appearance	Normal	0										
	Reduced grooming	1										
	Minor distention of abdomen, rough hair coat	3										
	Hunchback or distended abdomen, inactive	18										
Behaviour (Level of activity)	Normal	0										
	Slightly reduced	1										
	Moderately reduced	3										
	Distressed behaviour due to growing tumour	3										
	Obviously reduced	5										
	Isolated, inactive, lethargic	18										
Body weight (Compared to body weight before experiment)	Body weight:		23,4		24,2	24,3	24,5	23,7	25	24,2	24,4	
	No loss	0										
	Loss < 5%	1										
	Loss 5-9 %	3										
	Loss 10-14%	5										
	Loss ≥ 15%	18										
Clinical signs (Infection, bleeding, discoloration)	Normal	0	ser ikke xeno	-II-	-II-	-II-	-II-	-II-	-II-	-II-	-II-	-II-
	Minor irritation around surgical wound	1										
	Moderate irritation/infected surgical wound	3										
	Infected/open surgical wound	4*										
	Tumour > 1,0 cm in diameter	10										
	Tumour > 1,5 cm in diameter	18										
	Ulcerous tumour	18										
	Total:											
	Comments, treatments etc:		23-25g									

0-3: Normal
4-9: Pay attention to health state and consider treatment
10-13: Pay close attention to health state, consider treatment or euthanasia
14-18: Euthanasia *Start treatment with antibiotics. If no effect within 3 days, euthanize the animal

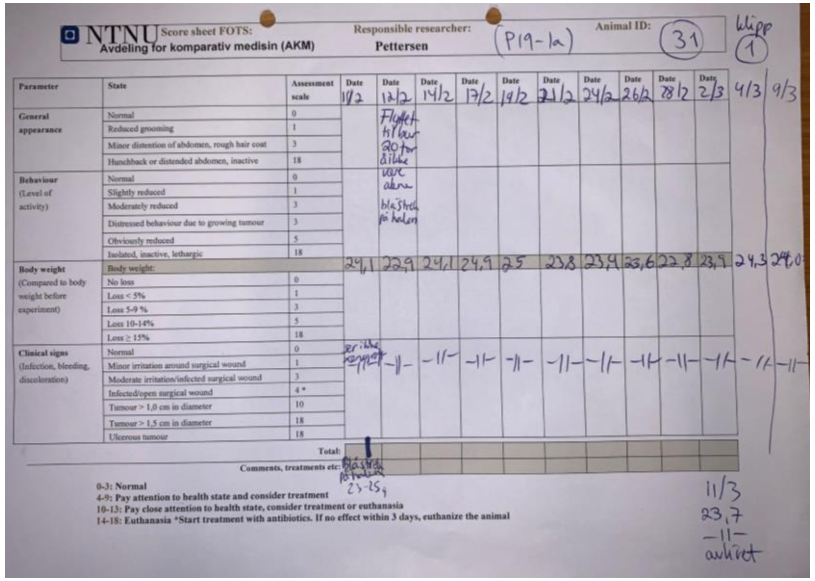

NTNU Score sheet FOTS: Avdeling for komparativ medisin (AKM) — Responsible researcher: Pettersen (P19-1a) — Animal ID: (31) — klipp (1)

Parameter	State	Assessment scale	Date 11/2	Date 12/2	Date 14/2	Date 17/2	Date 19/2	Date 21/2	Date 24/2	Date 26/2	Date 28/2	Date 2/3	4/3	9/3
General appearance	Normal	0		Flyttet til bur 20 for å ikke være alene										
	Reduced grooming	1												
	Minor distention of abdomen, rough hair coat	3												
	Hunchback or distended abdomen, inactive	18												
Behaviour (Level of activity)	Normal	0		blåstrek på halen										
	Slightly reduced	1												
	Moderately reduced	3												
	Distressed behaviour due to growing tumour	3												
	Obviously reduced	5												
	Isolated, inactive, lethargic	18												
Body weight (Compared to body weight before experiment)	Body weight:		24,1	22,9	24,1	24,9	25	23,8	23,9	23,6	22,8	23,9	24,3	24,0
	No loss	0												
	Loss < 5%	1												
	Loss 5-9 %	3												
	Loss 10-14%	5												
	Loss ≥ 15%	18												
Clinical signs (Infection, bleeding, discoloration)	Normal	0	ser ikke xenograft -II-	-II-	-II-	-II-	-II-	-II-	-II-	-II-	-II-	-II-	-II-	-II-
	Minor irritation around surgical wound	1												
	Moderate irritation/infected surgical wound	3												
	Infected/open surgical wound	4*												
	Tumour > 1,0 cm in diameter	10												
	Tumour > 1,5 cm in diameter	18												
	Ulcerous tumour	18												
	Total:													
	Comments, treatments etc:		blåstrek på halen 23-25g											

0-3: Normal
4-9: Pay attention to health state and consider treatment
10-13: Pay close attention to health state, consider treatment or euthanasia
14-18: Euthanasia *Start treatment with antibiotics. If no effect within 3 days, euthanize the animal

11/3
23,7
-II-
avlivet

Supplementary figure 1-31-6.

Mouse and xenograft growth curves

Weight curve

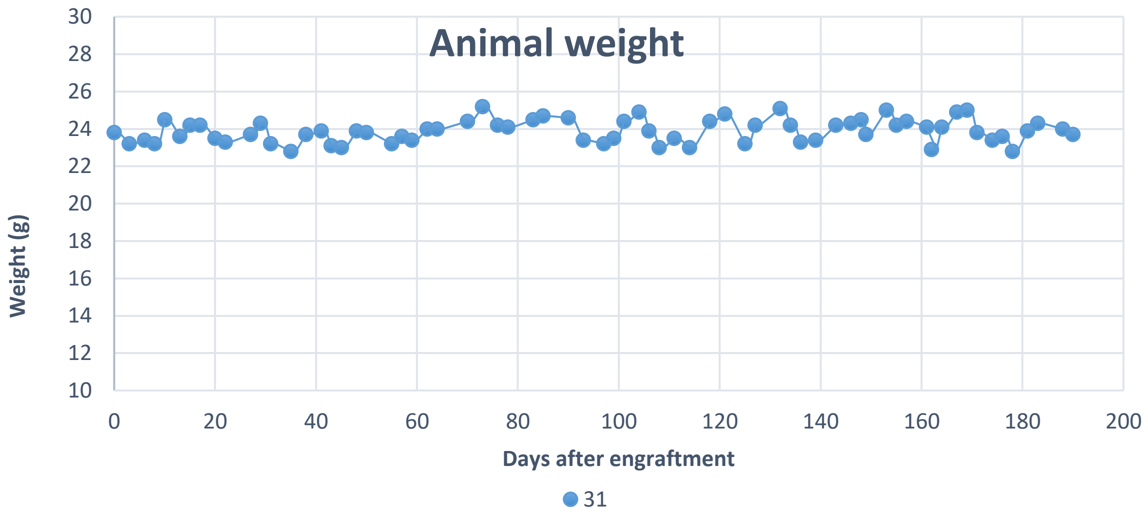

Xenograft growth curve

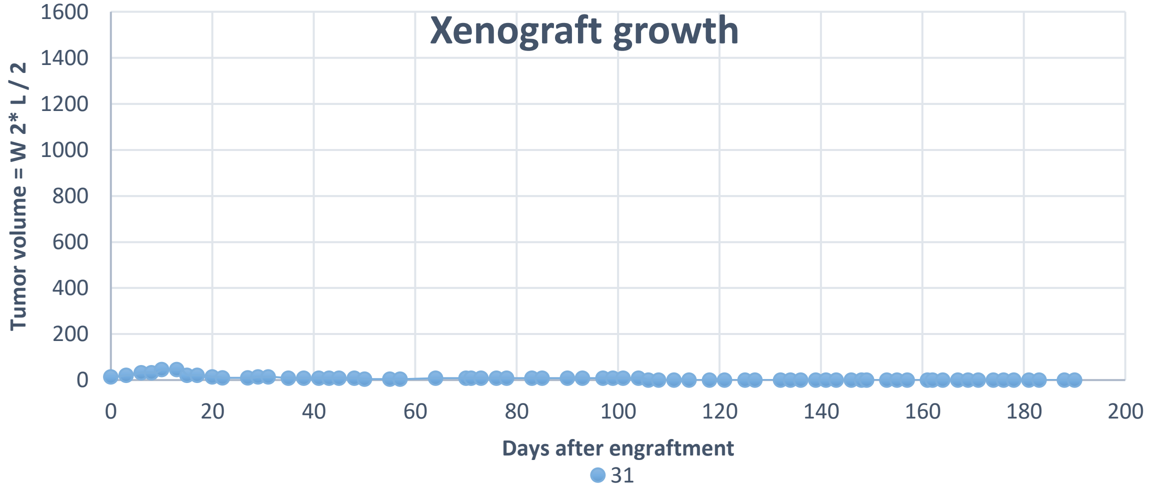

Supplementary figure 1-31-7. Histology pictures

M31X – Remainings of xenograft?

- Looks like an inflammatory reaction that might have killed the xenograft
- Can see multinuclear and lymphoid cells

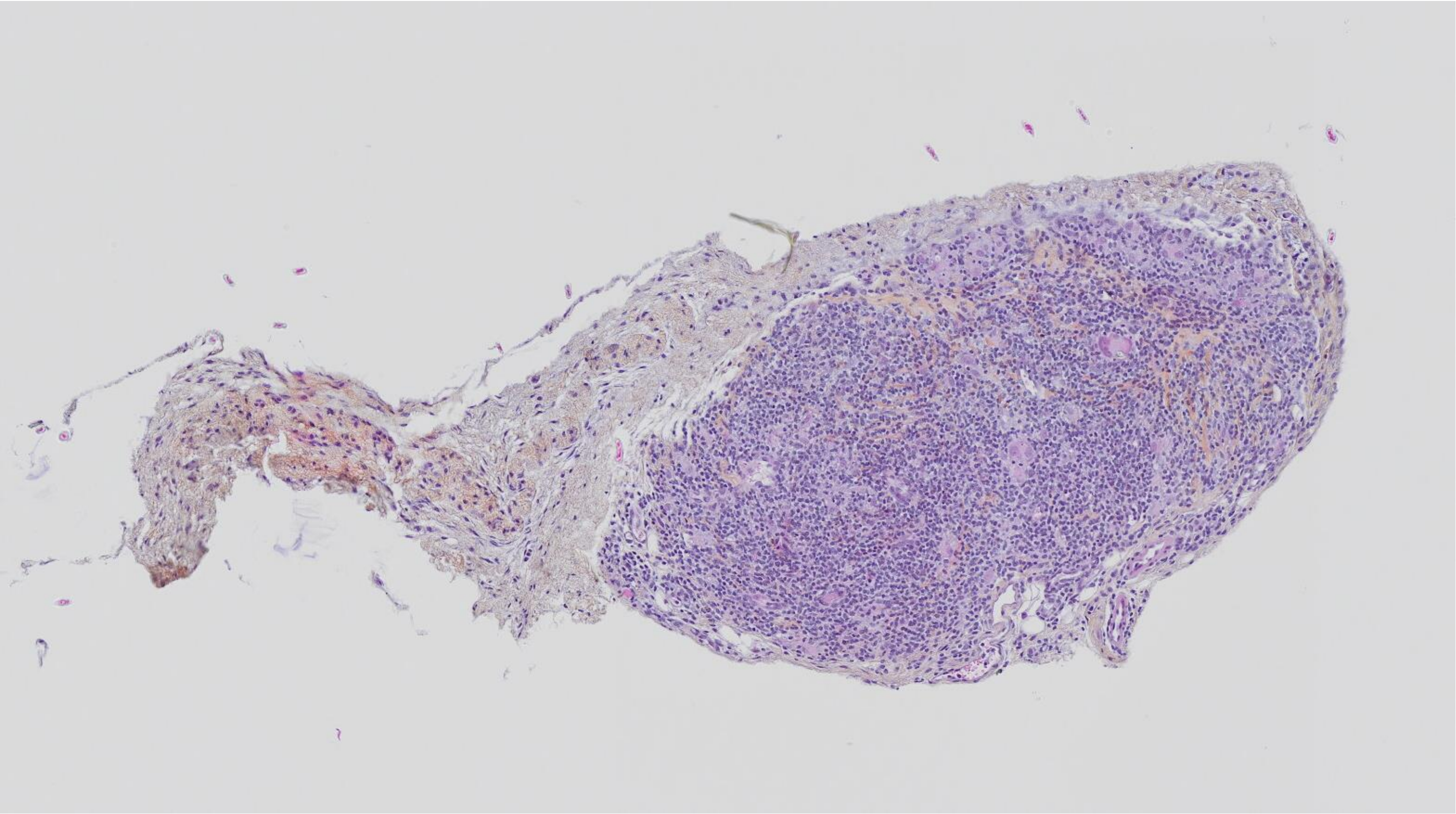

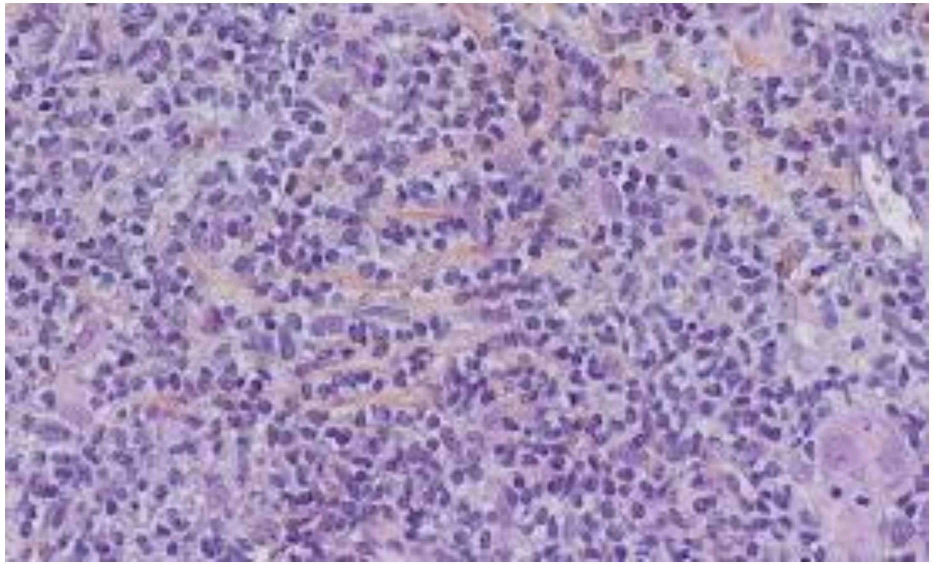

Supplementary figure 1-31-7. Histology pictures

M31MP – Spleen and pancreas
- Looks normal

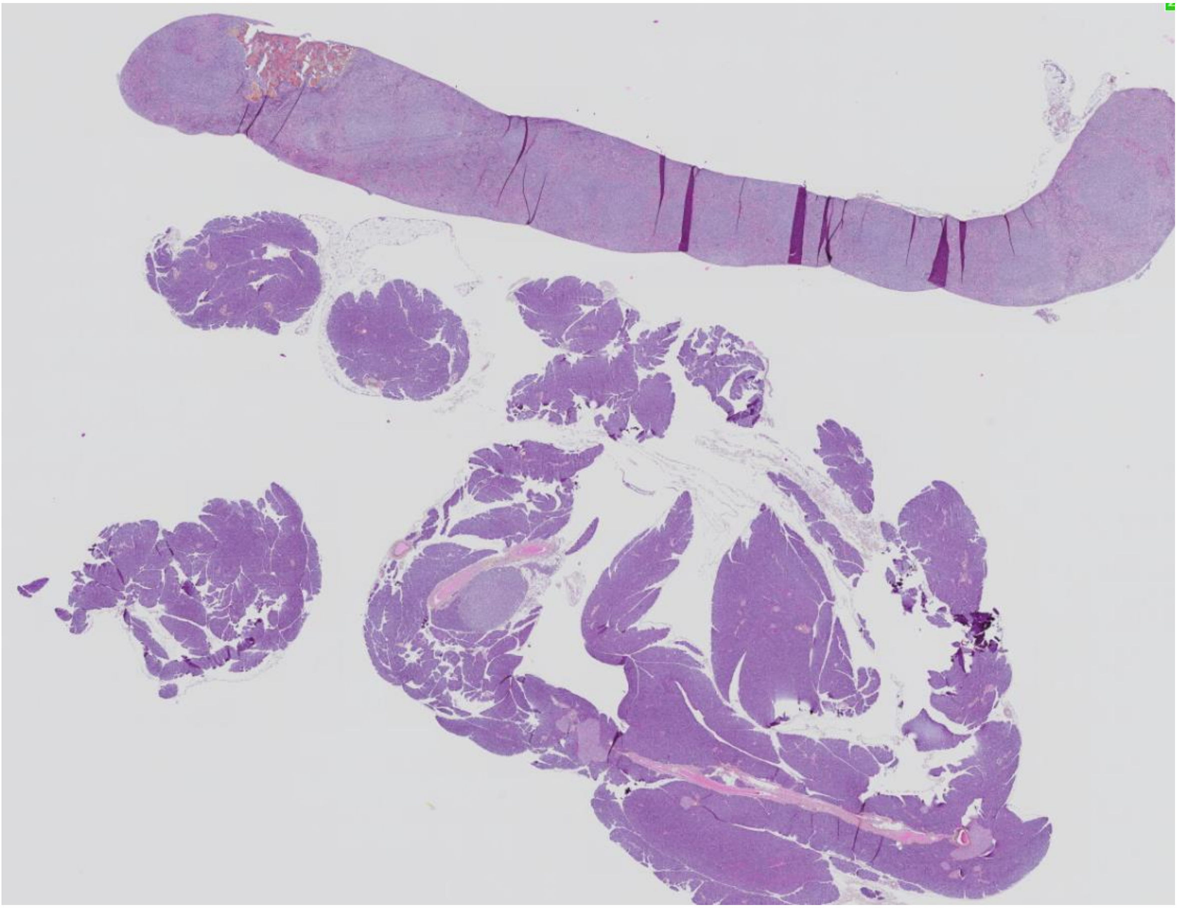

M31LH – Lungs and heart
- Looks normal

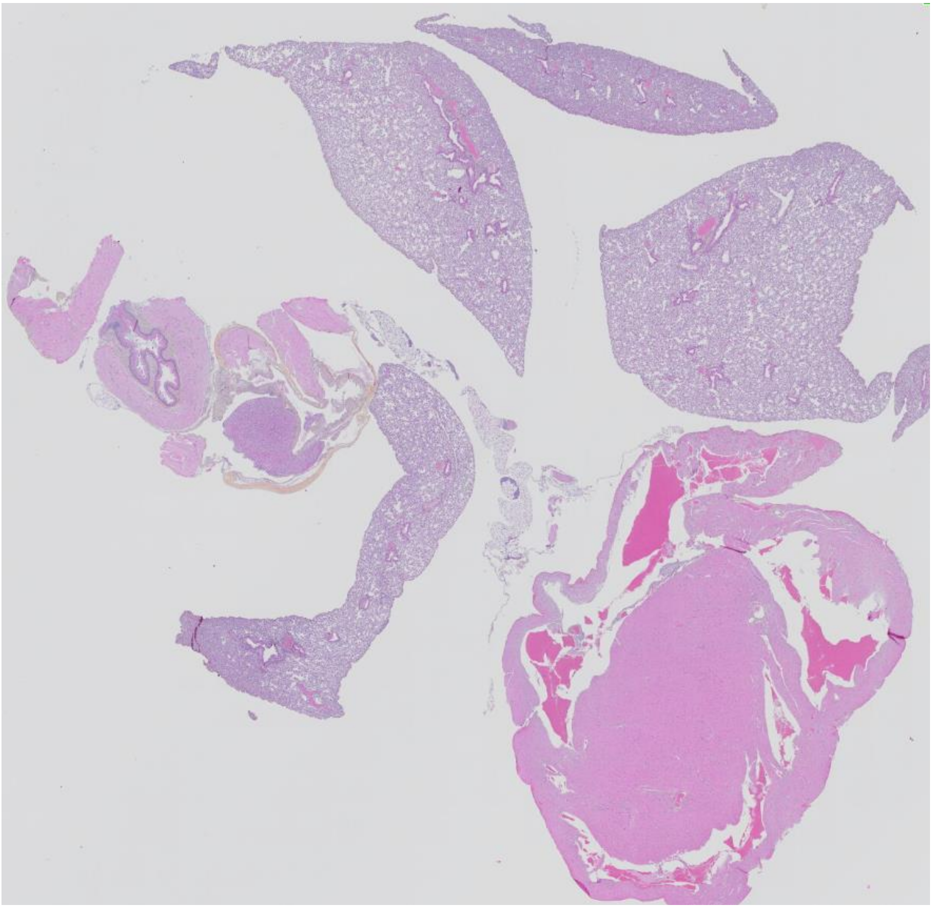

Mouse 32

- Surgery date: 03.09.19
  - Age (weeks) 33
  - Weight (g) 22.2
  - Tumor fragment P19-1b
  - Fragment size 3 x 3 mm
  - Ear marks 2

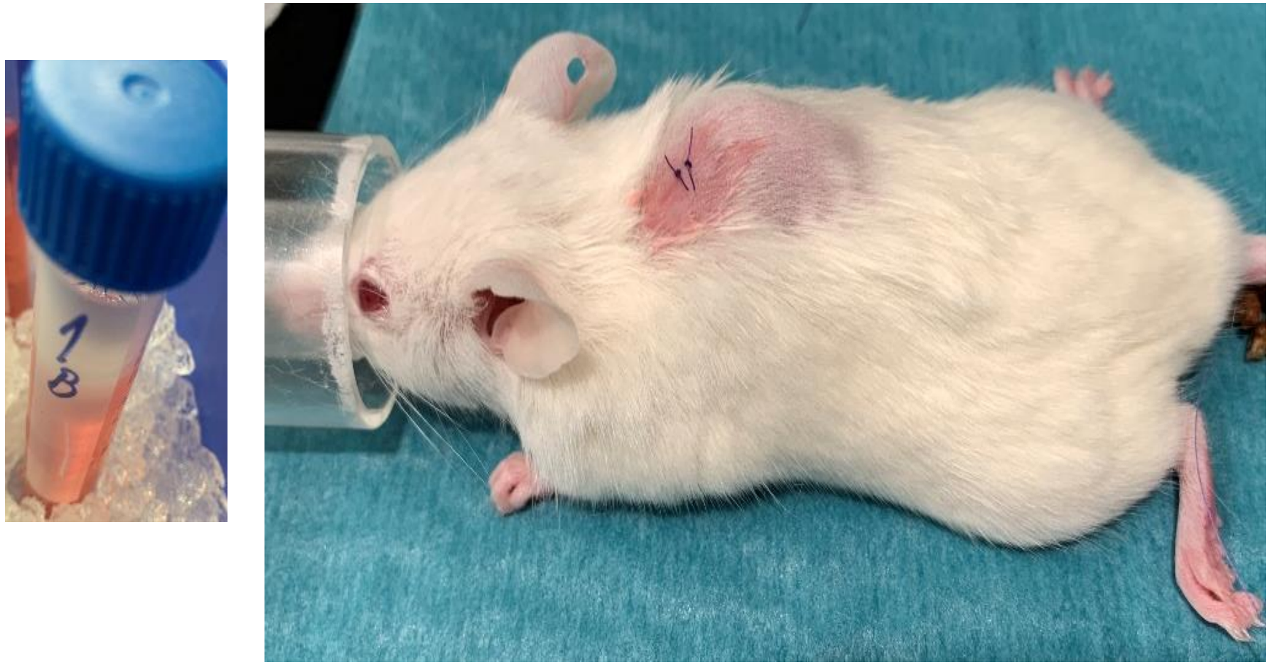

Supplementary figure 1-32-1. Pictures from the engraftment day

Supplementary figure 1-32-2. Pictures from follow up and xenograft size measurement

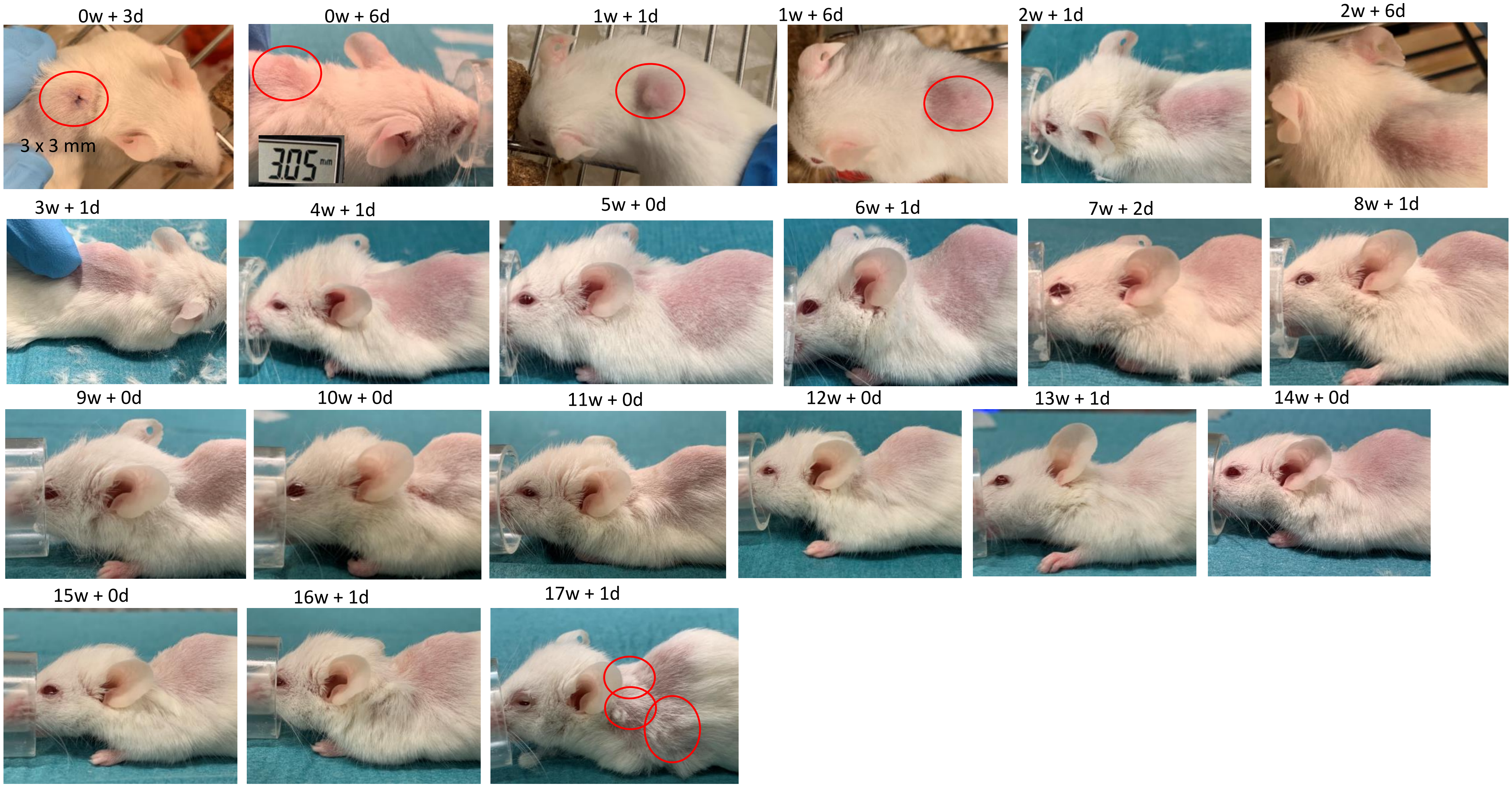

Supplementary figure 1-32-3. Pictures from euthanization (02.01.20)

- No treatment given
- Euthanized due to reduced health and development of secondary tumors before growth of xenograft was established

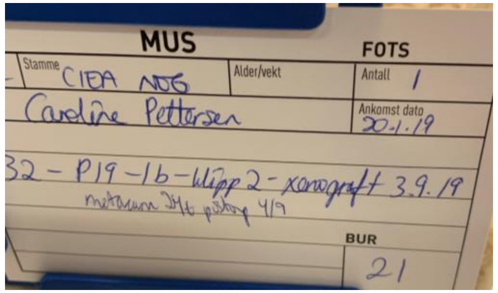

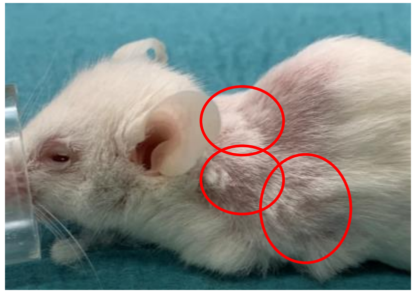

Reduced health, Thick wounded skin at the neck, Liquid in the eye/rinning eye, 3 secondary tumors, The mouse seemed anemic, could not be drawn blood from,

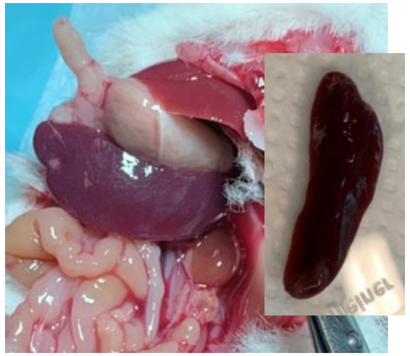

Large spleen, > 2 cm

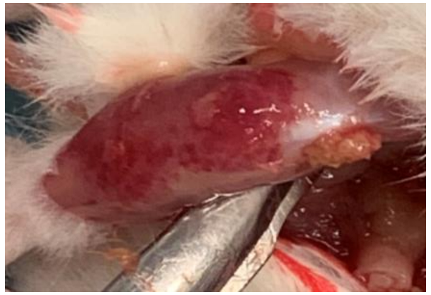

Red svollen legs

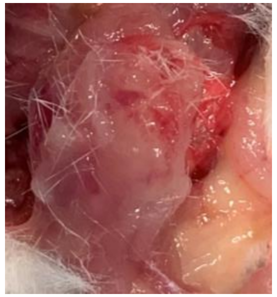

Red marks and svollen left shoulder. Tumor?

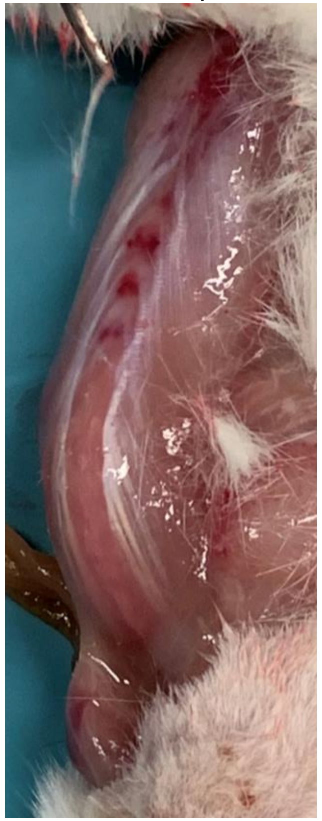

Red marks on the spine

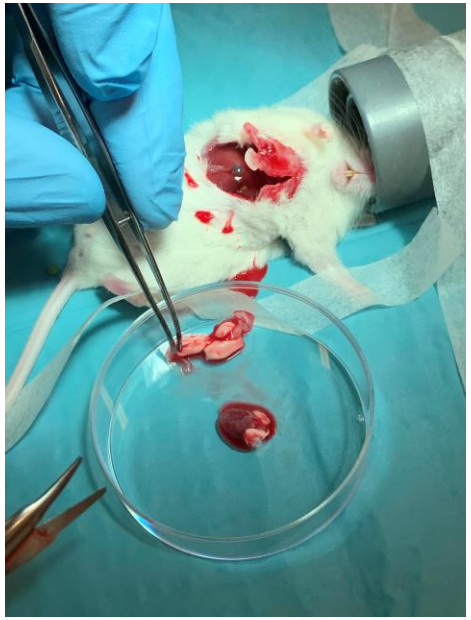

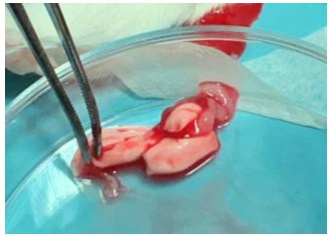

White lungs

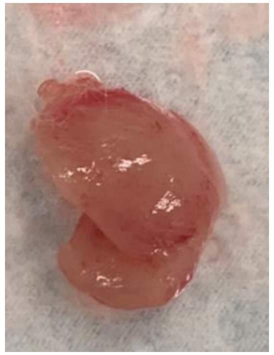

Tumor subcutanous over the ribs left side

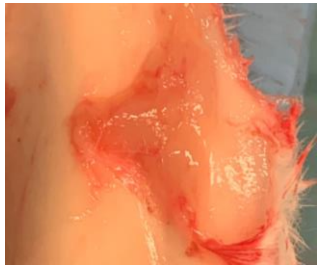

Xenograft? Not growing

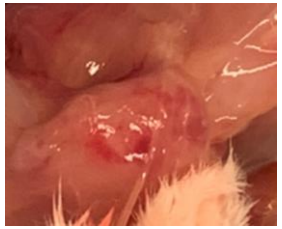

Red marks and svollen right shoulder. Tumor?

Euthanization mouse #32 (P19-1b) 02.01.2020

Before euthanization:

- Reduced health
- Breathing heavily
- Thick irritated skin at the neck for at least 2 weeks
- Rinning eye
- Shoulders seemed enlarged

After euthanization:

- Could not be taken blood sample (anemic?)
- Enlarged spleen >2 cm
- White lungs
- All legs/shoulders red/svollen
- Red marks on the spine
- Xenograft did not seem to have grown
- Subcutanous tumor flat over the ribs

Histology samples:

**M32X**	Xenograft
**M32TS**	Tumor Right Shoulder
**M32M**	Milt
**M32LH**	Lung and Heart
**M32TR**	Tumor over ribs right side
**M32L**	Leg left

Supplementary figure 1-32-4. Score sheets

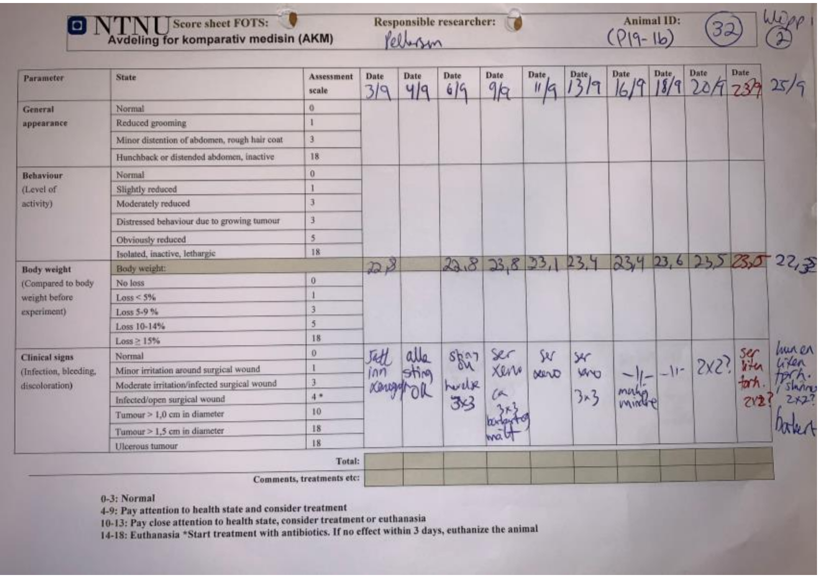

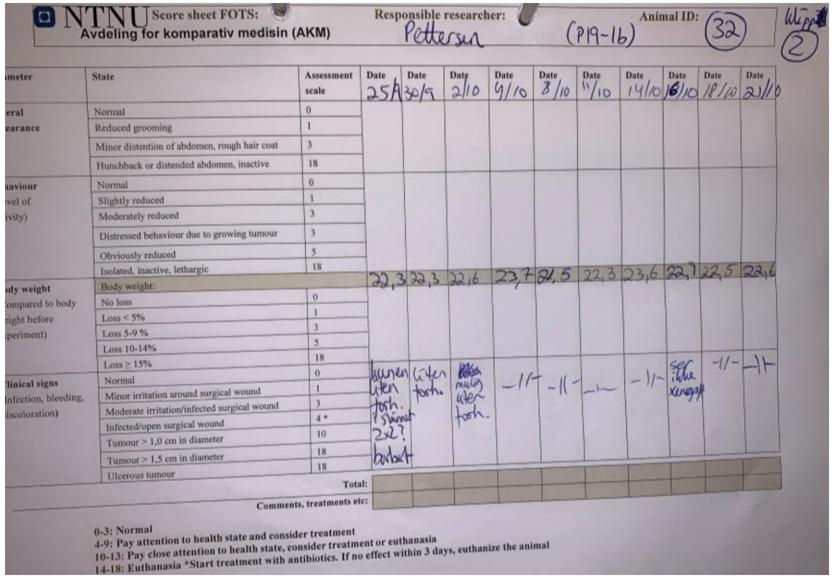

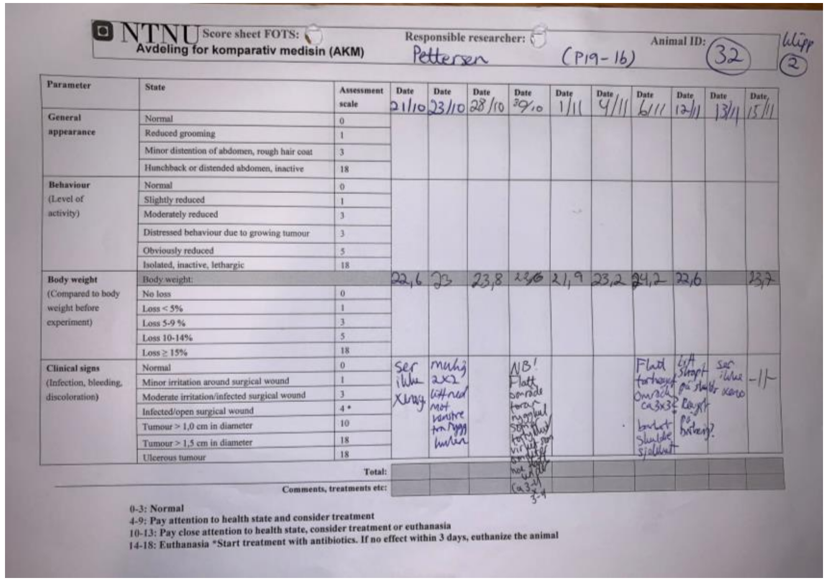

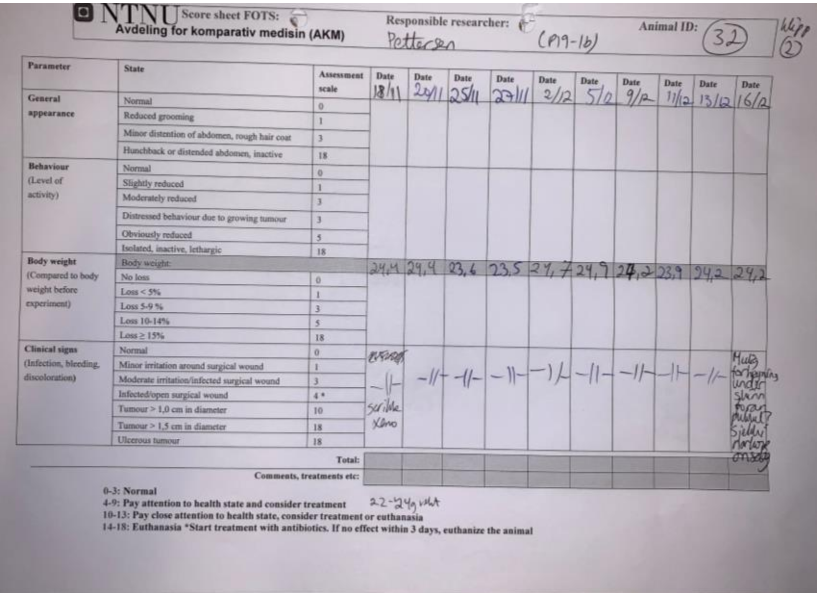

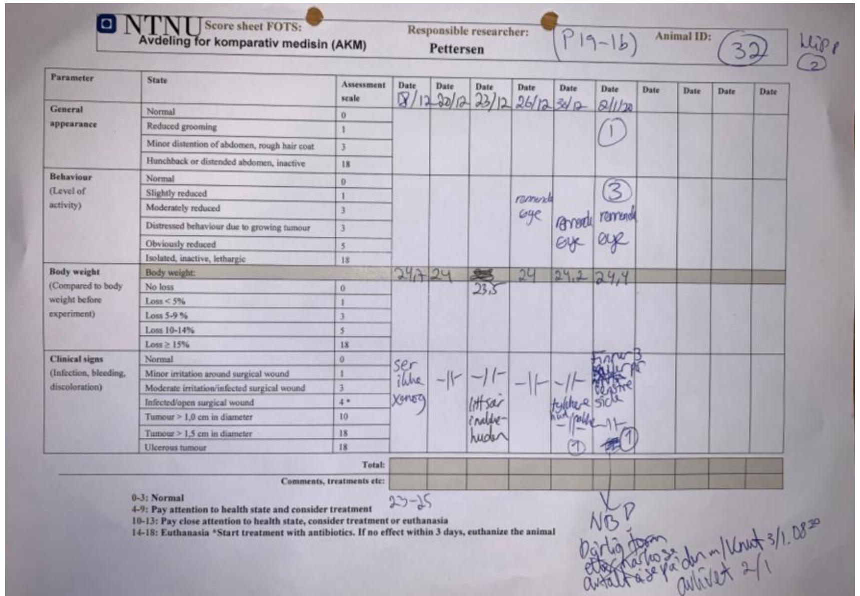

Supplementary figure 1-32-5.

Mouse and xenograft growth curves

Weight curve

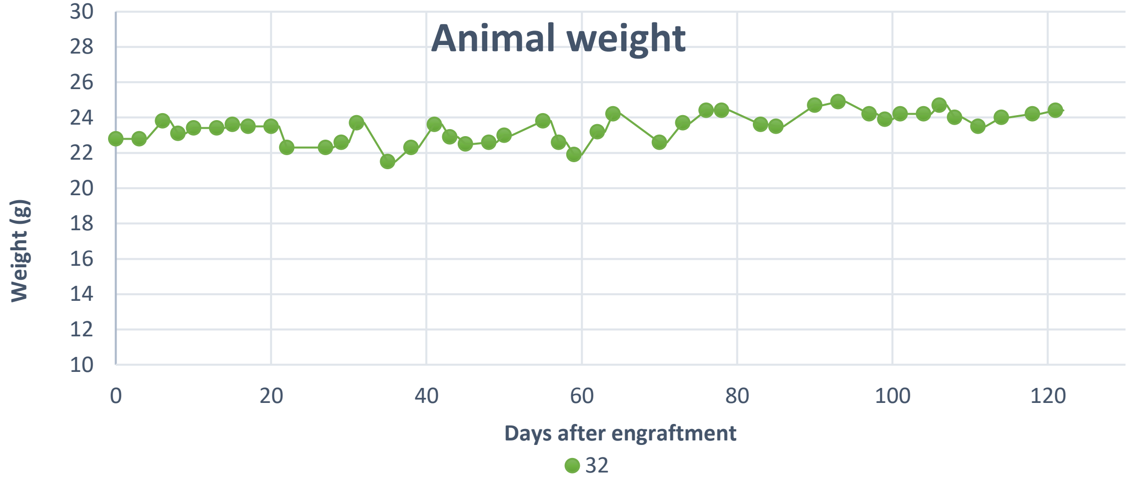

Xenograft growth curve

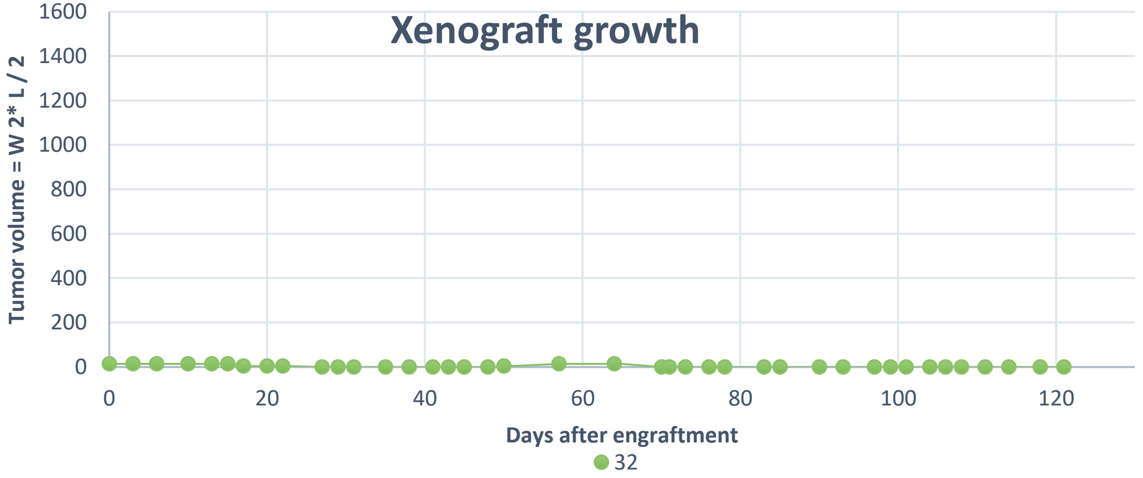

Secondary tumor growth curve

3 secondary tumors found at the day of euthanization. Size was not measured.

Supplementary figure 1-32-6. Histology pictures

M32X

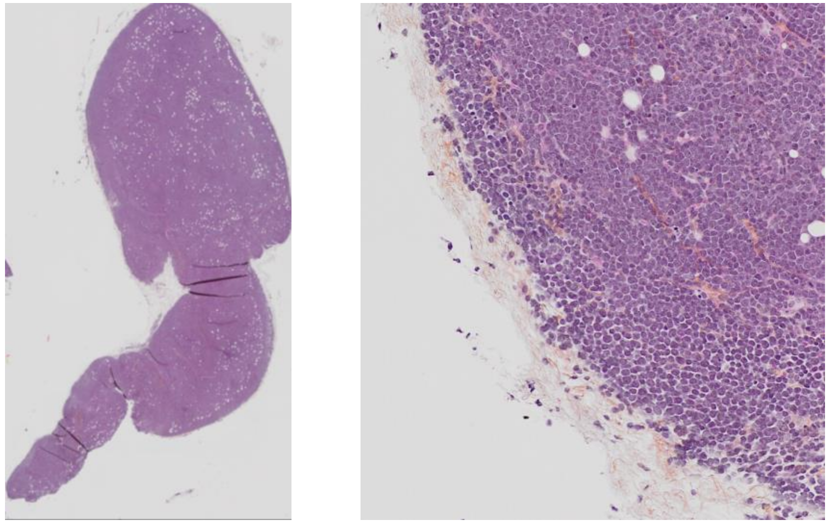

- Xenograft did not grow. During necroscopy what was belived to be remainings of the xenograft was sampled. However HES staining showed that it was probably not the xenograft, but another tissue structure.

M32LH

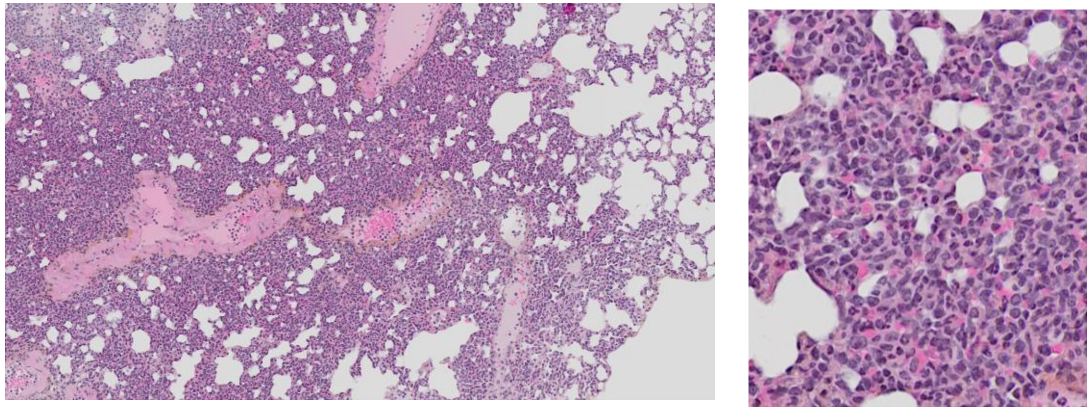

- Dense aereas in the lungs consisting of lymphoid cells

M32L

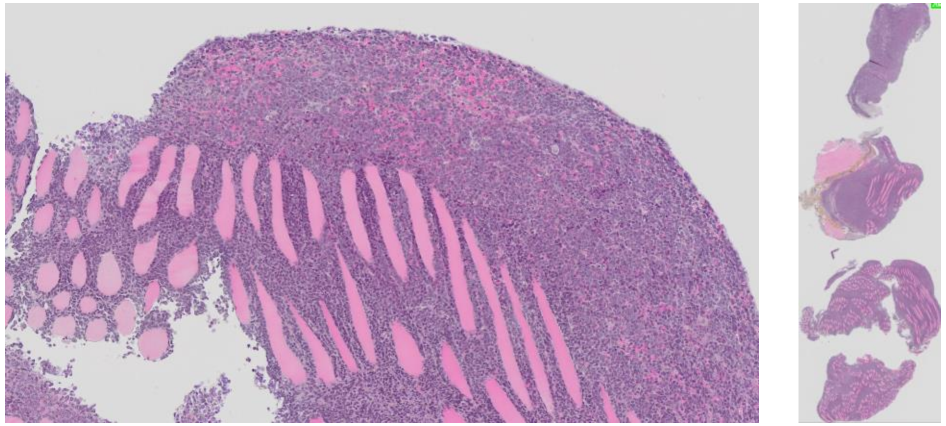

- Red svollen leg
- Lymphoid tumor cells present
- Bone structures

Supplementary figure 1-32-6. Histology pictures

M32M

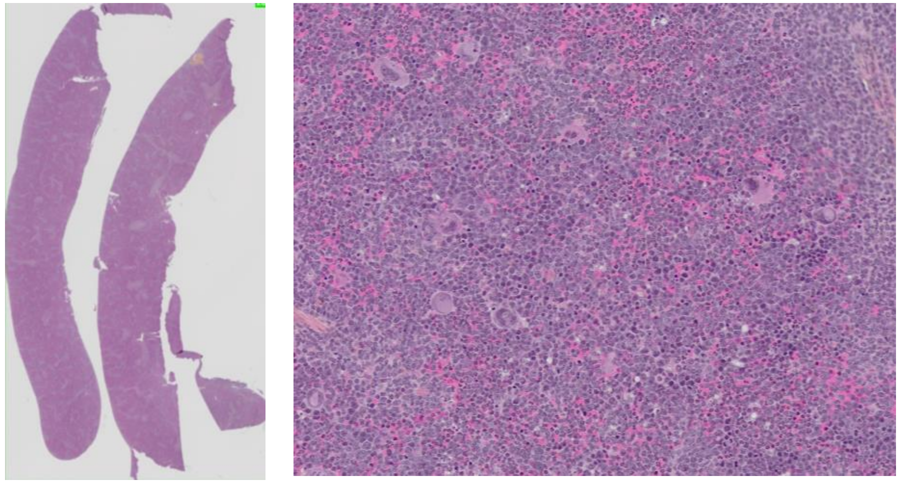

- Large spleen > 2 cm with lymphoid cells

M32TS

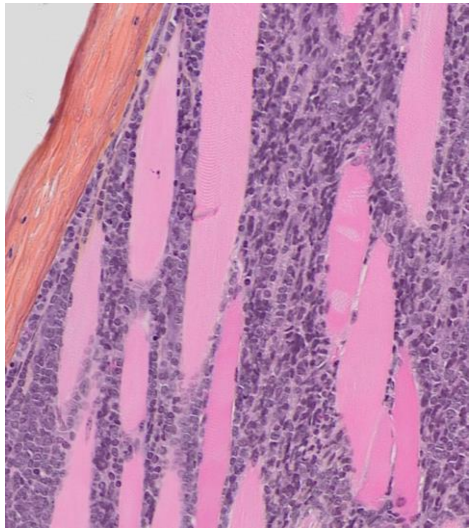

- Ca 1 cm tumor on right shoulder
- Bone structures
- Lymphoid tumor cells

M32TR – secondary tumor

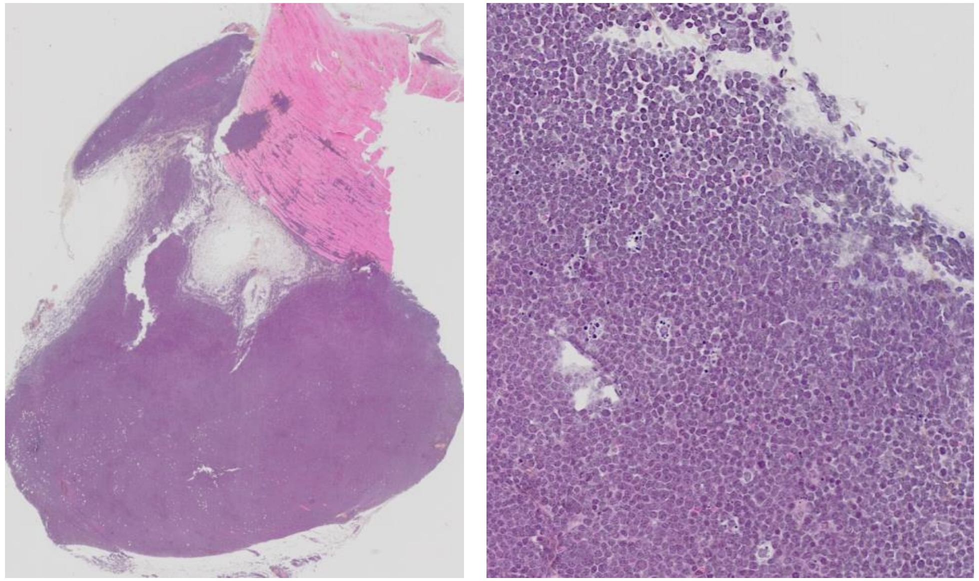

- Lymphoid tumor cells

Supplementary figure 1-32-7. Histology and immunohistochemistry pictures

M32TR – secondary tumor

- Lymphoma
- IHC staining for human spesific Ki67 and CD45 negative
- EBER-ISH staining for EBV negative
- Most likely a mouse tumor

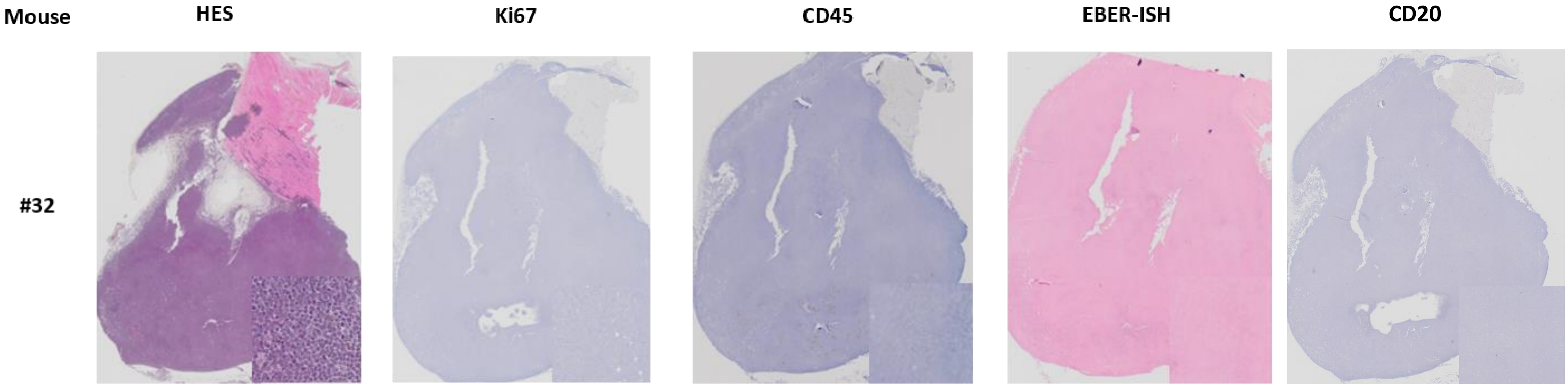

Mouse 33

- Surgery date: 03.09.19
    - Age (weeks) 33
    - Weight (g) 21.9
    - Tumor fragment P19-2a
    - Fragment size 3 x 3 mm
    - Ear marks 3

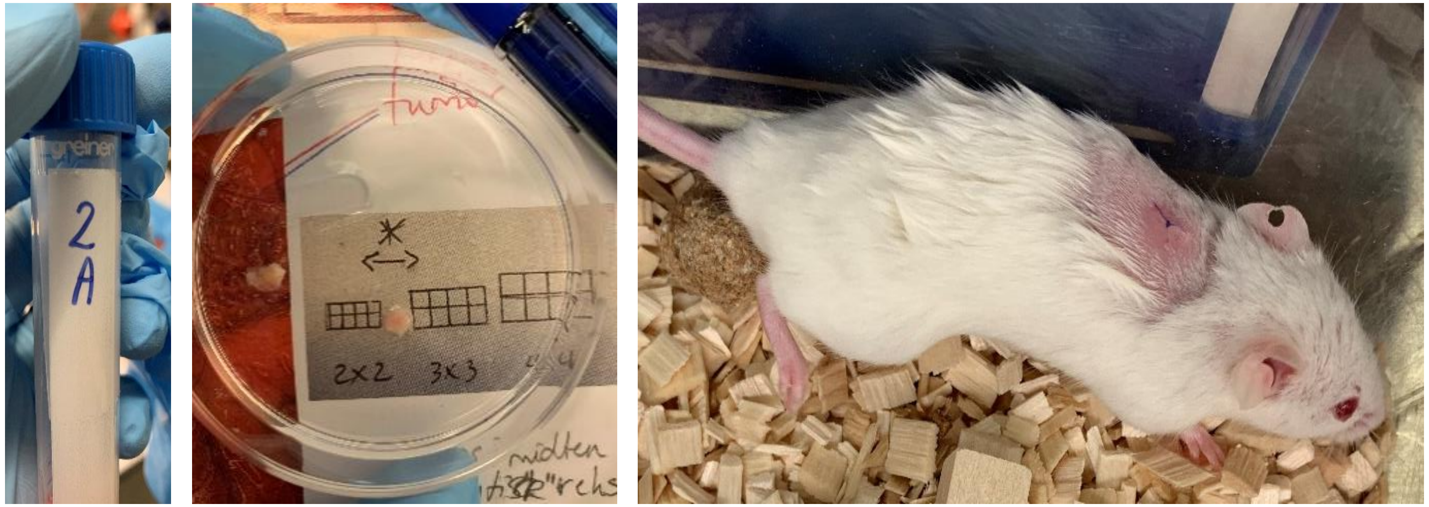

Supplementary figure 1-33-1. Pictures from the engraftment day

Supplementary figure 1-33-2. Pictures from follow up and xenograft size measurement

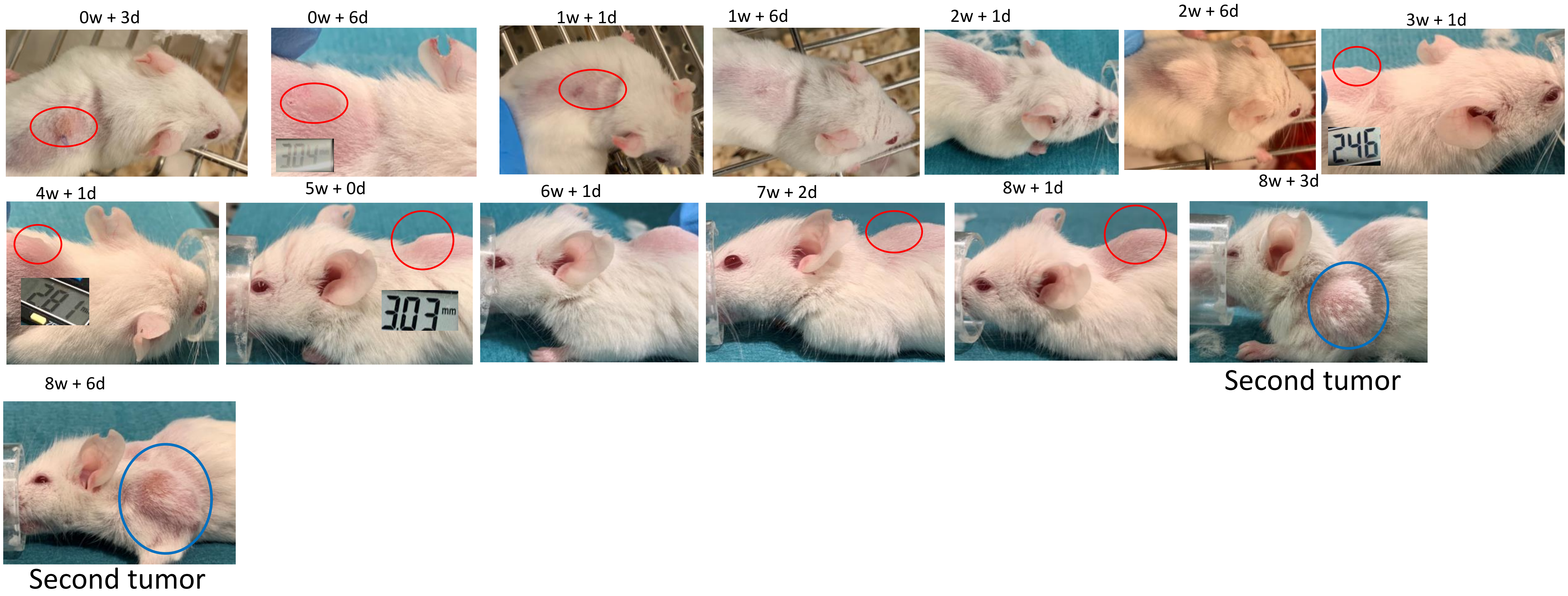

Supplementary figure 1-33-3. Pictures from euthanization (04.11.19)

- No treatment given (xenograft not growing)
- Euthanized due to large secondary tumor before growth of xenograft was established
- Secondary tumor: size: 14 x 9 mm

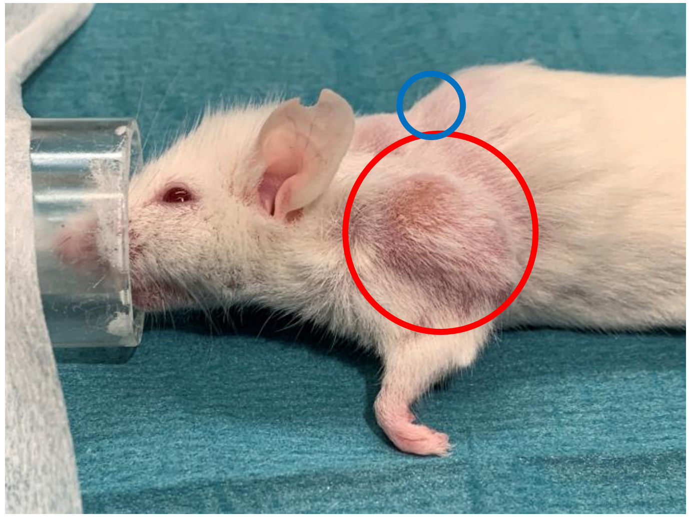

Blue; no visiable xenograft
Red; secondary tumor

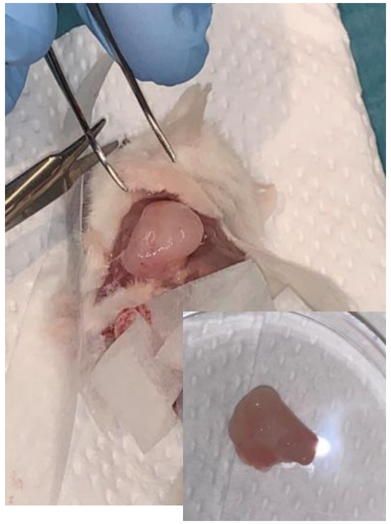

Secondary tumor

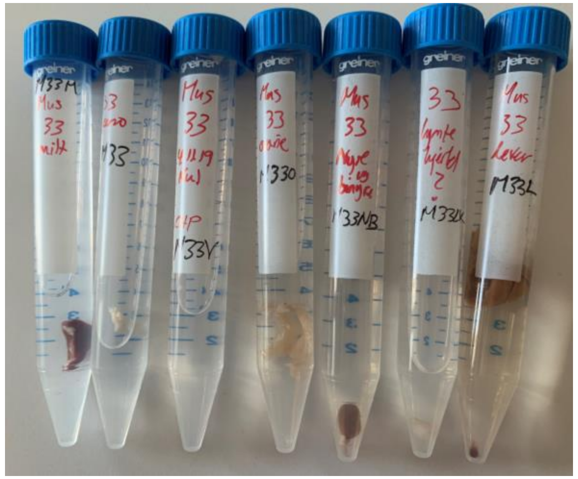

Histology samples

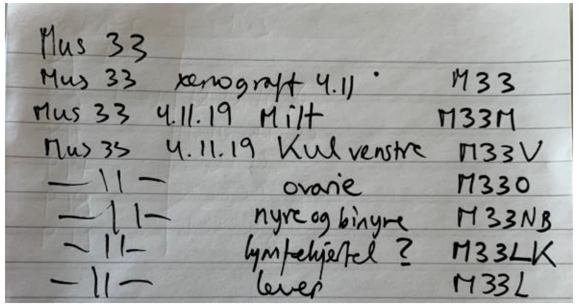

Xenograft
Spleen
Tumor left
Ovary
Kidney
Lymph node?
Liver

Naming of histology samples

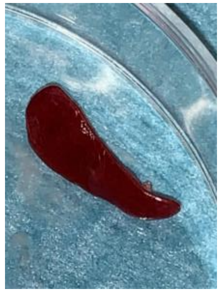

Enlarged spleen

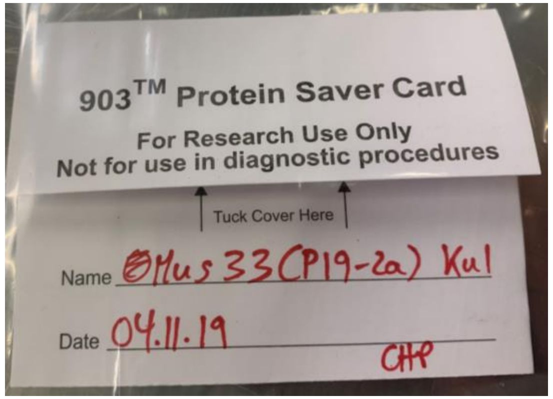

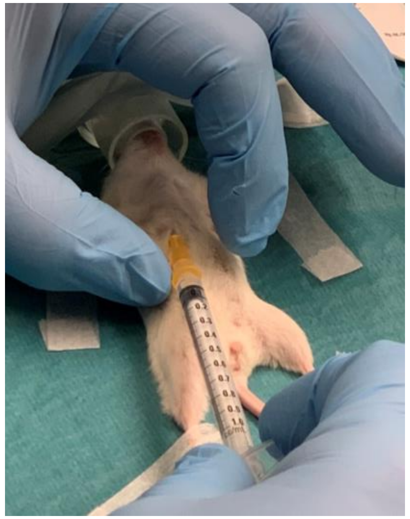

Blood sampling

Supplementary figure 1-33-4. Score sheets

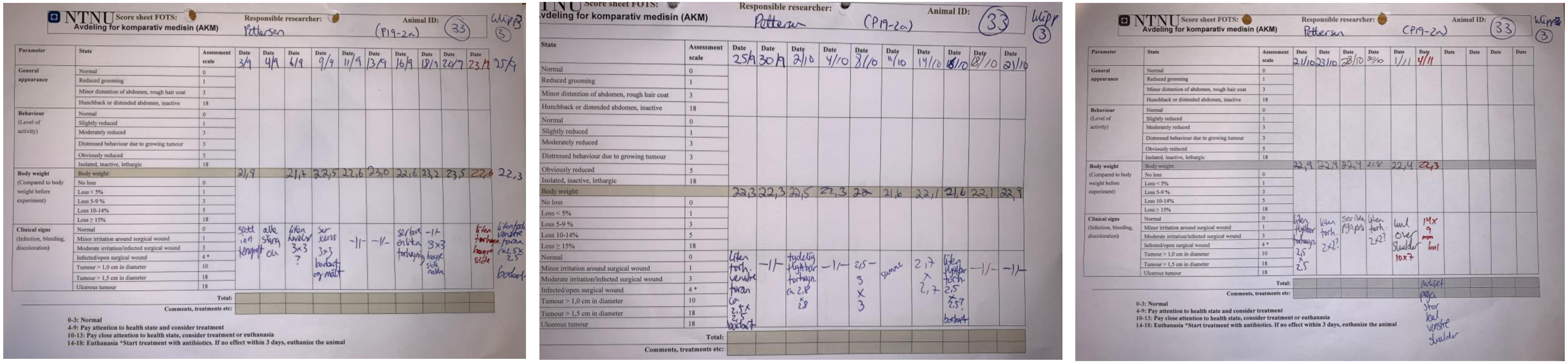

Supplementary figure 1-33-5.
Mouse, xenograft and secondary tumor growth curves

Weight curve

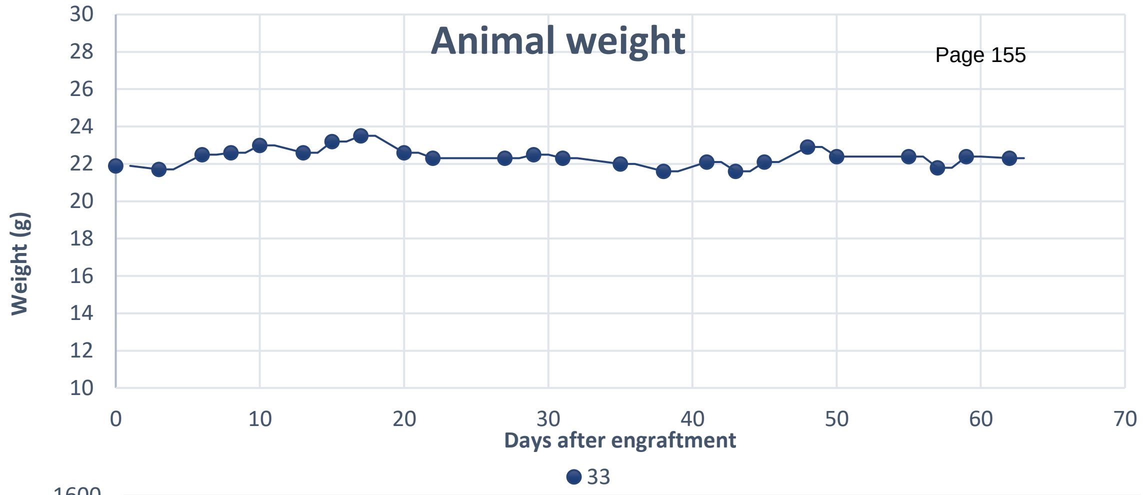

Xenograft growth curve

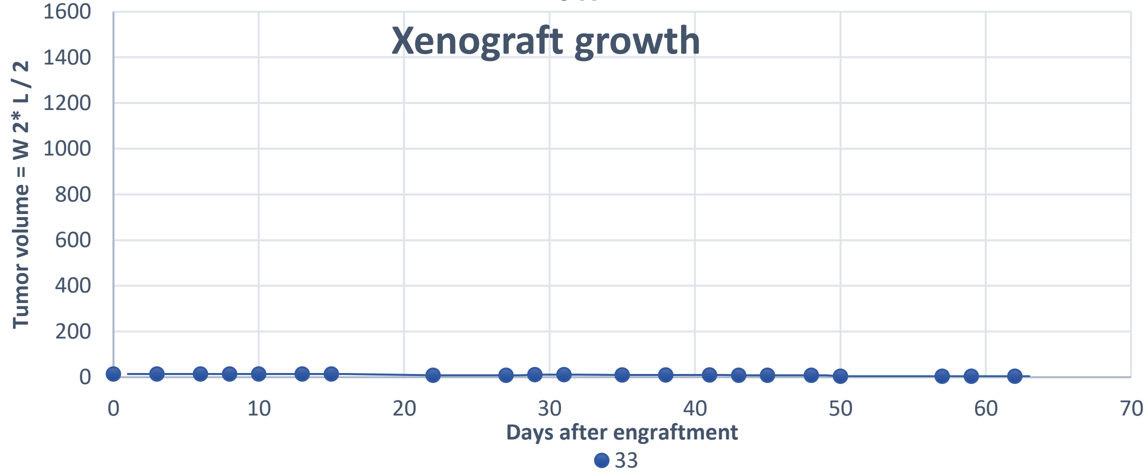

Secondary tumor growth curve

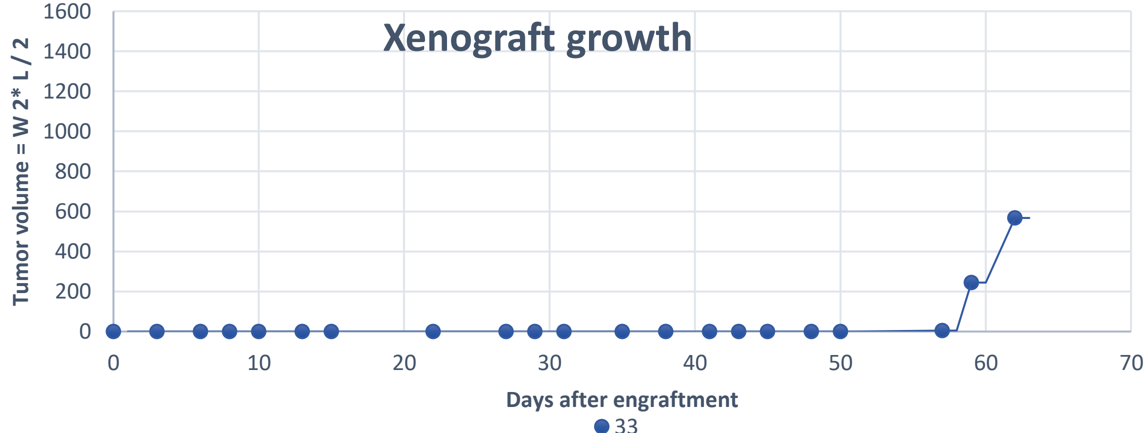

Supplementary figure 1-33-6. Histology pictures

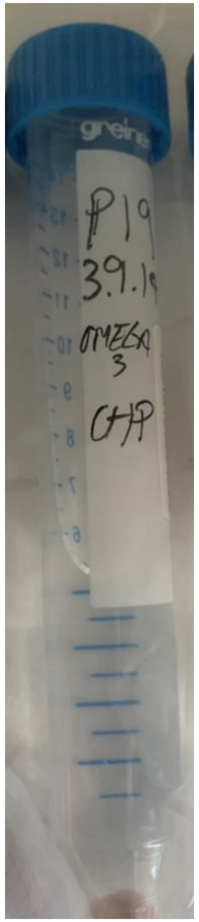

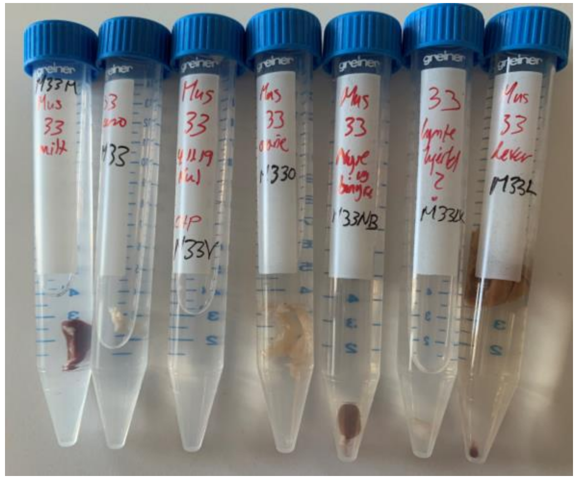

Histology samples

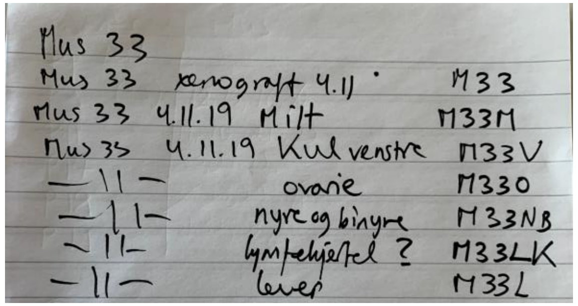

Xenograft
Spleen
Tumor left
Ovary
Kidney
Lymph node?
Liver

Naming of histology samples

M33X

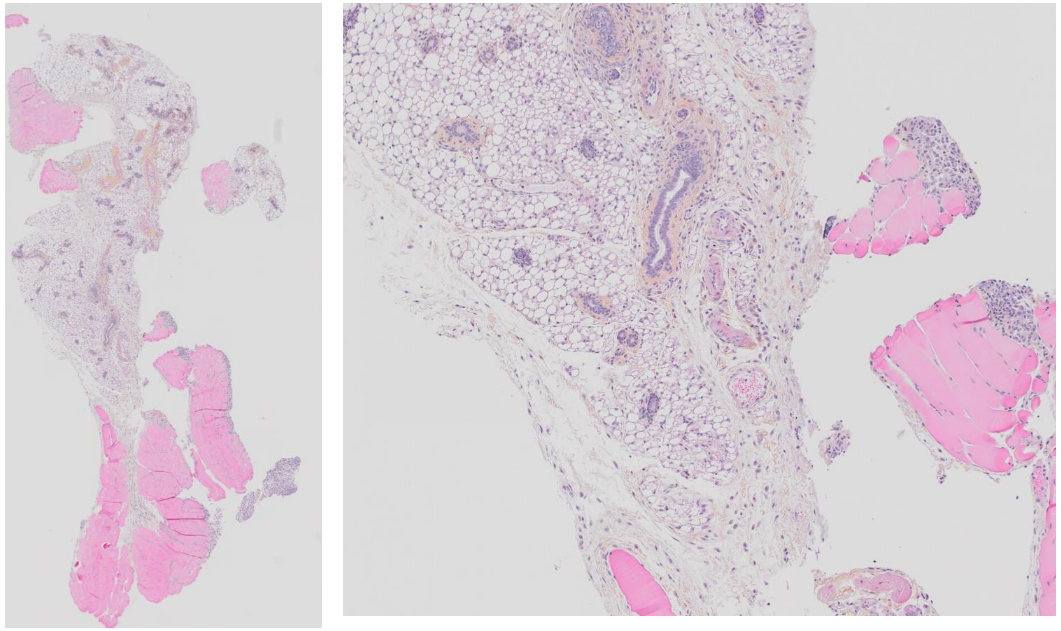

•Probably not the xenograft due to most muscle cells surrounded by muscle and connective tisue and lymphoid tumor cells, but not epithelial cells as in the colon

Supplementary figure 1-33-6. Histology pictures

M33M (spleen)

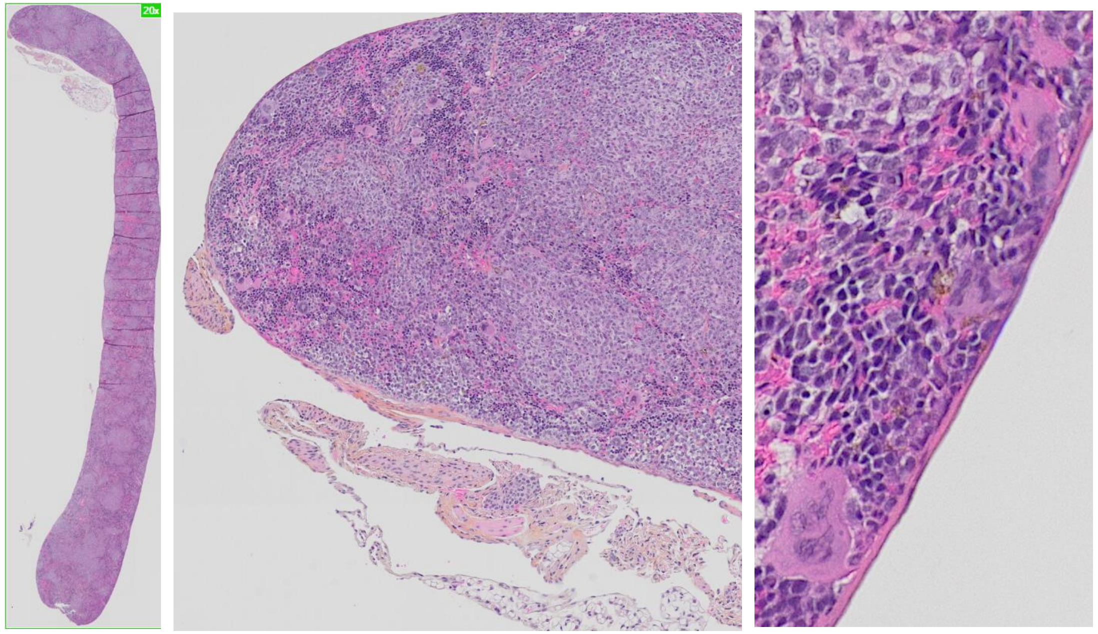

- Plenty lymphoid tumor cells in the spleen
- Measured on 1.3 cm on the slide, 2.56 cm calculated from picture of spleen in petri dish after euthanization
- Red aereas with possible bleedings
- Can see macrophages, giant cells/ghost cells

M33V (tumor left shoulder)

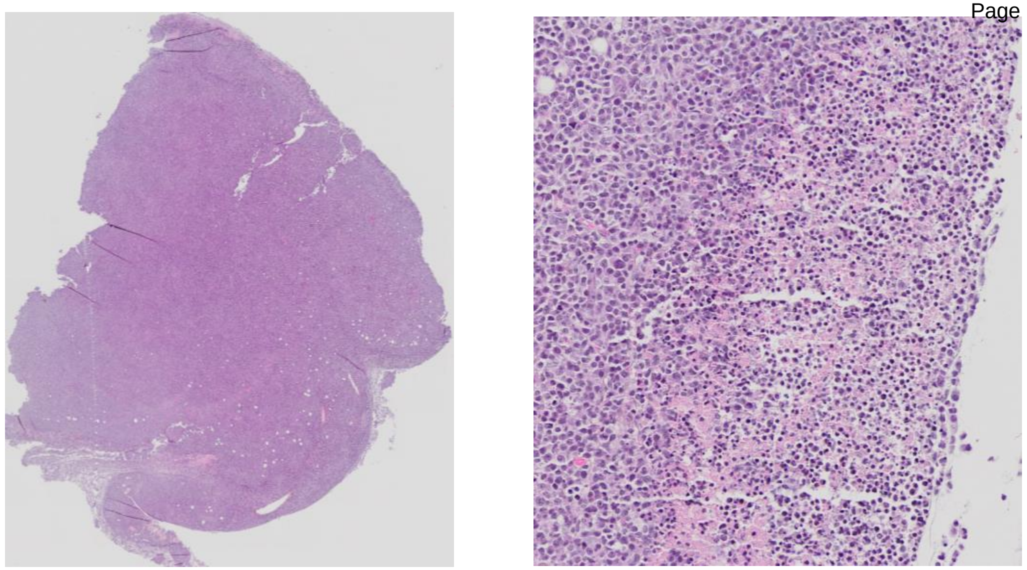

- Plenty lymphoid tumor cells packed dense together
- Smaller aereas with necrosis

M33LK (lymph node?)

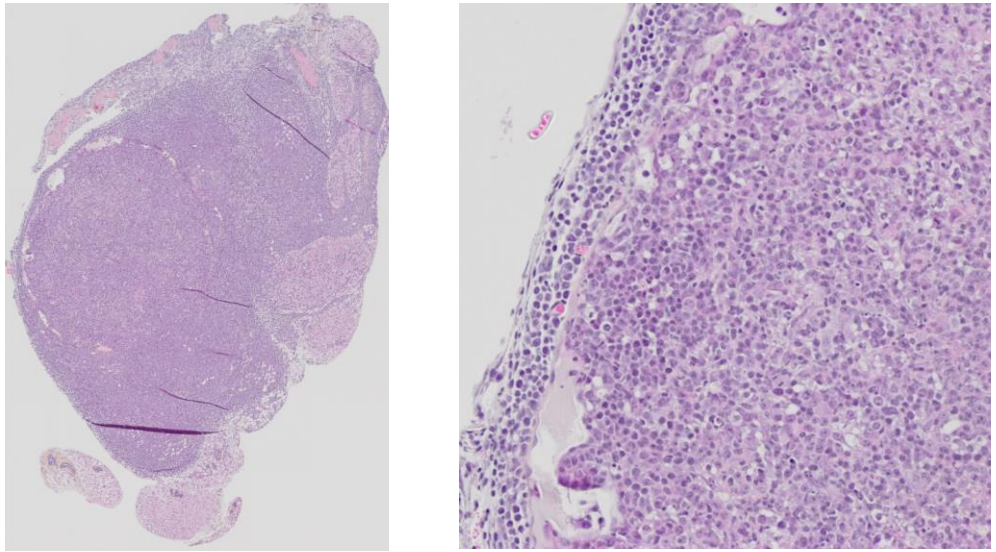

- Plenty lymphoid tumor cells densly packed together

Supplementary figure 1-33-7. Histology and immunohistochemistry pictures

M33V (tumor left shoulder)
-IHC staining for Ki67 and CD45
-EBER-ISH staining for EBV

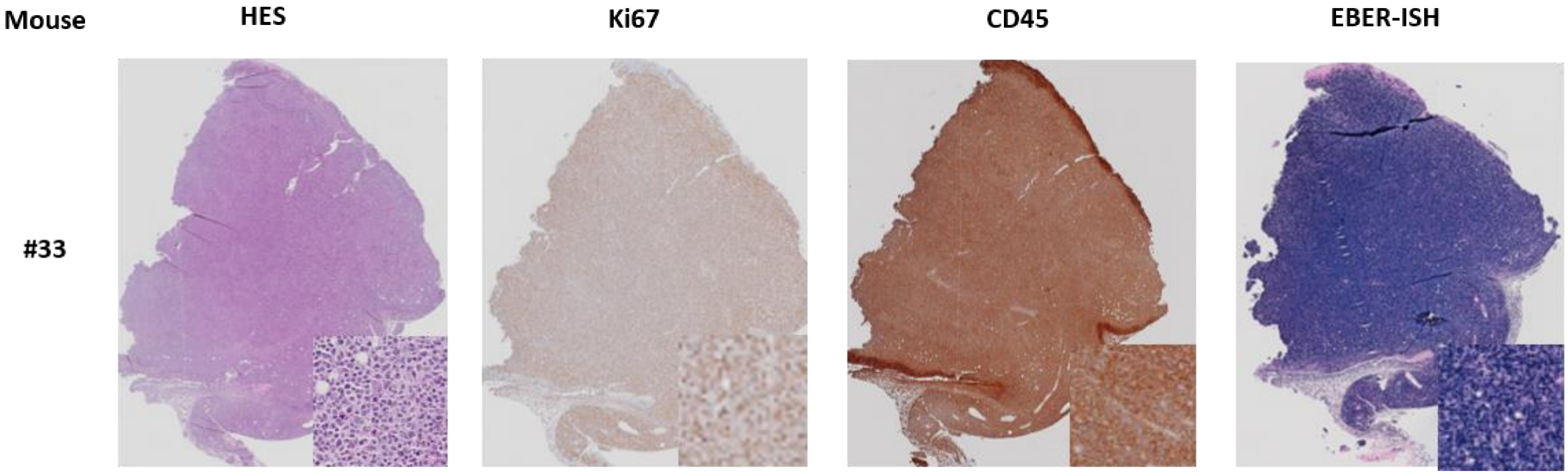

Supplementary file 2

Date: Mouse nr: Tumor: Patient:

**Heating chamber 37°C**	HiBiscrub, Klorhexidin, 4xNaCl	
Wash surface areas with ethanol		
Heating pad 37°C		
**Weight**		
Anasthezia chamber	N_2O_2 60 % - O2 40 % - Isofluran 2%	
Anasthezia mask	N_2O_2 60 % - O2 20 % - Isofluran 1.5-2%	
**Eye gel**		
**Ear mark**		
Shaving		
Change surgery tissue		
Fixate feet with tape		
**Metacam – *systemic anasthezia (~0.1 ml)***	Orange canyle	
Wash surgery area: HiBiscrub		
Wash surgery area: Chlorhexidine		
**Marcain – *local anasthezia (~0.08 ml)***	Yellow canyle	
Pinch foot with forceps		
Make a cut with scalpel		
Use sizzors to make a pocket under the skin		
**Flush tumor fragment with NaCl**		
Stich the wound		
Take a picture of the mouse/surgery area		

Comments:

Exuipment:
Eye gel
Heating pad
2 x Sterile surgery tissues
HiBiscrub
Chlorhexidine
Autoclaved surgery instruments (2 x forceps, 2 x sizzors, Sutur instruments)
2 x Scalpels
4 x sutures (nr 5)
8 x 1 ml syringe + 8 x yellow canyle
2 x white plastic dish for HiBiscrub and ethanolS
Sterilizer for surgery instruments between animals
Sterile q tips
Tape
Ear mark instrument
NaCl
Metacam
Marcain

Performed by: ______________________________

Supplementary figure 2-1. Bullet point form for surgery

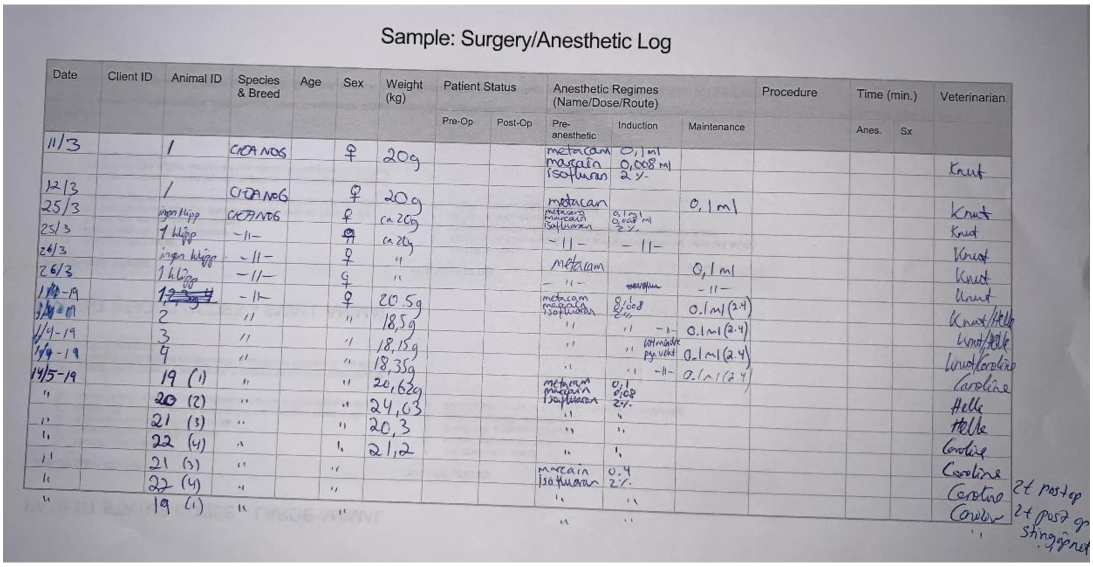

Sample: Surgery/Anesthetic Log

Date	Client ID	Animal ID	Species & Breed	Age	Sex	Weight (kg)	Patient Status: Pre-Op	Patient Status: Post-Op	Anesthetic Regimes (Name/Dose/Route): Pre-anesthetic	Induction	Maintenance	Procedure	Time (min.): Anes.	Sx	Veterinarian
11/3		1	CIEA NOG		♀	20g			metacam marcain isofluran	0,1 ml 0,008 ml 2%					Knut
12/3		1	CIEA NOG		♀	20g			metacan		0,1 ml				
25/3		ingen klipp	CIEA NOG		♀	ca 20g			metacam marcain isofluran	0,1 ml 0,008 ml 2%					Knut
25/3		1 klipp	-11-		♀	ca 20g			-11-	-11-					Knut
26/3		ingen klipp	-11-		♀	"			metacam		0,1 ml				Knut
26/3		1 klipp	-11-		♀	"			-11-	[illegible]	-11-				Knut
1/4-19		1 ~~2, 3, 4~~	-11-		♀	20.5g			metacam marcain isofluran	0,008 2%	0.1 ml (2.4)				Knut
1/4-19		2	"		"	18,5g			"	" -1-	0.1 ml (2.4)				Knut/Helle
1/4-19		3	"		"	18,15g			"	" litt mindre pga vekt	0.1 ml (2.4)				Knut/Helle
1/4-19		4	"		"	18,35g			"	" -11-	0.1 ml (2.4)				Knut/Caroline
14/5-19		19 (1)	"		"	20,62g			metacam marcain isofluran	0,1 0,08 2%					Caroline
"		20 (2)	"		"	24,03									Helle
"		21 (3)	"		"	20,3			"	"					Helle
"		22 (4)	"		"	21,2			"	"					Caroline
"		21 (3)	"		"				marcain isofluran	0,4 2%					Caroline
"		22 (4)	"		"				"	"					Caroline 2t postop
"		19 (1)	"		"				"	"					Caroline 2t post op sting åpnet

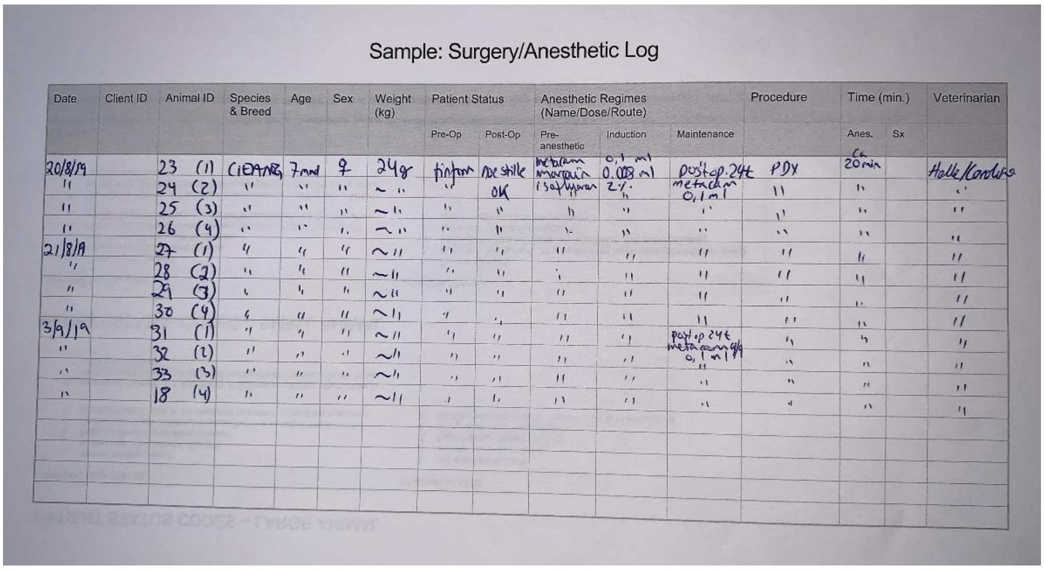

Sample: Surgery/Anesthetic Log

Date	Client ID	Animal ID	Species & Breed	Age	Sex	Weight (kg)	Patient Status: Pre-Op	Patient Status: Post-Op	Anesthetic Regimes (Name/Dose/Route): Pre-anesthetic	Induction	Maintenance	Procedure	Time (min.): Anes.	Sx	Veterinarian
20/8/19		23 (1)	CIEA NOG	7 mnd	♀	24gr	finfin	noe stille OK	metacam marcain isofluran	0,1 ml 0.008 ml 2%	postop. 24t metacam 0,1 ml	PDX	ca 20 min		Helle/Caroline
"		24 (2)	"	"	"	~ "	"			"		"	"		"
"		25 (3)	"	"	"	~ "	"	"	"	"	"	"	"		"
"		26 (4)	"	"	"	~ "	"	"	"	"	"	"	"		"
21/8/19		27 (1)	"	"	"	~ "	"	"	"	"	"	"	"		"
"		28 (2)	"	"	"	~ "	"	"	"	"	"	"	"		"
"		29 (3)	"	"	"	~ "	"	"	"	"	"	"	"		"
"		30 (4)	"	"	"	~ "	"	"	"	"	"	"	"		"
3/9/19		31 (1)	"	"	"	~ "	"	"	"	"	post op 24t metacam 0,1 ml	"	"		"
"		32 (2)	"	"	"	~ "	"	"	"	"	"	"	"		"
"		33 (3)	"	"	"	~ "	"	"	"	"	"	"	"		"
"		18 (4)	"	"	"	~ "	"	"	"	"	"	"	"		"

Supplementary figure 2-2. Picture of anesthesia logs

Suplementary file 3

Procedure for euthanization, blood- and tissue sampling of CIA NOG mice

Human endpoints:

- 8 weeks treatment with Smartfish Remune with fishoil or placebo
- Tumor volume ~1500 mm^3 or >10 % loss of body weight

Procedure:

1. Register mouse weight
2. Put mouse into anasthesia chamber with isofluarane 2% (O_2, N_2O)
3. Place the mouse on the back on a heating pad with surgery tissue and anesthesia mask
4. Take blood sample by heart punctation
   a. Collect full blood in a EDTA tube
   b. Spot 50 µl full blood onto a Whatman filtercard (dry 2 h at RT, freeze -80°C)
   c. Mark blood cards with mouse name and date
5. Centrifuge EDTA tube to prepare blood plasma (4°C, 3000 rpm, 10 min)
6. Meassure tumor size
7. Shave and wash (EtOH) the tumor area
8. Cut out the PDX using a scalpel
   a. Remove necrotic tissue/connective tissue
   b. Take 4 samples from the tumor (Histology prioritized if small PDX)
      i. 4x4x4 mm for histology in a tube with 4% NBF
      ii. 4x4x4 mm for RNA-sekvensering in a cryotube (snapfreeze N_2, then -80°C)
      iii. x4x4 for protein isolation in cryotubes (snapfreeze in N_2, then -80°C)
      iv. The rest of the PDX is frozen in fragments in cryotube (snapfreeze N_2, -80°C)
9. Wash (EtOH) and open the abdomen
10. Cut the aorta
11. Shut of the anastesia gas
12. Perform necroscopy
    a. Organs are sampled on tubes with 4% NBF
13. Fill out autopsy card
14. Pipett the blood plasma into a sterile tube and freeze at -80°C
15. Mark plasma tubes (mouse ID, plasma, date and initials

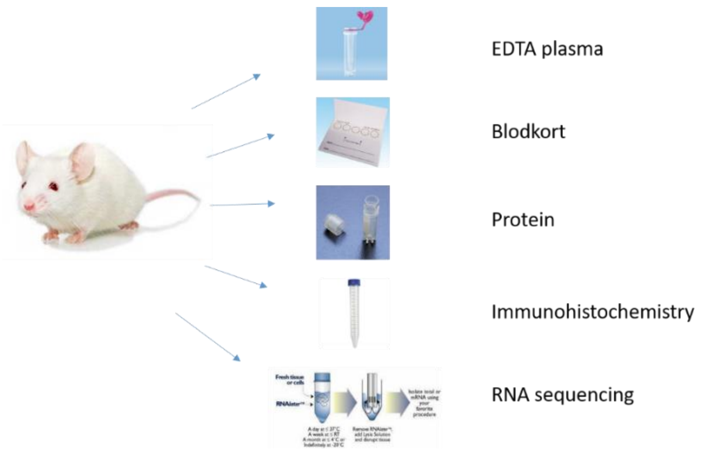

Supplementary material 4: Mice weight curves

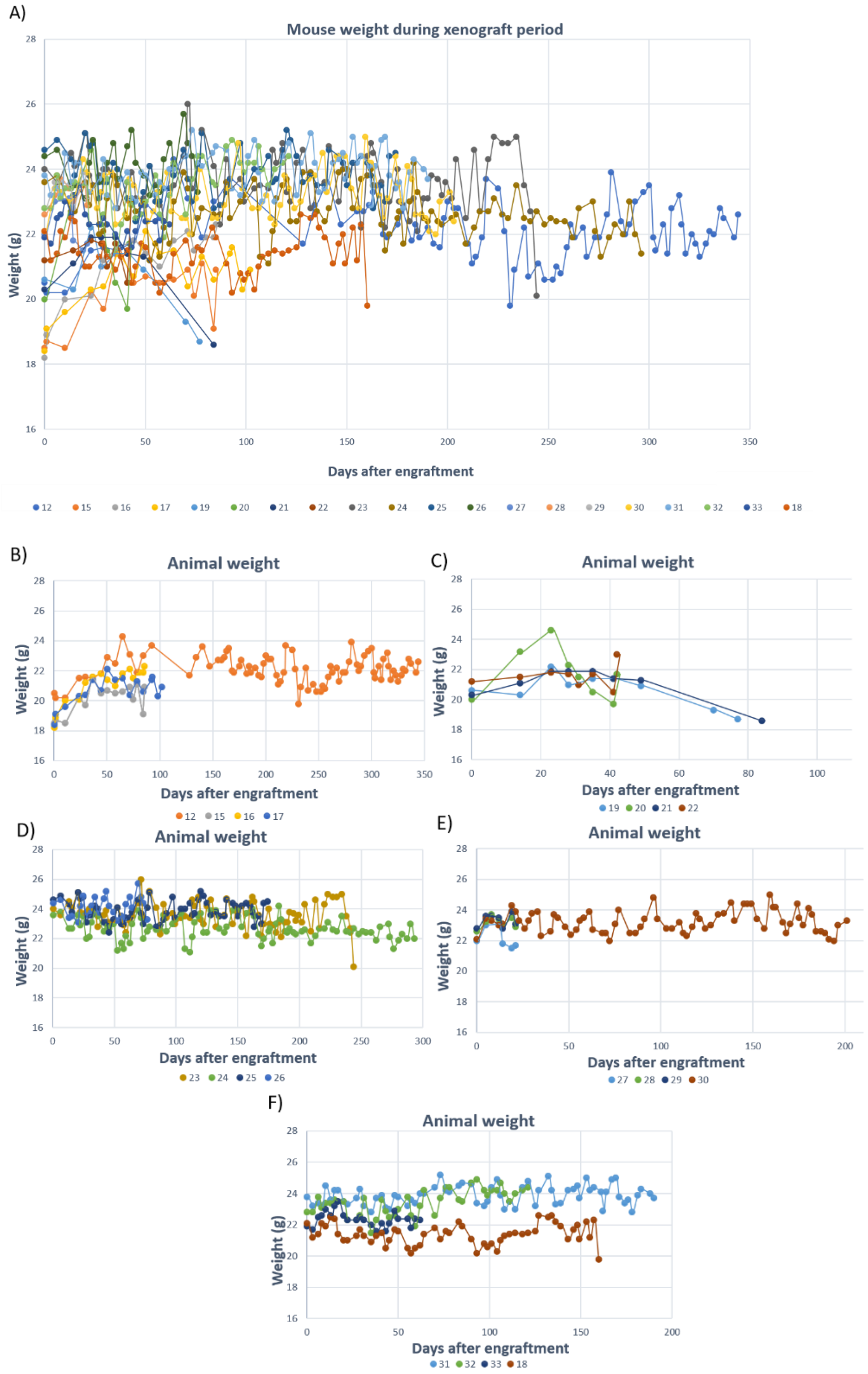

Supplementary figure 4-1. Animal weight curves for A) all mice B) mouse #12, 15, 16 and 17 (tumor fragments from patient 3), C) mouse #19, 20, 21 and 22 (tumor fragments from patient 11), D) mouse #23, 24, 25 and 26 (tumor fragments from patient 17), E) mouse #27, 28, 29 and 30 (tumor fragments from patient 18), and F) mouse #31, 32, 33 and 18 (tumor fragments from patient 19).

Supplementary file 5

Supplementary Table 5-1. Fatty acid profiling of patient and mouse whole blood

Sample ID	Tumor fragment	Treatment status	Fatty acid methyl ester content (g FAME / 100 g FAME)											
			C16:0 PA	C18:0 SA	C18:1 c-9 OA	C18:2 n-6 LA	C18:3 n-6 GLA	C18:3 n-3 ALA	C20:3 n-6 DGLA	C20:4 n-6 AA	C20:5 n-3 EPA	C22:5 n-3 DPA	C22:6 n-3 DHA	Omega-3 index
P3			25.7	16.2	23.7	19.7	0.08	0.39	1.17	8.13	0.85	1.05	2.89	6.12
P11			29.0	12.9	24.6	22.0	0.10	0.60	1.18	5.97	0.91	0.80	1.90	4.99
P17			31.3	13.0	24.1	19.0	0.28	0.44	0.97	4.89	**2.02**	0.92	**3.07**	8.00
P18			26.7	15.8	24.3	19.1	0.20	0.30	1.28	8.15	0.83	1.13	2.29	5.35
P19			29.6	14.8	26.2	17.4	0.19	0.33	1.58	6.88	0.47	0.89	1.64	4.06
M12	P3-1a	No	34.9	14.5	18.6	14.8	0.16	0.13	0.72	12.9	0.10	0.35	2.81	4.98
M15	P3-1b	Start	35.4	15.3	15.9	18.0	0.16	0.20	0.67	11.9	0.07	0.25	2.25	4.24
M15	P3-1b	PLACEBO	35.3	12.9	20.4	17.4	0.18	0.18	0.98	9.45	0.24	0.39	2.66	4.99
M16	P3-2a	Start	34.7	15.6	15.8	17.2	0.16	0.19	0.71	12.5	0.08	0.32	2.71	4.98
M16	P3-2a	OMEGA-3	33.1	15.0	18.1	17.1	0.12	0.19	1.03	7.30	2.02	0.71	5.34	10.80
M17	P3-2b	Start	33.8	14.2	19.8	17.9	0.17	0.36	0.65	10.5	0.09	0.31	2.24	4.26
M18	P19-2b	No	35.6	13.6	17.2	18.9	0.20	0.34	0.53	11.0	0.07	0.22	2.36	4.37
M19	P11-1a	OMEGA-3	32.1	15.5	16.6	12.4	0.18	0.15	0.80	8.72	6.30	0.77	6.43	17.9
M20	P11-1b	Start	32.9	15.5	15.3	16.2	0.15	0.12	0.67	15.3	0.06	0.35	3.43	5.66
M20	P11-1b	PLACEBO	35.4	11.7	22.0	19.8	0.17	0.18	0.72	7.68	0.19	0.25	1.93	4.01
M22	P11-2b	Start	33.0	16.1	14.5	15.6	0.13	0.11	0.83	15.8	0.09	0.37	3.47	5.75
M22	P11-2b	OMEGA-3	35.3	12.1	23.3	18.2	0.15	0.20	0.83	7.47	0.17	0.23	2.01	4.09
M23	P17-1a	Start	34.7	14.6	19.5	15.4	0.14	0.14	0.69	12.1	0.09	0.26	2.46	4.53
M23	P17-1a	PLACEBO	30.5	15.2	22.4	14.5	0.14	0.11	1.07	10.7	0.50	0.49	4.48	7.62
M24	P17-1b	No	34.1	14.7	15.9	15.5	0.12	0.14	0.82	15.2	0.10	0.25	3.20	5.43
M25	P17-2a	No	35.6	15.9	16.3	15.7	0.20	0.18	0.74	12.1	0.12	0.41	2.75	4.93
M26	P17-2b	Start	35.6	14.2	18.5	16.3	0.16	0.20	0.69	11.5	0.10	0.28	2.48	4.57
M26	P17-2b	OMEGA-3	32.3	14.7	19.2	17.1	0.15	0.22	0.91	9.45	0.31	0.64	5.07	8.09
M27	P18-1a	Start	34.3	16.0	14.7	15.7	0.16	0.15	0.92	14.2	0.11	0.36	3.38	5.68
M27	P18-1a	OMEGA-3	32.9	15.8	16.8	17.3	0.21	0.17	0.97	12.1	0.17	0.36	3.20	5.56
M28	P18-1b	Start	34.0	16.3	15.2	14.6	0.16	0.11	0.84	14.7	0.11	0.40	3.63	6.00
M28	P18-1b	PLACEBO	33.2	15.7	17.2	17.6	0.18	0.23	0.71	10.8	0.26	0.45	3.73	6.33
M29	P18-2a	OMEGA-3	33.5	15.2	17.7	18.4	0.10	0.24	0.77	7.91	1.60	0.49	4.14	8.72
M30	P18-2b	No	32.2	16.9	15.9	14.9	0.12	0.12	0.78	15.7	0.08	0.31	3.14	5.35
M31	P19-1a	No	33.8	15.7	17.2	13.5	0.13	0.11	0.76	15.1	0.07	0.34	3.28	5.51
M33	P19-2a	No	34.0	14.8	18.4	17.6	0.19	0.27	0.69	11.5	0.07	0.21	2.32	4.33
Average untreated mice			34.29	15.24	16.79	16.11	0.16	0.18	0.73	13.25	0.09	0.31	2.87	5.03
Standard deviation			1.02	0.91	1.70	1.46	0.03	0.08	0.09	1.84	0.02	0.06	0.49	0.60
Average Smartfish treated			33.20	14.72	18.62	16.75	0.15	0.20	0.89	8.83	1.76	0.53	4.37	9.19
Standard deviation			1.15	1.34	2.48	2.20	0.04	0.03	0.10	1.80	2.36	0.21	1.59	4.88
Average Placebo treated			33.60	13.88	20.50	17.33	0.17	0.18	0.87	9.66	0.30	0.40	3.20	5.74
Standard deviation			2.30	1.89	2.36	2.17	0.02	0.05	0.18	1.45	0.14	0.11	1.13	1.57

P=patient, M=mouse, FAME=fatty acid methyl ester, No=no treatment given, OMEGA-3=Smartfish Remune with fish oil, PLACEBO=Smartfish Remune Placebo with rapeseed oil

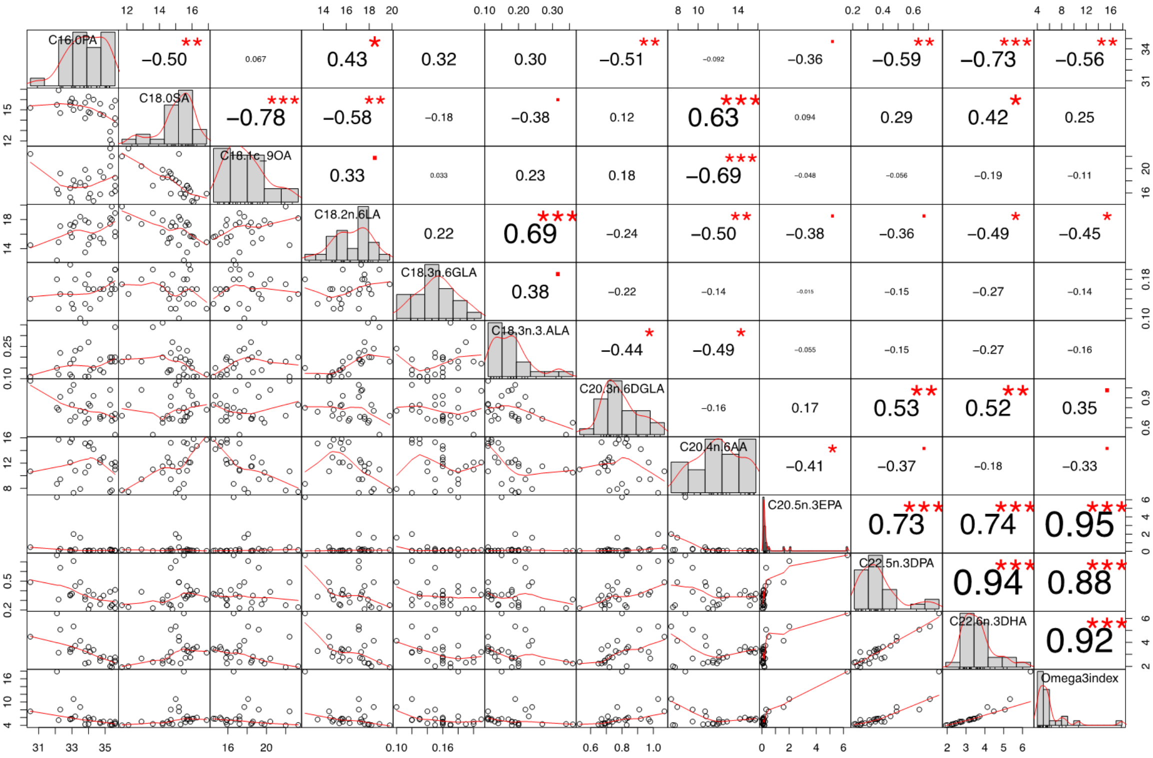

Supplementary figure 5-1. Correlation in values for different fatty acids measured in mouse whole blood.
